# Photochemical Deracemization of 4,7-Diaza-1-isoindolinones by Unidirectional Hydrogen Atom Shuttling

**DOI:** 10.1021/jacs.4c16053

**Published:** 2024-12-30

**Authors:** Philip Freund, Mike Pauls, Daria Babushkina, Thomas Pickl, Christoph Bannwarth, Thorsten Bach

**Affiliations:** † School of Natural Sciences, Department Chemie, and Catalysis Research Center (CRC), Technische Universität München, Lichtenbergstrasse 4, 85747 Garching, Germany; ‡ Institut für Physikalische Chemie, RWTH Aachen University, 52074 Aachen, Germany

## Abstract

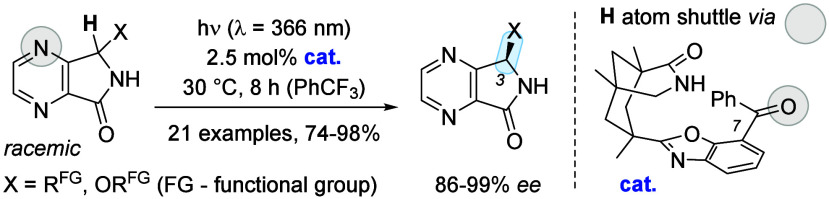

By coupling a photochemical and a thermal step, a single chiral catalyst can establish a photostationary state in which the enantiopure form of a chiral compound is favored over its racemate. Following this strategy, 3-substituted 4,7-diaza-1-isoindolones were successfully deracemized (74–98% yield, 86–99% *ee*) employing 2.5 mol % of a photocatalyst. Key to the success of the reaction is the fact that a chiral benzophenone recruits selectively one enantiomer of the substrate for a photoinduced hydrogen atom transfer. A combination of computational and experimental studies suggests that the hydrogen atom is shuttled via the oxygen atom of the catalyst to the 4-nitrogen atom of the substrate.

A single stereogenic center renders a molecule chiral; i.e., it is nonsuperimposable onto its mirror image. The two stereoisomeric forms are called enantiomers. Under thermal conditions in homogeneous solution, a 1:1 mixture of enantiomers (a racemate) cannot be converted into a single enantiomer of the same compound. The racemate is entropically favored,^1^ and the equilibrium cannot be shifted by a single catalyst.^2^ However, if a single chiral catalyst operates by two distinct pathways, i.e., a photochemical and a thermal step, the principle of microscopic reversibility is not violated and a photostationary state can be reached in which one enantiomer prevails.^3,4^ For the photochemical editing of stereogenic centers in organic compounds, reversible C–H bond cleavage has been identified as a powerful tool, given the abundance of tertiary stereogenic carbon centers.^5^ So far, most catalytic systems for the photochemical deracemization at stereogenic centers with a C–H bond have employed a dual catalytic system, in which one catalyst is responsible for hydrogen atom transfer (HAT) and the second for creating or restoring the stereogenic center.^6,7^

Examples for the use of a single chiral catalyst^8,9^ are limited to chiral benzophenone catalyst **1a**.^10^ Its mode of action in the deracemization of hydantoins has recently been elucidated^11^ and is shown in Scheme 1. Two key features are particularly relevant: (a) The catalyst processes selectively one of the two enantiomers, here the (*S*)-enantiomer. Hydrogen abstraction in the 1:1 substrate/catalyst complex **I** occurs once the benzophenone is promoted to its reactive *n*π* triplet state T_1_ by excitation and intersystem crossing (ISC). (b) Since the back HAT in complex **II** does not occur to the carbon center but rather to an oxygen atom, achiral enol intermediate **IV** is formed. Upon dissociation from the catalyst in complex **III**, enol intermediate **IV** converts to either enantiomer through keto-enol tautomerism with a statistical outcome. With the (*R*)-enantiomer not being processed, high enantioselectivities for the respective hydantoins have been recorded. The hydrogen atom (in bold) moves in one direction from the substrate to the final (*R*)-product via a protonated ketyl radical and enol **IV**.

**Scheme 1:**
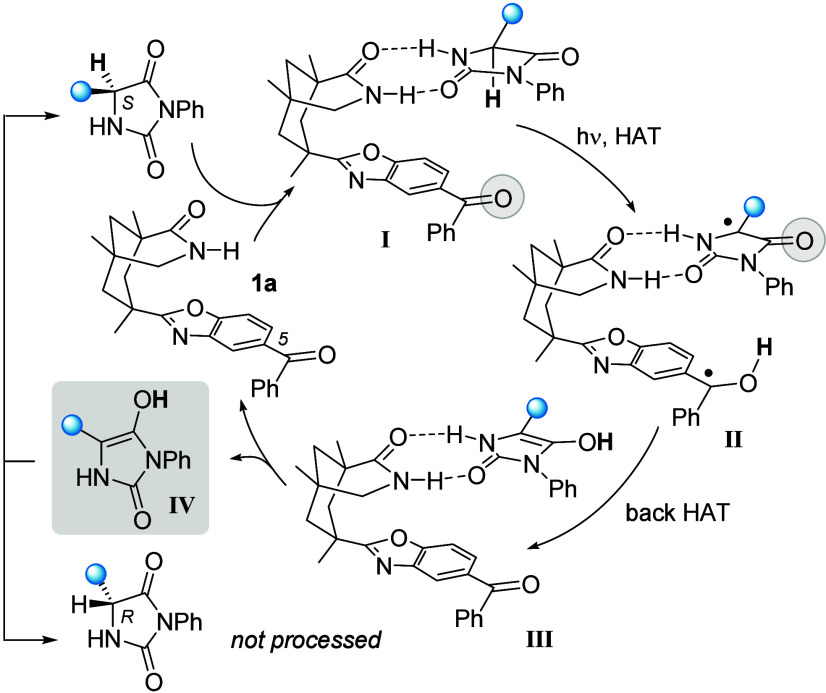
Photochemical Deracemization of Hydantoins by a Single Chiral Catalyst *via* an Enol Intermediate IV

Although this approach can be extended to substrates displaying a stereogenic center in the α-position to the lactam carbonyl group, the reactions require a higher catalyst loading.^10b,10c^ In an ideal setting with catalyst loadings of 5 mol % and below, back HAT should occur to a heteroatom not involved in binding. We hypothesized that a nitrogen atom might be competent to act as a hydrogen atom acceptor and enamines might be formed as short-lived achiral intermediates. We identified 3-substitued 4,7-diaza-1-isoindolinones as ideal substrates for such an endeavor with the potential to not only access this important compound class^12^ enantioselectively but also several chiral pyrazines^13^ by appropriate downstream chemistry.

Preliminary experiments commenced with racemic diazaisoindolinone *rac*-**2a**. Inspection of molecular models and initial calculations suggested that the photoactive benzoyl group, which was in the 5-position of the benzoxazole for catalyst **1a**, should be preferably translocated to the 7-position. Catalyst **1b** (Table 1) was, thus, synthesized from Kemp’s triacid^14^ and 3-amino-2-hydroxybenzophenone (see the Supporting Information for details) and purified by chiral HPLC. From the very first trials, the catalyst performed outstandingly well in the deracemization reaction. With a loading of 5 mol % (entry 1), the photostationary state was reached after 4 h delivering the product in 79% yield and with 98% enantiomeric excess (*ee*). Monitoring the *ee* over time with a catalyst loading of 2.5 mol % revealed the deracemization to be complete after 8 h with an improved yield of 90% (entry 2). Further lowering of the catalyst loading did not improve the outcome of the deracemization (entry 3; see the Supporting Information for a complete optimization table). Remarkably, catalyst **1a** failed to achieve any meaningful enantioselectivity (entry 4) emphasizing the importance of positioning the benzoyl group properly within the catalyst backbone. A solvent screen (entries 5–7) confirmed the superiority of trifluorotoluene as a reaction medium. Irradiation at shorter wavelengths was hampered by substrate decomposition (entry 8) notably compromising the yield.

**Table 1:**
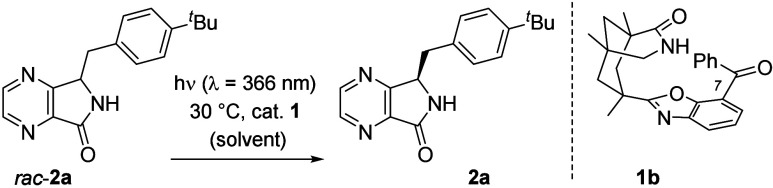
Photochemical Deracemization of 4,7-Diazaisoindolinone *rac*-**2a** Catalyzed by Chiral Benzophenones: Optimization of the Reaction Conditions

entry^a^	cat.	mol % **1**	solvent	yield (%)^b^	*ee* (%)^c^
1^d^	**1b**	5	PhCF_3_	79	98
2	**1b**	2.5	PhCF_3_	90	98
3	**1b**	1	PhCF_3_	91	86
4	**1a**	2.5	PhCF_3_	92	2
5	**1b**	2.5	MeCN	80	10
6	**1b**	2.5	PhCH_3_	58	70
7	**1b**	2.5	acetone	74	18
8^e^	**1b**	2.5	PhCF_3_	66	93
9^f^	**1b**	2.5	PhCF_3_	96	3

a The reaction mixture was irradiated under the indicated conditions for 8 h. The wavelength λ is provided as the emission maximum of the light source.

b Yields refer to isolated product.

c Determined by chiral HPLC analysis.

d Irradiation time: 4 h.

e The reaction was performed at λ = 350 nm.

f The *N*-methylated substrate was used (*rac*-**2a**-Me).

The two-point hydrogen bonding event between catalyst and substrate is critical for the success of the reaction, as shown by the poor performance of *N*-methylated substrate *rac*-**2a**-Me (entry 9, Figure 1). A further control was performed with 3-benzyl-1-isoindolinone (*rac*-**3**) as a substrate that lacks nitrogen atoms in the benzo ring. Here, the material was recovered in racemic form when subjected to the conditions of entry 2 (94%, 6% *ee*).

**Figure 1:**
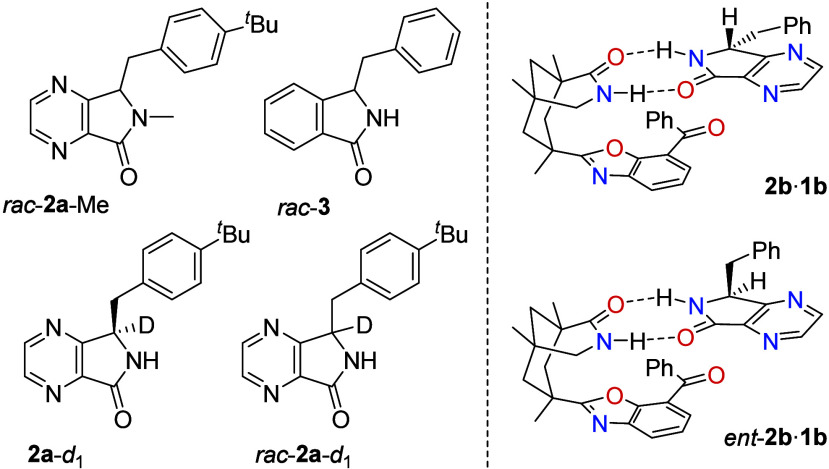
Structures of deracemization substrates (left) and of diastereomeric complexes **2b**·**1b** and *ent*-**2b**·**1b** (right).

Having identified optimal conditions for the deracemization of 4,7-diaza-1-isoindolinones, we employed different substrates in the established protocol (Scheme 2). A set of 3-arylmethyl-substituted compounds *rac*-**2a** to *rac*-**2i** delivered consistently good results, establishing the reaction to be compatible with fluoro, bromo, chloro, alkoxy, alkoxycarbonyl, and furyl groups. Enantioenriched product **2d** was further purified by chiral HPLC and the enantiopure material delivered crystals suitable for single crystal X-ray crystallography (see Supporting Information for details). Anomalous diffraction revealed the compound to be (*R*)-configured and the assignment of the absolute configuration for the other products was based on analogy. In the alkyl series (substrates *rac*-**2j** to *rac*-**2m**), the enantioselectivities remained extremely high, varying between 95% and 98% *ee*. 3-Oxygen-substituted 4,7-diaza-1-isoindolinones are difficult to obtain enantioselectively because the corresponding hydroxy compound, which is an *N*,*O*-hemiacetal, racemizes readily.^15^ With the deracemization approach, their preparation is facile, and eight representative products **2n** to **2u** were prepared from the respective racemates (74–98% yield, 86–99% *ee*). The reactions confirmed the deracemization to be compatible with chloro, alkenyl, ether, trifluoromethyl, and silyl substituents within the chain. Apart from limitations inherent to the benzophenone chromophore^16^ and related to oxidation-sensitive functional groups, the only other limitation was found to be the solubility of a given substrate in trifluorotoluene. If the solution is not homogeneous, hydrogen bonding to the catalyst is hampered, avoiding the required differentiation between the two enantiomers. While the solubility issue could be mitigated for selected substrates by using solvent mixtures of trifluorotoluene with acetonitrile or acetone, the yields were found to be lower (see Supporting Information for details). The deracemization can readily be scaled and allows processing larger quantities. Runs with both 0.2 (*rac*-**2l**) and 1.0 mmol (*rac*-**2t**) of substrate were conducted successfully.

**Scheme 2:**
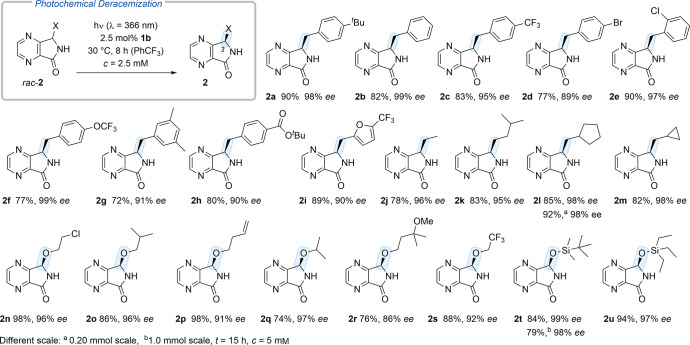
Catalytic Photochemical Deracemization of 4,7-Diazaisoindolinones (25 μmol Scale)

Mechanistic experiments were performed with 4,7-diaza-1-isoindolinones monodeuterated at positions C3. It was found by crossover experiments that no deuterium was exchanged if enantiomerically pure (*R*)-isomers **2a**-*d*_1_ (Figure 1) and **2j** were subjected to the reaction conditions. A hydrogen/deuterium scrambling of approximately 25% was found when the same reaction was performed with *rac*-**2a**-*d*_1_ and *rac*-**2j** (see the Supporting Information for details). The results suggest that the (*R*)-isomers are not processed by the catalyst and only the respective (*S*)-enantiomers *ent*-**2** undergo HAT within the complex *ent*-**2**·**1b**.

Computational studies were exemplarily performed for compounds **2b** and *ent*-**2b** to elucidate the further course of the reaction (Figure 2). The calculated free energies already account for dimerization of the pure species (Table S7), since dimerization was found to be exergonic for the substrates and catalyst. For the formation of the *ent-***2b**·**1b** complex, an association energy of −7 kJ·mol^–1^ is obtained, whereas the formation of **2b**·**1b** is only slightly exergonic with −1 kJ·mol^–1^. Hence, the formation of complex **2b**·**1b** is less favorable, and the association of **1b** with *ent-***2b** is preferred. In the thermodynamically most stable conformation of both complexes (Figure S13), two-point hydrogen bonding to catalyst **1b** is observed. However, only *ent-***2b** coordinates to the photocatalyst in a way that enforces the hydrogen atom on the stereogenic carbon center to point toward the benzophenone moiety. The arrangement allows for HAT via a photochemically induced pathway (see below). No such reaction coordinate is possible within complex **2b**·**1b**. Our calculations also indicate a less favorable arrangement for catalyst **1a** (Figure S15), in line with the experimentally observed lack of activity of this catalyst in the deracemization of *rac-***2a**. Upon irradiation with light (λ = 366 nm), the lowest excited singlet states (S_1_, S_2_) of *ent-***2b**·**1b** are energetically accessible (Figures S17, S19). They correspond to fragment-localized *n* → π* excitations on the respective moieties *ent-***2b** and **1b** in the *ent*-**2b**·**1b** complex (Figure S18). Due to the orbital symmetry, the substrate-localized *n* → π* excitation has a sizable oscillator strength of 0.0083 and can be directly excited. ISC to the corresponding energetically proximate triplet states (T_1_, T_2_) appears feasible (Figure S19), and the existence of triplet states is confirmed experimentally. Our quantum chemical calculations suggest that forward HAT occurs from the substrate-localized T_1_ state (Figure S20), and the photochemical conversion proceeds via a transition state (TS) showing a tiny barrier of +4 kJ·mol^–1^. In this step, the bond distance between the involved OH atom pair is reduced from 2.49 Å at the T_1_ minimum to 1.48 Å within the TS. After HAT, the achiral triplet intermediate **4b** is obtained (Figure 2). Complex **4b**·**1b′** displays hydrogen bonding interactions between the OH group of the protonated ketyl radical **1b′** and the nitrogen atom in the 4-position (Figure S14). Through reverse ISC (rISC) from the T_1_ to the S_0_ potential energy surface, the hydrogen back transfer to the substrate becomes possible, while reforming a closed-shell species again. Other positions (including the carbonyl oxygen atom at C1 and carbon atom C7a) for the return HAT have been identified (Figure S16) but the resulting products are energetically disfavored relative to enamine **5b**. Therefore, the main product of the photochemical conversion is **5b**·**1b**. Tautomerization to enantiomers **2b** and *ent*-**2b** is expected to occur statistically after dissociation of enamine **5b** from the complex.^21^

**Figure 2:**
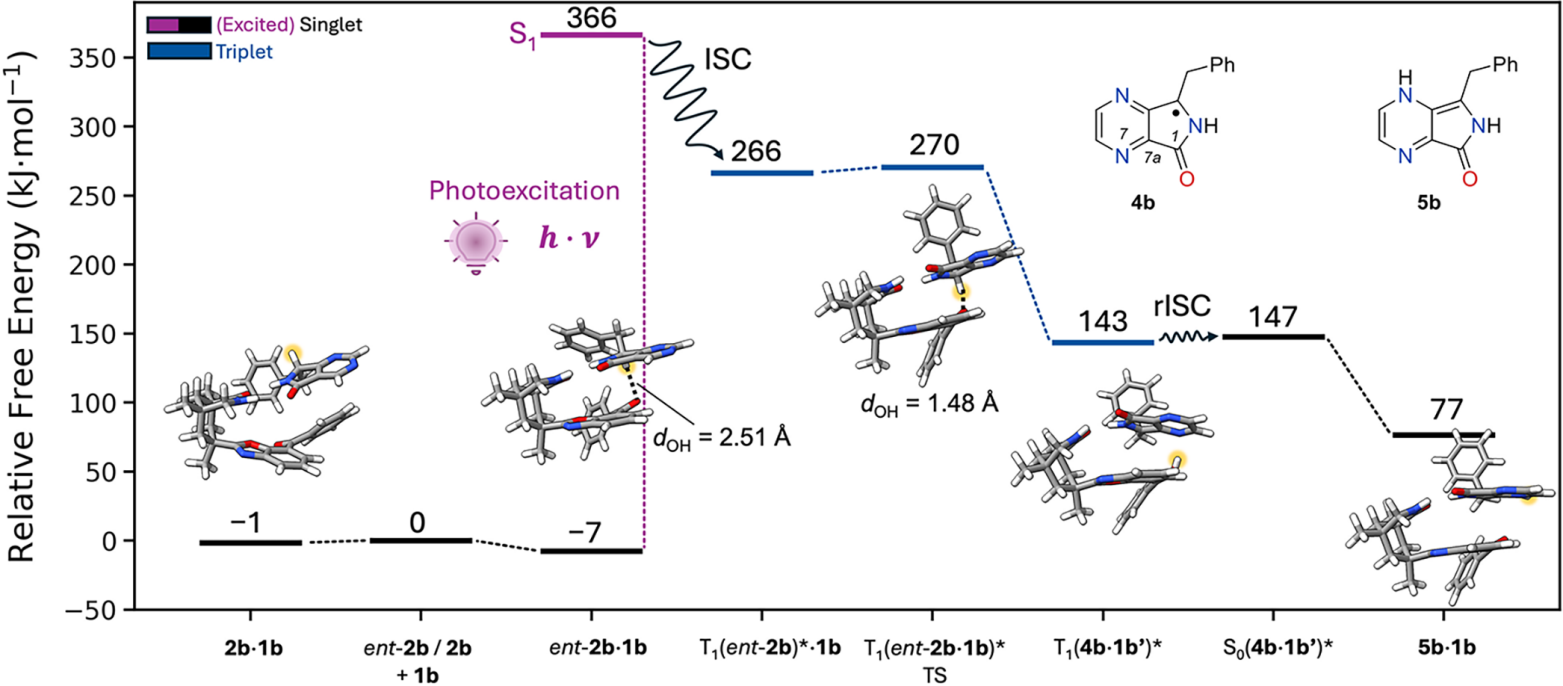
Computed free energy reaction profile for the photocatalytic deracemization of *rac*-**2b**. Electronic energies are calculated at the PW6B95-D4/def2-QZVPP//PBEh-3c^17−20^ level of theory (see the SI for details on the free energy contributions). Relative free energies are given relative to the most stable dimer species of *rac-***2b** and *rac-***1b** in the electronic ground state. The reactive hydrogen atom is highlighted in yellow. The open-shell singlet S_0_(**4b**·**1b**′)* is structurally indistinguishable from the shown T_1_(**4b**·**1b**′)*.

The enantioenriched 4,7-diazaisoindolinones **2** obtained by photochemical deracemization invite consecutive reactions, some of which were preliminarily explored (Scheme 3). Lactam ring opening of **2l** was facile after protection with a *tert*-butoxycarbonyl (Boc) group. Product **6** could be saponified to the respective carboxylic acid **7**, which is a valuable intermediate for further transformations. Decarboxylation^22^ led to pyrazine **8** with a stereogenic center in the α-position to the heterocyclic core. Esterification with trimethylsilyl(TMS)diazomethane delivered ester **9** in a high yield. Chloroethoxy-substituted isoindolinone **2n** enabled an intramolecular alkylation^23^ to oxazolidine **10**, which was readily accomplished with base under phase transfer conditions. Ullmann coupling (CuI, *N*,*N*′-dimethylethylendiamine, K_3_PO_4_) of compound **2t** (TBS = *tert*-butyldimethylsilyl) with 4-chloro-2-iodopyridine furnished product **11** in 66% yield.^24^

**Scheme 3:**
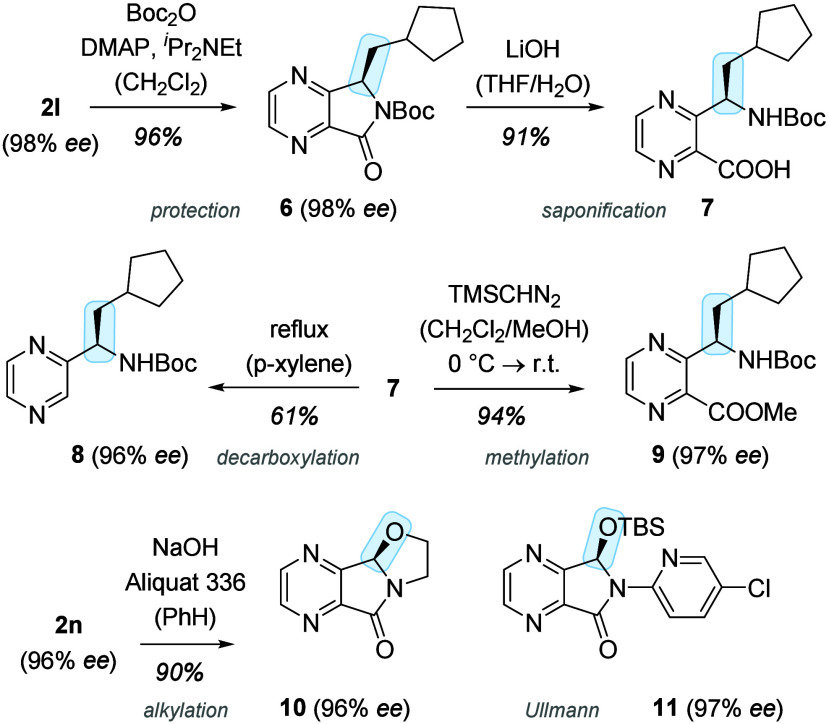
Consecutive Reactions of Enantioenriched 4,7-Diazaisoindolinones: The Absolute Configuration at C3 Was Retained

In summary, a conceptually new approach to photochemical deracemization has been established. It employs benzophenone catalyst **1b** that triggers upon excitation a HAT cascade which commences by an abstraction from the stereogenic carbon atom C3 of substrates *ent*-**2**. The hydrogen atom is redelivered from the catalyst to the N4 nitrogen atom of the substrate leading to an enamine intermediate which tautomerizes either to **2** or *ent*-**2**. With enantiomer **2** not being processed, a photostationary state is reached in which this enantiomer prevails. The remarkably low catalyst loading (2.5 mol %) suggests an efficient HAT within complex *ent*-**2**·**1b**, which might be related to the almost degenerate triplet states involved in the event.

## Supporting Information Available

Experimental procedures, analytical data and NMR spectra for all new compounds, HPLC traces of all compounds (racemates, enantioenriched products, consecutive products), optimization and mechanistic experiments, details of the quantum chemical calculations (PDF)
Optimized geometries of the molecules considered in the computational study (ZIP)
NMR data for compounds **1b**, **2**, **6**–**11** (ZIP)
