# Correction to “Photochemical Deracemization of 4,7-Diaza-1-isoindolinones by Unidirectional Hydrogen Atom Shuttling”

**DOI:** 10.1021/jacs.5c12600

**Published:** 2025-09-09

**Authors:** Philip Freund, Mike Pauls, Daria Babushkina, Thomas Pickl, Christoph Bannwarth, Thorsten Bach

In the original article, Figure 2 erroneously showed a 3-benzyl-4,6-diaza-1-isoindolinone instead of the investigated 3-benzyl-4,7-diaza-1-isoindolinone (*ent*-**2b**) as the substrate for catalyst **1b**. A corrected version of Figure 2 is attached. The computed values reported in the article were not affected and correctly refer to compound *ent*-**2b** and the products derived from it.

**Figure 2:**
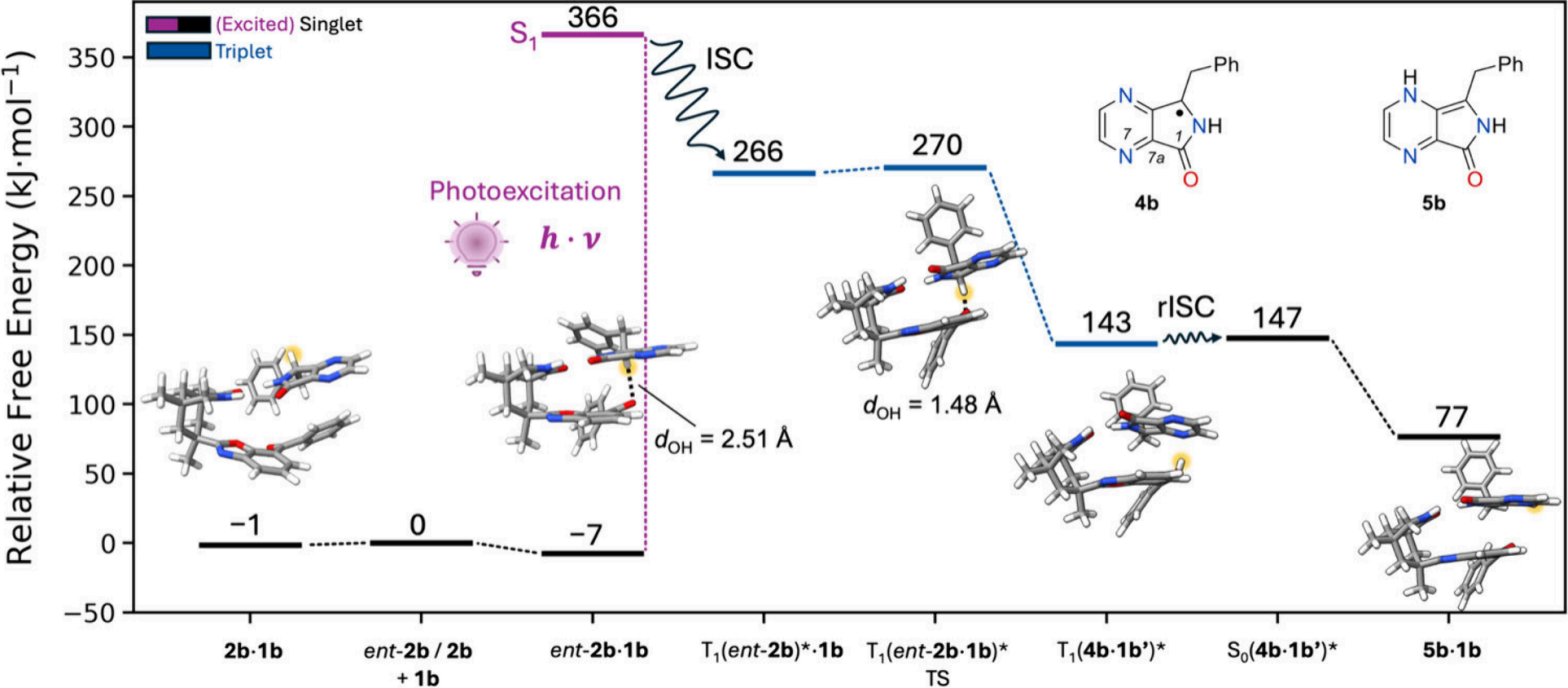
Computed free energy reaction profile for the photocatalytic deracemization of *rac*-**2b**. Electronic energies are calculated at the PW6B95-D4/def2-QZVPP//PBEh-3c level of theory (see the SI for details on the free energy contributions). Relative free energies are given relative to the most stable dimer species of *rac-* **2b** and **1b** in the electronic ground state. The reactive hydrogen atom is highlighted in yellow. The open-shell singlet S_0_(**4b·1b′**)* is structurally indistinguishable from the shown T_1_(**4b·1b′**)*.

In the calculations presented in the Supporting Information (SI), 3-benzyl-4,6-diaza-1-isoindolinone was erroneously taken as the substrate for catalyst **1a**. The calculations have now been repeated for substrate *ent*-**2b**, and the SI has been updated accordingly. The corrected SI (changed parts shaded in gray) and the corrected xyz files are available herein.

These corrections do not affect the scientific content nor the conclusions presented in the published article.

## Supplementary Material





## Notes

Experimental procedures, analytical data and NMR spectra for all new compounds, HPLC traces of all compounds (racemates, enantioenriched products, consecutive products), optimization and mechanistic experiments, details of the quantum chemical calculations (corrected) (PDF)
Optimized geometries of the molecules considered in the computational study (corrected) (ZIP)

