# Supplementary material for: Correction to “Photochemical Deracemization of 4,7-Diaza-1-isoindolinones by Unidirectional Hydrogen Atom Shuttling”
Source: J Am Chem Soc. 2025 Sep 9;147(37):34181–2. doi: 10.1021/jacs.5c12600 (PMC12447476; doi:10.1021/jacs.5c12600)
Supplement: Supplementary file 1 [file ja5c12600_si_001.pdf]

Supporting Information for

Photochemical Deracemization of 4,7-Diaza-1-isoindolinones by Unidirectional Hydrogen Atom Shuttling

Philip Freund,[a] Mike Pauls,[b] Daria Babushkina,[b] Thomas Pickl,[a] Christoph Bannwarth,[b*] Thorsten Bach[a*]

[a] School of Natural Sciences, Department Chemie, and Catalysis Research Center (CRC), Technische Universität München, Lichtenbergstrasse 4, 85747 Garching, Germany;

[b] Institut für Physikalische Chemie, RWTH Aachen University, 52074 Aachen, Germany.

Correspondence to: bannwarth@pc.rwth-aachen.de, thorsten.bach@ch.tum.de

Table of Contents

1. General Information 3
2. Analytical Methods 7
3. General Procedures 8
4. Synthesis of Benzophenone Catalyst 1b 11
5. Characterization of (+)-1b 16
6. Luminescence Measurements 19
7. Condition Optimization and Kinetic Studies 21
8. H/D-Cross-over Experiments 24
9. Cristallographic Data 28
10. Computational Studies 33
Correction on p. 39
11. Substrate Synthesis 48
12. Photochemical Deracemization Reactions 79
13. Downstream Synthetic Transformations 91
14. NMR Spectra 98
15. Chiral HPLC Traces 140
16. References 170

1. General Information

All reactions sensitive to air or moisture, were carried out in flame-dried glassware under positive pressure of argon using standard Schlenk techniques.
Commercially available chemicals were used without further purification, unless otherwise mentioned. For moisture sensitive reactions, dichloromethane (CH_2Cl_2) and tetrahydrofuran (THF) were purified using a MBSPS 800 *MBraun* solvent purification system. The following columns were used:

CH_2Cl_2: 2 × MB-KOL-A type (aluminium oxide)

THF: 2 × MB-KOL-M type 2 (3 Å molecular sieve)

Anhydrous *α,α,α*-trifluorotoluene ($PhCF_3$) was purchased from *Sigma Aldrich (Merck).* $PhCF_3$ used for photochemical deracemization reactions was additionally stored over 3 Å molecular sieves. Anhydrous acetonitrile (MeCN), benzene (PhH), chloroform ($CHCl_3$), dichloroethane (DCE), dimethylformamide (DMF), methanol (MeOH) and toluene ($PhCH_3$), were purchased from *Thermo Fisher Scientific* and stored over 3 Å molecular sieves.
Technical solvents for column chromatography [acetone (acetone), chloroform (CH_3Cl), ethyl acetate (EtOAc), methanol (MeOH), *n*-pentane (Pn)] were used after simple distillation.
Normal-phase flash column chromatography (FCC) was performed on silica 60 (*Merck*, 230-400 mesh) with the indicated eluent mixture.
Commercially available chemicals were purchased either from *Sigma Aldrich (Merck)*, *TCI Chemicals*, *ABCR* or *BLDpharm* and, were used without further purification, if not further mentioned.
Unless otherwise stated, photochemical reactions at $\lambda = 366$ nm were carried out in Duran phototubes (∅ 1 cm, 10, 20 or 200 mL) under argon atmosphere in a positive geometry setup with a cylindrical array of 16 fluorescent light tubes, UV-A, $\lambda_{max} = 366$ nm (Figures **S1** and **S2**). Prior to the start of a photoreaction, each reaction mixture was degassed by being sparged with argon under ultrasonication for 15 min.

Lehrstuhl OC 1 - TUM

200 nm | 250 nm | 300 nm | 350 nm | 400 nm | 450 nm | 500 nm | 550 nm | 600 nm | 650 nm

Datasheet FLT024 Philipps-BLB-365

Basic Information

Type	Fluorescent light tube
Description	Philipps TL 8W BLB
Manufacturer / Supplier	Philipps / Beleuchtungdirekt.de
Order number / Date of purch.	n/a / 02/2021
Internal lot / serial number	2021-02 / FLT024

Specification Manufacturer

Type / size	T5 tube, G5 socket
Mechanical specification	16 mm diameter, 288 mm length
Electrical specification	8 W
Wavelength (range, typ.)	350 - 400 nm
Spectral width (FWHM)	~ 16 nm
Datasheet	n/a

Characterization

Description of measurement	Measured with Ocean-optics USB4000 spectrometer using a calibrated setup (cosine corrector/fibre). The cosine corrector was placed at 20 mm distance from a single fluorescent tube at half height.	
Measured dominant wavelength / Int.	365 nm	168 µW/mm²nm
Measured spectral width (FWHM)	16 nm	
Integral Reference intensity / range	3059 µW/cm²	300-450 nm

Spectrum

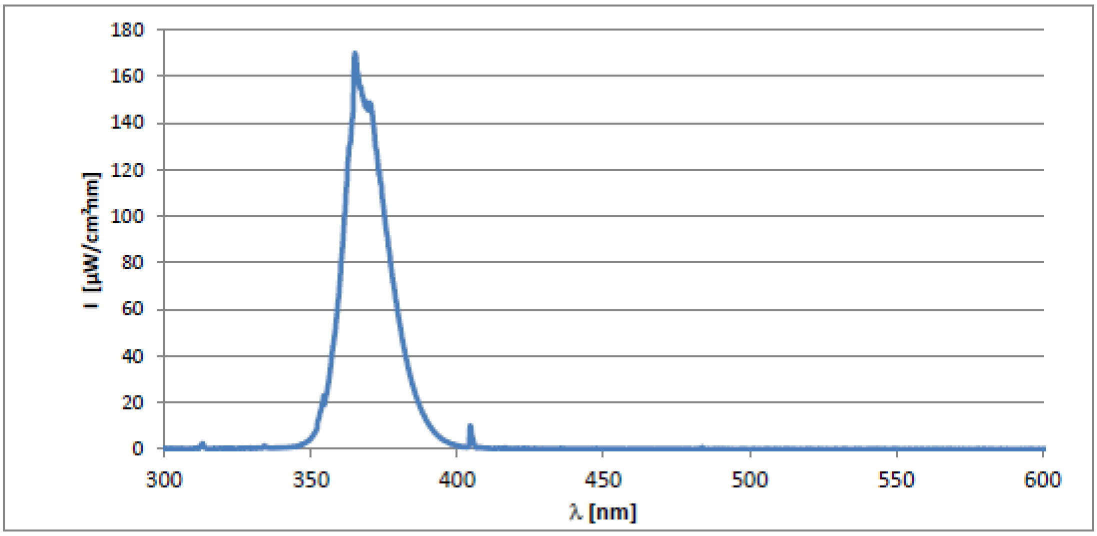

Figure S1: Emission spectrum of the 366 nm fluorescent light tube.

Lehrstuhl OC 1 - TUM

200 nm 250 nm 300 nm 350 nm 400 nm 450 nm 500 nm 550 nm 600 nm 650 nm

Datasheet FLT021 — LZC-UVA

Basic Information

Type	Fluorescent light tube
Description	Luzchem LZC-UVA
Manufacturer / Supplier	Hitachi / Luzchem
Order number / Date of purch.	LZC-UVA / 09/2015
Internal lot / serial number	2015-09 / FLT021

Specification Manufacturer

Type / size	T5 tube, G5 socket
Mechanical specification	16 mm diameter, 288 mm length
Electrical specification	8 W
Wavelength (range, typ.)	300 - 400 nm, 350m nm, UV-A
Spectral width (FWHM)	~ 40 nm
Datasheet	

Characterization

Description of measurement	Measured with Ocean-optics USB4000 spectrometer using a calibrated setup (cosine corrector/fibre). The cosine corrector was placed at 20 mm distance from a single fluorescent tube at half height.	
Measured dominant wavelength / Int.	350 nm	115 µW/mm²nm
Measured spectral width (FWHM)	40 nm	
Integral Reference intensity / range	5017 µW/cm²	300-425 nm

Spectrum

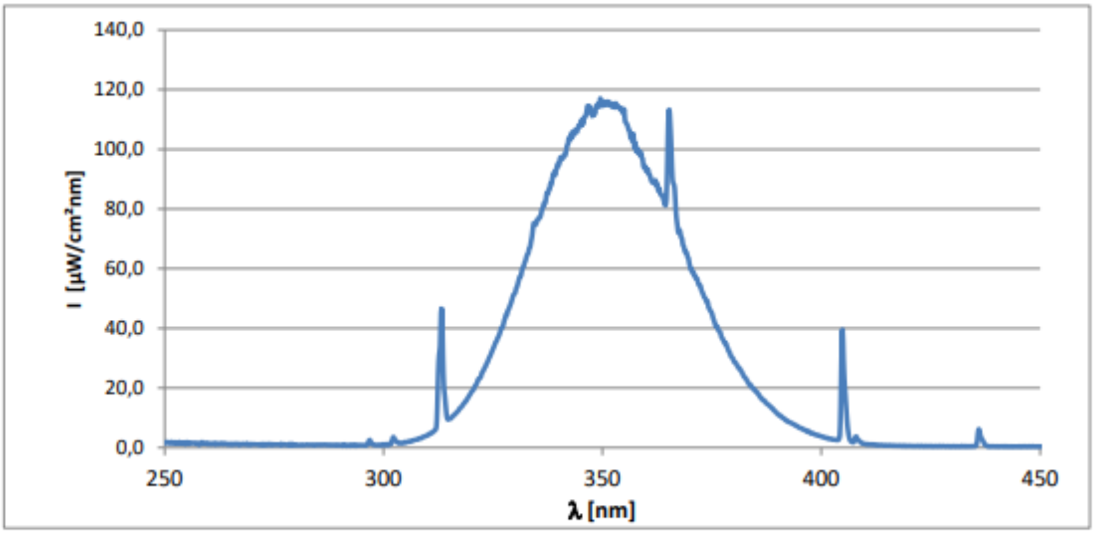

Figure S2: Emission spectrum of the 350 nm fluorescent light tube.

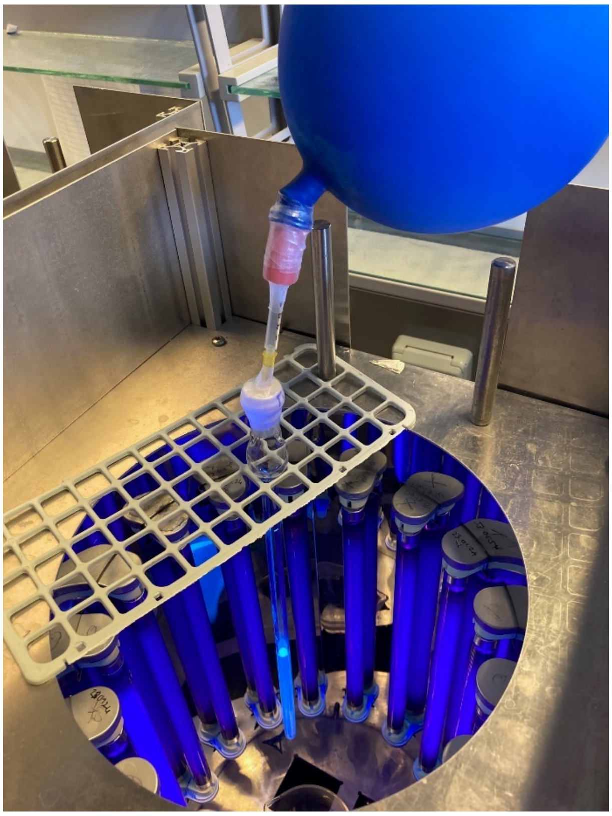

Figure S3: Typical setup for a photoreaction.

2. Analytical Methods

Thin layer chromatography (TLC) was performed on silica coated glass plates (silica gel 60 F_{254}) with detection by UV-light (λ = 254 nm) and potassium permanganate stain.

Infrared spectra (IR) were recorded on a *JASCO* IR-4100 or a *Perkin Elmer* Frontier IR-FTR spectrometer by ATR technique. The signal intensity is assigned using the following abbreviations: s (strong), m (medium), w (weak). The following abbreviations were used: aliph = aliphatic, arom = aromatic.

Melting points (M.p.) were determined using a Kofler ("Thermopan", Fs *Reichert*, Wien) apparatus.

Nuclear magnetic resonance (NMR) (^{1}H, ^{13}C and ^{19}F-NMR) spectra were recorded at room temperature (r.t.) on either a *Bruker* AVHD-400, AVHD-500, or a *Bruker* AV-II-500 equipped with cryo probe head. Chemical shifts of the NMR spectra are reported relative to $CHCl_3$ (^{1}H-NMR: δ = 7.26 ppm, ^{13}C-NMR: δ = 77.16 ppm) or DMSO (^{1}H-NMR: δ = 2.50 ppm, ^{13}C-NMR: δ = 39.52 ppm). The data are reported as follows: chemical shift (δ) [multiplicity, coupling constant J (Hz), relative integral, number of protons] where multiplicity is defined as: m = multiplet, s = singlet, d = doublet, t = triplet, q = quartet, non = nonet, br = broad. Apparent multiplets which occur because of coupling constant equality between magnetically non-equivalent protons are marked as virtual (*virt.*).

Mass spectrometry (MS) and **high-resolution mass spectrometry (HRMS)** were measured on a *Thermo Scientific* LTQ-FT Ultra (ESI) or a *Thermo Scientific* DFS-HRMS spectrometer (EI, 70 eV).

Specific Rotation was determined using an ADP440+ polarimeter (Fa *Bellingham+Stanley*) and is reported as follows: $[\alpha]_D^T$ (c in g per 100 mL solvent).

High Performance Liquid Chromatography (HPLC) was performed using a chiral stationary phase [ChiralPak AD-H (250 × 4.6 mm), Chiralpak IC (250 × 4.6 mm), Chiralpak AS-H (250 × 4.6 mm), AS-RH (150 × 4.6 mm), *Daicel Chemical Industries*] with UVD 340 Photodiode Array Detector, P580 Pump and an ASI-100 Automated Sample Injector at 20 °C. For normal-phase HPLC a *Daicel* ChiralPak AD-H, ChiralPak IC, and a ChiralPak AS-H was used as stationary phase, and a mixture of *n*-heptane/*i*-propanol was used as mobile phase. For reverse-phase HPLC a Daicel ChiralPak AS-RH, was used as stationary phase and a mixture of acetonitrile/water as mobile phase.

3. General Procedures

General Procedure A (GP A): Synthesis of benzyl-substituted racemic compounds

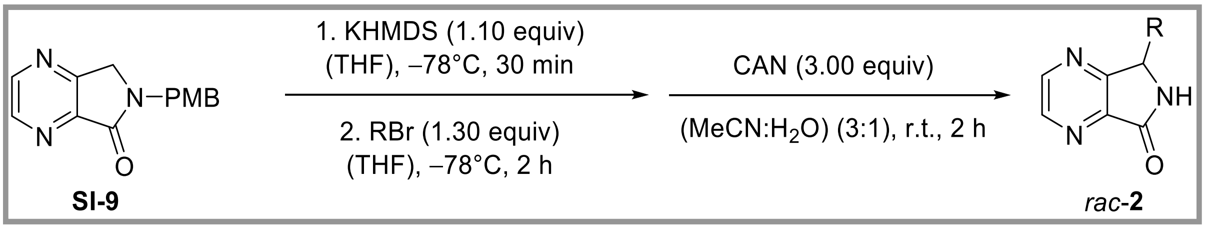

Following modified procedures,[1,2] a 1 M solution of KHMDS in THF (1.10 equiv.) was added dropwise to a suspension of 6-(4-methoxybenzyl) 6,7-dihydro-5*H*-pyrrolo[3,4-*b*]pyrazin-5-one (**SI-9**) (1.00 equiv.) in anhydrous THF (30 mL) at –78 °C, upon which the reaction mixture turned to a dark blue-purple solution. After 20 min, the respective bromide (1.30 equiv.) was added in one portion. The reaction was left to stir for 4 h at –78 °C. Afterwards, the solution was allowed to warm up to room temperature and quenched by the addition of sat. NH_4Cl (6 mL) and distilled water (20 mL). The aqueous layer was extracted with CH_2Cl_2 (3 × 25 mL), and the combined organic phases were dried over Na_2SO_4, filtered and concentrated under reduced pressure. The crude product was then dissolved in MeCN:H_2O (3:1) (29 mL), and diammonium cerium(IV) nitrate (3.00 equiv.) was added to the solution at room temperature. The yellow solution was stirred for 90 min at the same temperature. Subsequently, water (30 mL) was added, and the mixture was extracted with CH_2Cl_2 (3 × 50 mL). The combined organic layers were washed with brine (50 mL) and dried over Na_2SO_4. After filtration, the solvent was removed under reduced pressure and the residual crude product was subjected to FCC (SiO_2, EtOAc) to yield the desired racemic substrate *rac*-**2.**

General Procedure B (GP B): Synthesis of alkyl-substituted racemic substrates

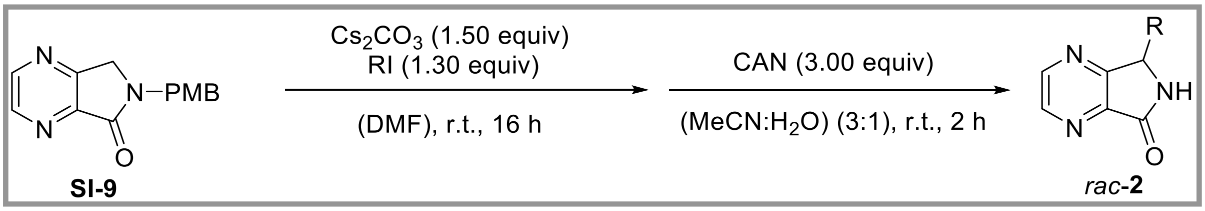

Following a modified procedure,[1,2] to a solution of 6-(4-methoxybenzyl) 6,7-dihydro-5*H*-pyrrolo[3,4-*b*]pyrazin-5-one (**SI-9**) (1.00 equiv.) in anhydrous DMF (5 mL) was added Cs_2CO_3 (1.50 equiv.) at room temperature. The resulting mixture was stirred for 5 min after which the respective iodide (1.30 equiv.) was added. The reaction was stirred for 16 h at the same temperature. Subsequently, the reaction was quenched by the addition of

sat. NH_4Cl (2 mL) and distilled water (10 mL). The aqueous layer was extracted with CH_2Cl_2 (3 × 25 mL), and the combined organic layers were washed with sat. LiCl solution (25 mL), brine (25 mL) and dried over Na_2SO_4. After filtration, the solvent was removed under reduced pressure. The crude product was then dissolved in MeCN:H_2O (3:1) (29 mL) and diammonium cerium(IV) nitrate (3.00 equiv.) was added to the solution at room temperature. The yellow solution was stirred for 90 min at the same temperature. Subsequently, water (30 mL) was added, and the mixture was extracted with CH_2Cl_2 (3 × 50 mL). The combined organic layers were washed with brine (50 mL) and dried over Na_2SO_4. After filtration, the solvent was removed under reduced pressure and the residual crude product was subjected to FCC (SiO_2, EtOAc) to yield the desired racemic substrate *rac*-**2.**

General Procedure C (GP C): Synthesis of alkoxy-substituted racemic substrates

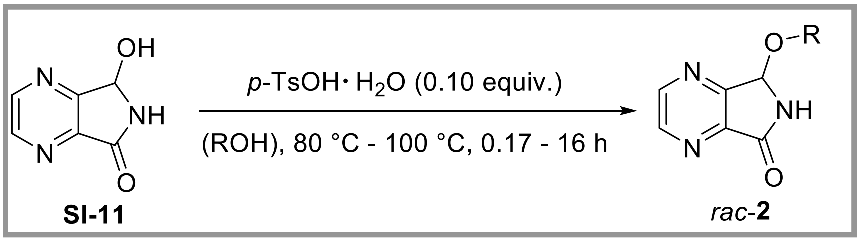

Following a modified procedure,[3] to a suspension of 7-hydroxy-6,7-dihydro-5*H*-pyrrolo[3,4-*b*]pyrazin-5-one (**SI-11**) (1.00 equiv.) in the respective alcohol (20.0 equiv.) was added *p*-TsOH (0.10 equiv.). The resulting mixture was then stirred between 80 °C and 100 °C until a clear solution prevailed. Afterwards, the mixture was allowed to cool to room temperature and was quenched by addition of a sat. bicarb solution (5 mL). The mixture was extracted with CH_2Cl_2 (3 × 20 mL), and the combined organic layers were washed with brine (20 mL) and dried over Na_2SO_4. After filtration, the solvent was removed under reduced pressure and the residual crude product was subjected to FCC (SiO_2, Pn/EtOAc) to yield the desired racemic substrate *rac*-**2.**

General Procedure D (GP D): Synthesis of silyl protected racemic substrates

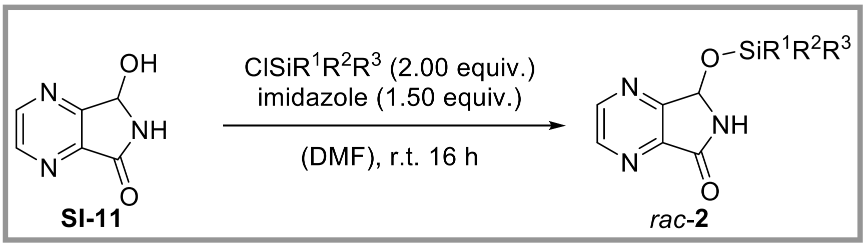

Following a modified procedure,[4] to a solution of 7-hydroxy-6,7-dihydro-5H-pyrrolo[3,4-b]pyrazin-5-one (**SI-11**) (1.00 equiv.) DMF was added imidazole (1.50 equiv.) and the respective chloro silane (2.00 equiv.) at room temperature. The resulting mixture was stirred for 16 h at the same temperature. Subsequently, the mixture was quenched by the addition of a sat. NH_4Cl solution (5 mL). The mixture was extracted with CH_2Cl_2 (3 × 20 mL), and the combined organic layers were washed with brine (20 mL) and dried over Na_2SO_4. After filtration, the solvent was removed under reduced pressure and the residual crude product was subjected to FCC (SiO_2, Pn/EtOAc) to yield the desired racemic substrate *rac*-**2**.

General Procedure E (GP E): Photochemical deracemization

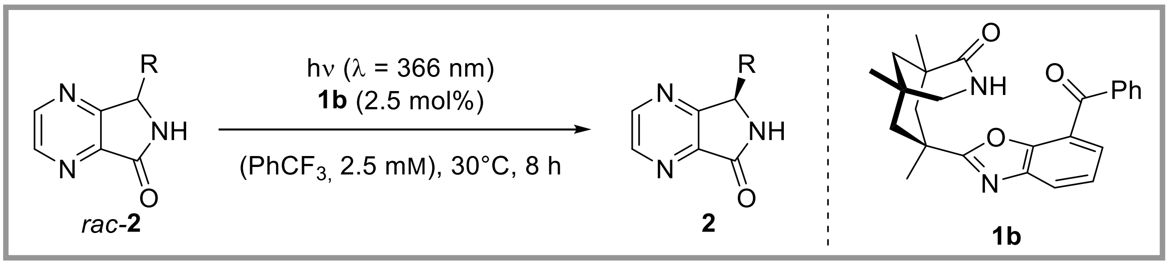

The corresponding racemic 4,7-diazaisoindolinone *rac*-**2** (25.0 µmol, c = 2.5 mM, 1.00 equiv.) and enantiomerically pure (+)-benzophenone **1b** (2.5 mol%) were dissolved in 10 mL pre-degassed α,α,α-trifluorotoluene ($PhCF_3$) in a flame dried phototube (Ø = 1 cm) and further degassed by bubbling argon through the solution for 15 min under ultrasonication. The solution was irradiated at λ = 366 nm for 8 h. The solvent was evaporated under reduced pressure and the crude product was subjected to flash column chromatography (SiO_2, EtOAc) to yield the enantiomerically enriched products **2**. The enantiomeric excess as well as the specific rotation were determined from the purified enantioenriched/enantiopure products, which were obtained as white solids.

4. Synthesis of Benzophenone Catalyst 1b

(2-Hydroxy-3-nitrophenyl)(phenyl)methanone (SI-1)

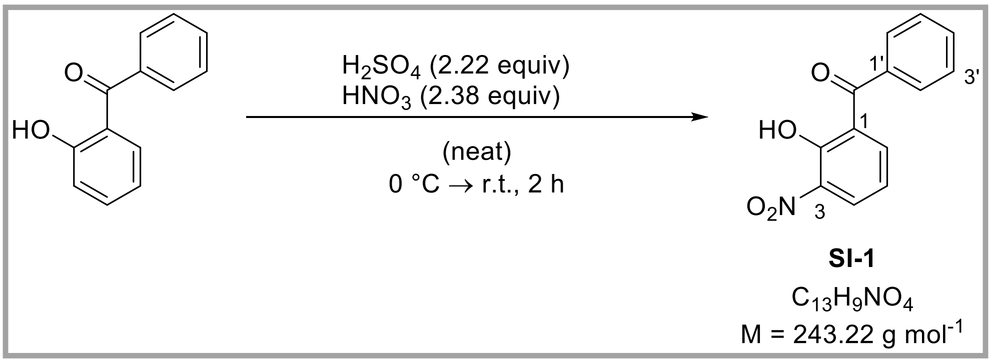

Following a literature known procedure,[5] a mixture of sulfuric acid (3.35 g, 34.2 mmol, 2.26 equiv.) and nitric acid (2.11 g, 33.5 mmol, 2.22 equiv.) was added dropwise to (2-hydroxyphenyl)(phenyl)methanone (3.00 g, 15.1 mmol, 1.00 equiv.) at 0 °C and was stirred for 1 h at the same temperature. The reaction mixture was allowed to warm to room temperature and stirred for an additional 2 h. Afterwards, the mixture was taken in ice water and the aqueous phase extracted with CH_2Cl_2 (3 × 40 mL). The combined organic layers were washed with sat. bicarb (50 mL), brine (40 mL) and dried over Na_2SO_4. The solvent was removed under reduced pressure and the residual crude product was subjected to FCC (SiO_2, Pn:EtOAc = 8:1 → 6:1) to yield the desired compound **SI-1** (851 mg, 3.50 mmol, 23%) as an orange-red oil which solidified upon standing.

TLC (Pn:EtOAc = 6:1): R_f = 0.43 [UV] [$KMnO_4$].

M.p.: 98 °C.

^{1}H-NMR (400 MHz, $CDCl_3$, 300 K): δ [ppm] = 11.68 (s, 1H, OH), 8.25 (dd, ^3J = 8.4 Hz, ^4J = 1.7 Hz, 1H, H4), 7.84 – 7.74 (m, 3H, H6, H2', H6'), 7.64 (tt, ^3J = 7.5 Hz, ^4J = 1.2 Hz, 1H, H4'), 7.54 – 7.47 (m, 2H, H3', H5'), 7.08 (*virt.* t, $^3J \approx {}^3J \approx$ 7.7 Hz, 1H, H5).

^{13}C-NMR (101 MHz, $CDCl_3$, 300 K): δ [ppm] = 196.3 (CO), 154.5 (C2), 138.0 (C6), 136.9 (C1'), 135.9 (C3), 133.7 (C4'), 129.7 (C2'), 129.1 (C4), 128.8 (C3'), 127.8 (C1), 119.3 (C-5).

HRMS (ESI) m/z: Calculated for $[M+H]^+$: 244.0604, found: 244.0616.

IR (film): $\tilde{\nu}$max/cm^{-1} = 3088 (m, CH_{arom}), 2924 (m, CH_{aliph}), 2854 (m, OCH_3), 1520 (s, $C_{arom}NO_2$), 1453 (m, C=C_{arom}), 1257 (C=O).

(3-Amino-2-hydroxyphenyl)(phenyl)methanone (SI-2)

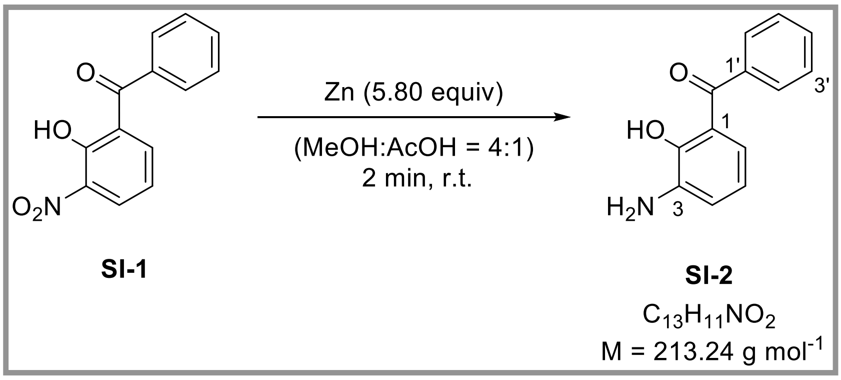

According to a modified procedure,[6] zinc dust (779 mg, 11.9 mmol, 5.80 equiv.) was added in one portion to a solution of benzophenone **SI-1** (500 mg, 2.05 mmol, 1.00 equiv.) in MeOH:AcOH (50 mL, 4:1) at room temperature. The suspension was stirred for 2 min and the excess zinc dust was filtered through cotton. Water (70 mL) was added to the filtrate and the aqueous layer was extracted with Et_2O (4 × 50 mL). The combined organic layers were washed with sat. bicarb (150 mL), brine (40 mL) and dried over Na_2SO_4. The solvent was removed under reduced pressure and the residual crude product was subjected to FCC (SiO_2, Pn:EtOAc = 8:1 → 4:1) to yield the desired compound **SI-2** (260 mg, 1.21 mmol, 59%) as a red oil.

TLC (Pn:EtOAc = 6:1): R_f = 0.52 [UV] [$KMnO_4$].

M.p.: 98 °C.

^{1}H-NMR (400 MHz, $CDCl_3$, 300 K): δ [ppm] = 12.21 (s, 1H, OH), 7.75-7.66 (m, 2H, H3'), 7.58 (tt, ^3J = 6.6 Hz, ^4J = 1.3 Hz, 1H, H4'), 7.52 – 7.45 (m, 2H, H2'), 6.99 (dd, ^3J = 7.9 Hz, ^4J = 1.5 Hz, 1H, H6), 6.93 (dd, ^3J = 7.9 Hz, ^4J = 1.5 Hz, 1H, H4), 6.71 (*virt.* t, $^3J \approx {}^3J \approx$ 7.9 Hz, 1H, H5), 3.98 (s, 2H, NH_2).

^{13}C-NMR (101 MHz, $CDCl_3$, 300 K): δ [ppm] = 202.2 (CO), 151.2 (C3), 138.4 (C1'), 136.6 (C1), 131.9 (C4'), 129.3 (C3'), 128.3 (C2'), 123.0 (C6), 120.2 (C4), 118.7 (C5), 118.6 (C2).

HRMS (ESI) m/z: calculated for $[M+H]^+$: 214.0863 found: 214.0872.

IR (film) ṽ max/cm^{-1} = 3461 (bs, NH), 3369 (bs, NH), 3055 (m, CH_{arom}), 1612 (s, C=O), 1597 (m, $C=C_{arom}$), 1573 (s, NH), 1452 (m, $C=C_{arom}$), 1224 (C-O).

2-Amino-6-benzoylphenyl (1*SR*,5*RS*,7*RS*)-1,5,7-trimethyl-2-oxo-3-azabicyclo[3.3.1]nonane-7-carboxylate (*rac*-SI-4)

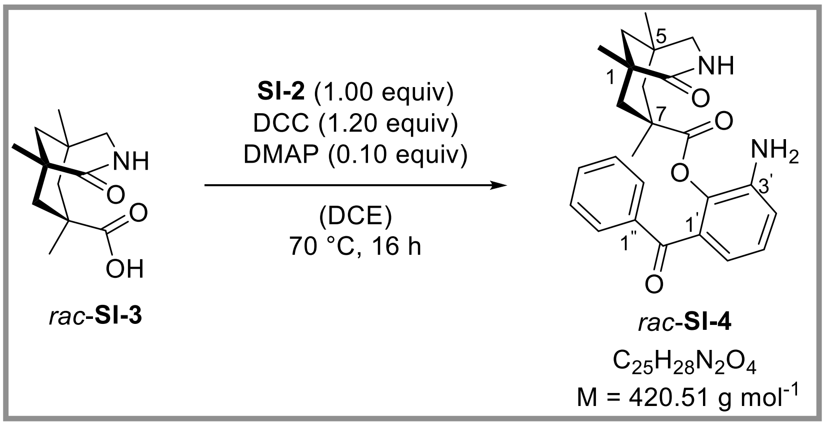

According to a modified procedure,[7] DCC (54.9 mg, 266 µmol, 1.20 equiv.) and DMAP (2.71 mg, 22.0 µmol, 0.10 equiv.) were added to a solution of *rac*-**SI3**[8] (50.0 mg, 221 µmol, 1.00 equiv.) and **SI-2** (47.3 mg, 221 µmol, 1.00 equiv.) in DCE (1.5 mL). The resulting solution was stirred for 16 h at 70 °C. Afterwards, the reaction was allowed to cool to room temperature, and the formed precipitate was removed by filtration. The solvent was removed under reduced pressure and the residual crude product was subjected to FCC (SiO_2, Pn:EtOAc = 3:1 → 1:1) to yield the title compound *rac*-**SI-4** (65.0 mg, 154 µmol, 70%) as a light yellow foam.

TLC (Pn:EtOAc = 1:1): R_f = 0.15 [UV] [$KMnO_4$].

M.p.: >260 °C.

^{1}H-NMR (400 MHz, $CDCl_3$, 300 K): δ [ppm] = 7.86 (dt, ^3J = 7.1 Hz, ^3J = 1.2 Hz, 2H, H2), 7.59 (*virt.* tt, $^3J \approx {}^3J \approx$ 7.3 Hz, 1.3 Hz, 1H, H4), 7.46 (*virt.* tt, $^3J \approx {}^3J \approx$ 7.5 Hz, $^4J \approx {}^4J \approx$ 1.3 Hz, 2H, H3), 7.16 (dd, ^3J = 8.0 Hz, ^4J = 1.9 Hz, 1H, H4'), 7.12 (*virt.* t, $^3J \approx {}^3J \approx$ 7.6, 1H, H5'), 6.77 (dd, ^3J = 7.2 Hz, ^4J = 1.9 Hz, 1H, H6'), 6.22 (s, 1H, CONH), 4.62 (s, 2H, NH_2), 3.21 (d, ^2J = 12.0 Hz, 1H, H_a4), 3.04 (d, ^2J = 12.0 Hz, 1H, H_b4), 2.75 (*virt.* dt, ^2J = 14.3 Hz, $^4J \approx {}^4J \approx$ 2.2 Hz, 1H, H_a8), 2.41 (*virt.* dt, ^2J = 14.3 Hz, $^4J \approx {}^4J \approx$ 2.2, 1H, H $_a6$), 1.76 (*virt.* dt, ^2J = 12.9 Hz, $^4J \approx {}^4J \approx$ 2.2 Hz, 1H, H_a9), 1.28 – 1.24 (m, 1H, H_b9), 1.20 (s, 3H, $C7CH_3$), 1.17 – 1.13 (m, 1H, $H8_b$), 1.00 (s, 3H, $C1CH_3$), 0.95 (s, 3H, $C5CH_3$), 1.00 – 0.94 (m, 1H, H_b6).

^{13}C-NMR (101 MHz, $CDCl_3$, 300 K): δ [ppm] = 194.4 (CO), 177.0 (CONH), 174.5 (COO), 136.8 (C3'), 136.7 (C1), 134.2 (C1'), 133.5 (C4), 130.8 (C2'), 130.7 (C2), 128.5 (C3), 126.1 (C5'), 121.2 (C4'), 120.6 (C6'), 52.9 (C4), 46.4 (C6), 45.4 (C8), 44.6 (C9), 43.0 (C1), 38.7 (C7), 31.4 (C1 CH_3), 30.4 (C5), 28.9 (C5 CH_3), 24.8 (C7CH_3).

HRMS (ESI) m/z: Calculated for $[M+H]^+$: 421.2122 found: 421.2137.

IR (film): $\tilde{v}$ max/cm^{-1} = 3335 (m, NH), 3226 (m, NH), 3062 (m, CH_{arom}), 2956 (m, CH_{aliph}), 1741 (s, C=O) 1650 (s, NHC=O), 1598 (m, $C=C_{arom}$), 1579 (s, NH), 1452 (m, $C=C_{arom}$).

(1*SR*,5*SR*,7*RS*)-7-(7-Benzoylbenzo[*d*]oxazol-2-yl)-1,5,7-trimethyl-3-azabicyclo[3.3.1]nonan-2-one (*rac*-1b)

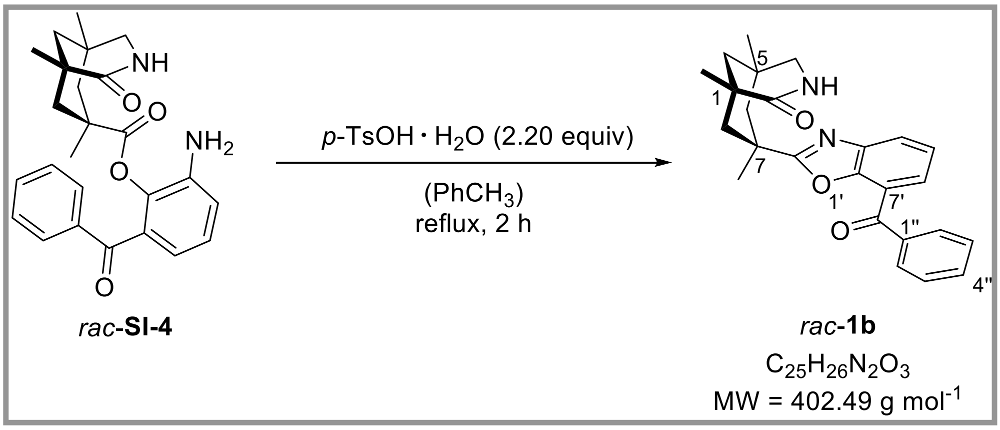

According to a modified procedure,[7] *p*-toluenesulfonic acid monohydrate (79.6 mg, 419 µmol, 2.20 equiv.) was heated to 100 °C in a schlenk tube under vacuum for 20 min. Afterwards, the tube was flushed with argon. Toluene (22 mL) and *rac*-**SI-4** (80.0 mg, 190 µmol, 1.00 equiv.) were added and the resulting suspension was refluxed for 2 h. Subsequently, the mixture was allowed to cool to room temperature and sat. bicarb solution (20 mL) was added. The organic layer was separated, and the aqueous layer was extracted with EtOAc (3 × 15 mL). The combined organic layers were washed with brine (15 mL) and dried over Na_2SO_4. After filtration, the solvent was removed under reduced pressure and the residual crude product was subjected to FCC (SiO_2, Pn:EtOAc = 2:1) to yield the title compound *rac*-**1b** (62.0 mg, 154 µmol, 81%) as a light-yellow foam.

TLC (Pn:EtOAc = 2:1): R_f = 0.31 [UV] [$KMnO_4$].

M.p.: >260 °C.

^{1}H-NMR (400 MHz, $CDCl_3$, 300 K): δ [ppm] = 7.89 (dd, ^{3}J = 7.5 Hz, ^{4}J = 1.2 Hz, 2H, H2), 7.85 (d, ^{3}J = 7.4 Hz, 1H, H4'), 7.69 (dd, ^{3}J = 7.7 Hz, ^{4}J = 1.0 Hz, 1H, H6'), 7.64 (tt, ^{3}J = 7.4 Hz, ^{4}J = 1.1 Hz, 1H, H4), 7.57 (*virt.* t, $^{3}J \approx {}^{3}J \approx$ 7.5 Hz, 2H, H3), 7.43 (*virt.* t, $^{3}J \approx {}^{3}J \approx$ 7.6 Hz, 1H, H5'), 4.90 (s, 1H, CONH), 3.36 (d, ^{2}J = 11.6 Hz, 1H, $H4_a$), 2.97 (d, ^{2}J = 12.0 Hz, 1H, $H4_b$), 2.79 (m, 2H, $H6_a$, $H8_b$), 1.76 (d, ^{3}J = 12.8 Hz, 1H, $H9_a$), 1.39 – 1.30 (m, 3H, $H6_b$, $H8_b$, $H9_b$), 1. (s, 3H, $C7CH_3$), 1.20 (s, 3H, $C1CH_3$), 1.05 (s, 3H, $C5CH_3$).

^{13}C-NMR (101 MHz, $CDCl_3$, 300 K): δ [ppm] = 193.0 (CO), 176.0 (CONH), 171.5 (C2'), 148.5 (C7a'), 142.3 (C3a'), 137.8 (C1), 132.9 (C4), 130.1 (C2), 128.4 (C3) 126.7 (C6'), 124.1 (C5'), 124.0 (C4'), 122.3 (C7'), 52.9 (C4), 46.4 (C6/C8), 46.3 (C6/C8), 44.7 (C9), 38.2 (C7), 37.6 (C1), 33.4 (C1*C*H_3), 30.6 (C5), 29.1 (C5*C*H_3), 24.8 (C7*C*H_3).

HRMS (ESI) m/z: Calculated for $[M+H]^+$: 403.2016 found: 403.2032.

IR (film): $\tilde{v}$max/cm^{-1} = 3376 (m, NH), 3063 (m, CH_{arom}), 2960 (m, CH_{aliph}), 2927 (m, CH), 2855 (m, OCH_3), 1651 (s, NHC=O), 1617 (m, $C{=}C_{arom}$), 1598 (m, $C{=}C_{arom}$), 1489 (m, $C{=}C_{arom}$), 1289 (C-O-C).

Chiral HPLC (AD-H, 250 × 4.6 mm, n-Hep:iso-PrOH = 50:50, 1mL/min, λ = 210 nm): 4.09 min (**1b**), 12.9 min (*ent*-**1b**).

The two enantiomers of *rac*-**1b** were separated on semi-preparative HPLC (*Daicel* Chiralpak AD, 250 × 20 mm, n-heptane/*i*-propanol 50:50).

Optical Rotation: $[\alpha]_D^{20}$: −53 (c = 1.0, CH_2Cl_2).

5. Characterization of (+)-1b

Determination of the absolute configuration of catalyst 1b

The absolute configuration of the enantiomers of compound **1b** was established using NMR titration experiments with 7-substituted 3-azabicyclo[3.3.1]nonan-2-one **SI-5** serving as reference, as previously described by our group.[9] This method leverages the heterochiral recognition properties of **SI-5**, which selectively forms dimers with chiral lactams of opposite configurations through hydrogen bonding interactions. Hydrogen bonding creates a distinct chemical environment for the lactam's NH proton, resulting in a characteristic shift in the ^{1}H NMR (matched) signal relative to non-bound case. Conversely, compounds with the same configuration as **SI-5** do not engage in hydrogen bonding, and thus no NMR shift is observed in the homochiral (mismatched) cases.

For this study, ^{1}H NMR spectra of **1b** and *ent*-**1b** (0.06 M in C_6D_6) were acquired on a *Bruker* AV-500 spectrometer at 298 K. Enantiomerically pure compound (–)-**SI-5** was incrementally added in 0.50 equivalent portions, with new ^{1}H NMR measurements recorded after each addition. The recorded spectra indicate that **1b** possesses the (+)-absolute configuration, while *ent*-**1b** exhibits the (–)-absolute configuration.

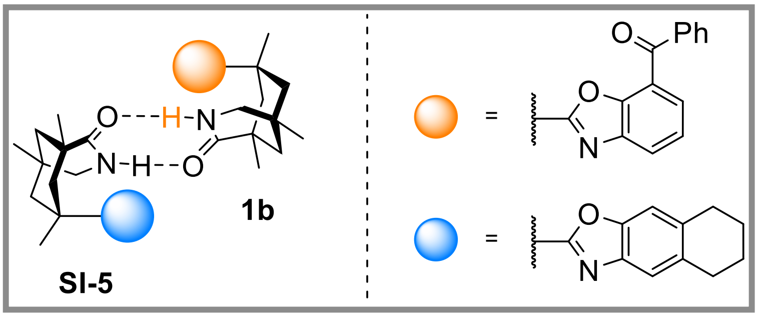

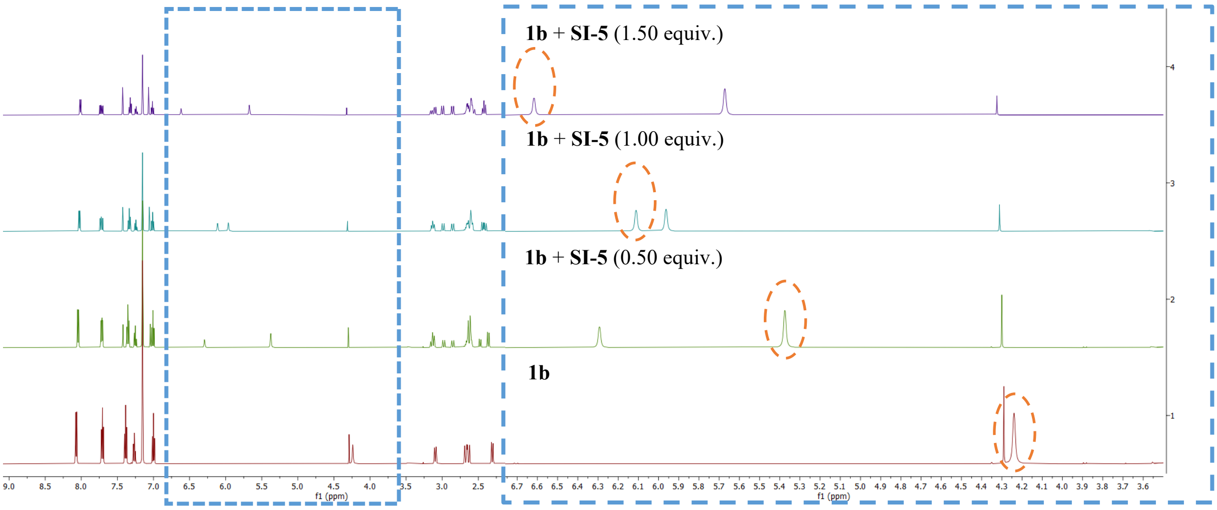

Figure S4: NMR-titration of **1b** with **SI-5** as reference compound in benzene-d6.

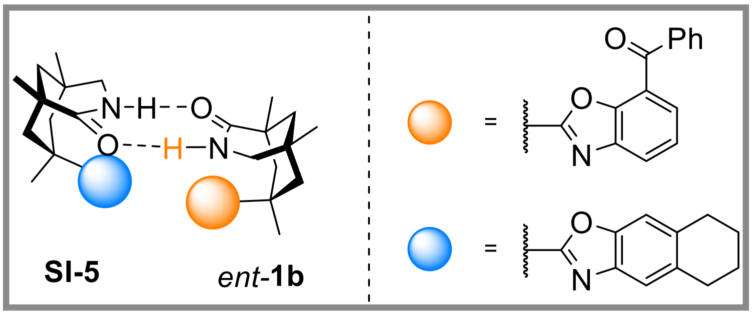

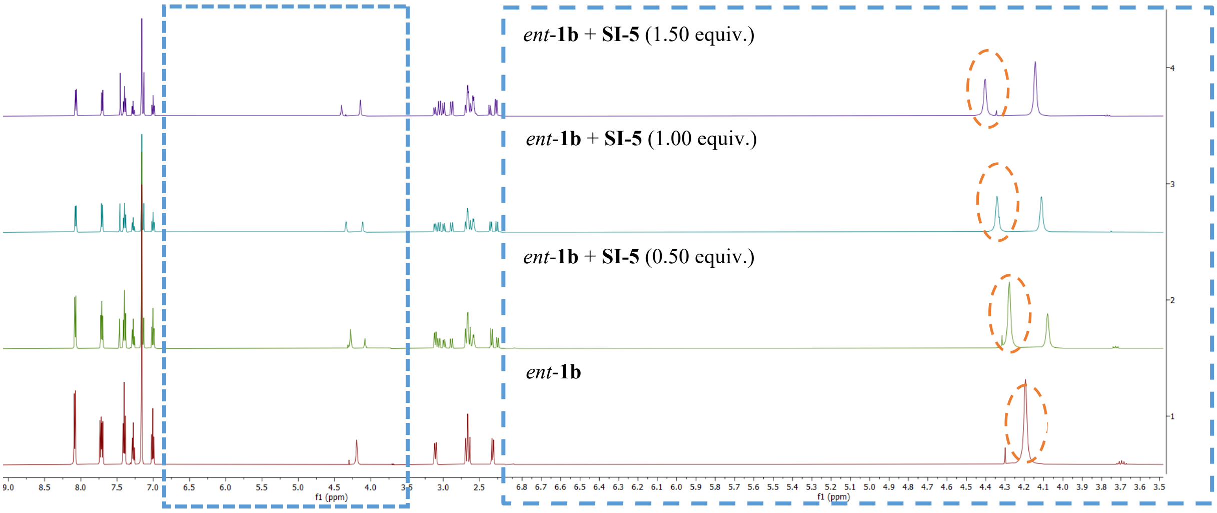

Figure S5: NMR-titration of *ent*-**1b** with **SI-5** as reference compound in benzene-d6.

UV/Vis Spectra of (+)-1b

The UV/Vis absorption spectrum of a 1 mM solution of (+)-**1b** in $PhCF_3$ was measured in *Hellma* precision cells made of quartz SUPRASIL® with a light pathway of 10 mm on a Perkin Elmer Lambda 35 UV/Vis spectrometer (Figure **S6**). The extinction coefficient ε at 360 nm for the forbidden first electronic nπ*-transition was calculated to be ε = 105 $M^{-1}cm^{-1}$.

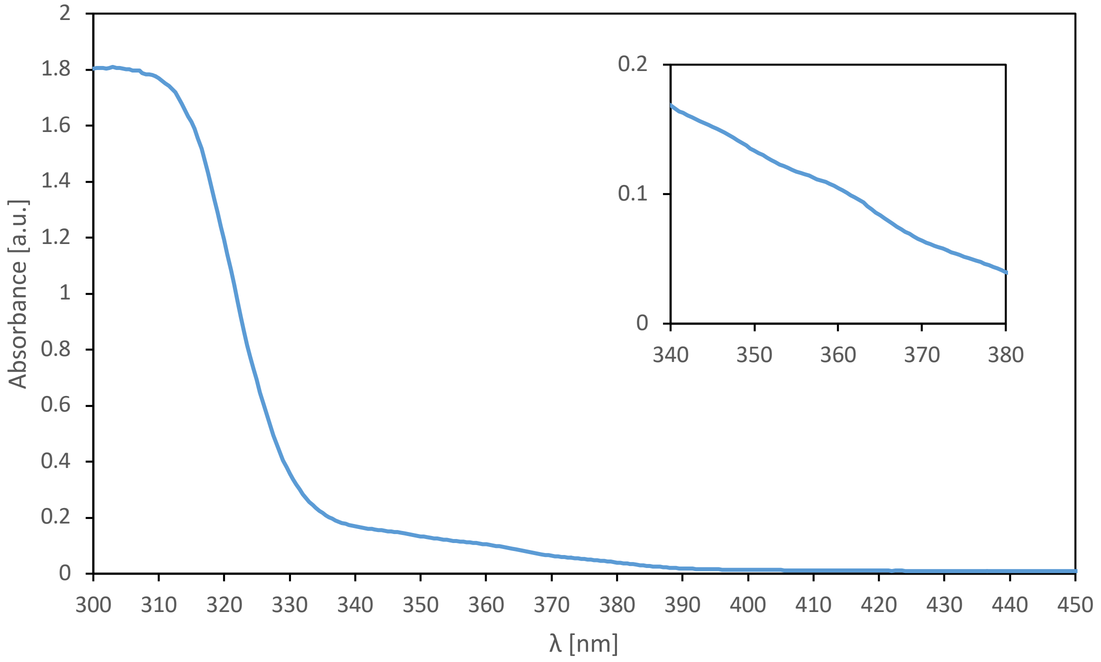

Figure S6: UV/Vis Spectra of (+)-**1b** in $PhCF_3$, (c = 1mM), 1 cm pathway.

6. Luminescence Measurements

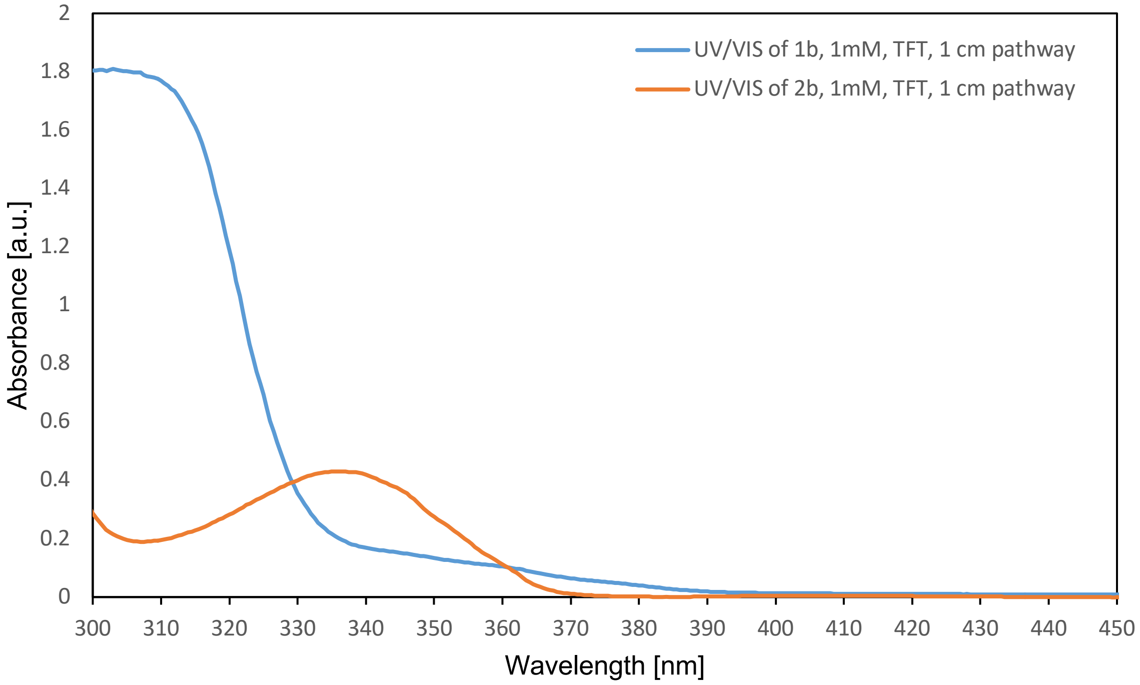

Figure S7: Recorded UV/Vis of **1b** and **2b** in $PhCF_3$ (c=1 mM). The extinction coefficient for substrate **2b** ε at 336 nm for the forbidden first electronic nπ*-transition was calculated to be ε = 403 $M^{-1}cm^{-1}$. The extinction coefficient ε for **2b** at 360 nm (maximum of **1b** for the forbidden first electronic nπ*-transition) was calculated to be ε = 106 $M^{-1}cm^{-1}$.

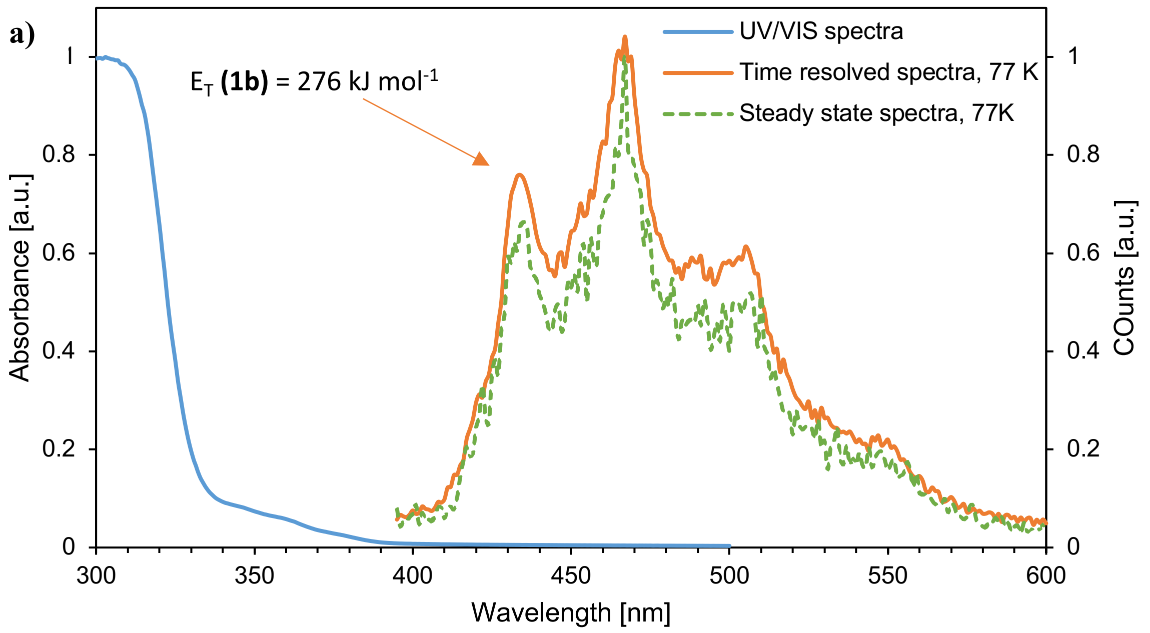

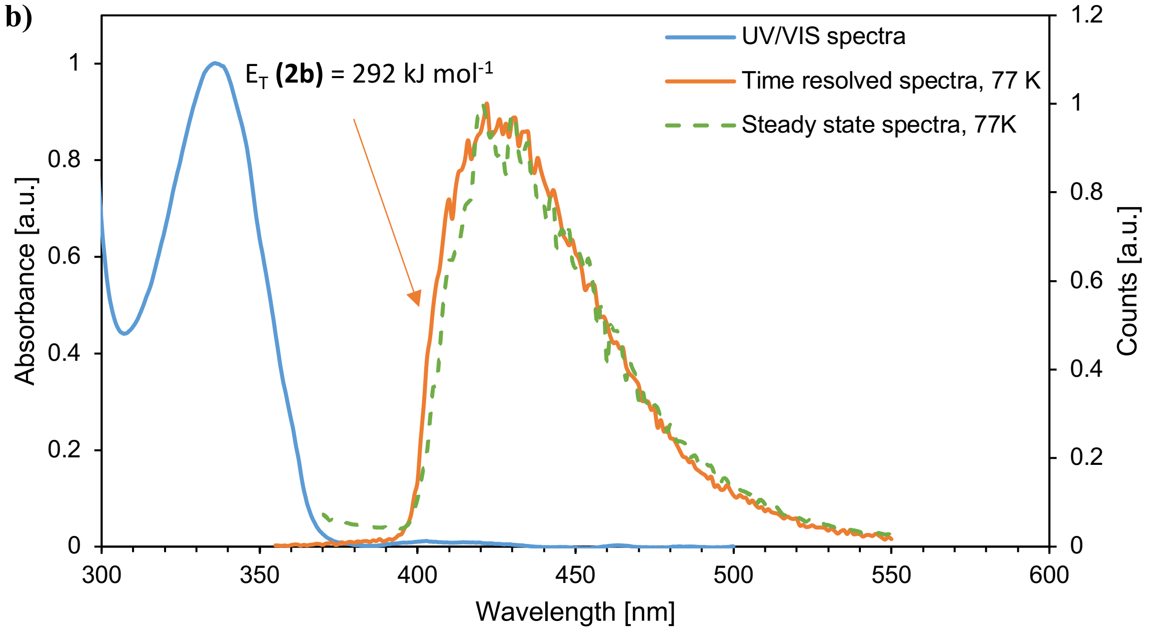

Figure S8: a) Recorded UV/Vis of **1b** in $PhCF_3$ (c=100 µM) normalized to A300 nm; steady state spectrum of **1b** in $PhCF_3$ (c=100 µM) at 77 K (dashed lines); time resolved spectrum of **1b** in $PhCF_3$ (c=100 µM) at 77K after 50 µs delay (solid line). The (0,0) transition results in a T_1 energy level of 276 kJ mol^{-1}. **b)** Recorded UV/Vis of **2b** in $PhCF_3$ (c=100 µM) normalized to A336 nm. Stady state spectrum of **2b** in $PhCF_3$ (c=100 µM) at 77 K (dashed lines); time resolved spectrum of **2b** in $PhCF_3$ (c=100 µM) at 77K after 50 µs delay (solid line). The point of inflection (409 nm) results in a T_1 energy level of 292 kJ mol^{-1}.

7. Condition Optimization and Kinetic Studies

The reaction conditions were optimized using substrate *rac*-**2a.** The reported yields and *ee* refer to the isolated product **2a**.

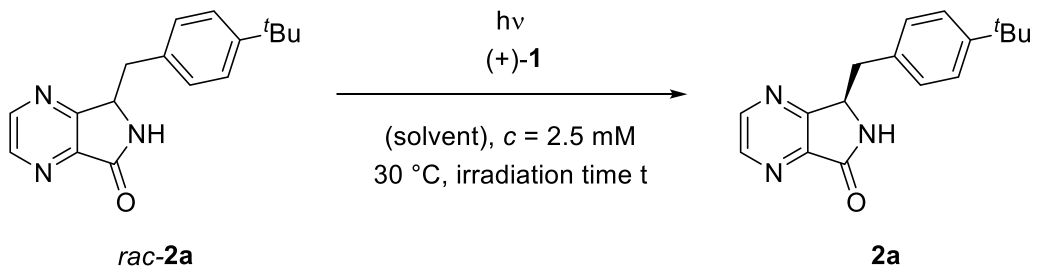

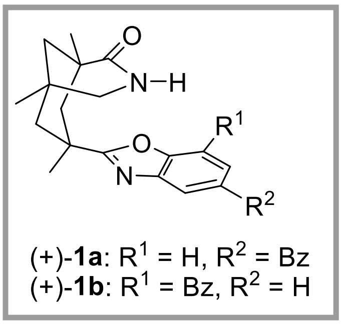

Table S1: Optimization of reaction conditions for the deracemization of *rac*-**2a**. All reactions were performed on a 25.0 µmol scale.

Entry	λ [nm]	Catalyst	[mol%]	solvent	t [h]	Yield [%]	*ee* [%]
1	366	**1a**	2.5	$PhCF_3$	8	92	2
2	366	**1b**	5	$PhCF_3$	13	72	97
3	366	**1b**	5	$PhCF_3$	4	79	98
4	366	**1b**	2.5	$PhCF_3$	4	91	80
5	366	**1b**	2.5	$PhCF_3$	6	89	93
6	366	**1b**	2.5	$PhCF_3$	8	90	98
7	366	**1b**	1	$PhCF_3$	8	91	86
8	366	**1b**	2.5	MeCN	8	80	10
9	366	**1b**	2.5	Acetone	8	74	18
10	366	**1b**	2.5	$PhCH_3$	8	58	70
11	350	**1b**	2.5	$PhCF_3$	8	66	93
12[a]	366	**1b**	2.5	$PhCF_3$	8	96	3
13	366	**1b**	2.5	$PhCF_3$: MeCN (9:1)	8	56	98

[a] The *N*-methylated substrate (*rac*-**2a**-Me) was used in this reaction.

To validate the optimal conditions identified in the initial screening, a kinetic study was performed. Samples of the reaction mixture (0.1 mL each) were taken at defined time intervals, using an argon-flushed syringe, followed by solvent evaporation. The resulting crude product was then dissolved in MeOH and analyzed using chiral HPLC. Care was taken to ensure that catalyst **1b** had a distinct retention time from the enantiomers of substrate **2a**, facilitating clear separation and accurate quantification of the enantiomeric ratio without interference from the catalyst. The enantiomeric excess shows a plateau after 8 h irradiation time which validates the conducted screening. The irradiation time for scope was not prolonged as we expect the yield to decrease and *ee* to erode with a prolonged irradiation time.

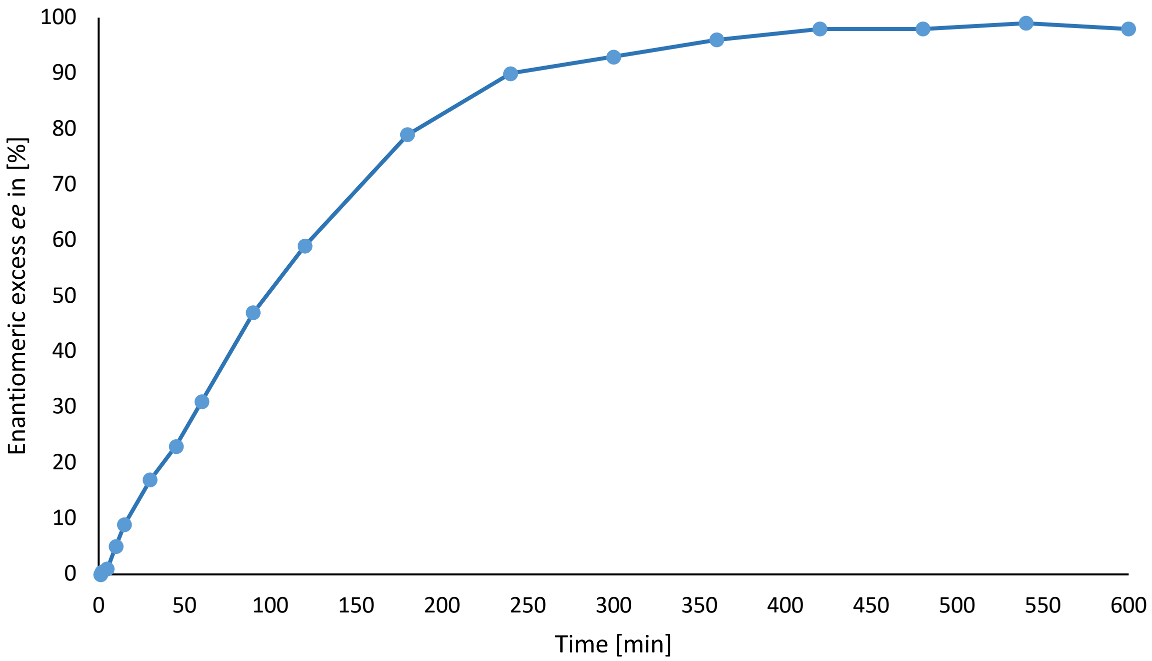

Figure S9: Kinetic profile for the deracemization of *rac*-**2a** using catalyst **2b** (2.5 mol%) in $PhCF_3$ (2.5 mM) irradiating at 366 nm.

Due to the poor solubility of certain investigated substrates in pure $PhCF_3$, a representative solvent screening was conducted using substrate *rac*-**SI-6**. We focused on more polar solvents to improve solubility. The clarity of the solution following degassing was recorded in the table as either “clear” or “turbid”. The results of the screening suggest that limited solubility is a critical factor contributing to the observed low *ee*. Additionally, a control experiment performed in the absence of irradiation (entry 12) revealed that loss of material is not responsible for the low yields achieved in the other entries.

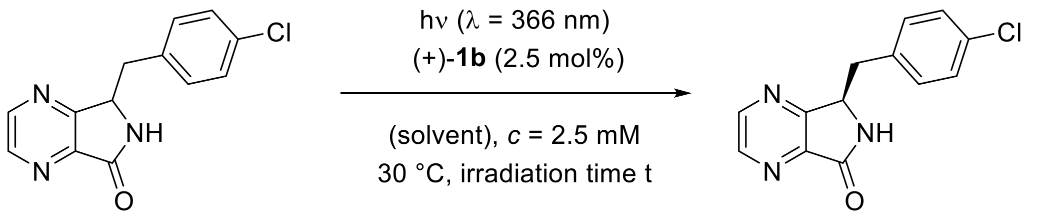

rac-**SI-6** **SI-6**

Table S2: Optimization of reaction conditions for the deracemization of *rac*-**SI-6**. All reactions were performed on a 25.0 μmol scale.

Entry	Solvent mixture	t [h]	Optical appearance	Yield [%]	*ee* [%]
1	$PhCF_3$	8	Turbid	76	20
2	$PhCF_3$:CH_2Cl_2 (7:3)	8	Clear	63	35
3	$PhCF_3$:CH_2Cl_2 (8:2)	8	Clear	58	55
4	$PhCF_3$:CH_2Cl_2 (9:1)	8	Turbid	70	7
5	$PhCF_3$:$CHCl_3$ (9:1)	8	Turbid	65	25
6	$PhCF_3$:acetone (8:2)	8	Clear	39	82
7	$PhCF_3$:acetone (9:1)	8	Clear	47	91
8	$PhCF_3$:acetone (9.5:0.5)	8	Turbid	75	12
9	$PhCF_3$:MeCN (9:1)	8	Clear	54	96
10	$PhCF_3$:MeCN (9.5:0.5)	8	Turbid	66	38
11	$PhCF_3$:MeCN (9:1)	4	Clear	73	38
12[a]	$PhCF_3$:MeCN (9:1)	8	Clear	96	0

[a] The mixture was left in the dark for the given reaction time.

Since a mixture of $PhCF_3$:MeCN (9:1) resulted in the best *ee*, those conditions were applied to other substrates which were insoluble in pure $PhCF_3$. However, here we received mixed results. While the 3-benzyloxy substitution resulted in complete decomposition, the phenyl-substituted substrate **SI-7** was obtained with good *ee,* but only moderate yield.

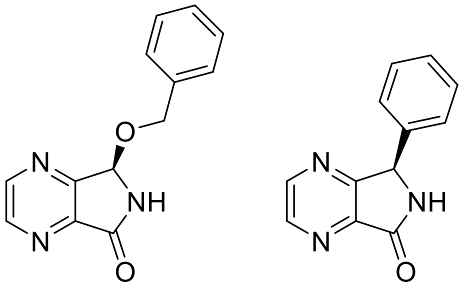

decomposition **SI-7** 63%, 94% *ee*

8. H/D-Cross-over Experiments

Racemic experiment

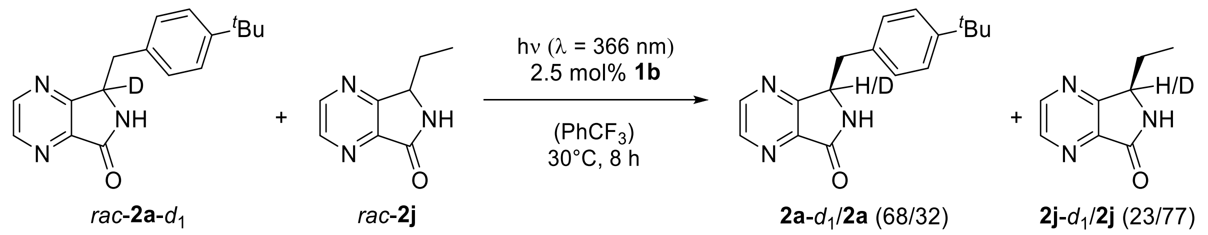

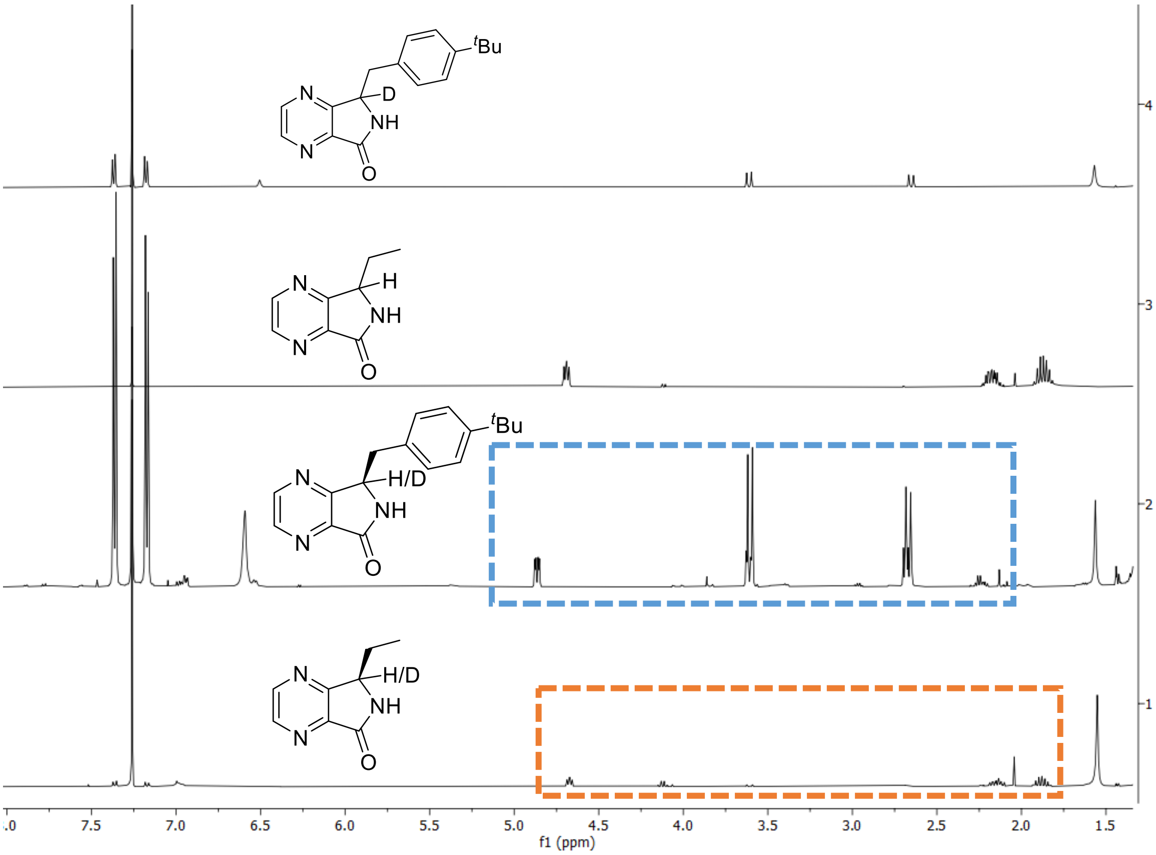

Figure S10: Deuterium cross-over experiment between *rac*-**2a**-d_1 and *rac*-**1c** under irradiation conditions with catalyst **1b** (2.5 mol%). The level of deuterium scrambling was determined after column chromatography by NMR. The NMR was recorded in $CDCl_3$ on AVHD500 *Brucker* NMR.

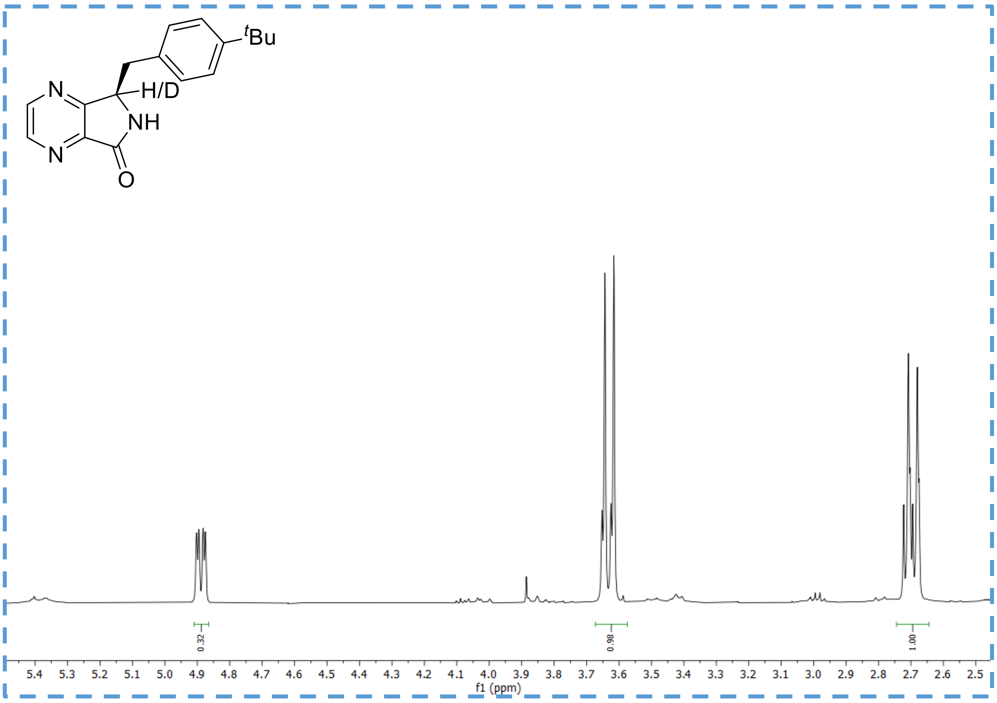

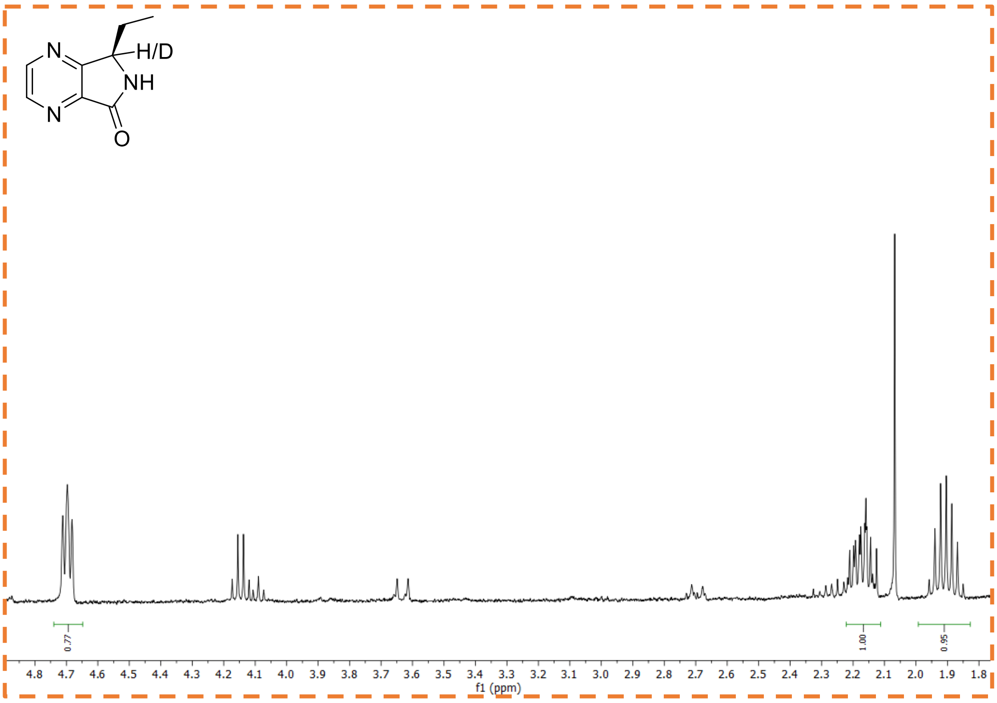

According to GP E, a degassed solution of *rac*-**2a**-d1 (3.53 mg, 12.5 µmol, 1.00 equiv.), *rac*-**2j** (2.04 mg, 12.5 µmol, 1.00 equiv.) and enantiomerically pure (+)-benzophenone **1b** (252 µg, 625 nmol, 2.5 mol% with respect to the amount of stereogenic centers) in α,α,α-trifluorotoluene (10 mL) was irradiated at λ = 366 nm for 8 h. After irradiation, the solvent was evaporated and the residue was purified by FCC (SiO_2, EtOAc) to obtain **2a**-d1 (2.94 mg, 10.5 µmol, 84%) and *rac*-**2j** (1.62 mg, 9.92 µmol, 79%).

Experiment with (*R*)-enantiomers

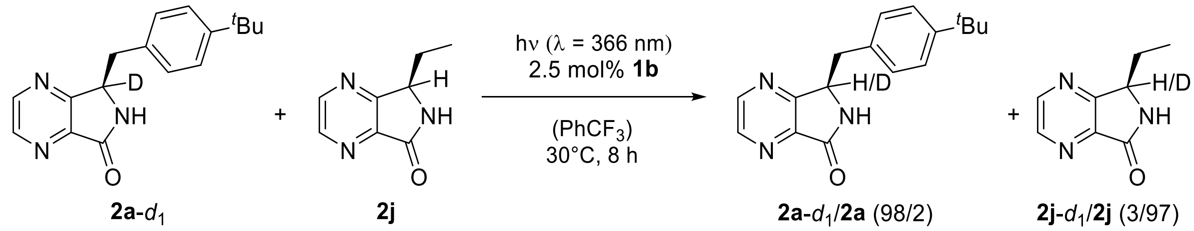

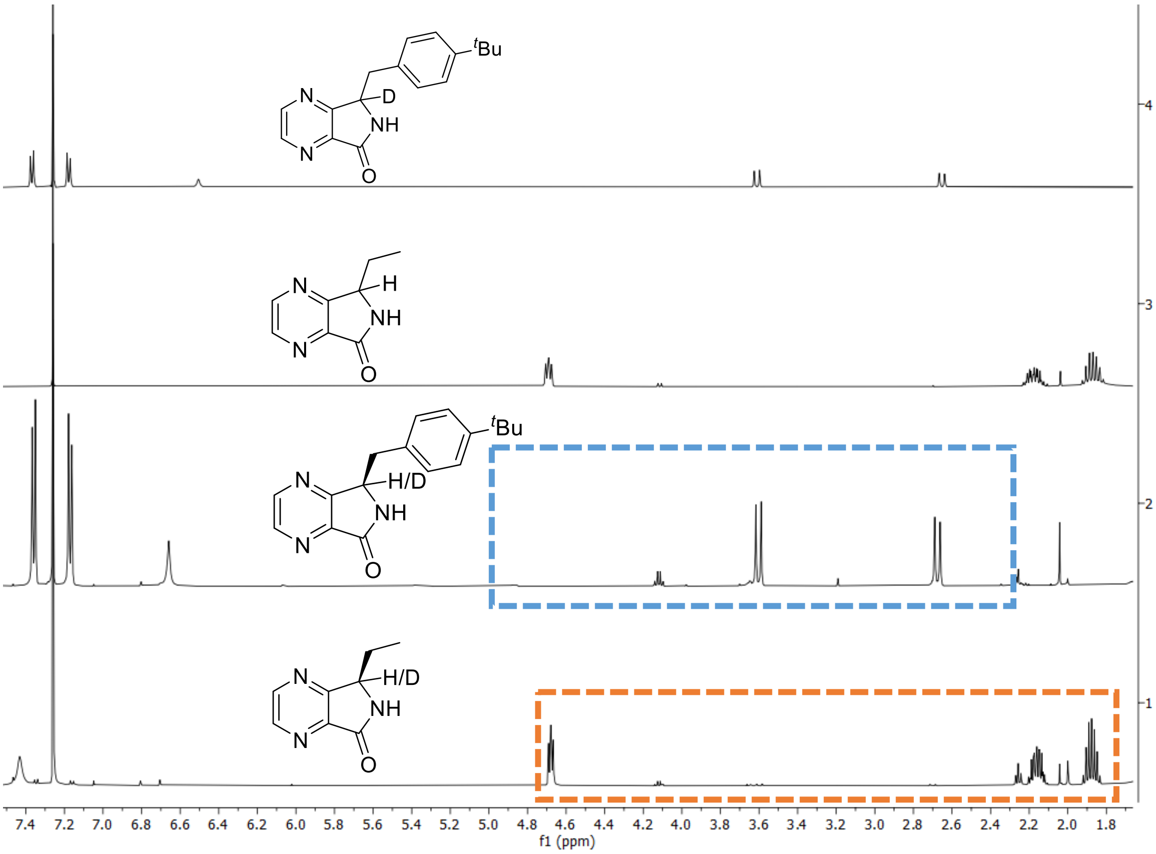

Figure S11: Deuterium cross-over experiment between **2a**-d_1 and **1c** under irradiation conditions with catalyst **1b** (2.5 mol%). The level of deuterium scrambling was determined after column chromatography by NMR. The NMR was recorded in $CDCl_3$ on AVHD500 Brucker NMR.

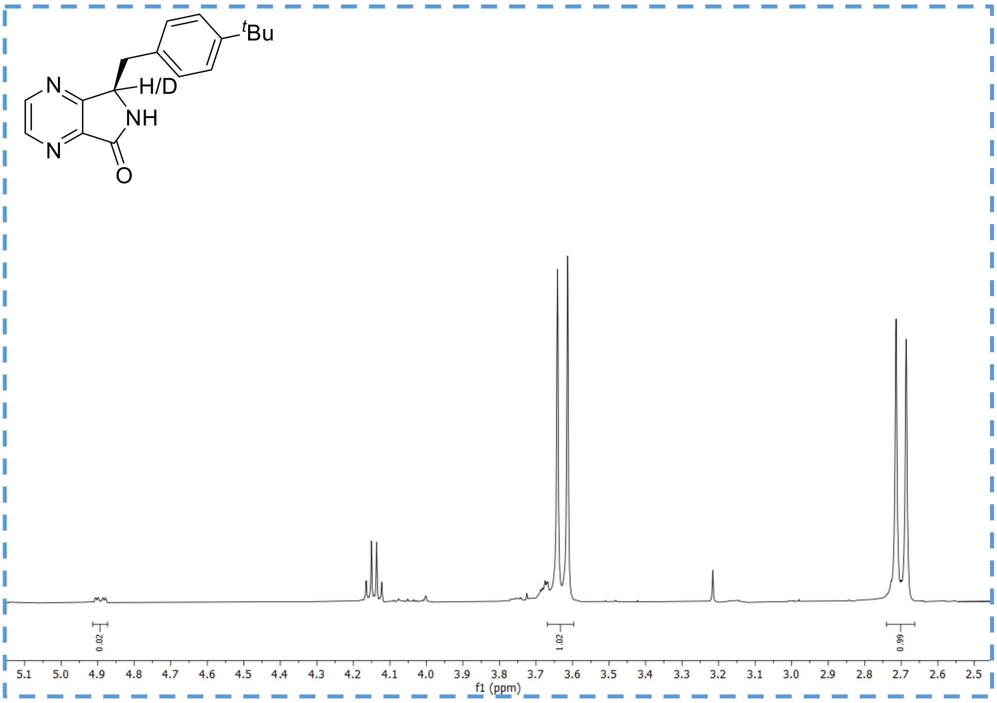

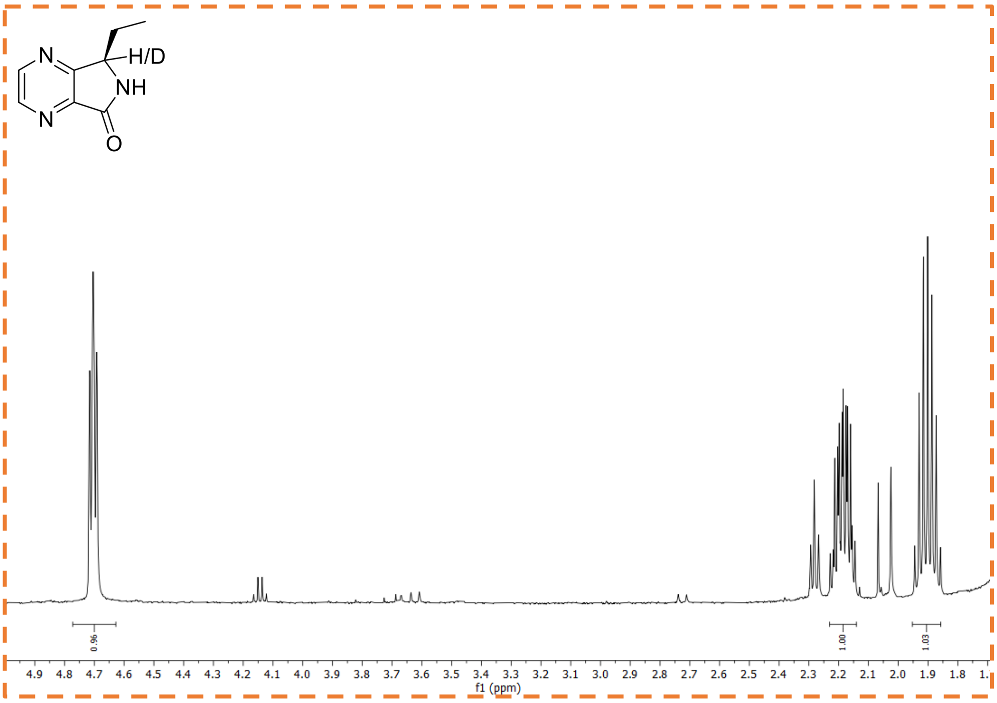

According to GP E, a degassed solution of *rac*-**2a**-d_1 (3.53 mg, 12.5 µmol, 1.00 equiv.), *rac*-**2j** (2.04 mg, 12.5 µmol, 1.00 equiv.) and enantiomerically pure (+)-benzophenone **1b** (252 µg, 625 nmol, 2.5 mol% with respect to the amount of stereogenic centers) in α,α,α-trifluorotoluene (10 mL) was irradiated at λ = 366 nm for 8 h. After irradiation, the solvent was evaporated and the residue was purified by FCC (SiO_2, EtOAc) to obtain **2a**-d_1 (3.41 mg, 12.1 µmol, 97%) and *rac*-**2j** (1.87 mg, 11.5 µmol, 92%).

9. Cristallographic Data

SC-XRD structure report for compound 2d

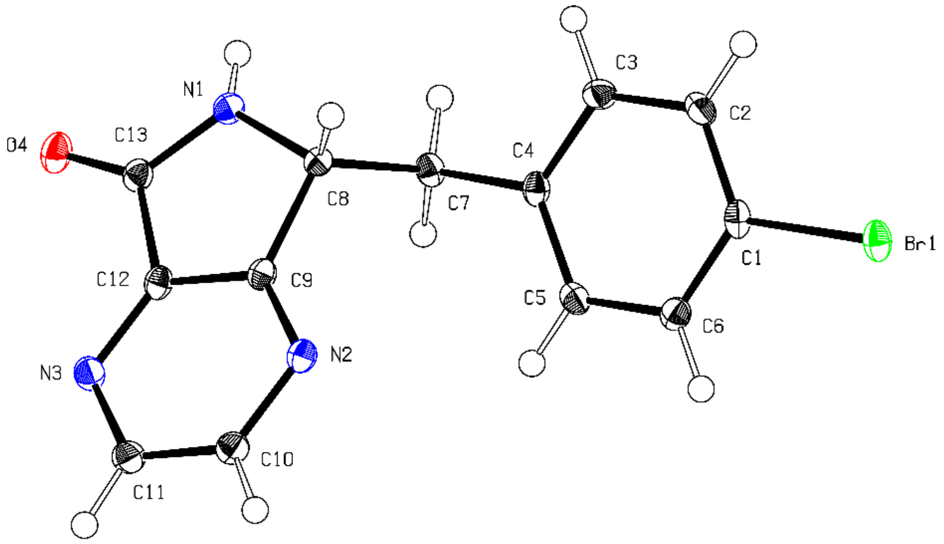

A colorless, rod-shaped crystal of $C_{13}H_{10}BrN_3O$ coated with perfluorinated ether and fixed on top of a *Kapton* micro sampler was used for X-ray crystallographic analysis. The X-ray intensity data were collected at 100(2) K on a *Bruker* D8 VENTURE three-angle diffractometer with a TXS rotating anode with MoK_α radiation (λ=0.71073 Å) using APEX4.[10] The diffractometer was equipped with a *Helios* optic monochromator, a *Bruker* PHOTON III detector, and an *Oxford* Cryostream low temperature device.

A matrix scan was used to determine the initial lattice parameters. All data were integrated with the *Bruker* SAINT V8.40B software package using a narrow-frame algorithm and the reflections were corrected for Lorentz and polarisation effects, scan speed, and background.[11] The integration of the data using a monoclinic unit cell yielded a total of 67117 reflections within a 2θ range [°] of 5.27 to 56.69 (0.75 Å), of which 2929 were independent. Data were

corrected for absorption effects including odd and even ordered spherical harmonics by the multi-scan method (SADABS 2016/2).[12] Space group assignment was based upon systematic absences, E statistics, and successful refinement of the structure.

The structure was solved by direct methods using SHELXT and refined by full-matrix least-squares methods against F^2 by minimizing $\Sigma w(F_o^2 - F_c^2)^2$ using SHELXL in conjunction with SHELXLE.[13-15] All non-hydrogen atoms were refined with anisotropic displacement parameters. Hydrogen atoms were refined isotropically on calculated positions using a riding model with their U_{iso} values constrained to 1.5 times the U_{eq} of their pivot atoms for terminal sp^3 carbon atoms and a C–H distance of 0.98 Å. Non-methyl hydrogen atoms were refined using a riding model with methylene, aromatic, and other C–H distances of 0.99 Å, 0.95 Å, and 1.00 Å, respectively, and U_{iso} values constrained to 1.2 times the U_{eq} of their pivot atoms.

Neutral atom scattering factors for all atoms and anomalous dispersion corrections for the non-hydrogen atoms were taken from International Tables for Crystallography.[16] Supplementary crystallographic data reported in this paper have been deposited with the Cambridge Crystallographic Data Centre (CCDC 2401033) and can be obtained free of charge from The Cambridge Crystallographic Data Centre via www.ccdc.cam.ac.uk/structures.[17] This report and the CIF file were generated using FinalCif.[18]

Table S3 Crystal data and structure refinement for compound **2d**.

CCDC number	2401033
Empirical formula	$C_{13}H_{10}BrN_3O$
Formula weight	304.15
Temperature [K]	100(2)
Crystal system	Monoclinic
Space group (number)	$P2_1$ (4)
a [Å]	4.8857(3)
b [Å]	10.7814(6)
c [Å]	11.1467(6)
α [°]	90
β [°]	95.210(2)
γ [°]	90
Volume [Å^3]	584.72(6)
Z	2
ρ_{calc} [gcm^{-3}]	1.727
μ [mm^{-1}]	3.505
F(000)	304
Crystal size [mm^3]	0.027×0.064×0.140
Crystal colour	Colourless
Crystal shape	Rod
Radiation	MoK_α (λ=0.71073 Å)
2θ range [°]	5.27 to 56.69 (0.75 Å)

Index ranges	$-6 \leq h \leq 6$<br>$-14 \leq k \leq 14$<br>$-14 \leq l \leq 14$
Reflections collected	67117
Independent reflections	2929<br>R_{int} = 0.0469<br>R_{sigma} = 0.0207
Completeness to θ = 25.242°	99.8 %
Data / Restraints / Parameters	2929 / 1 / 166
Goodness-of-fit on F^2	1.068
Final R indexes [$I \geq 2\sigma(I)$]	R_1 = 0.0158<br>wR_2 = 0.0366
Final R indexes [all data]	R_1 = 0.0164<br>wR_2 = 0.0368
Largest peak/hole [eÅ^{-3}]	0.24/−0.20
Flack y parameter	0.019(3)

Table S4 Bond lengths and angles for compound **2d**.

**Atom–Atom**	**Length [Å]**
Br1–C1	1.901(2)
C2–C1	1.384(3)
C2–C3	1.390(3)
C2–H2	0.9500
C3–C4	1.397(3)
C3–H3	0.9500
C4–C5	1.392(3)
C4–C7	1.509(3)
C5–C6	1.392(3)
C5–H5	0.9500
C1–C6	1.381(3)
C6–H6	0.9500
C7–C8	1.541(3)
C7–H7A	0.9900
C7–H7B	0.9900
C8–N1	1.470(3)
C8–C9	1.506(3)
C8–H8	1.0000
C9–N2	1.332(3)
C9–C12	1.383(3)
C10–N2	1.347(3)
C10–C11	1.400(3)
C10–H10	0.9500
C11–N3	1.338(3)
C11–H11	0.9500
C12–N3	1.334(3)
C12–C13	1.490(3)
C13–O4	1.227(3)
C13–N1	1.347(3)
N1–H1	0.75(3)
**Atom–Atom–Atom**	**Angle [°]**
C1–C2–C3	118.9(3)
C1–C2–H2	120.6

C3–C2–H2	120.6
C2–C3–C4	121.0(2)
C2–C3–H3	119.5
C4–C3–H3	119.5
C5–C4–C3	118.6(2)
C5–C4–C7	121.85(19)
C3–C4–C7	119.52(19)
C4–C5–C6	120.8(2)
C4–C5–H5	119.6
C6–C5–H5	119.6
C6–C1–C2	121.4(2)
C6–C1–Br1	119.00(16)
C2–C1–Br1	119.59(19)
C1–C6–C5	119.2(2)
C1–C6–H6	120.4
C5–C6–H6	120.4
C4–C7–C8	114.47(18)
C4–C7–H7A	108.6
C8–C7–H7A	108.6
C4–C7–H7B	108.6
C8–C7–H7B	108.6
H7A–C7–H7B	107.6
N1–C8–C9	100.41(17)
N1–C8–C7	109.47(18)
C9–C8–C7	115.51(18)
N1–C8–H8	110.3
C9–C8–H8	110.3
C7–C8–H8	110.3
N2–C9–C12	123.1(2)
N2–C9–C8	127.3(2)
C12–C9–C8	109.68(19)
N2–C10–C11	123.3(2)
N2–C10–H10	118.3
C11–C10–H10	118.3
N3–C11–C10	122.3(2)
N3–C11–H11	118.8
C10–C11–H11	118.8
N3–C12–C9	124.0(2)
N3–C12–C13	126.6(2)
C9–C12–C13	109.4(2)
O4–C13–N1	127.6(2)
O4–C13–C12	128.0(2)
N1–C13–C12	104.41(19)
C13–N1–C8	115.78(19)
C13–N1–H1	120(3)
C8–N1–H1	123(3)
C9–N2–C10	113.5(2)
C12–N3–C11	113.8(2)

Table **S5:** Torsion angles for compound **2d**.

**Atom–Atom–Atom–Atom**	**Torsion Angle [°]**
C1–C2–C3–C4	−0.1(3)
C2–C3–C4–C5	1.3(3)
C2–C3–C4–C7	−177.3(2)
C3–C4–C5–C6	−1.2(3)
C7–C4–C5–C6	177.38(19)
C3–C2–C1–C6	−1.4(3)
C3–C2–C1–Br1	178.28(17)
C2–C1–C6–C5	1.6(3)
Br1–C1–C6–C5	−178.14(15)
C4–C5–C6–C1	−0.2(3)
C5–C4–C7–C8	89.3(2)
C3–C4–C7–C8	−92.1(2)
C4–C7–C8–N1	162.45(18)
C4–C7–C8–C9	−85.2(2)
N1–C8–C9–N2	−175.65(19)
C7–C8–C9–N2	66.8(3)
N1–C8–C9–C12	4.3(2)
C7–C8–C9–C12	−113.3(2)
N2–C10–C11–N3	−0.9(3)
N2–C9–C12–N3	−1.1(3)
C8–C9–C12–N3	178.91(18)
N2–C9–C12–C13	177.92(18)
C8–C9–C12–C13	−2.0(2)
N3–C12–C13–O4	−2.6(3)
C9–C12–C13–O4	178.4(2)
N3–C12–C13–N1	177.63(19)
C9–C12–C13–N1	−1.4(2)
O4–C13–N1–C8	−175.2(2)
C12–C13–N1–C8	4.6(2)
C9–C8–N1–C13	−5.6(2)
C7–C8–N1–C13	116.4(2)
C12–C9–N2–C10	0.8(3)
C8–C9–N2–C10	−179.23(19)
C11–C10–N2–C9	0.1(3)
C9–C12–N3–C11	0.3(3)
C13–C12–N3–C11	−178.57(19)
C10–C11–N3–C12	0.6(3)

10. Computational Studies

We consider the enantiomeric 4,7-diaza derivative with benzylic substituent, *ent*-**2b** and the chiral benzophenone catalyst **1b** for our computational study (Figure **S12**).

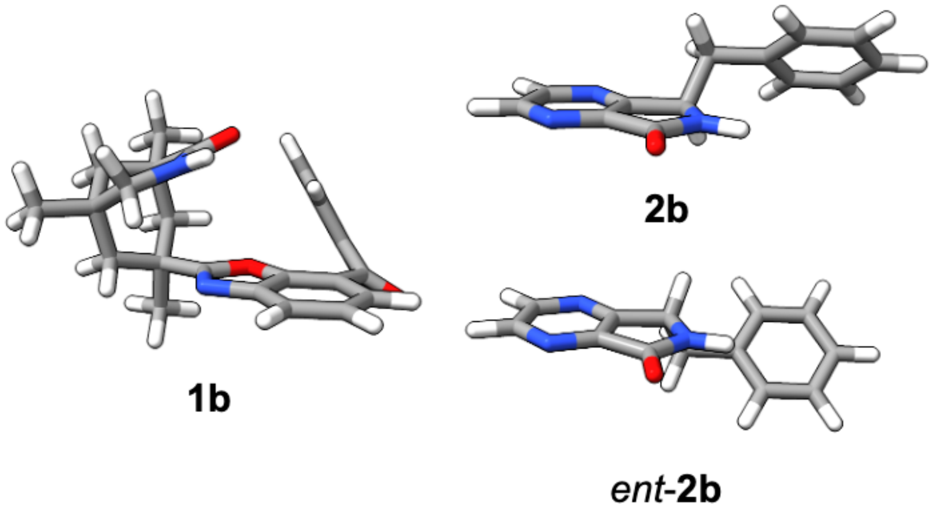

Figure S12: Energetically lowest geometries of the benzyl-substituted 4,7-diaza-1-isoindolinone enantiomers **2b** and *ent*-**2b** and the benzophenone catalyst **1b** optimized using PBEh-3c.

All geometries are initially optimized using the semiempirical electronic structure theory method GFN2-xTB[19] as implemented in the xtb code (version 6.6.1).[20] Implicit solvation effects are taken into account with the analytically linearized Poisson-Boltzmann (ALPB) model.[21] Since the experimentally used solvent α, α, α-trifluorotoluene is not parametrized for the ALPB model, we use dichloromethane (DCM) due to its similar dielectric constant.

These geometries are used for metadynamics-based (MTD) conformational sampling with the conformer rotamer ensemble sampling tool (CREST) (version 3.0.1)[22,23] using default settings with GFN2-xTB.

For the generation of noncovalent complexes, we first compute localized molecular orbitals (LMOs) using GFN1-xTB.[24] These orbitals are then used to construct the intermolecular force-field approach xTB-iFF[25] (version 1.1) to associate rigid fragments. All associated structures are successively optimized at the GFN2-xTB theory level.

Further geometry optimizations of the identified conformer ensembles (CE) are performed with the ORCA program (version 5.0.3)[26,27] addressing the GPU-accelerated electronic structure code TeraChem[28,29] for energy and gradient computations (keyword "extopt" in ORCA). A development version of TeraChem (based on version 1.9.2) is used, which includes several density functional theory (DFT) composite methods of the 3c-family.[30] To describe the

electronic ground state, restricted Kohn-Sham (RKS) DFT calculations are performed with the PBEh-3c composite method[31] and the conductor-like polarizable continuum (CPCM)[32] implicit solvation model (ϵ = 9.18) as implemented in TeraChem. PBEh-3c is a hybrid DFT method utilizing a modified def2-SVP[33] basis set (def2-mSVP) and a global hybrid functional based on the functional of Perdew, Burke and Ernzerhof (PBE)[34] with 42 % of non-local Fock exchange. As common for members of the "3c" methods, dispersion interactions are accounted for using a Grimme dispersion correction (D3[35,36] for PBEh-3c), and a geometric counter poise correction (gCP)[37] to address the basis set superposition error (BSSE).
T_1 minima are computed with unrestricted Kohn-Sham (UKS) DFT using PBEh-3c and the conductor-like polarizable continuum model (CPCM) for DCM as implemented in ORCA.[38]

Calculation of Free Energies
The Gibbs free energy of species i is computed according to:

$$G_i = E_{\mathrm{el},i} + G_{\mathrm{TRV},i} + \delta G_{\mathrm{solv},i}\ . \quad (1)$$

$E_{\mathrm{el},i}$ is the electronic gas phase energy computed at the PW6B95[39]-D4[40]/ def2-QZVPP[33]//PBEh-3c level of theory. For this single-point energy calculation, the resolution-of-the-identity (RI-J)[41,42] approximation for the evaluation of the Coulomb integrals with the def2/J[43] auxiliary basis set and the chain of spheres for exchange (COSX)[44] is used. Translational, rotational, and vibrational (TRV) contributions to the free energy are described in the modified rigid rotor-harmonic oscillator (mRRHO)[45] formalism. A standard state solvation correction $\delta G_{\mathrm{solv},i}$ from 1 mol of gas at 1 bar to a 1 M solution is computed at the PBEh-3c minimum geometries with GFN2-xTB and the ALPB model. Table **S6** lists the theory levels and details for computing the free energy contributions of the considered species.

Table S6: Employed levels of theory for the free energy calculations of each structure in this computational study.

**Geometry Optimizations**	**PBEh-3c + CPCM**
**Electronic energies $\boldsymbol{E_{\mathrm{el},i}}$**	PW6B95-D4/def2-QZVPP
**Nuclear contributions to the free energy $\boldsymbol{G_{\mathrm{TRV},i}}$**	PBEh-3c + CPCM(DCM) harmonic frequencies computed on the respective minima (scaled by 0.95[46]); particle-in-a-box, rigid rotor and modified harmonic oscillator model to account for translational, rotational and vibrational contributions to the nuclear free thermal energy. Harmonic frequencies below 50 cm^{-1} are handled by the interpolated free rotor-harmonic oscillator model.
**Solvation free energy correction $\boldsymbol{\delta G_{\mathrm{solv},i}}$**	$\delta G_{\mathrm{solv},i} = G_{\mathrm{GFN2\text{-}xTB/ALPB(DCM)},i} - E_{\mathrm{GFN2\text{-}xTB/gas},i}$ based on PBEh-3c geometries. This standard state correction is calculated for 1 mol of gas at 1 bar compared to a 1 M solution (keyword “bar1M” in xtb).

For the computation of free energies of triplet species, we use UKS-DFT with the above-mentioned theory level to compute electronic energies and harmonic frequencies. The standard state solvation corrections are based on an open-shell GFN2-xTB configuration (keyword "uhf 2") approximating the S_1/T_1 state correspondingly. The open-shell singlet/triplet states are indistinguishable at the GFN2-xTB level of theory, because the GFN2 Hamiltonian does not include spin-discriminating terms.[19,47] For a recent development on the incorporation of spin-polarization effects for the GFN1 and GFN2 methods, see Ref. [48] and [49].

We identify the most stable conformer based on the lowest free energy G_i within each CE. Throughout this work, all relative free energy differences between molecular species refer to these single conformers and we provide the corresponding contributions to the Gibbs energies in Table **S8** below. The used geometries and relevant input/output data for the calculations are provided in the esi-structures.zip file.

Association Energies

The identified free energy minima of complexes **2b·1b** and *ent*-**2b·1b** are illustrated in Figure **S13**.

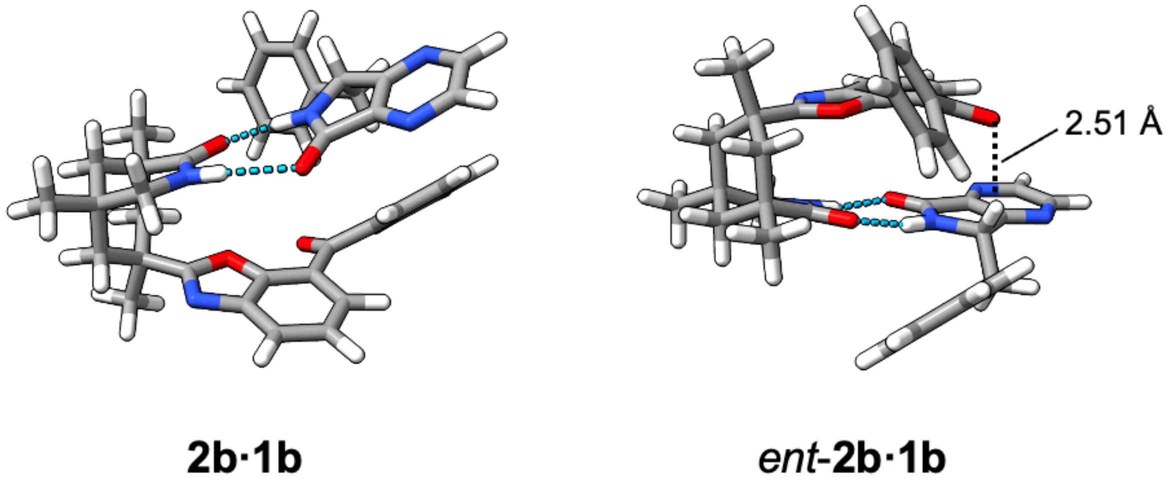

Figure S13: Lowest free energy (Eq. (1)) conformer of the substrate–catalyst complexes **2b·1b** (left) and *ent*-**2b·1b** (right). The oxygen-hydrogen bond distance along the hydrogen atom transfer coordinate is given for the reactive species, *ent*-**2b·1b** with 2.51 Å. In the shown complex geometry of **2b·1b**, this reaction coordinate is inaccessible.

Two-point hydrogen bonding to catalyst **1b** is feasible for both isoindolinone enantiomers as evident from the shown geometries. The thermodynamically most stable configuration of *ent*-**2b·1b** shows a bond distance between the benzophenone oxygen and the hydrogen on the chiral carbon center of $d_{OH} = 2.51$ Å. An analogous reaction coordinate is not present for **2b·1b** in its most stable conformation. Here, the hydrogen atom on the chiral center of **2b,** which is involved in the photochemically induced hydrogen atom shuttling process, is pointing away from the benzophenone group of **1b** and thus inaccessible.

The association free energies for the formation of both complexes are provided in Table **S7**.

Table S7: Computed association energies for the isoindolinones **2b** and *ent*-**2b** to the catalyst **1b**, and dimerization energies. The effective association energies, where the constituent dimerization is corrected for, are provided as well. Electronic energies refer to the PW6B95-D4/def2-QZVPP//PBEh-3c+CPCM theory level.

Reaction	ΔG (kJ·mol^{-1})	ΔG_{eff} (kJ·mol^{-1})
Catalyst Association		
**2b** + **1b**	−12	−1
ent-**2b** + **1b**	−18	−7
2×**1b**	−11	-
Substrate Association		
2×**2b**	−10	-
ent-**2b** + **2b**	−10	-

The association free energies of **2b**·**1b** and *ent*-**2b**·**1b** are negative with $\Delta G = -12$ kJ·mol^{-1} and -18 kJ·mol^{-1} respectively. Correcting for the substrates' dimerization, *ent*-**2b** still associates with **1b** with $\Delta G_{\mathrm{eff}} = -18$ kJ·mol^{-1} + $\frac{1}{2}$ (10 kJ·mol^{-1} + 11 kJ·mol^{-1}) ≈ −7 kJ·mol^{-1}, whereas only a negligibly small association energy of $\Delta G_{\mathrm{eff}} = -1$ kJ·mol^{-1} is found for **2b**·**1**.

The exergonic association energy to form *ent*-**2b**·**1b** suggests that a stable species, where the hydrogen shuttling can take place, is present in solution. After this species is promoted to an excited state, the benzophenone group can act as acceptor for the photochemically induced hydrogen abstraction as will be clarified in the following.

Forward Hydrogen Atom Transfer

For benzophenone catalyst **1a**, photochemically induced hydrogen atom transfer (HAT) has already been investigated by some of us.[50] Here we take up on the proposed mechanism proceeding via a triplet state, which is reached through intersystem crossing (ISC) from an excited singlet state (S_1). After ISC to the T_1 state, forward HAT is expected to occur, where the hydrogen atom on the chiral carbon center is transferred to the catalysts benzophenone group (see below). In the following, we outline the methodology used to investigate further steps involved in photochemical conversion of *ent*-**2b** in the presence of **1b**.

Identification of Transition States

To identify a T_1 transition state (TS) of the forward HAT reaction step, we perform distance scans along the HAT reaction coordinate using an open-shell configuration with GFN2-xTB as stated above. This is done to generate reasonable guess geometries for successive TS optimization with ORCA using eigenvector following (keyword "opts").[51] For this step, the self consistent field (SCF) convergence criteria are tightened and adjusted settings for the direct inversion of the iterative subspace (DIIS) [52,53] convergence scheme (keywords "tightscf" and "slowconv") are used.

The TS is verified by means of its single imaginary frequency. An intrinsic reaction coordinate (IRC)[54] computation is used to confirm that the reactants are connected via the found TS.

Figure **S14** shows the T_1 minimum, the identified transition state and the achiral isoindolinone compound **4b** resulting from the first hydrogen shuttling.

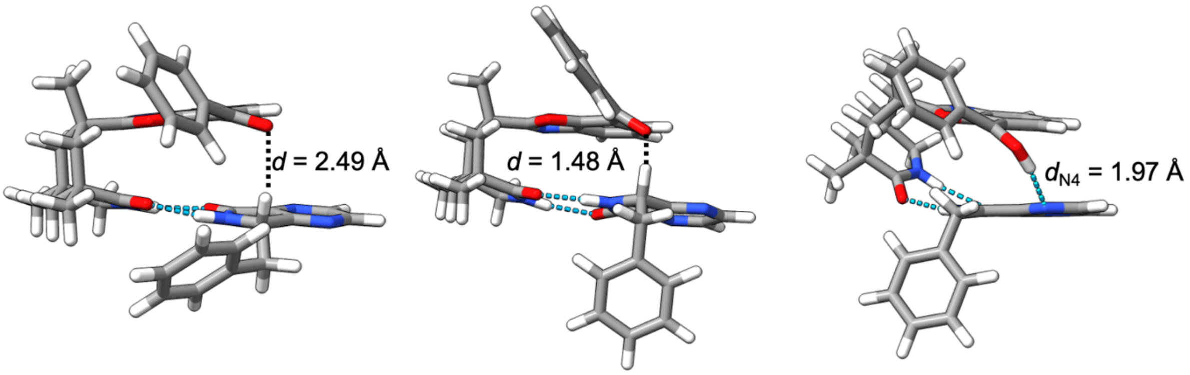

Figure S14: T_1 minimum geometry of *ent*-**2b**·**1b** prior to forward HAT (left), the corresponding TS for forward HAT (center) and the resulting substrate–catalyst complex with achiral **4b** (right). For the T_1 (*ent*-**2b**·**1b**) to the TS, the bond distance from the benzophenone oxygen to the hydrogen atom at the chiral center is reduced from 2.49 Å to 1.48 Å. After HAT, hydrogen bonding interactions of the hydroxy group of the catalyst with the nitrogen at 4-position of **4b** is highlighted (d_{N4} = 1.97 Å).

At the T_1 minimum, d_{OH} amounts to 2.49 Å, which is only subordinately smaller than the distance found at the S_0 minimum (d_{OH} = 2.51 Å, Fig. S2). At the T_1 TS, this distance is reduced to 1.48 Å. After forward HAT, a hydrogen bonding interaction with the isoindolinone nitrogen atom at 4-position is observed at the T_1 minimum (**4b**·**1b'**) with a bond distance of d_{N4} = 1.97 Å. This interaction hints at a position to which backward HAT might be feasible.

Complex Geometries of Catalyst 1a

To understand the drastic differences in the *ee* obtained using catalyst **1a** in contrast to **1b**, we investigated its complex geometry with *ent*-**2b**, *ent*-**2b**·**1a** in the ground and excited T_1 state. Figure **S15** shows the S_0 complex geometries of the noncovalent complexes with bond distances for forward HAT highlighted.

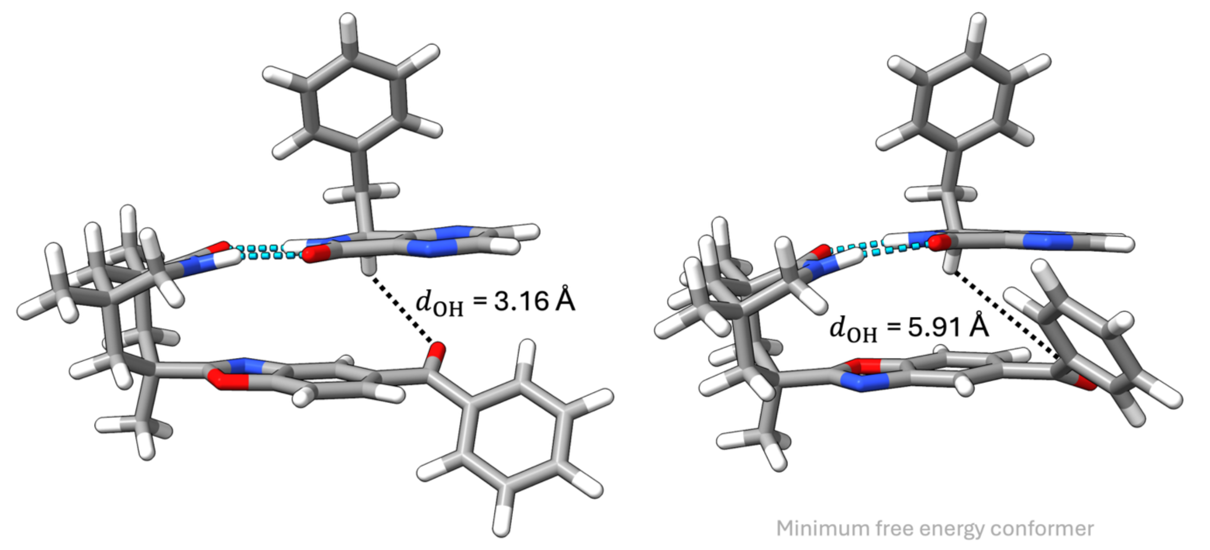

Figure S15: Ground state complex geometries of *ent*-**2b**·**1a** computed at the RKS-PBEh-3c+CPCM(DCM) theory level. A selected conformer showing the smallest O–H bond distance along the forward HAT reaction coordinate (d_{OH} = 3.16 Å, left) and the free energy minimum conformer (d_{OH} = 5.91 Å, right) are presented. The free energy difference between the shown conformers computed according to Eq. (1) with the theory levels outlined in Tab. **S6** amounts to +6.0 kJ·mol^{-1}.

Comparing complex *ent*-**2b**·**1a** to the analogue involving **1b** shown in Figure **S13** and **S14**, we find clear differences in the O–H bond distances for forward HAT d_{OH}.

The smallest d_{OH} among the identified conformers amounts to 3.16 Å for *ent*-**2b**·**1a** (Fig. **S15**) in contrast to *ent*-**2b**·**1b** with 2.51 Å. At the T_1 minimum, d_{OH} of *ent*-**2b**·**1a** is slightly elongated (3.19 Å) and the free energy difference with respect to the free energy minimum conformer increases (ΔG = +8.2 kJ·mol^{-1}) in contrast to the electronic ground state (ΔG = +6.0 kJ·mol^{-1}), which is shown above.

For these reasons, we argue that the energy barrier for HAT to **1a** in *ent*-**2b**·**1a** is energetically disfavored in contrast to *ent*-**2b**·**1b** (see also Fig. **S13** and below).

Back Hydrogen Atom Transfer

While the interaction of the benzophenone OH group in **4b·1b'** with the nitrogen at 4-position in Figure **S14** suggests one product of backward HAT, we aim to identify further possible products of backward HAT in an unbiased manner, that is, without prior knowledge from the performed distance scan. To do so, we first employ the minimum energy crossing point (MECP) search algorithm as implemented in CREST.[55,56]

The MECP screening algorithm follows a derivative coupling vector-free formalism. Here, an artificial seam potential energy surface (PES) is created from the mean of two GFN2-xTB states, the closed-shell S_0 and open-shell configuration (approximating the S_1/T_1 state). An energy gap-dependent bias potential is applied next, which drives the energy between the two states towards zero and converges to a targeted crossing point of the PESs.[56] Using this approach, a diverse set of S_0/T_1 MECP guess structures is generated in a computationally efficient manner.

The set of structures obtained this way is relatively large for moderately-sized systems such as *ent*-**2b·1** (84 atoms) already. For this reason, we employ the molecular identifier MolBar[57,58] for prior categorization of the structures. This is done to avoid unnecessary MECP reoptimizations at DFT level. MolBar groups the structures into ensembles containing the same molecular identifier, hence allowing to filter unfeasible MECP candidates beforehand. With these filtered MECP guesses, two separate geometry refinements are performed:

- UKS-PBEh-3c T_1 optimizations to see whether backward HAT occurs on the triplet PES.
- HAT transfer via the S_0/T_1 MECPs to S_0 is described reoptimizing the MECPs guesses with UKS-DFT at the PBEh-3c+CPCM(DCM) theory level first. CREST addresses TeraChem for the UKS-DFT gradient computations ("generic" runmode) in this derivative coupling vector-free MECP optimization.[54] Successively, an RKS-DFT S_0 optimization is performed.

The former approach did not provide evidence for back HAT on the T_1 PES. However, with the latter optimizations, products where the hydrogen atom is transferred back to the isoindolinone, are observed. The identified products of back HAT are illustrated in Figure **S16**.

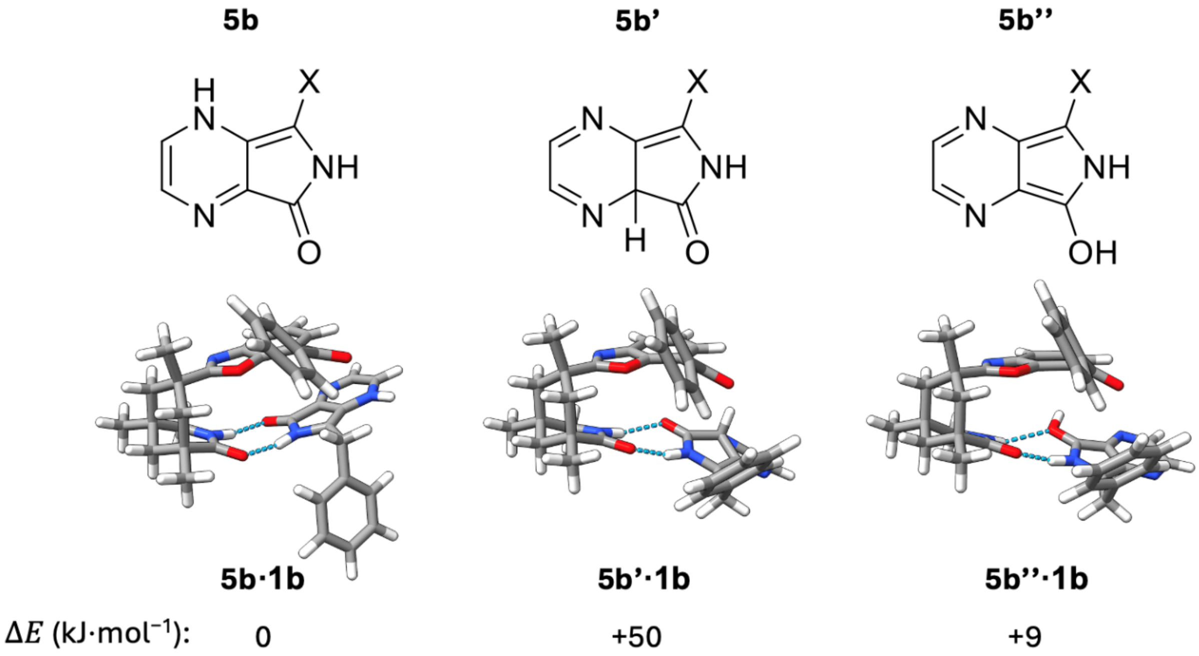

Figure S16: Identified back hydrogen atom transfer products **5b**, **5b'** and **5b"** associated to **1b**. The structures are obtained from S_0 optimization starting from the reoptimized UKS S_0/T_1 minimum energy crossing points. The relative electronic energy differences ΔE at the PBEh-3c + CPCM(DCM) theory level are given.

Among the identified products, product **5b** is thermodynamically most stable. The other products **5b'** and **5b"** show a higher electronic energy of $\Delta E = +50$ kJ·mol^{-1} (**5b'**) and +9 kJ·mol^{-1} (**5b"**) in their complex geometry relative to **5b**·**1b**. Since the latter products are still energetically below the **4b**·**1b'** triplet intermediate (Fig. **2**), a product distribution between these three may be observed for thermodynamic reasons. However, for the formation of **5b"**, a rotation of **4b** in the absence of one of the two hydrogen bonds has to occur, which is unlikely from a kinetic point of view. We, thus, assume that exclusively **5b** will be formed in the back HAT step.

Description of Excited States

To study the photophysical properties of the involved molecules, we first compute vertical excitation energies using Tamm-Dancoff approximated (TDA) DFT with the PBEh-3c composite method and the CPCM solvation model. Figure **S17** contains the vertical excitation energies for *ent*-**2b**, **1b** and the corresponding noncovalent complexes **2b**·**1** and *ent*-**2b**·**1**.

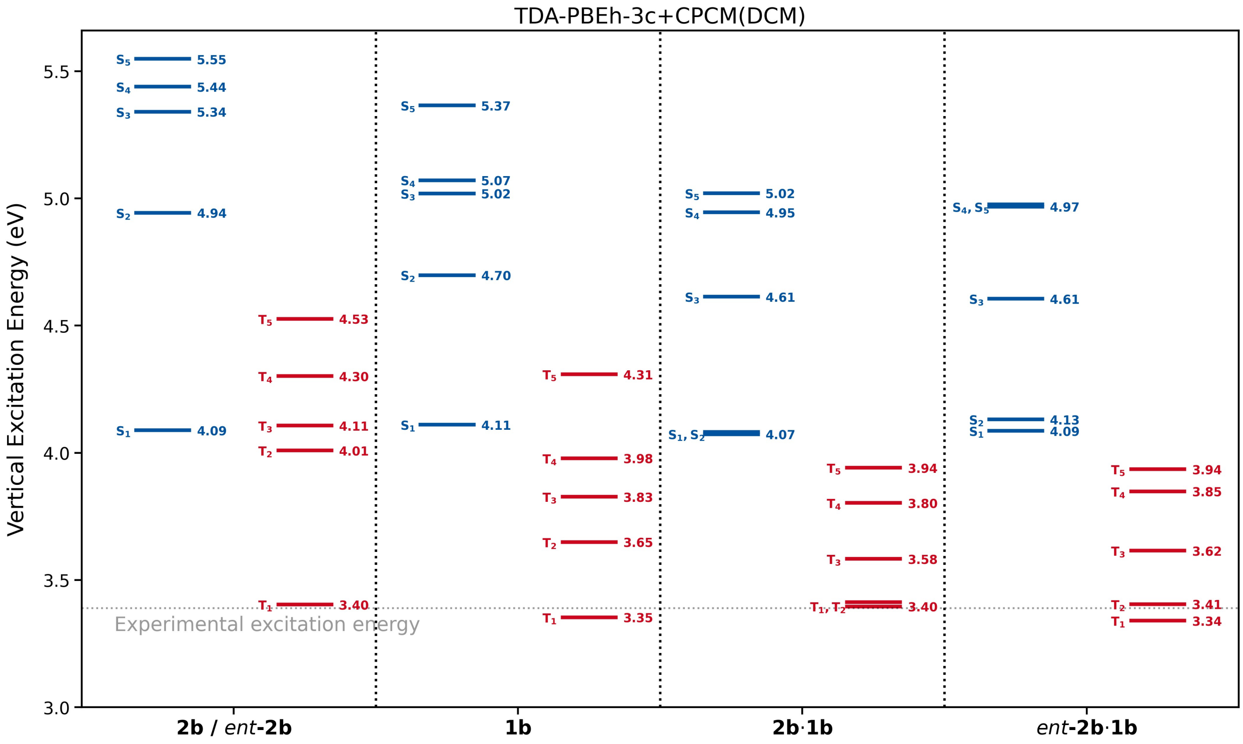

Figure S17: TDA-PBEh-3c+CPCM(DCM) vertical excitation energies (in eV) of *ent*-**2b**, **1b** and the corresponding complexes *ent*-**2b**·**1b**. The experimental excitation wavelength (λ = 366 nm ≈ 3.4 eV) is drawn for comparison.

The vertical excitation energies are generally overestimated with TDA-DFT. This can be recognized since the lowest excited singlet state(s) of neither species are energetically accessible given the experimental excitation wavelength of 3.4 eV. Hence, the S_1 could not be populated upon irradiation with light. Among other causalities, TDA-DFT does not account for multiply excited determinants and therefore misses important orbital relaxation effects. The employed linear response (LR-)CPCM solvation model does not properly account for important (state-specific) contributions.[59,60]

However, first qualitative information of the lowest excited states can be deduced using TDA-DFT. *ent*-**2b** and **1b** show similar vertical S_1 energies at the chosen theory level. In the corresponding complex geometries, the S_1 states are quasi-degenerate, and no significant energy splitting is observed. The natural transition orbitals (NTOs)[61] associated with the largest orbital contributions for the S_1 and S_2 states are illustrated in Figure **S18**.

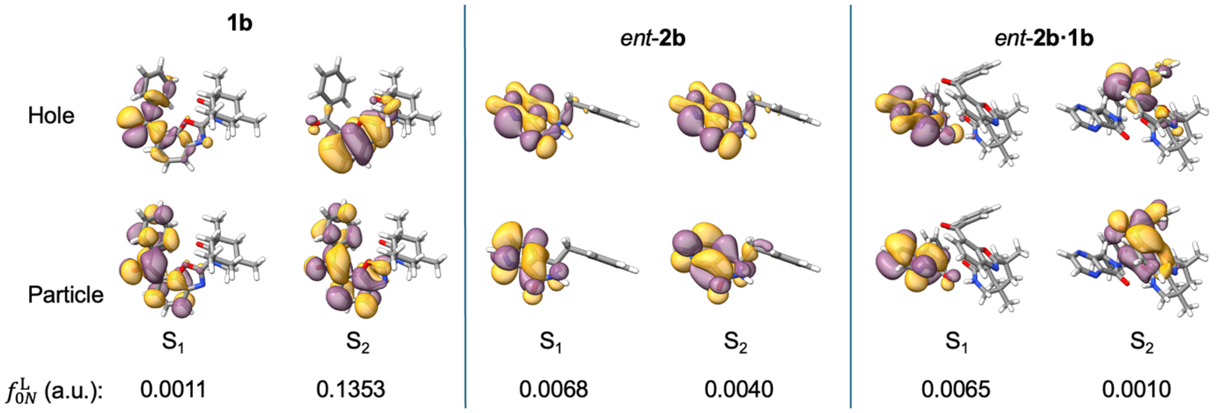

Figure S18: Dominant natural transition orbitals for the S_1 and S_2 excited states of **1b** (left), *ent*-**2b** (center) and **2b·1b** (right) computed at the TDA-PBEh-3c + CPCM(DCM) level of theory at the ground state geometry. All isosurfaces are shown with a contour value of ± 0.02. Oscillator strength using the length formalism of the electric transition dipole moment f^{L}_{0N} are given for all states.

The dominant hole and particle NTOs for the S_1 state of **1b** show $n \rightarrow \pi^*$ character, which is also evident from the relatively small oscillator strength f^{L}_{01}= 0.0011. The S_2 state is mostly comprises $\pi \rightarrow \pi^*$ excitation (f^{L}_{02}= 0.1353). For *ent*-**2b**, both states show $n \rightarrow \pi^*$ character with f^{L}_{0N} of 0.0068 (S_1) and 0.0040 (S_2) respectively. In complex *ent*-**2b·1b**, we find similar fragment localized states on the respective moieties *ent*-**2b** (S_1) and **1b** (S_2) that correspond to the S1 states of the individual fragment.

In **1b** and *ent*-**2b** the S_2 state is about is about 0.60 eV and 0.85 eV higher in energy than the S_1 state, respectively. We argue that at the chosen irradiation wavelength, only the first excited singlet state is populated. In *ent*-**2b·1b**, both $n \rightarrow \pi^*$ transitions are energetically close and either state may be accessible.

For a more accurate assessment of the vertical excitation energies, we use DFT in combination with multireference configuration interaction (DFT/MRCI).[62] Here, the newest available Hamiltonian (R2022[63]) with short parametrization and the corresponding energy threshold of 0.8 E_h to treat configuration state functions (CSFs) (denoted as "short settings" in the following) is used. The closed-shell anchor configuration is computed using Turbomole (version 7.6)[64] with the BHLYP[65] exchange–correlation (XC) functional and a def2-SV(P)[33] basis set. The command line tool cefine[66] tool is used to generate the required input files. Throughout, the resolution of the identity for Coulomb integrals (RI-J and RI-C) is employed[42,67] with corresponding auxiliary basis set.[43] An initial configuration interaction (CI) reference space is generated from a (4,4) active space restricted to single and double excitations.

Orbitals with energies ϵ outside the interval $-10.0\ \mathrm{E_h} < \epsilon < 3.0\ \mathrm{E_h}$ of the BHLYP/def2-SV(P) anchor configuration are kept frozen for the MRCI. Since the computed excitation energies slightly depend on the requested number of roots[68], we set these to five excited states for both singlet and triplet states consistently.

In Figure **S19**, the vertical excitation energies computed with DFT/MRCI are shown.

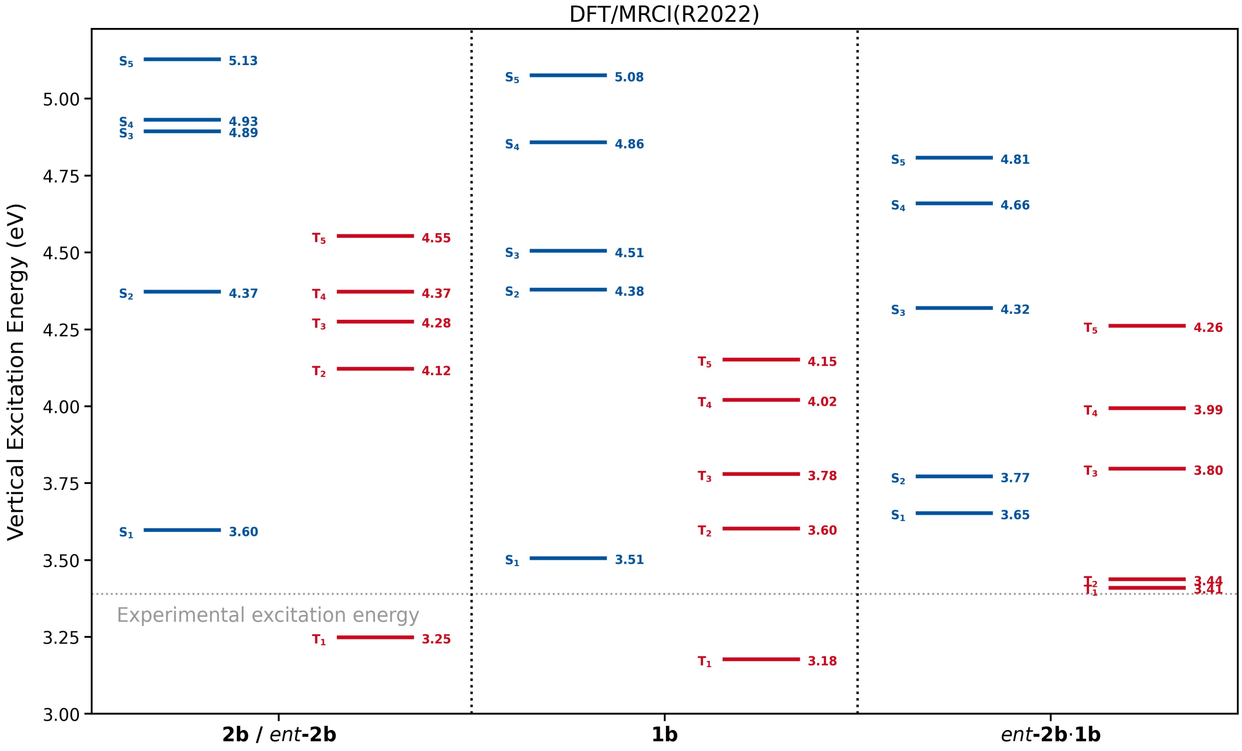

Figure S19: Vertical excitation energies of free *ent*-**2b**, **1b** and the corresponding complexes computed with DFT/MRCI(R2022) using short settings ("short" parameters and $E_{\mathrm{cut}} = 0.8\ \mathrm{E_h}$). The reference configuration is computed at the BHLYP/def2-SV(P)//PBEh3c+CPCM theory level.

The lowest singlet excitation energies are reduced by about 0.50 eV compared to TDA-DFT and are closer to the experimental irradiation wavelength. Assessments of DFT/MRCI suggest that vertical excitation energies deviate about <0.2 eV.[68] We cannot differentiate whether prior catalyst excitation is favored overpopulating the substrate localized n → π* state directly in *ent*-**2b**·**1**. Based on our calculations, direct excitation of *ent*-**2b** is possible and the substrate-localized state can be populated.

The above assignment of n → π∗ transitions is also supported from the oscillator strength computed using DFT/MRCI. For the $S_0 \rightarrow S_1$ and $S_0 \rightarrow S_2$ transitions, these amount to 0.0083 and 0.0025 respectively.

To further understand, which states are responsible for the observed photochemical conversion, we follow a state-specific approach, namely ΔSCF[69] to describe the S_1 state. We use the ΔSCF implementation available with ORCA version 6.0.0. Using the maximum overlap method

(MOM),[70] the SCF can converge to an open-shell singlet solution by maximizing the overlap between the occupied orbitals in each iteration with respect to a given set of reference orbitals. It is worth noting that the resulting wavefunction does not represent a spin-pure state and only approximates the S_1 singlet state. We use the UKS T_1 wavefunction as initial guess for the S_1 computations (IMOM-PBEh-3c + CPCM).

To assess how the excited states are positioned at the orbital-optimized theory level, we consider the spin (ΔUKS or UKS) or difference densities (TDA-DFT) of the S_1 and T_1 states (Figure **S20**).

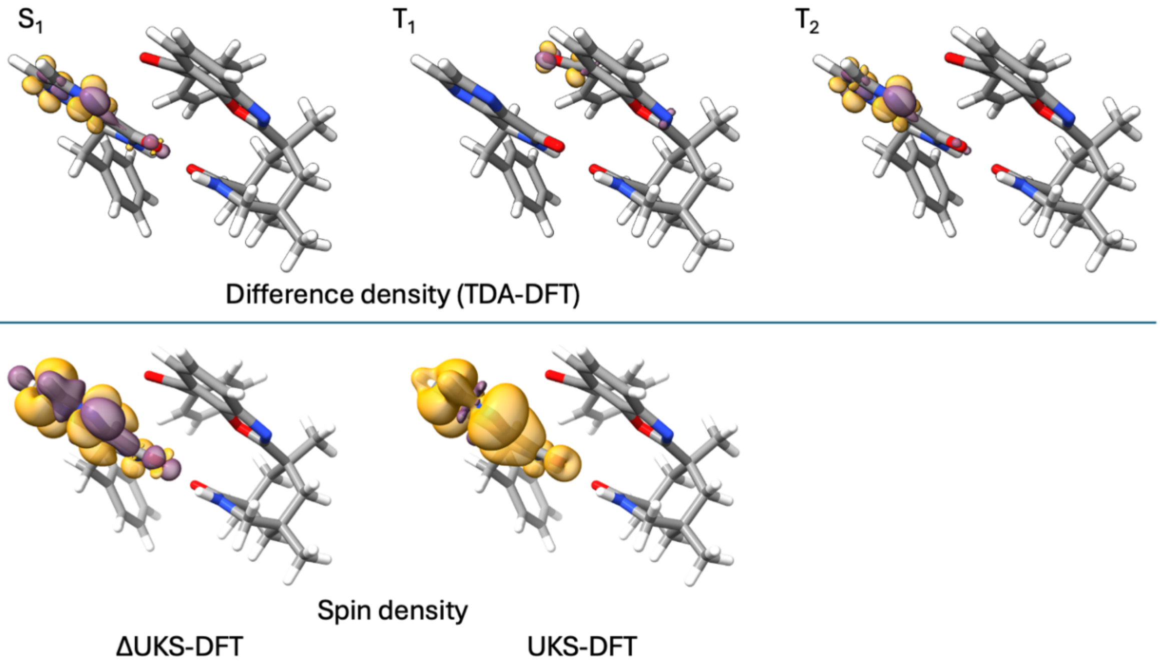

Figure S20: Top: Difference densities between the ground and excited state (S_1 left, T_1 center T_2, right) computed with TDA-DFT. Bottom: Spin densities of *ent*-**2b**·**1b** for the S_1 state (left) computed with ΔUKS- and the T_1 state (right) using UKS-DFT. All calculations refer to the PBEh-3c + CPCM theory level and are performed on the ground state geometry. Isosurfaces are plotted with contour values of ±0.01.

The identified S_1 state looks similar with ΔUKS- and TDA-DFT. The S_1 state is localized on the substrate moiety. Similar to the S_1 state is the T_1 computed using UKS-DFT. With TDA-DFT, the T_1 state shows small orbital contributions on the benzophenone oxygen on **1b**. The T_2 difference densities on the other hand, again resemble the UKS T_1 state closely. Thus, it is possible that the ordering of the T_1 and T_2 states is interchanged at the TDA-DFT level of theory. With the ΔUKS S_1 and the RKS ground state energy, the vertical excitation energy amounts to 3.67 eV, which is close to the value computed using DFT/MRCI. Since ΔUKS cures some flaws of TDA-DFT and DFT/MRCI like partially missing orbital relaxation or a state-specific solvation description, the similarity of the reported results assures a good theoretical description

of the S_1 state with ΔUKS. For this reason, we take the latter method for the results reported in Figure 1 in the main article.

In regard to measured absorption spectra of **1b** and *ent*-**2b**, where a significant spectral overlap is observed, the energetic proximity of the fragment localized states appears reasonable. A comparison of the measured fluorescence and phosphorescence spectra of *ent*-**2b** renders direct ISC of the substrate to a triplet species feasible. For these reasons, we expect that the substrate localized triplet state is accessible from direct irradiation in presence of **1b**, but potentially also via energy transfer from **1b** to *ent*-**2b**.

In its excited state, the C–H bond in *ent*-**2b** is destabilized and due to the spatially proximate hydrogen acceptor **1b**, hydrogen shuttling process can take place.

Gibbs Free Energy Contributions

Table S8: Free energy contributions for the species investigated in this study (cf. Eq (1)), divided into isolated (monomeric) and associated (dimeric) species. The values refer to the theory level outlined in Table **S6**. [a]: Energies are instead computed at the RKS- or ΔUKS-PBEh-3c+CPCM(DCM) theory level in ORCA, as outlined above. [b]: Energetically higher-lying conformer. BS: Broken symmetry.

Species	State	E_{el} (E_h)	G_{TRV} (E_h)	δG_{solv} (E_h)	G (E_h)
		Monomeric Species			
**1b**	S_0	−1305.838250	0.404690	−0.037266	−1305.470826
	T_1	−1305.734259	0.401567	−0.038635	−1305.371327
ent-**2b**	S_0	−742.793247	0.176266	−0.023688	−742.640669
	T_1	−742.682507	0.169998	−0.023797	−742.536307
		Dimeric Species			
**2b**·**2b**	S_0	−1485.616704	0.376974	−0.045390	−1485.285120
ent-**2b**·**2b**	S_0	−1485.615588	0.376483	−0.046151	−1485.285257
**1b**·**1b**	S_0	−2611.708037	0.835904	−0.073711	−2610.945843
**2b**·**1b**	S_0	−2048.661383	0.606047	−0.060608	−2048.115945
	T_1	−2048.550152	0.600216	−0.060580	−2048.010515
ent-**2b**·**1b**	S_0 [a]	−2040.694672	-	-	-
	S_1 [a]	−2040.558917	-	-	-
	S_0	−2048.664158	0.605886	−0.060262	−2048.118534
	T_1	−2048.551926	0.599248	−0.061251	−2048.013930
ent-**2b**·**1b** (TS)	T_1	−2048.554685	0.600687	−0.058360	−2048.012357
**4b**·**1b**	T_1	−2048.609917	0.604369	−0.055324	−2048.060872
	S_0^*(BS)	−2048.610583	0.606401	−0.055145	−2048.059327
ent-**2b**·**1a**	S_0	−2048.667954	0.606744	−0.058991	−2048.120201
	S_0 [b]	−2048.664746	0.606589	−0.059778	−2048.117935
	T_1	−2048.557083	0.600618	−0.058461	−2048.014926
	T_1 [b]	−2048.552733	0.600552	−0.059623	−2048.011804
**5b**·**1b**	S_0	−2048.630727	0.605346	−0.061148	−2048.086529
**5b’**·**1b**	S_0	−2048.617219	0.604441	−0.060050	−2048.072829
**5b”**·**1b**	S_0	−2048.631761	0.604483	−0.059949	−2048.087227

11. Substrate Synthesis

Methyl 3-(bromomethyl)pyrazine-2-carboxylate (SI-8)

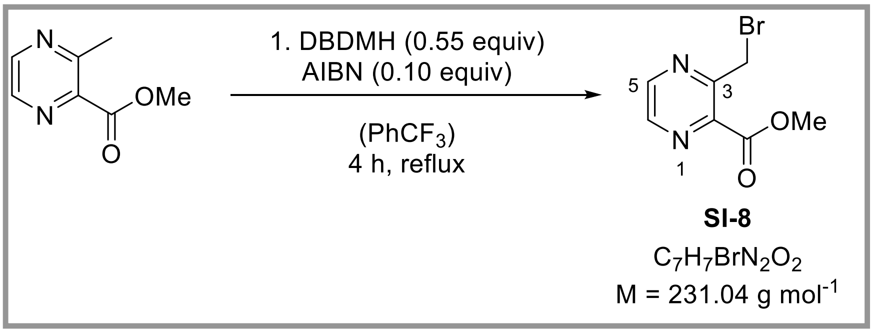

According to a literature procedure,[71] to a solution of methyl 3-methylpyrazine-2-carboxylate (2.50 g, 16.4 mmol, 1.00 equiv.) in $PhCF_3$ (48 mL) was added 1,3-dibromo-5,5-dimethylhydantoin (DBDMH) (2.58 g, 9.04 mmol, 0.55 equiv.) and azobis(isobutyonitril) (AIBN) (270 mg, 1.64 mmol, 0.10 equiv.). The resulting suspension was refluxed for 4 h. Afterwards, the now slightly orange solution was allowed to cool to room temperature and the solution was filtered through cotton. Subsequently, the solvent was removed and the residual crude product was subjected to FCC (SiO_2, Pn:EtOAc = 5:2 → 2:1) to yield **SI-8** (860 mg, 3.72 mmol, 23%) as a yellow oil.

TLC (Pn:EtOAc = 3:1): R_f = 0.23 [UV] [$KMnO_4$].

^{1}H-NMR (400 MHz, $CDCl_3$, 300 K): δ [ppm] = 8.69 (d, ^3J = 2.3 Hz, 1H, H5), 8.66 (d, ^3J = 2.3 Hz, 1H, H6), 5.05 (s, 2H, CH_2Br), 4.08 (s, 3H, $COOCH_3$).

^{13}C-NMR (101 MHz, $CDCl_3$, 300 K): δ [ppm] = 164.8 (COO), 154.0 (C2), 146.5 (C5), 143.4 (C6), 142.3 (C3), 53.5 (CH_3), 30.5 (CH_2Br).

IR (film): $\tilde{\nu}$ max/cm^{-1} = 3074 (w, CH_{arom}), 2954 (w, CH_{aliph}), 1715 (s, C=O), 1611 (m, $C=C_{arom}$), 1377 (m, C=Car).

6-(4-Methoxybenzyl)-6,7-dihydro-5*H*-pyrrolo[3,4-*b*]pyrazin-5-one (SI-9)

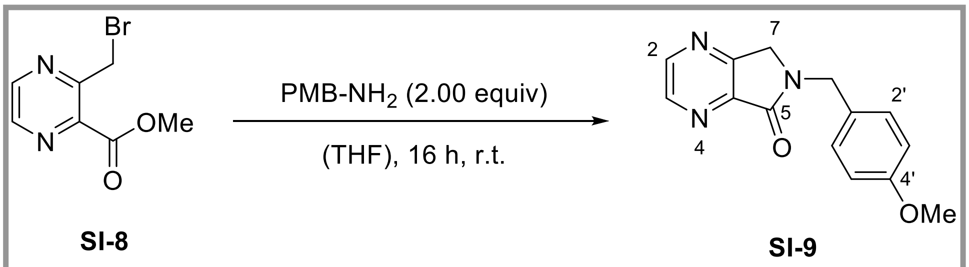

$C_{14}H_{13}N_3O_2$
M = 255.27 g mol^{-1}

According to a literature procedure,[72] to a solution of **SI-8** (780 mg, 3.38 mmol, 1.00 equiv.) in THF (10 mL) was added 4-methoxybenzylamine (PMB-NH_2) (882 µL, 926 mg, 6.75 mmol, 2.00 equiv.) at room temperature in one portion. The resulting suspension was stirred for 16 h at the same temperature. The reaction was quenched by the addition of sat. ammonium chloride solution (20 mL) and water (20 mL) and the mixture was extracted with EtOAc (3 × 50 mL). The combined organic layers were washed with brine (50 mL) and dried over Na_2SO_4. After filtration, the solvent was removed under reduced pressure and the residual crude product was subjected to FCC (SiO_2, Pn:EtOAc = 2:1 → 0:1) to yield the desired 6-(4-methoxybenzyl)-6,7-dihydro-5*H*-pyrrolo[3,4-*b*]pyrazin-5-one **SI-9** (858 mg, 3.36 mmol, *quant.*) as an off-white solid.

TLC (EtOAc): R_f = 0.33 [UV] [$KMnO_4$].

M.p.: 182 °C.

^{1}H-NMR (400 MHz, $CDCl_3$, 300 K): δ [ppm] = 8.76 (d, ^3J = 2.7 Hz, 1H, H2), 8.63 (d, ^3J = 2.7 Hz, 1H, H3), 7.32– 7.23 (m, 2H, H2’, H6’), 6.92– 6.84 (m, 2H, H3’, H5’), 4.84 (s, 2H, CH_2), 4.34 (s, 2H, H7), 3.79 (s, 3H, OCH_3).

^{13}C-NMR (101 MHz, $CDCl_3$, 300 K): δ [ppm] = 164.7 (C5), 159.6 (C4’), 156.4 (C4a), 146.3 (C3), 146.0 (C2), 145.2 (C7a), 130.0 (C2’/C6’), 128.1 (C4’), 114.5 (C3’/C5’), 55.5 (OCH3), 48.6 (C7), 46.4 (CH_2).

HRMS (ESI) m/z: Calculated for $[M+H]^+$: 256.1081 found: 256.1087.

IR (film): $\tilde{v}$ max/cm^{-1} = 3074 (w, CH_{arom}), 2956 (w, CH_{aliph}), 1694 (s, C=O), 1611 (m, $C=C_{arom}$), 1377 (m, C=Car).

7-(4-(*tert*-Butyl)benzyl)-6,7-dihydro-5*H*-pyrrolo[3,4-*b*]pyrazin-5-one (*rac*-2a)

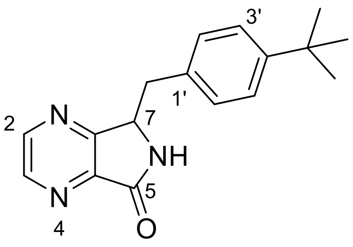

rac-**2a**
$C_{17}H_{19}N_3O$
M = 281.36 g mol^{-1}

According to GP A, a 1 M solution of KHMDS (1.08 mL, 216 mg, 1.08 mmol, 1.10 equiv.) was added dropwise to a suspension of 6-(4-methoxybenzyl) 6,7-dihydro-5*H*-pyrrolo[3,4-*b*]pyrazin-5-one **SI-9** (250 mg, 979 µmol, 1.00 equiv.) in anhydrous THF (30 mL) at –78 °C. After 20 min, 1-(bromomethyl)-4-(tert-butyl)benzene (234 µL, 289 mg, 1.27 mmol, 1.30 equiv.) was added in one portion. The reaction was left to stir for 4 h at –78 °C. Afterwards, the solution was allowed to warm up to room temperature and quenched by the addition of sat. NH_4Cl (6 mL) and distilled water (20 mL). The aqueous layer was extracted with CH_2Cl_2 (3 × 25 mL), and the combined organic phases were dried over Na_2SO_4, filtered and concentrated under reduced pressure. The crude product was then dissolved in MeCN:H_2O (3:1) (29 mL) and diammonium cerium(IV) nitrate (1.61 g, 2.94 mmol, 3.00 equiv.) was added to the solution at room temperature. The yellow solution was stirred for 90 min at the same temperature. Subsequently, water (30 mL) was added, and the mixture was extracted with CH_2Cl_2 (3 × 50 mL). The combined organic layers were washed with brine (50 mL) and dried over Na_2SO_4. After filtration, the solvent was removed under reduced pressure and the residual crude product was subjected to FCC (SiO_2, EtOAc) to yield the desired racemic substrate *rac*-**2a** (64 mg, 228 µmol, 23%) as a white solid**.**

TLC (EtOAc): R_f = 0.21 [UV] [$KMnO_4$].

M.p.: 249 °C.

^{1}H-NMR (400 MHz, $CDCl_3$, 300 K): δ [ppm] = 8.79 (d, ^3J = 2.6 Hz, 1H, H2), 8.73 (d, ^3J = 2.6 Hz, 1H, H3), 7.38 – 7.30 (m, 2H, H2', H6'), 7.19 – 7.12 (m, 2H, H3', H5'), 6.86 (s, 1H, NH), 4.88 (dd, ^2J =9.7 Hz, ^3J = 3.5 Hz, 1H, H7), 3.60 (dd, ^2J = 13.8 Hz, ^3J = 3.7 Hz, 1H, C7-CH_a-C1'), 2.72 (dd, J = 13.8, 9.7 Hz, 1H, C7-CH_b-C1'), 1.30 [s, 9H, $C(CH_3)_3$].

^{13}C-NMR (101 MHz, $CDCl_3$, 300 K): δ [ppm] = 166.1 (C5), 160.2 (C7a), 150.6 (C4'), 146.7 (C3), 146.2 (C2), 144.6 (C4a), 132.9 (C1'), 129.0 (C3', C5'), 126.1 (C2', C6'), 57.6 (C7), 38.8 (C3-CH$_2$-C1'), 34.6 [C(CH_3)$_3$], 31.4 [C(CH$_3$)$_3$].

HRMS (ESI) m/z: Calculated for $[M+H]^+$: 282.1601; found: 282.1600.

IR (film): $\tilde{\nu}_{max}$/cm^{-1} = 3307 (m, NH), 3062 (m, CH_{arom}), 2955 (m, CH_{aliph}), 1704 (s, C=O) 1385 (m, $C{=}C_{arom}$), 1161 (m, CN), 823 (m, CH_{arom}).

7-Benzyl-6,7-dihydro-5*H*-pyrrolo[3,4-*b*]pyrazin-5-one (*rac*-2b)

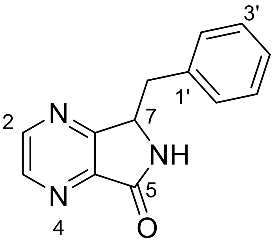

rac-**2b**
$C_{13}H_{11}N_3O$
M = 225.25 g mol^{-1}

According to GP A, a 1 M solution of KHMDS (1.08 mL, 216 mg, 1.08 mmol, 1.10 equiv.) was added dropwise to a suspension of 6-(4-methoxybenzyl) 6,7-dihydro-5*H*-pyrrolo[3,4-*b*]pyrazin-5-one **SI-9** (250 mg, 979 µmol, 1.00 equiv.) in anhydrous THF (30 mL) at –78 °C. After 20 min, benzyl bromide (151 µL, 217 mg, 1.27 mmol, 1.30 equiv.) was added in one portion. The reaction was left to stir for 4 h at –78 °C. Afterwards, the solution was allowed to warm up to room temperature and quenched by the addition of sat. NH_4Cl (6 mL) and distilled water (20 mL). The aqueous layer was extracted with CH_2Cl_2 (3 × 25 mL), and the combined organic phases were dried over Na_2SO_4, filtered and concentrated under reduced pressure. The crude product was then dissolved in MeCN:H_2O (3:1) (29 mL) and diammonium cerium(IV) nitrate (1.61 g, 2.94 mmol, 3.00 equiv.) was added to the solution at room temperature. The yellow solution was stirred for 90 min at the same temperature. Subsequently, water (30 mL) was added, and the mixture was extracted with CH_2Cl_2 (3 × 50 mL). The combined organic layers were washed with brine (50 mL) and dried over Na_2SO_4. After filtration, the solvent was removed under reduced pressure and the residual crude product was subjected to FCC (SiO_2, EtOAc) to yield the desired racemic substrate *rac*-**2b** (61 mg, 272 µmol, 28%) as a white solid.

TLC (EtOAc): R_f = 0.18 [UV] [$KMnO_4$].

M.p.: 217 °C.

^{1}H-NMR (400 MHz, $CDCl_3$, 300 K): δ [ppm] = 8.75 (d, ^{3}J = 2.6, 1H, H2), 8.71 (d, ^{3}J = 2.6 Hz, 1H, H3), 7.82 (bs, 1H, NH), 7.25 – 7.16 (m, 3H, H2', H4', H6'), 7.16 – 7.11 (m, 2H, H3', H5'), 4.97 – 4.89 (dd, ^{2}J = 8.1 Hz, ^{3}J = 4.1 Hz, 1H, H7), 3.55 (dd, ^{2}J = 13.9 Hz, ^{3}J = 4.1 Hz, 1H, C7-CH_a-C1'), 2.94 (dd, J = 13.9, 8.1 Hz, 1H, C7-CH_b-C1').

^{13}C-NMR (101 MHz, $CDCl_3$, 300 K)**:** δ [ppm] = 166.7 (C5), 160.2 (C7a), 146.6 (C3), 146.1 (C2), 144.7 (C4a), 135.5 (C-1'), 129.5 (C-3', C-5'), 128.8 (C-2', C-6'), 127.4 (C-4'), 57.4 (C7), 38.8 (C7-*C*H_2-C1').

HRMS (ESI) Calculated for $[M+H]^+$: 226.0975; found: 226.0974.

IR (film) $\tilde{\nu}_{max}/cm^{-1}$**:** 3207 (m, NH), 3031 (m, CH_{arom}), 2918 (m, CH_{aliph}), 1710 (s, C=O) 1377 (m, $C{=}C_{arom}$), 1156 (m, CN), 802 (m, CH_{arom}).

7-(4-(Trifluoromethyl)benzyl)-6,7-dihydro-5*H*-pyrrolo[3,4-*b*]pyrazin-5-one (*rac*-2c)

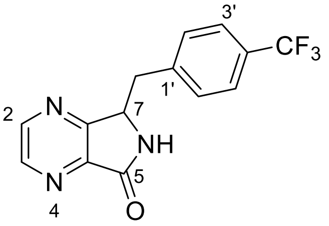

rac-**2c**
$C_{14}H_{10}F_3N_3O$
M = 293.25 g mol^{-1}

According to GP A, a 1 M solution of KHMDS (1.08 mL, 216 mg, 1.08 mmol, 1.10 equiv.) was added dropwise to a suspension of 6-(4-methoxybenzyl) 6,7-dihydro-5*H*-pyrrolo[3,4-*b*]pyrazin-5-one **SI-9** (250 mg, 979 µmol, 1.00 equiv.) in anhydrous THF (30 mL) at –78 °C. After 20 min, 1-(bromomethyl)-4-(trifluoromethyl)benzene (196 µL, 304 mg, 1.27 mmol, 1.30 equiv.) was added in one portion. The reaction was left to stir for 4 h at –78 °C. Afterwards, the solution was allowed to warm up to room temperature and quenched by the addition of sat. NH_4Cl (6 mL) and distilled water (20 mL). The aqueous layer was extracted with CH_2Cl_2 (3 × 25 mL), and the combined organic phases were dried over Na_2SO_4, filtered and concentrated under reduced pressure. The crude product was then dissolved in MeCN:H_2O (3:1) (29 mL) and diammonium cerium(IV) nitrate (1.61 g, 2.94 mmol, 3.00 equiv.) was added to the solution at room temperature. The yellow solution was stirred for 90 min at the same temperature. Subsequently, water (30 mL) was added, and the mixture was extracted with CH_2Cl_2 (3 × 50 mL). The combined organic layers were washed with brine (50 mL) and dried over Na_2SO_4. After filtration, the solvent was removed under reduced pressure and the residual crude product was subjected to FCC (SiO_2, EtOAc) to yield the desired racemic substrate *rac*-**2c** (25 mg, 85.3 µmol, 9%) as a white solid.

TLC (EtOAc): R_f = 0.21 [UV] [$KMnO_4$].

M.p.: 209 °C.

^{1}H-NMR (400 MHz, $CDCl_3$, 300 K): δ [ppm] = 8.79 (d, ^3J = 2.5 Hz, 1H, H2), 8.74 (d, ^3J = 2.6 Hz, 1H, H3), 7.53 (d, ^3J = 8.0 Hz, 2H, H3', H5'), 7.50 (bs, 1H, NH), 7.30 (d, ^3J = 8.0 Hz, 2H, H2', H6'), 4.97 (dd, ^3J = 8.0 Hz, 4.1 Hz, 1H, H7), 3.64 (dd, ^2J = 13.9 Hz, ^3J = 4.1 Hz, 1H, C7-CH_a-C1'), 2.96 (dd, ^2J = 13.9, ^3J = 8.0 Hz, 1H, C7-CH_b-C1').

^{13}C-NMR (101 MHz, $CDCl_3$, 300 K)**:** δ [ppm] = 166.7 (C5), 159.8 (C7a), 146.8 (C3), 146.4 (C2), 144.6 (C4a), 139.5 (C1'), 130.0 (C2'/C6'), 129.7 (q, $^2J_{CF}$ = 32.5 Hz, C4'), 125.8 (q, $^3J_{CF}$ = 3.8 Hz, C3'/C5'), 124.1 (q, $^1J_{CF}$ = 272.2 Hz, CF_3), 56.95 (C7), 38.57 (C7-*C*H_2-C1').

^{19}F-NMR (126 MHz, $CDCl_3$, 300 K)**:** δ [ppm] = –62.62 (CF_3).

HRMS (ESI) m/z**:** Calculated for $[M+H]^+$: 294.0849; found: 294.0847.

IR (film) $\tilde{\nu}_{max}$/cm^{-1}**:** 3227 (m, NH), 3136 (m, CH_{arom}), 2925 (m, CH_{aliph}), 1709 (s, C=O), 1376 (m, $C{=}C_{arom}$), 1323 (s, CF_{aliph}), 1157 (m, CN), 829 (m, CH_{arom}).

7-(4-Bromobenzyl)-6,7-dihydro-5*H*-pyrrolo[3,4-*b*]pyrazin-5-one (*rac*-2d)

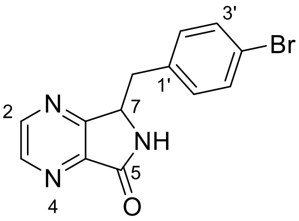

rac-**2d**

$C_{13}H_{10}BrN_3O$

M = 304.15 g mol^{-1}

According to GP A, a 1 M solution of KHMDS (1.08 mL, 216 mg, 1.08 mmol, 1.10 equiv.) was added dropwise to a suspension of 6-(4-methoxybenzyl) 6,7-dihydro-5*H*-pyrrolo[3,4-*b*]pyrazin-5-one **SI-9** (250 mg, 979 µmol, 1.00 equiv.) in anhydrous THF (30 mL) at –78 °C. After 20 min, 1-bromo-4-(bromomethyl)benzene (318 mg, 1.27 mmol, 1.30 equiv.) was added in one portion. The reaction was left to stir for 4 h at –78 °C. Afterwards, the solution was allowed to warm up to room temperature and quenched by the addition of sat. NH_4Cl (6 mL) and distilled water (20 mL). The aqueous layer was extracted with CH_2Cl_2 (3 × 25 mL), and the combined organic phases were dried over Na_2SO_4, filtered and concentrated under reduced pressure. The crude product was then dissolved in MeCN:H_2O (3:1) (29 mL) and diammonium cerium(IV) nitrate (1.61 g, 2.94 mmol, 3.00 equiv.) was added to the solution at room temperature. The yellow solution was stirred for 90 min at the same temperature. Subsequently, water (30 mL)

was added, and the mixture was extracted with CH_2Cl_2 (3 × 50 mL). The combined organic layers were washed with brine (50 mL) and dried over Na_2SO_4. After filtration, the solvent was removed under reduced pressure and the residual crude product was subjected to FCC (SiO_2, EtOAc) to yield the desired racemic substrate *rac*-**2d** (101 mg, 332 µmol, 34%) as a white solid**.**

TLC (EtOAc): R_f = 0.14 [UV] [$KMnO_4$].

M.p.: 212 °C.

^{1}H-NMR (400 MHz, $CDCl_3$, 300 K): δ [ppm] = 8.79 (d, ^3J = 2.6 Hz, 1H, H2), 8.73 (d, ^3J = 2.6 Hz, 1H, H3), 7.42 – 7.37 (m, 2H, H2', H6'), 7.24 (bs, 1H, NH), 7.07 – 7.02 (m, 2H, H3', H5'), 4.93 (dd, ^2J = 8.2 Hz, ^3J = 4.0 Hz, 1H, H7), 3.60 (dd, ^2J = 14.0 Hz, ^3J = 4.0 Hz, 1H, C7-CH_a-C1'), 2.72 (dd, J = 14.0 Hz, 8.3 Hz, 1H, C7-CH_b-C1').

^{13}C-NMR (101 MHz, $CDCl_3$, 300 K): δ [ppm] = 166.8 (C5), 160.0 (C7a), 150.6 (C4'), 146.8 (C3), 146.3 (C2), 144.7 (C4a), 134.3 (C1'), 132.0 (C3', C5'), 131.3 (C2', C6'), 121.6 (C4'), 57.1 (C7), 38.2 (C3-CH$_2$-C1').

HRMS (ESI) m/z: Calculated for $[M+H]^+$: 304.0080; found: 304.0085.

IR (film) $\tilde{\nu}_{max}$/cm^{-1}**:** 3081 (m, NH), 3002 (m, CH_{arom}), 2791 (m, CH_{aliph}), 1712 (s, C=O), 1460 (m, $C{=}C_{arom}$), 1368 (m, $C{=}C_{arom}$), 1138 (m, CN), 1072 (s, CBr_{arom}), 847 (m, CH_{arom}).

7-(2-Chlorobenzyl)-6,7-dihydro-5*H*-pyrrolo[3,4-*b*]pyrazin-5-one (*rac*-2e)

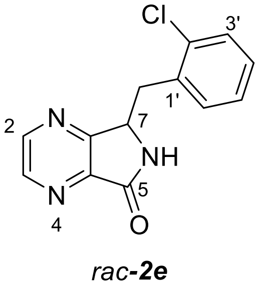

rac-**2e**
$C_{13}H_{10}ClN_3O$
M = 259.69 g mol^{-1}

According to GP A, a 1 M solution of KHMDS (1.08 mL, 216 mg, 1.08 mmol, 1.10 equiv.) was added dropwise to a suspension of 6-(4-methoxybenzyl) 6,7-dihydro-5*H*-pyrrolo[3,4-*b*]pyrazin-5-one **SI-9** (250 mg, 979 µmol, 1.00 equiv.) in anhydrous THF (30 mL) at –78 °C. After 20 min, 1-(bromomethyl)-2-chlorobenzene (166 µL, 261 mg, 1.27 mmol, 1.30 equiv.) was added in one portion. The reaction was left to stir for 4 h at –78 °C. Afterwards, the solution was allowed to warm up to room temperature and quenched by the addition of sat. NH_4Cl (6 mL) and distilled water (20 mL). The aqueous layer was extracted with CH_2Cl_2 (3 × 25 mL), and the combined organic phases were dried over Na_2SO_4, filtered and concentrated under

reduced pressure. The crude product was then dissolved in MeCN:H_2O (3:1) (29 mL) and diammonium cerium(IV) nitrate (1.61 g, 2.94 mmol, 3.00 equiv.) was added to the solution at room temperature. The yellow solution was stirred for 90 min at the same temperature. Subsequently, water (30 mL) was added, and the mixture was extracted with CH_2Cl_2 (3 × 50 mL). The combined organic layers were washed with brine (50 mL) and dried over Na_2SO_4. After filtration, the solvent was removed under reduced pressure and the residual crude product was subjected to FCC (SiO_2, EtOAc) to yield the desired racemic substrate *rac*-**2e** (58 mg, 223 µmol, 23%) as a white solid**.**

TLC (EtOAc): R_f = 0.24 [UV] [$KMnO_4$].

M.p.: 187 °C.

^{1}H-NMR (400 MHz, $CDCl_3$, 300 K): δ [ppm] = 8.80 (d, ^3J = 2.6 Hz, 1H, H2), 8.74 (d, ^3J = 2.6 Hz, 1H, H3), 7.42 (dd, ^3J = 7.7 Hz, ^4J = 1.5 Hz, 1H, H3'), 7.24 (*virt.* dt, $^3J \approx {}^3J \approx$ 7.5 Hz, ^4J = 2.0 Hz, 1H, H4'), 7.20 (*virt.* dt, $^3J \approx {}^3J \approx$ 7.5 Hz, ^4J = 2.0 Hz, 1H, H5'), 7.15 (dd, ^3J = 7.5 Hz, ^4J = 2.0 Hz, 1H, H6'), 5.05 (dd, ^3J = 9.1 Hz, ^3J = 4.1 Hz, 1H, H7), 3.84 (dd, ^2J = 13.9 Hz, ^3J = 4.1 Hz, 1H, C7-CH_a-C1'), 2.87 (dd, ^2J = 13.9 Hz, ^3J = 9.1 Hz, 1H, C7-CH_b-C1').

^{13}C-NMR (101 MHz, $CDCl_3$, 300 K): δ [ppm] = 166.1 (C5), 160.1 (C7a), 146.8 (C3), 146.4 (C2), 144.4 (C4a), 134.6 (C2'), 133.8 (C1'), 131.7 (C6'), 130.3 (C3'), 129.3 (C4'), 127.4 (C5'), 55.8 (C7), 37.1 (C3-CH_2-C1').

HRMS (ESI) m/z: Calculated for $[M+H]^+$: 260.0585; found: 260.0584.

IR (film) $\tilde{\nu}_{max}/cm^{-1}$**:** 3219 (m, NH), 3089 (m, CH_{arom}), 1723 (s, C=O), 1379 (m, $C{=}C_{arom}$), 1156 (m, CN), 1051 (s, CCl_{arom}), 747 (m, CH_{arom}).

7-(4-(Trifluoromethoxy)benzyl)-6,7-dihydro-5*H*-pyrrolo[3,4-*b*]pyrazin-5-one (*rac*-2f)

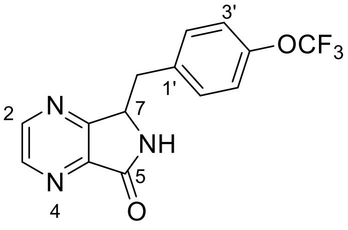

rac-**2f**
$C_{14}H_{10}F_3N_3O_2$
M = 309.25 g mol^{-1}

According to GP A, a 1 M solution of KHMDS (1.08 mL, 216 mg, 1.08 mmol, 1.10 equiv.) was added dropwise to a suspension of 6-(4-methoxybenzyl) 6,7-dihydro-5*H*-pyrrolo[3,4-*b*]pyrazin-5-one **SI-9** (250 mg, 979 µmol, 1.00 equiv.) in anhydrous THF (30 mL) at –78 °C. After 20 min, 1-(bromomethyl)-4-(trifluoromethoxy)benzene (204 µL, 324 mg, 1.27 mmol, 1.30 equiv.) was added in one portion. The reaction was left to stir for 4 h at –78 °C. Afterwards, the solution was allowed to warm up to room temperature and quenched by the addition of sat. NH_4Cl (6 mL) and distilled water (20 mL). The aqueous layer was extracted with CH_2Cl_2 (3 × 25 mL), and the combined organic phases were dried over Na_2SO_4, filtered and concentrated under reduced pressure. The crude product was then dissolved in MeCN:H_2O (3:1) (29 mL) and diammonium cerium(IV) nitrate (1.61 g, 2.94 mmol, 3.00 equiv.) was added to the solution at room temperature. The yellow solution was stirred for 90 min at the same temperature. Subsequently, water (30 mL) was added, and the mixture was extracted with CH_2Cl_2 (3 × 50 mL). The combined organic layers were washed with brine (50 mL) and dried over Na_2SO_4. After filtration, the solvent was removed under reduced pressure and the residual crude product was subjected to FCC (SiO_2, EtOAc) to yield the desired racemic substrate *rac*-**2f** (58 mg, 186 µmol, 19%) as a white solid**.**

TLC (EtOAc): R_f = 0.15 [UV] [$KMnO_4$].

M.p.: 194 °C.

^{1}H-NMR (400 MHz, $CDCl_3$, 300 K): δ [ppm] = 8.79 (d, ^3J = 2.6 Hz, 1H, H2), 8.74 (d, ^3J = 2.6 Hz, 1H, H3), 7.34 (bs, 1H, NH), 7.24 – 7.18 (m, 2H, H2', H6'), 7.15 – 7.10 (m, 2H, H3', H5'), 4.98 (dd, ^2J = 8.5 Hz, ^3J = 4.0 Hz, 1H, H7), 3.60 (dd, ^2J = 14.0 Hz, ^3J = 4.0 Hz, 1H, C7-CH_a-C1'), 2.72 (dd, J = 14.0 Hz, 8.5 Hz, 1H, C7-CH_b-C1').

^{13}C-NMR (101 MHz, $CDCl_3$, 300 K)**:** δ [ppm] = 166.5 (C5), 159.9 (C7a), 148.7 (C4'), 146.8 (C3), 146.4 (C2), 144.6 (C4a), 134.4 (C1'), 130.9 (C2', C6'), 121.5 (C3', C5'), 120.6 (q, $^1J_{CF}$ = 257.5.2 Hz, OCF_3), 57.2 (C7), 38.4 (C7-$C$$H_2$-C1').

^{19}F-NMR (126 MHz, $CDCl_3$, 300 K)**:** δ [ppm] = -57.9 (CF_3).

HRMS (ESI) m/z**:** Calculated for $[M+H]^+$: 310.0798; found: 310.0807.

IR (film) $\tilde{\nu}_{max}$/cm^{-1}**:** 3310 (m, NH), 2914 (m, CH_{aliph}), 1705 (s, C=O), 1508 (m, $C{=}C_{arom}$), 1147 (s, CF_{aliph}), 1124 (m, CN), 863 (m, CH_{arom}).

7-(3,5-Dimethylbenzyl)-6,7-dihydro-5*H*-pyrrolo[3,4-*b*]pyrazin-5-one (*rac*-2g)

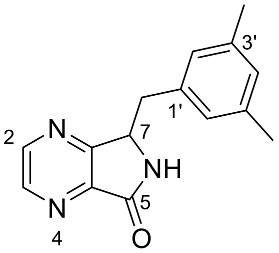

rac-**2g**
$C_{15}H_{15}N_3O$
M = 253.31 g mol^{-1}

According to GP A, a 1 M solution of KHMDS (1.08 mL, 216 mg, 1.08 mmol, 1.10 equiv.) was added dropwise to a suspension of 6-(4-methoxybenzyl) 6,7-dihydro-5*H*-pyrrolo[3,4-*b*]pyrazin-5-one **SI-9** (250 mg, 979 µmol, 1.00 equiv.) in anhydrous THF (30 mL) at –78 °C. After 20 min, 1-(bromomethyl)-3,5-dimethylbenzene (206µL, 254 mg, 1.27 mmol, 1.30 equiv.) was added in one portion. The reaction was left to stir for 4 h at –78 °C. Afterwards, the solution was allowed to warm up to room temperature and quenched by the addition of sat. NH_4Cl (6 mL) and distilled water (20 mL). The aqueous layer was extracted with CH_2Cl_2 (3 × 25 mL), and the combined organic phases were dried over Na_2SO_4, filtered and concentrated under reduced pressure. The crude product was then dissolved in MeCN:H_2O (3:1) (29 mL) and diammonium cerium(IV) nitrate (1.61 g, 2.94 mmol, 3.00 equiv.) was added to the solution at room temperature. The yellow solution was stirred for 90 min at the same temperature. Subsequently, water (30 mL) was added, and the mixture was extracted with CH_2Cl_2 (3 × 50 mL). The combined organic layers were washed with brine (50 mL) and dried over Na_2SO_4. After filtration, the solvent was removed under reduced pressure and the residual crude product was subjected to FCC (SiO_2, EtOAc) to yield the desired racemic substrate *rac*-**2g** (87 mg, 344 µmol, 35%) as a white solid.

TLC (EtOAc): R_f = 0.26 [UV] [$KMnO_4$].

M.p.: 213 °C.

^{1}H-NMR (400 MHz, $CDCl_3$, 300 K): δ [ppm] = 8.79 (d, ^3J = 2.6 Hz, 1H, H2), 8.72 (d, ^3J = 2.6 Hz, 1H, H3), 6.97 (s, 1H, NH), 6.90 (s, 1H, H4’), 6.83 (s, 2H, H2’, H6’), 4.85 (dd,

^2J = 9.9 Hz, ^3J = 3.7 Hz, 1H, H7), 3.55 (dd, ^2J = 13.6 Hz, ^3J = 3.7 Hz, 1H, C7-CH_a-C1'), 2.62 (dd, J = 13.6 Hz, 9.9 Hz, 1H, C7-CH_b-C1'), 2.28 (s, 6H, 2 CH_3).

^{13}C-NMR (101 MHz, $CDCl_3$, 300 K): δ [ppm] = 166.2 (C5), 160.3 (C7a), 146.7 (C3), 146.3 (C2), 144.6 (C4a), 138.8 (C3', C5'), 136.0 (C1'), 129.2 (C4'), 127.1 (C2', C6'), 57.7 (C7), 39.3 (C3-*C*H_2-C1'), 21.4 (CH_3).

HRMS (ESI) m/z: Calculated for $[M+H]^+$: 254.1293; found: 254.1306.

IR (film): $\tilde{\nu}$max/cm^{-1} = 3213 (m, NH), 3020 (m, CH_{arom}), 2929 (m, CH_{aliph}), 1705 (s, C=O), 1605 (m, $C=C_{arom}$), 1146 (m, CN), 732 (m, CH_{arom}).

***tert*-Butyl 4-((7-oxo-6,7-dihydro-5*H*-pyrrolo[3,4-*b*]pyrazin-5-yl)methyl)benzoate (*rac*-2h)**

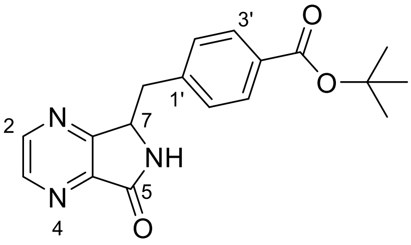

rac-**2h**
$C_{18}H_{19}N_3O_3$
M = 325.37 g mol^{-1}

According to GP A, a 1 M solution of KHMDS (1.08 mL, 216 mg, 1.08 mmol, 1.10 equiv.) was added dropwise to a suspension of 6-(4-methoxybenzyl) 6,7-dihydro-5*H*-pyrrolo[3,4-*b*]pyrazin-5-one **SI-9** (250 mg, 979 µmol, 1.00 equiv.) in anhydrous THF (30 mL) at –78 °C. After 20 min, tert-butyl 4-(bromomethyl)benzoate (345 mg, 1.27 mmol, 1.30 equiv.) was added in one portion. The reaction was left to stir for 4 h at –78 °C. Afterwards, the solution was allowed to warm up to room temperature and quenched by the addition of sat. NH_4Cl (6 mL) and distilled water (20 mL). The aqueous layer was extracted with CH_2Cl_2 (3 × 25 mL), and the combined organic phases were dried over Na_2SO_4, filtered and concentrated under reduced pressure. The crude product was then dissolved in MeCN:H_2O (3:1) (29 mL) and diammonium cerium(IV) nitrate (1.61 g, 2.94 mmol, 3.00 equiv.) was added to the solution at room temperature. The yellow solution was stirred for 90 min at the same temperature. Subsequently, water (30 mL) was added, and the mixture was extracted with CH_2Cl_2 (3 × 50 mL). The combined organic layers were washed with brine (50 mL) and dried over Na_2SO_4. After filtration, the solvent was removed under reduced pressure and the residual crude product was subjected to FCC (SiO_2, EtOAc) to yield the desired racemic substrate *rac*-**2h** (45 mg, 138 µmol, 14%) as a white solid.

TLC (EtOAc): R_f = 0.19 [UV] [$KMnO_4$].

M.p.: 219 °C.

^{1}H-NMR (400 MHz, $CDCl_3$, 300 K): δ [ppm] = 8.78 (d, ^{3}J = 2.6 Hz, 1H, H2), 8.73 (d, ^{3}J = 2.6 Hz, 1H, H3), 7.90 – 7.86 (m, 2H, H3’, H5’), 7.23 – 7.18 (m, 2H, H2’, H6’), 7.26* (bs, 1H, NH), 4.95 (dd, ^{2}J =8.2 Hz, ^{3}J = 3.9 Hz, 1H, H7), 3.60 (dd, ^{2}J = 13.8 Hz, ^{3}J = 3.9 Hz, 1H, C7-CH_a-C1’), 2.99 (dd, J = 13.8 Hz, 8.2 Hz, 1H, C7-CH_b-C1’), 1.58 [s, 9H, $C(CH_3)_3$].
*The Signal is overlapping with the solvent signal.

^{13}C-NMR (101 MHz, $CDCl_3$, 300 K): δ [ppm] = 166.1 (C5), 165.4 (COO), 159.9 (C7a), 146.7 (C3), 146.4 (C2), 144.6 (C4a), 140.2 (C-1’), 131.4 (C4’), 130.1 (C3’, C5’), 129.4 (C2’, C6’), 81.3 [O*C*$(CH_3)_3$] 57.1 (C7), 38.9 (C3-*C*H_2-C1’), 28.3 [C(*C*$H_3)_3$].

HRMS (ESI) m/z: Calculated for $[M+H]^+$: 326.1499; found: 326.1497.

IR (film): $\tilde{v}$max/cm^{-1} = 3172 (m, NH), 3091 (m, CH_{arom}), 2975 (m, CH_{aliph}), 1709 (s, C=O), 1610 (m, $C{=}C_{arom}$), 1291 (m, CN), 750 (m, CH_{arom}).

7-((5-(Trifluoromethyl)furan-2-yl)methyl)-6,7-dihydro-5*H*-pyrrolo[3,4-*b*]pyrazin-5-one (*rac*-2i)

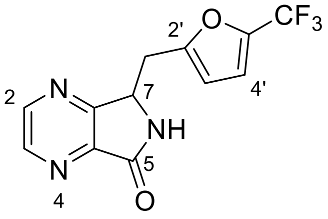

rac-**2i**
$C_{12}H_8F_3N_3O_2$
M = 283.21 g mol^{-1}

According to GP A, a 1 M solution of KHMDS (1.08 mL, 216 mg, 1.08 mmol, 1.10 equiv.) was added dropwise to a suspension of 6-(4-methoxybenzyl) 6,7-dihydro-5*H*-pyrrolo[3,4-*b*]pyrazin-5-one **SI-9** (250 mg, 979 µmol, 1.00 equiv.) in anhydrous THF (30 mL) at –78 °C. After 20 min, 2-(bromomethyl)-5-(trifluoromethyl)furan (292 mg, 1.27 mmol, 1.30 equiv.) was added in one portion. The reaction was left to stir for 4 h at –78 °C. Afterwards, the solution was allowed to warm up to room temperature and quenched by the addition of sat. NH_4Cl (6 mL) and distilled water (20 mL). The aqueous layer was extracted with CH_2Cl_2 (3 × 25 mL), and the combined organic phases were dried over Na_2SO_4, filtered and concentrated under reduced pressure. The crude product was then dissolved in MeCN:H_2O (3:1) (29 mL) and diammonium cerium(IV) nitrate (1.61 g, 2.94 mmol, 3.00 equiv.) was added to the solution at room temperature. The yellow solution was stirred for 90 min at the same temperature. Subsequently, water (30 mL) was added, and the mixture was extracted with CH_2Cl_2

(3 × 50 mL). The combined organic layers were washed with brine (50 mL) and dried over Na_2SO_4. After filtration, the solvent was removed under reduced pressure and the residual crude product was subjected to FCC (SiO_2, EtOAc) to yield the desired racemic substrate *rac*-**2i** (55 mg, 194 µmol, 20%) as a white solid**.**

TLC (EtOAc): R_f = 0.41 [UV] [$KMnO_4$].

M.p.: 167 °C.

^{1}H-NMR (400 MHz, $CDCl_3$, 300 K): δ [ppm] = 8.80 (d, ^3J = 2.6 Hz, 1H, H2), 8.73 (d, ^3J = 2.6 Hz, 1H, H3), 8.06 (bs, 1H, NH), 6.65 (dq, ^3J =2.4 Hz, $^4J_{CF}$ = 1.2 Hz, 1H, H4’), 6.20 (d, ^3J =2.4 Hz, 1H, H3’), 5.02 (dd, ^2J =7.7 Hz, ^3J = 4.1 Hz, 1H, H7), 3.60 (dd, ^2J = 15.4 Hz, ^3J = 4.1 Hz, 1H, C7-CH_a-C1’), 3.15 (dd, J = 15.4, 7.7 Hz, 1H, C7-CH_b-C1’).

^{13}C-NMR (101 MHz, $CDCl_3$, 300 K): δ [ppm] = 166.9 (C5), 159.7 (C7a), 153.0 (C2’), 146.9 (C3), 146.5 (C-2), 144.4 (C4a), 141.6 (q, $^2J_{CF}$ = 42.9 Hz, C5’), 118.9 (q, $^1J_{CF}$ = 226.8 Hz, CF_3), 112.6 (q, $^3J_{CF}$ = 2.8 Hz, C4’), 109.4 (C3’), 54.9 (C7), 31.5 (C3-CH_2-C1’).

HRMS (ESI) m/z: Calculated for $[M+H]^+$: 284.0641; found: 286.0640.

IR (film): $\tilde{\nu}$max/cm^{-1} = 3193 (m, NH), 3098 (m, CH_{arom}), 1726 (s, C=O), 1560 (m, $C=C_{arom}$), 1322 (m, CN), 750 (m, CH_{arom}).

7-Ethyl-6,7-dihydro-5*H*-pyrrolo[3,4-*b*]pyrazin-5-one (*rac*-2j)

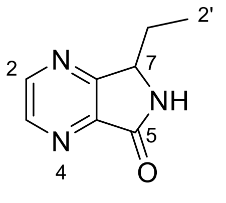

rac-**2j**
$C_8H_9N_3O$
M = 163.18 g mol^{-1}

According to GP B, to solution of of 6-(4-methoxybenzyl) 6,7-dihydro-5*H*-pyrrolo[3,4-*b*]pyrazin-5-one **SI-9** (250 mg, 979 µmol, 1.00 equiv.) in anhydrous DMF (5 mL) was added Cs_2CO_3 (479 mg, 1.47 mmol, 1.50 equiv.) at room temperature. The resulting mixture was stirred 5 min after which iodoethane (102 µL, 199 mg, 1.27 mmol, 1.30 equiv.) was added. The reaction was stirred for 16 h at the same temperature. Afterwards, the reaction was quenched by the addition of sat. NH_4Cl (2 mL) and distilled water (10 mL). The aqueous layer was extracted with CH_2Cl_2 (3 × 25 mL), and the combined organic layers were washed with sat. LiCl solution (25 mL), brine (25 mL) and dried over Na_2SO_4. After filtration, the solvent was removed under reduced pressure. The

crude product was then dissolved in MeCN:H_2O (3:1) (29 mL) and diammonium cerium(IV) nitrate (1.61 g, 2.94 mmol, 3.00 equiv.) was added to the solution at room temperature. The yellow solution was stirred for 90 min at the same temperature. Subsequently, water (30 mL) was added, and the mixture was extracted with CH_2Cl_2 (3 × 50 mL). The combined organic layers were washed with brine (50 mL) and dried over Na_2SO_4. After filtration, the solvent was removed under reduced pressure and the residual crude product was subjected to FCC (SiO_2, EtOAc) to yield the desired racemic substrate *rac*-**2j** (42 mg, 257 µmol, 26%) as a white solid**.**

TLC (EtOAc): R_f = 0.14 [UV] [$KMnO_4$].

M.p.: 135°C.

^{1}H-NMR (400 MHz, $CDCl_3$, 300 K): δ [ppm] = 8.78 (d, ^3J = 2.6 Hz, 1H, H2), 8.70 (d, ^3J = 2.6 Hz, 1H, H3), 8.14 (bs, 1H, NH), 4.69 (dd, ^2J =7.8 Hz, ^3J = 4.2 Hz, 1H, H7), 2.18 (ddt, $^2J \approx {}^3J \approx$ 7.3 Hz, ^3J = 4.2 Hz, 1H, H_a1'), 1.87 (m, 1H, H_b2') 1.03 (t, ^3J = 7.4 Hz, 1H, H2').

^{13}C-NMR (101 MHz, $CDCl_3$, 300 K): δ [ppm] = 167.5 (C5), 161.0 (C7a), 146.7 (C3), 146.0 (C2), 144.6 (C4a), 57.6 (C7), 25.9 (C1'), 9.6 (C2').

HRMS (ESI) m/z: Calculated for $[M+H]^+$: 164.0818; found: 164.0819.

IR (film): $\tilde{\nu}$max/cm^{-1} = 3192 (m, NH), 3095 (m, CH_{arom}), 2967 (m, CH_{aliph}), 2877 (m, CH_{aliph}), 1710 (s, C=O), 1365 (m, CN).

7-Isopentyl-6,7-dihydro-5*H*-pyrrolo[3,4-*b*]pyrazin-5-one (*rac*-2k)

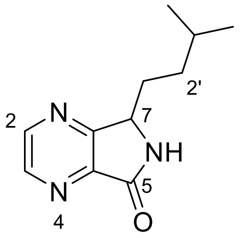

rac-**2k**

$C_{11}H_{15}N_3O$

M = 205.26 g mol^{-1}

According to GP B, to solution of of 6-(4-methoxybenzyl) 6,7-dihydro-5*H*-pyrrolo[3,4-*b*]pyrazin-5-one **SI-9** (250 mg, 979 µmol, 1.00 equiv.) in anhydrous DMF (5 mL) was added Cs_2CO_3 (479 mg, 1.47 mmol, 1.50 equiv.) at room temperature. The resulting mixture was stirred 5 min after which 1-iodo-3-methylbutane (168 µL, 252 mg, 1.27 mmol, 1.30 equiv.) was added. The reaction was stirred for 16 h at the same temperature. Afterwards, the reaction was quenched by the addition of sat. NH_4Cl (2 mL) and distilled water (10 mL). The aqueous layer was extracted with CH_2Cl_2 (3 × 25 mL) and the combined organic layers were washed with sat. LiCl

solution (25 mL), brine (25 mL) and dried over Na_2SO_4. After filtration, the solvent was removed under reduced pressure. The crude product was then dissolved in MeCN:H_2O (3:1) (29 mL) and diammonium cerium(IV) nitrate (1.61 g, 2.94 mmol, 3.00 equiv.) was added to the solution at room temperature. The yellow solution was stirred for 90 min at the same temperature. Subsequently, water (30 mL) was added, and the mixture was extracted with CH_2Cl_2 (3 × 50 mL). The combined organic layers were washed with brine (50 mL) and dried over Na_2SO_4. After filtration, the solvent was removed under reduced pressure and the residual crude product was subjected to FCC (SiO_2, EtOAc) to yield the desired racemic substrate *rac*-**2k** (34 mg, 166 µmol, 17%) as a white solid**.**

TLC (EtOAc): R_f = 0.26 [UV] [$KMnO_4$].

M.p.: 133°C.

^{1}H-NMR (400 MHz, $CDCl_3$, 300 K): δ [ppm] = 8.78 (d, ^3J = 2.6 Hz, 1H, H2), 8.69 (d, ^3J = 2.6 Hz, 1H, H3), 8.09 (bs, 1H, NH), 4.69 (dd, ^2J =7.5 Hz, ^3J = 4.9 Hz, 1H, H7), 2.12 (m, 1H, H_a1'), 1.79 (m, 1H, H_b2'), 1.58 (m, 1H, H3'), 1.46 (m, 1H, H_a2'), 1.22 (m, 1H, H_b2'), 0.90 (d, ^3J = 6.6 Hz, 3H, H_a4'), 0.88 (d, ^3J = 6.6 Hz, 3H, H_b4').

^{13}C-NMR (101 MHz, $CDCl_3$, 300 K): δ [ppm] = 167.3 (C5), 161.2 (C7a), 146.7 (C3), 146.0 (C2), 144.5 (C4a), 56.7 (C7), 34.3 (C2'), 30.8 (C1'), 28.14 (C3'), 22.6 (C_a4'), 22.4 (C_b4').

HRMS (ESI) m/z: Calculated for $[M+H]^+$: 206.1288; found: 206.1288.

IR (film): $\tilde{v}$ max/cm^{-1} = 3179 (m, NH), 3097 (m, CH_{arom}), 2957 (m, CH_{aliph}), 2873 (m, CH_{aliph}), 1706 (s, C=O), 1376 (m, CN).

7-(Cyclopentylmethyl)-6,7-dihydro-5*H*-pyrrolo[3,4-*b*]pyrazin-5-one (*rac*-2l)

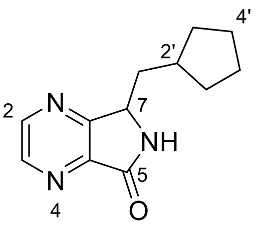

rac-**2l**
$C_{12}H_{15}N_3O$
M = 217.27 g mol^{-1}

According to GP B, to solution of of 6-(4-methoxybenzyl) 6,7-dihydro-5*H*-pyrrolo[3,4-*b*]pyrazin-5-one **SI-9** (250 mg, 979 µmol, 1.00 equiv.) in anhydrous DMF (5 mL) was added Cs_2CO_3 (479 mg, 1.47 mmol, 1.50 equiv.) at room temperature. The resulting mixture was stirred 5 min after which (iodomethyl)cyclopentane (167 µL, 267 mg, 1.27 mmol, 1.30 equiv.) was added. The reaction was stirred for 16 h at the same temperature. Afterwards, the reaction was quenched by the addition of sat. NH_4Cl (2 mL) and distilled water (10 mL). The aqueous layer was extracted with CH_2Cl_2 (3 × 25 mL), and the combined organic layers were washed with sat. LiCl solution (25 mL), brine (25 mL) and dried over Na_2SO_4. After filtration, the solvent was removed under reduced pressure. The crude product was then dissolved in MeCN:H_2O (3:1) (29 mL) and diammonium cerium(IV) nitrate (1.61 g, 2.94 mmol, 3.00 equiv.) was added to the solution at room temperature. The yellow solution was stirred for 90 min at the same temperature. Subsequently, water (30 mL) was added, and the mixture was extracted with CH_2Cl_2 (3 × 50 mL). The combined organic layers were washed with brine (50 mL) and dried over Na_2SO_4. After filtration, the solvent was removed under reduced pressure and the residual crude product was subjected to FCC (SiO_2, EtOAc) to yield the desired racemic substrate *rac*-**2l** (76 mg, 350 µmol, 36%) as a white solid**.**

TLC (EtOAc): R_f = 0.31 [UV] [$KMnO_4$].

M.p.: 159 °C.

^{1}H-NMR (400 MHz, $CDCl_3$, 300 K): δ [ppm] = 8.71 (d, ^3J = 2.6 Hz, 1H, H2), 8.62 (d, ^3J = 2.6 Hz, 1H, H3), 7.86 (bs, 1H, NH), 4.66 (dd, ^2J = 8.4 Hz, ^3J = 4.5 Hz, 1H, H7), 2.03 (m, 2H, H_a1'/H2'), 1.84 (m, 1H, H_a3'/H6$_a$'), 1.59 (m, 6H, H_a4', H_b4', H_a5', H_b5', H_b1', H_a3'/H_a6'), 1.19 (m, 1H, H_b3'/H_b6'), 1.06 (m, 1H, H_b6'/H_b3').

^{13}C-NMR (101 MHz, $CDCl_3$, 300 K): δ [ppm] = 170.0 (C5), 161.4 (C7a), 146.6 (C3), 146.0 (C2), 144.4 (C4a), 56.2 (C7), 39.3 (C1'), 37.1 (C2'), 33.4 (C3'/C6'), 32.8 (C6'/C3'), 25.3 (C4'/C5'), 25.2 (C5'/C4').

HRMS (ESI) m/z: Calculated for $[M+H]^+$: 218.1288; found: 218.1288.

IR (film): $\tilde{\nu}$ max/cm^{-1} = 3188 (m, NH), 3080 (m, CH_{arom}), 2948 (m, CH_{aliph}), 2868 (m, CH_{aliph}), 1709 (s, C=O), 1377 (m, CN).

7-(Cyclopropylmethyl)-6,7-dihydro-5*H*-pyrrolo[3,4-*b*]pyrazin-5-one (*rac*-2m)

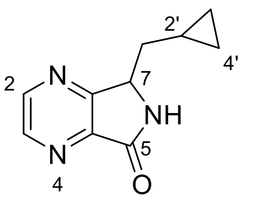

rac-**2m**
$C_{10}H_{11}N_3O$
M = 189.22 g mol^{-1}

According to GP B, to solution of of 6-(4-methoxybenzyl) 6,7-dihydro-5*H*-pyrrolo[3,4-*b*]pyrazin-5-one **SI-9** (250 mg, 979 µmol, 1.00 equiv.) in anhydrous DMF (5 mL) was added Cs_2CO_3 (479 mg, 1.47 mmol, 1.50 equiv.) at room temperature. The resulting mixture was stirred 5 min after which (iodomethyl)cyclopropan (119 µL, 231 mg, 1.27 mmol, 1.30 equiv.) was added. The reaction was stirred for 16 h at the same temperature. Afterwards, the reaction was quenched by the addition of sat. NH_4Cl (2 mL) and distilled water (10 mL). The aqueous layer was extracted with CH_2Cl_2 (3 × 25 mL), and the combined organic layers were washed with sat. LiCl solution (25 mL), brine (25 mL) and dried over Na_2SO_4. After filtration, the solvent was removed under reduced pressure. The crude product was then dissolved in MeCN:H_2O (3:1) (29 mL) and diammonium cerium(IV) nitrate (1.61 g, 2.94 mmol, 3.00 equiv.) was added to the solution at room temperature. The yellow solution was stirred for 90 min at the same temperature. Subsequently, water (30 mL) was added, and the mixture was extracted with CH_2Cl_2 (3 × 50 mL). The combined organic layers were washed with brine (50 mL) and dried over Na_2SO_4. After filtration, the solvent was removed under reduced pressure and the residual crude product was subjected to FCC (SiO_2, EtOAc) to yield the desired racemic substrate *rac*-**2m** (77 mg, 407 µmol, 42%) as a white solid**.**

TLC (EtOAc): R_f = 0.38 [UV] [$KMnO_4$].

M.p.: 126 °C.

^{1}H-NMR (400 MHz, $CDCl_3$, 300 K): δ [ppm] = 8.77 (d, ^3J = 2.6 Hz, 1H, H2), 7.69 (bs, 1H, NH), 8.58 (d, ^3J = 2.6 Hz, 1H, H3), 4.81 (dd, ^2J = 7.9 Hz, ^3J = 4.4 Hz, 1H, H7), 2.12 (ddd, ^2J = 14.2 Hz, ^3J = 6.8 Hz, ^2J = 4.4 Hz, 1H, H_a1'), 1.62 (m, 1H, H_b1'), 0.88 (m, 1H, H2'), 0.51 (m, 2H, H_a3', H_a4'), 0.18 (m, 1H, H_b3'), 0.08 (m, 1H, H_b4').

^{13}C-NMR (101 MHz, $CDCl_3$, 300 K): δ [ppm] = 167.4 (C5), 161.1 (C7a), 146.6 (C3), 146.0 (C2), 144.6 (C4a), 57.2 (C7), 37.8 (C1'), 7.3 (C2'), 5.2 (C3'/C4'), 4.2 (C4'/C3').

HRMS (ESI) m/z: Calculated for $[M+H]^+$: 190.0975; found: 190.0975.

IR (film): $\tilde{v}$ max/cm^{-1} = 3170 (m, NH), 3079 (m, CH_{arom}), 2917 (m, CH_{aliph}), 2870 (m, CH_{aliph}), 1710 (s, C=O), 1379 (m, CN).

Pyrazine-2,3-dicarboximide (SI-10)

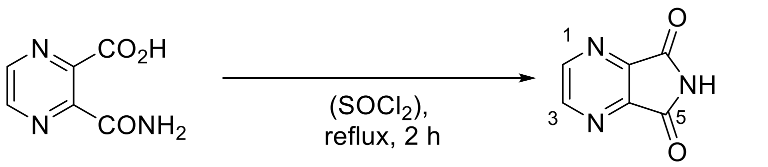

SI-10

$C_6H_3N_3O_2$

M = 149.11 g mol^{-1}

According to a modified procedure,[73] a mixture of 3-carbamoylpyrazine-2-carboxylic acid (1.00 g, 5.98 mmol, 1.00 equiv.) in thionyl chloride (10.0 mL, 16.4 g, 138 mmol, 23.0 equiv.) was refluxed for 2 h. After the mixture cooled to room temperature, the excess thionyl chloride was removed under reduced pressure. Then, the crude reaction mixture was directly subjected to FCC (SiO_2, acetone) to yield **SI-10** (892 mg, 5.98 mmol, *quant.*) as a white solid.

TLC (acetone): R_f = 0.63 [UV] [$KMnO_4$].

M.p.: >260 °C.

^{1}H-NMR (400 MHz, DMSO-d6, 300 K): δ [ppm] = 12.01 (s, 1H, NH), 9.00 (s, 2H, H2, H3).

^{13}C-NMR (101 MHz, DMSO-d6, 300 K): δ [ppm] = 165.7 (C4a, C7a), 148.6 (C2, C3).

HRMS (ESI) m/z: Calculated for $[M+H]^+$: 150.0298; found: 150.0299.

IR (film): $\tilde{v}$ max/cm^{-1} = 3162 (m, NH), 3077 (m, CH_{arom}), 1727 (s, C=O), 1347 (m, CN).

7-Hydroxy-6,7-dihydro-5*H*-pyrrolo[3,4-*b*]pyrazin-5-one (SI-11)

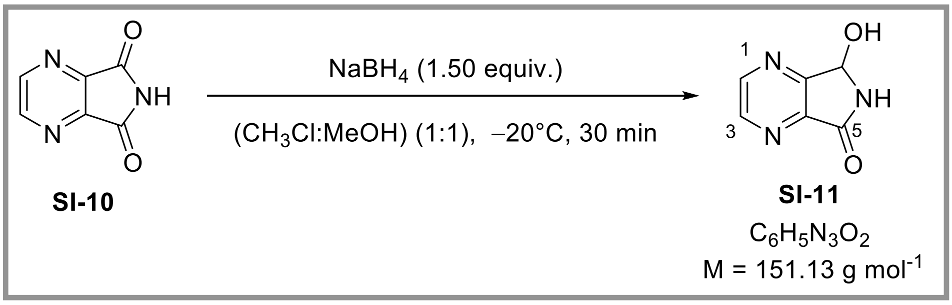

According to a modified procedure, [74] to a solution of **SI-10** (1.40 g, 9.39 mmol, 1.00 equiv.) in CH_3Cl:MeOH (1:1, 90 mL), was added sodium borohydride (533 mg, 14.1 mmol, 1.50 equiv.) portionwise at –20°C. The mixture was stirred for 30 min at the same temperature. Afterwards, the reaction was quenched by addition of 1 M HCl until the pH reached a value of approximately 2. The mixture was stirred for 10 min at this pH value at -20°C. Subsequently, 1 M NaOH was added until the pH reached a value of approximately 9. Subsequently, the solvents were removed under reduced pressure and the crude reaction mixture was directly subjected to FCC (SiO_2, CH_3Cl:MeOH = 9:1 → 5:1) to yield **SI-11** (1.14 g, 7.54 mmol, 80%.) as a white solid.

TLC (CH_3Cl:MeOH = 5:1): R_f = 0.41 [UV] [$KMnO_4$].

M.p.: 215°C (Decomposition).

^{1}H-NMR (400 MHz, DMSO-d6, 300 K): δ [ppm] = 9.56 (s, 1H, NH), 9.00 (m, 2H, H2, H3), 6.66 (d, ^3J = 9.2 Hz, 1H, OH), 5.91 (d, ^3J = 9.2 Hz, 1H, H7).

^{13}C-NMR (101 MHz, DMSO-d6, 300 K): δ [ppm] = 165.0 (C5), 159.6 (C7a), 146.9 (C3), 146.3 (C2), 144.5 (C4a), 76.4 (C7).

HRMS (ESI) m/z: Calculated for $[M+H]^+$: 152.0455; found: 152.0459.

IR (film): $\tilde{v}$ max/cm^{-1} = 3245 (s, OH), 3095 (m, CH_{arom}), 1692 (s, C=O), 1384 (m, CN).

7-(2-Chloroethoxy)-6,7-dihydro-5*H*-pyrrolo[3,4-*b*]pyrazin-5-one (*rac*-2n)

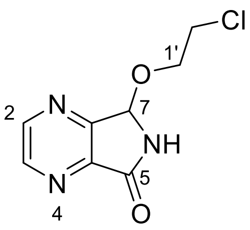

rac-**2n**
$C_8H_8ClN_3O_2$
M = 213.62 g mol^{-1}

According to GP C, to a suspension of **SI-11** (100 mg, 662 µmol 1.00 equiv.) in 2-chloroethan-1-ol (888 µL, 1.07 g, 13.2 mmol, 20.0 equiv.) was added *p*-TsOH (13.6 mg, 66.2 µmol, 0.10 equiv.). The resulting mixture was then stirred at 80 °C for 20 min. Afterwards, the mixture was allowed to cool to room temperature and was quenched by addition of a sat. bicarb solution (5 mL). The mixture was extracted with CH_2Cl_2 (3 × 20 mL), and the combined organic layers were washed with brine (20 mL) and dried over Na_2SO_4. After filtration, the solvent was removed under reduced pressure and the residual crude product was subjected to FCC (SiO_2, EtOAc) to yield the desired racemic substrate *rac*-**2n** as a white solid.

TLC (EtOAc): R_f = 0.38 [UV] [$KMnO_4$].

M.p.: 116 °C.

^{1}H-NMR (400 MHz, $CDCl_3$, 300 K): δ [ppm] = 8.78 (d, ^3J = 2.7 Hz, 1H, H2), 8.71 (d, ^3J = 2.7 Hz, 1H, H3), 8.48 (bs, 1H, NH), 6.00 (d, ^3J = 1.5 Hz, 1H, H7), 4.06 – 3.95 (m, 1H, H_a1'), 3.89 – 3.79 (m, 1H, H_b1'), 3.70 – 3.57 (m, 2H, H2').

^{13}C-NMR (101 MHz, $CDCl_3$, 300 K): δ [ppm] = 166.6 (C5), 157.1 (C7a), 147.5 (C3), 147.4 (C2), 144.8 (C4a), 82.8 (C7), 68.1 (C1'), 42.6 (C2').

HRMS (ESI) m/z: Calculated for $[M+H]^+$: 214.0378; found: 214.0378.

IR (film): $\tilde{\nu}_{max}/cm^{-1}$ = 3231 (m, NH), 3072 (m, CH_{arom}), 2961 (m, CH_{aliph}), 1722 (s, C=O), 1380 (m, CN), 1102 (s, CO), 788 (CCl_{aliph}).

7-Isobutoxy-6,7-dihydro-5*H*-pyrrolo[3,4-*b*]pyrazin-5-one (*rac*-2o)

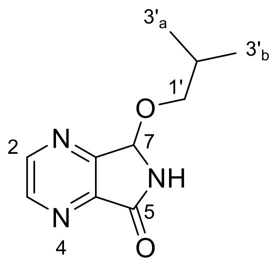

rac-**2o**
$C_{10}H_{13}N_3O_2$
M = 207.23 g mol^{-1}

According to GP C, to a suspension of **SI-11** (100 mg, 662 µmol, 1.00 equiv.) in 2-methylpropan-1-ol (1.23 mL, 981 mg, 13.2 mmol, 20.0 equiv.) was added *p*-TsOH (13.6 mg, 66.2 µmol, 0.10 equiv.). The resulting mixture was then stirred at 100 °C for 14 h. Afterwards, the mixture was allowed to cool to room temperature and was quenched by addition of a sat. bicarb solution (5 mL). The mixture was extracted with CH_2Cl_2 (3 × 20 mL), and the combined organic layers were washed with brine (20 mL) and dried over Na_2SO_4. After filtration, the solvent was removed under reduced pressure and the residual crude product was subjected to FCC (SiO_2, Pn:EtOAc = 1:2 → 0:1) to yield the desired racemic substrate *rac*-**2o** as a white solid.

TLC (EtOAc): R_f = 0.43 [UV] [$KMnO_4$].

M.p.: 146 °C.

^{1}H-NMR (400 MHz, $CDCl_3$, 300 K): δ [ppm] = 8.83 (d, ^3J = 2.6 Hz, 1H, H2), 8.77 (d, ^3J = 2.6 Hz, 1H, H3), 8.57 (bs, 1H, NH), 5.97 (d, ^3J = 1.4 Hz, 1H, H7), 3.55 (dd, ^2J = 8.6 Hz, ^3J = 6.6 Hz, 1H, H_a1'), 3.38 (dd, ^2J = 8.6 Hz, ^3J = 6.6 Hz, 1H), 1.93 (*virt.* non., $^3J \approx {^3J} \approx {^3J} \approx$ 6.6 Hz, 1H, H2'), 0.94 (d, ^3J = 6.6 Hz, 3H, H_a3'), 0.93 (d, ^3J = 6.6 Hz, 3H, H_b3').

^{13}C-NMR (101 MHz, $CDCl_3$, 300 K): δ [ppm] = 166.8 (C5), 157.6 (C7a), 147.3 (C3), 147.1 (C2), 144.9 (C4a), 82.8 (C7), 74.8 (C1'), 28.7 (C2'), 19.3 (C_a3'), 19.3 (C_b3').

HRMS (ESI) m/z: Calculated for $[M+H]^+$: 208.1081; found: 208.1081.

IR (film): $\tilde{v}$ max/cm^{-1} = 3223 (m, NH), 3060 (m, CH_{arom}), 2913 (m, CH_{aliph}), 2874 (m, CH_{aliph}), 1692 (s, C=O), 1338 (m, CN), 1070 (s, CO).

7-(But-3-en-1-yloxy)-6,7-dihydro-5*H*-pyrrolo[3,4-*b*]pyrazin-5-one (*rac*-2p)

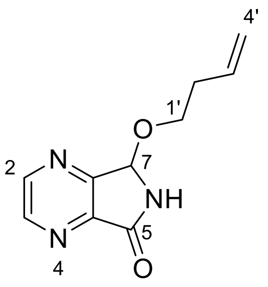

rac-**2p**

$C_{10}H_{11}N_3O_2$

M = 205.22 g mol^{-1}

According to GP C, to a suspension of **SI-11** (100 mg, 662 µmol 1.00 equiv.) in but-3-en-1-ol (1.14 mL, 954 mg, 13.2 mmol, 20.0 equiv.) was added *p*-TsOH (13.6 mg, 66.2 µmol, 0.10 equiv.). The resulting mixture was then stirred at 80 °C for 16 h. Afterwards, the mixture was allowed to cool to room temperature and was quenched by addition of a sat. bicarb solution (5 mL). The mixture was extracted with CH_2Cl_2 (3 × 20 mL), and the combined organic layers were washed with brine (20 mL) and dried over Na_2SO_4. After filtration, the solvent was removed under reduced pressure and the residual crude product was subjected to FCC (SiO_2, Pn:EtOAc = 1:2 → 0:1) to yield the desired racemic substrate *rac*-**2p** as a white solid.

TLC (EtOAc): R_f = 0.58 [UV] [$KMnO_4$].

M.p.: 87 °C.

^{1}H-NMR (400 MHz, $CDCl_3$, 300 K): δ [ppm] = 8.76 (d, ^3J = 2.6 Hz, 1H, H2), 8.69 (d, ^3J = 2.6 Hz, 1H, H3), 7.79 (bs, 1H, NH), 5.90 (d, ^3J = 1.4 Hz, 1H, H7), 5.74 (ddt, ^3J = 17.1 Hz, ^3J = 10.2 Hz, 6.7 Hz, 1H, H3’), 5.05 (*virt.* dq, ^3J = 17.3 Hz, $^2J \approx {}^4J \approx$ 1.6 Hz, 1H, H_a4’), 5.00 (*virt.* dq, ^3J = 10.2 Hz, $^2J \approx {}^4J \approx$ 1.5 Hz, 1H, H_b4’), 3.76 (dt, ^3J = 8.9 Hz, ^3J = 6.7 Hz, 1H, H_a1’), 3.56 (dt, ^3J = 8.9 Hz, ^3J = 6.7 Hz, 1H, H_b1’), 2.35 (qt, $^3J \approx {}^3J \approx$ 6.7 Hz, $^2J \approx {}^2J \approx$ 1.4 Hz, 2H, H2’).

^{13}C-NMR (101 MHz, $CDCl_3$, 300 K): δ [ppm] = 166.2 (C5), 157.4 (C7a), 147.3 (C3), 144.8 (C2), 144.9 (C4a), 134.3 (C3’), 117.4 (C4’), 82.6 (C7), 67.3 (C1’), 34.1 (C2’).

HRMS (ESI) m/z: Calculated for $[M+H]^+$: 206.0924; found: 206.0924.

IR (film): $\tilde{v}$ max/cm^{-1} = 3193 (m, NH), 2981 (m, CH_{aliph}), 2874 (m, CH_{aliph}), 1691 (s, C=O), 1380 (m, CN), 1082 (s, CO).

7-Isopropoxy-6,7-dihydro-5*H*-pyrrolo[3,4-*b*]pyrazin-5-one (*rac*-2q)

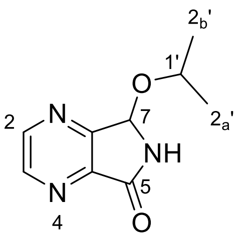

rac-**2q**

$C_9H_{11}N_3O_2$

M = 193.21 g mol^{-1}

According to GP C, to a suspension of **SI-11** (100 mg, 662 µmol 1.00 equiv.) in propan-2-ol (1.01 mL, 795 mg, 13.2 mmol, 20.0 equiv.) was added *p*-TsOH (13.6 mg, 66.2 µmol, 0.10 equiv.). The resulting mixture was then stirred at 80 °C for 2 h. Afterwards, the mixture was allowed to cool to room temperature and was quenched by addition of a sat. bicarb solution (5 mL). The mixture was extracted with CH_2Cl_2 (3 × 20 mL), and the combined organic layers were washed with brine (20 mL) and dried over Na_2SO_4. After filtration, the solvent was removed under reduced pressure and the residual crude product was subjected to FCC (SiO_2, Pn:EtOAc = 1:2 → 0:1) to yield the desired racemic substrate *rac*-**2q** (102 mg, 528 µmol, 80%) as a white solid.

TLC (EtOAc): R_f = 0.45 [UV] [$KMnO_4$].

M.p.: 134 °C.

^{1}H-NMR (400 MHz, $CDCl_3$, 300 K): δ [ppm] = 8.81 (d, ^3J = 2.6 Hz, 1H, H2), 8.74 (d, ^3J = 2.6 Hz, 1H, H3), 8.14 (bs, 1H, NH), 5.97 (d, ^3J = 1.3 Hz, 1H, H7), 4.14 (*virt.* hept., $^3J \approx {}^3J \approx$ 6.1 Hz, 1H, H1’), 1.36 (d, ^3J = 6.1 Hz, 3H, $H2_a$’), 1.31 (d, ^3J = 6.1 Hz, 3H, $H2_b$’).

^{13}C-NMR (101 MHz, $CDCl_3$, 300 K): δ [ppm] = 166.5 (C5), 158.0 (C7a), 147.3 (C3), 147.1 (C2), 144.7 (C4a), 81.3 (C7), 72.2 (C1’), 23.1 (C_a2’), 22.7 (C_b2’).

HRMS (ESI) m/z: Calculated for $[M+H]^+$: 194.0924; found: 194.0924.

IR (film): $\tilde{v}$ max/cm^{-1} = 3255 (m, NH), 3068 (m, CH_{arom}), 2975 (m, CH_{aliph}), 2935 (m, CH_{aliph}), 1721 (s, C=O), 1379 (m, CN), 1070 (s, CO).

7-(3-Methoxy-3-methylbutoxy)-6,7-dihydro-5*H*-pyrrolo[3,4-*b*]pyrazin-5-one (*rac*-2r)

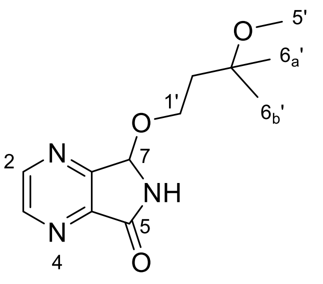

rac-**2r**
$C_{12}H_{17}N_3O_3$
M = 251.29 g mol^{-1}

According to GP C, to a suspension of **SI-11** (100 mg, 662 µmol 1.00 equiv.) in 3-methoxy-3-methylbutan-1-ol (1.68 mL, 1.56 g, 13.2 mmol, 20.0 equiv.) was added *p*-TsOH (13.6 mg, 66.2 µmol, 0.10 equiv.). The resulting mixture was then stirred at 90 °C for 14 h. Afterwards, the mixture was allowed to cool to room temperature and was quenched by addition of a sat. bicarb solution (5 mL). The mixture was extracted with CH_2Cl_2 (3 × 20 mL), and the combined organic layers were washed with brine (20 mL) and dried over Na_2SO_4. After filtration, the solvent was removed under reduced pressure and the residual crude product was subjected to FCC (SiO_2, EtOAc) to yield the desired racemic substrate *rac*-**2r** (96 mg, 382 µmol, 56%) as a white solid.

TLC (EtOAc): R_f = 0.24 [UV] [$KMnO_4$].

M.p.: 82 °C.

^{1}H-NMR (400 MHz, $CDCl_3$, 300 K): δ [ppm] = 8.76 (d, ^3J = 2.6 Hz, 1H, H2), 8.68 (d, ^3J = 2.6 Hz, 1H, H3), 7.48 (bs, 1H, NH), 5.87 (d, ^3J = 1.3 Hz, 1H, H7), 3.81 (ddd, ^3J = 9.2 Hz, ^3J = 7.9 Hz, ^3J = 6.4 Hz, 1H, H1$_a$'), 3.60 (ddd, ^3J = 9.2 Hz, ^3J = 7.9 Hz, ^3J = 6.4 Hz, 1H, H1$_b$'), 3.10 (s, 3H, H5'), 1.88 – 1.72 (m, 2H, H2'), 1.10 (s, 3H, H6$_a$'), 1.10 (s, 3H, H6$_b$').

^{13}C-NMR (101 MHz, $CDCl_3$, 300 K): δ [ppm] = 166.9 (C5), 157.4 (C7a), 147.3 (C2/C3), 147.3 (C3/C2), 144.8 (C4a), 82.7 (C7), 73.8 (C3'), 64.5 (C1'), 49.4 (C5'), 39.7 (C2'), 25.4, (C6$_a$', C6$_b$').

HRMS (ESI) m/z: Calculated for [M+H]$^+$: 252.1343; found: 252.1343.

IR (film): $\tilde{v}$ max/cm^{-1} = 3301 (m, NH), 2972 (m, CH$_{arom}$), 2940 (m, CH$_{aliph}$), 2884 (m, CH$_{aliph}$), 1721 (s, C=O), 1381 (m, CN), 1080 (s, CO).

7-(2,2,2-Trifluoroethoxy)-6,7-dihydro-5*H*-pyrrolo[3,4-*b*]pyrazin-5-one (*rac*-2s)

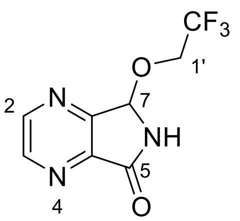

rac-**2s**

$C_9H_8F_3N_3O_2$

M = 247.18 g mol^{-1}

According to GP C, to a suspension of **SI-11** (100 mg, 662 µmol 1.00 equiv.) in 2,2,2-trifluoroethan-1-ol (959 µL, 1.32 g, 13.2 mmol, 20.0 equiv.) was added *p*-TsOH (13.6 mg, 66.2 µmol, 0.10 equiv.). The resulting mixture was then stirred at 80 °C for 10 min. Afterwards, the mixture was allowed to cool to room temperature and was quenched by addition of a sat. bicarb solution (5 mL). The mixture was extracted with CH_2Cl_2 (3 × 20 mL), and the combined organic layers were washed with brine (20 mL) and dried over Na_2SO_4. After filtration, the solvent was removed under reduced pressure and the residual crude product was subjected to FCC (SiO_2, SiO_2, Pn:EtOAc = 1:2 → 0:1) to yield the desired racemic substrate *rac*-**2s** (64 mg, 275 µmol, 41%) as a white solid.

TLC (EtOAc): R_f = 0.64 [UV] [$KMnO_4$].

M.p.: 148 °C.

^{1}H-NMR (400 MHz, $CDCl_3$, 300 K): δ [ppm] = 8.90 (d, ^3J = 2.6 Hz, 1H, H2), 8.80 (d, ^3J = 2.6 Hz, 1H, H3), 7.82 (bs, 1H, NH), 6.11 (d, ^3J = 1.6 Hz, 1H, H7), 4.16 (dq, ^2J = 12.1 Hz, $^3J_{H-F}$ = 8.5 Hz, 1H, H1$_a$'), 4.05 (dq, ^3J = 12.1 Hz, $^3J_{H-F}$ = 8.3 Hz, 1H, H1$_b$').

^{13}C-NMR (101 MHz, $CDCl_3$, 300 K): δ [ppm] = 165.9 (C5), 156.4 (C7a), 147.9 (C2), 147.7 (C3), 144.6 (C4a), 123.4 (q, $^1J_{CF}$ = 278.4 Hz, C2'), 82.7 (C7), 64.7 (q, $^2J_{CF}$ = 35.5 Hz, C1').

HRMS (ESI) m/z: Calculated for $[M+H]^+$: 234.0485; found: 234.0485.

IR (film): $\tilde{v}$max/cm^{-1} = 3297 (m, NH), 3091 (m, CH_{arom}), 2958 (m, CH_{aliph}), 1726 (s, C=O), 1388 (m, CN), 1133 (CF_{aliph}), 1070 (s, CO).

7-((*tert*-Butyldimethylsilyl)oxy)-6,7-dihydro-5*H*-pyrrolo[3,4-*b*]pyrazin-5-one (*rac*-2t)

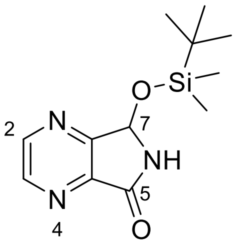

rac-**2t**

$C_{12}H_{19}N_3O_2Si$

M = 265.39 g mol^{-1}

According to GP D, to a solution of **SI-11** (100 mg, 662 µmol 1.00 equiv.) in DMF (3 mL) was added imidazole (90.1 mg, 1.32 mmol, 1.50 equiv.) and *tert*-butylchlorodimethylsilane (159µL, 150 mg, 993 µmol, 2.00 equiv.) at room temperature. The resulting mixture was stirred for 16 h at the same temperature. Afterwards, the mixture was quenched by the addition of a sat. NH_4Cl solution (5 mL). The mixture was extracted with CH_2Cl_2 (3 × 20 mL), and the combined organic layers were washed with brine (20 mL) and dried over Na_2SO_4. After filtration, the solvent was removed under reduced pressure and the residual crude product was subjected to FCC (SiO_2, Pn:EtOAc = 1:2) to yield the desired racemic substrate *rac*-**2t** (113 mg, 426 µmol, 64%) as a white solid.

TLC (Pn:EtOAc = 1:2): R_f = 0.47 [UV] [$KMnO_4$].

M.p.:.> 260°C.

^{1}H-NMR (400 MHz, $CDCl_3$, 300 K): δ [ppm] = 8.78 (d, ^3J = 2.6 Hz, 1H, H2), 8.72 (d, ^3J = 2.6 Hz, 1H, H3), 7.24 (bs, 1H, NH), 6.13 (d, ^3J = 1.5 Hz, 1H, H7), 0.94 [s, 9H, C(C*H*$_3$)$_3$], 0.24 (s, 3H, Si-C*H*$_3$CH$_3$), 0.21 (s, 3H, Si-CH$_3$C*H*$_3$).

^{13}C-NMR (101 MHz, $CDCl_3$, 300 K): δ [ppm] = 166.0 (C5), 159.2 (C7a), 147.2 (C3), 146.9 (C2), 144.3 (C4a), 77.9 (C7), 25.7 (C(*C*H$_3$)$_3$), 18.27 (*C*(CH$_3$)$_3$), –4.13 (Si-*C*H$_3$CH$_3$), –4.17 (Si-CH$_3$*C*H$_3$).

HRMS (ESI) m/z: Calculated for $[M+H]^+$: 265.1319; found: 265.1335.

IR (film): $\tilde{v}$ max/cm^{-1} = 3276 (m, NH), 2953 (m, CH$_{arom}$), 2858 (m, CH$_{aliph}$), 1701 (s, C=O), 1384 (m, CN), 1084 (s, CO).

7-((Triethylsilyl)oxy)-6,7-dihydro-5*H*-pyrrolo[3,4-*b*]pyrazin-5-one (*rac*-2u)

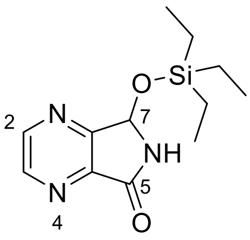

rac-**2u**
$C_{12}H_{19}N_3O_2Si$
M = 265.39 g mol^{-1}

According to GP D, to a solution of **SI-11** (100 mg, 662 µmol 1.00 equiv.) DMF was added imidazole (90.1 mg, 1.32 mmol, 1.50 equiv.) and chlorotriethylsilane (167 µL, 150 mg, 993 µmol, 2.00 equiv.) at room temperature. The resulting mixture was stirred for 16 h at the same temperature. Afterwards, the mixture was quenched by addition of a sat. NH_4Cl solution (5 mL). The mixture was extracted with CH_2Cl_2 (3 × 20 mL), and the combined organic layers were washed with brine (20 mL) and dried over Na_2SO_4. After filtration, the solvent was removed under reduced pressure and the residual crude product was subjected to FCC (SiO_2, Pn:EtOAc = 1:1) to yield the desired racemic substrate *rac*-**2u** (136 mg, 513 µmol, 77%) as a white solid.

TLC (Pn:EtOAc = 1:1): R_f = 0.32 [UV] [$KMnO_4$].

M.p.: 118°C.

^{1}H-NMR (400 MHz, $CDCl_3$, 300 K): δ [ppm] = 8.79 (d, ^3J = 2.6 Hz, 1H, H2), 8.72 (d, ^3J = 2.6 Hz, 1H, H3), 7.02 (bs, 1H, NH), 6.12 (d, ^3J = 1.4 Hz, 1H), 1.01 (t, ^3J = 7.9 Hz, 9H, 3 CH_3), 0.83 – 0.64 (m, 6H, 3 CH_2).

^{13}C-NMR (101 MHz, $CDCl_3$, 300 K): δ [ppm] = 165.6 (C5), 159.1 (C7a), 147.3 (C3), 146.9 (C2), 144.2 (C4a), 77.6 (C7), 6.7 (Si-CH$_3$CH$_3$), 5.1 (Si-CH$_3$$CH_3$).

HRMS (ESI) m/z: Calculated for [M+H]$^+$: 266.1319; found:266.1318.

IR (film): $\tilde{\nu}$max/cm^{-1} = 3198 (m, NH), 2955 (m, CH$_{arom}$), 2877 (m, CH$_{aliph}$), 1736 (s, C=O), 1346 (m, CN), 1083 (s, CO).

7-(4-(*tert*-Butyl)benzyl)-6-methyl-6,7-dihydro-5*H*-pyrrolo[3,4-*b*]pyrazin-5-one (*rac*-**2a**-Me)

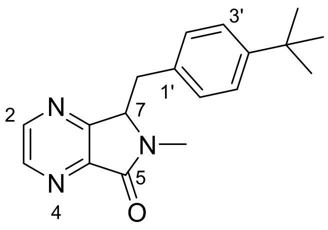

rac-**2a**-Me
$C_{18}H_{21}N_3O$
M = 295.39 g mol^{-1}

According to a modified procedure,[75] to a solution of *rac*-**2a** (60.0 mg, 213 µmol, 1.00 equiv.) in DMF (1.0 mL) was added Cs_2CO_3 (104 mg, 320 µmol, 1.50 equiv.) and methyl iodide (53.1 µL, 121 mg, 853 µmol, 4.00 equiv.) at 0°C. The resulting mixture was allowed to warm to room temperature and was stirred for 48 h. Afterwards, the mixture was quenched by the addition conc. ammonia solution (0.5 mL) and sat. NH_4Cl solution (5 mL). The mixture was extracted with CH_2Cl_2 (3 × 10 mL), and the combined organic layers were washed with brine (20 mL) and dried over Na_2SO_4. After filtration, the solvent was removed under reduced pressure and the residual crude product was subjected to FCC (SiO_2, Pn:EtOAc = 1:1 → 0:1) to yield the desired methylated substrate *rac*-**2a**-Me (22 mg, 74.5 µmol, 22%) as a white solid.

TLC (EtOAc): R_f = 0.25 [UV] [$KMnO_4$].

M.p.: 145 °C.

^{1}H-NMR (400 MHz, $CDCl_3$, 300 K): δ [ppm] = 8.68 (s, 2H, H2, H3), 7.19 – 7.13 (m, 2H, H2', H6'), 6.86 – 6.78 (m, 2H, H3', H5'), 4.80 (t, ^3J = 4.7 Hz, 1H), 3.46 (dd, ^2J = 14.6 Hz, ^3J = 4.7 Hz, 1H), 3.35 (dd, ^2J = 14.6, ^3J = 4.7 Hz, 1H), 3.23 (s, 3H, CH_3), 1.22 [s, 9H, $C(CH_3)_3$].

^{13}C-NMR (126 MHz, $CDCl_3$, 300 K): δ [ppm] = 165.1 (C5), 158.7 (C7a), 150.2 (C4'), 146.0 (C2/C3) 145.8 (C2/C3), 145.1 (C4a), 131.2 (C1'), 129.1 (C3', C5'), 125.5 (C2', C6'), 61.9 (C7), 35.5 (C3-*C*H_2-C1'), 34.5 [*C*$(CH_3)_3$], 31.3 [C(*C*$H_3)_3$], 28.9 (CH_3).

HRMS (ESI) m/z: Calculated for $[M+H]^+$: 296.1757; found: 296.1767.

7-(4-chlorobenzyl)-6,7-dihydro-5H-pyrrolo[3,4-b]pyrazin-5-one (*rac*-SI-6)

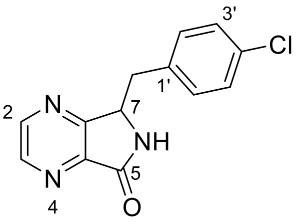

rac-**SI-6**
$C_{13}H_{10}ClN_3O$
M = 259.69 g mol^{-1}

According to GP A, a 1 M solution of KHMDS (1.08 mL, 216 mg, 1.08 mmol, 1.10 equiv.) was added dropwise to a suspension of 6-(4-methoxybenzyl) 6,7-dihydro-5*H*-pyrrolo[3,4-*b*]pyrazin-5-one **SI-9** (250 mg, 979 µmol, 1.00 equiv.) in anhydrous THF (30 mL) at –78 °C. After 20 min, 1-chloro-4-(bromomethyl)benzene (262 mg, 1.27 mmol, 1.30 equiv.) was added in one portion. The reaction was left to stir for 4 h at –78 °C. Afterwards, the solution was allowed to warm up to room temperature and quenched by the addition of sat. NH_4Cl (6 mL) and distilled water (20 mL). The aqueous layer was extracted with CH_2Cl_2 (3 × 25 mL), and the combined organic phases were dried over Na_2SO_4, filtered and concentrated under reduced pressure. The crude product was then dissolved in MeCN:H_2O (3:1) (29 mL) and diammonium cerium(IV) nitrate (1.61 g, 2.94 mmol, 3.00 equiv.) was added to the solution at room temperature. The yellow solution was stirred for 90 min at the same temperature. Subsequently, water (30 mL) was added, and the mixture was extracted with CH_2Cl_2 (3 × 50 mL). The combined organic layers were washed with brine (50 mL) and dried over Na_2SO_4. After filtration, the solvent was removed under reduced pressure and the residual crude product was subjected to FCC (SiO_2, EtOAc) to yield the desired racemic substrate *rac*-**SI-6** (69.5 mg, 270 µmol, 23%) as a white solid**.**

TLC (EtOAc): R_f = 0.12 [UV] [$KMnO_4$].

M.p.: 226 °C.

^{1}H-NMR (400 MHz, DMSO-d6, 300 K): δ [ppm] = 9.33 (s, 1H), 8.81 (d, ^3J = 2.7 Hz, 1H, H2), 8.74 (d, ^3J = 2.6 Hz, 1H, H3), 7.24 – 7.16 (m, 2H, , H2', H6'), 7.04 – 6.96 (m, 2H, , H3', H5'), 5.08 – 5.01 (m, 1H, H7), 3.21 (m, 2H, C7-CH_a-C1', C7-CH_b-C1').

^{13}C-NMR (101 MHz, DMSO-d6, 300 K): δ [ppm] = 165.4 (C5), 159.8 (C7a), 146.3 (C2), 145.6 (C3), 144.6 (C4a), 134.3 (C1'), 131.5 (C3', C5'), 131.2 (C4'), 127.7 (C2', C6'), 55.2 (C7), 36.4 (C3-*C*H_2-C1').

HRMS (ESI) m/z: Calculated for $[M+H]^+$: 260.0585; found: 260.0584.

IR (film) $\tilde{\nu}_{max}$/cm^{-1}**:** 3222 (m, NH), 3092 (m, CH_{arom}), 2923 (m, CH_{aliph}) 2853 (m, CH_{aliph}), 1704 (s, C=O), 1457 (m, $C{=}C_{arom}$), 1377 (m, $C{=}C_{arom}$), 1157 (m, CN).

7-Phenyl-6,7-dihydro-5*H*-pyrrolo[3,4-*b*]pyrazin-5-one (*rac*-SI-7)

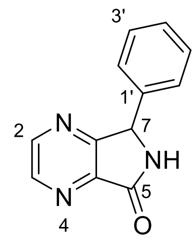

rac-**SI-7**
$C_{12}H_9N_3O$
M = 211.22 g mol^{-1}

According to a modified procedure,[3] to a suspension of **SI-11** (100 mg, 662 µmol, 1.00 equiv.) in 660 µL conc. sulfuric acid was added benzene (1.17 mL, 1.03 g, 13.2 mmol, 20.0 equiv.). The resulting suspension was stirred for 90 minutes at room temperature. Subsequently, the reaction was quenched by the addition of sat. $NaHCO_3$ (20 mL). The aqueous layer was extracted with CH_2Cl_2 (3 × 15 mL), and the combined organic layers were washed with brine (30 mL) and dried over Na_2SO_4. After filtration, the solvent was removed under reduced pressure and the residual crude product was subjected to FCC (SiO_2, EtOAc = 1) to yield the desired racemic substrate *rac*-**SI-7** (55.0 mg, 260 µmol, 39%) as a white solid**.**

TLC (EtOAc): R_f = 0.40 [UV] [$KMnO_4$].

M.p.: >260 °C.

^{1}H-NMR (400 MHz, DMSO-d6, 300 K): δ [ppm] = 9.75 (s, 1H), 8.83 (d, ^3J = 2.6 Hz, 1H, H2), 8.73 (d, ^3J = 2.6 Hz, 1H, H3), 7.43 – 7.29 (m, 5H, H2', H3', H4', H5', H6'), 5.86 (s, 1H, H7).

^{13}C-NMR (101 MHz, DMSO-d6, 300 K): δ [ppm] = 165.8 (C5), 160.7 (C7a), 146.8 (C3), 145.9 (C2), 143.5 (C4a), 136.7 (C1'), 128.6 (C3', C5'), 128.2 (C4'), 127.1 (C2', C6'), 58.4 (C7).

HRMS (ESI) m/z: Calculated for $[M+H]^+$: 212.0818; found: 212.0818.

IR (film) $\tilde{\nu}_{max}$/cm^{-1}**:** 3221 (m, NH), 3036 (m, CH_{arom}), 2954 (m, CH_{aliph}) 2924 (m, CH_{aliph}), 1697 (s, C=O), 1379 (m, $C{=}C_{arom}$), 1159 (m, CN).

3-Benzylisoindolin-1-one (*rac*-3)

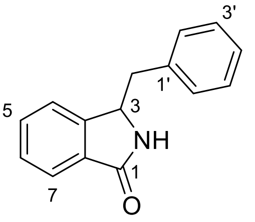

rac-**3**

$C_{15}H_{13}NO$

M = 223.28 g mol^{-1}

According to a modified procedure, a 1 M solution of KHMDS (1.41 mL, 283 mg, 1.41 mmol, 1.10 equiv.) was added dropwise to a suspension of tert-butyl 1-oxoisoindoline-2-carboxylate (300 mg, 1.28 mmol, 1.00 equiv.) in anhydrous THF (23 mL) at –78 °C. After 20 min, benzyl bromide (200 µL, 286 mg, 1.67 mmol, 1.30 equiv.) was added in one portion. The reaction was left to stir for 2 h at –78 °C. Afterwards, the solution was allowed to warm up to room temperature and HCl in 1,4 dioxane (6.43 mL, 937 mg, 25.7 mmol, 20.0 equiv.) was added and the mixture was stirred for an additional 16 h. The reaction was quenched by the addition of sat. $NaHCO_3$ (20 mL) The aqueous layer was extracted with CH_2Cl_2 (3 × 25 mL), and the combined organic layers were washed with brine (50 mL) and dried over Na_2SO_4. After filtration, the solvent was removed under reduced pressure and the residual crude product was subjected to FCC (SiO_2, Pn:EtOAc = 1:1) to yield the desired racemic substrate *rac*-**3** (210 mg, 941 µmol, 73%) as a white solid**.**

TLC (Pn:EtOAc = 1:1): R_f = 0.25 [UV] [$KMnO_4$].

^{1}H-NMR (400 MHz, $CDCl_3$, 300 K): δ [ppm] = 7.85 (dd, ^3J = 7.5 Hz, ^4J = 1.2 Hz, 1H, H7), 7.56 (virt. td, $^3J \approx {}^3J \approx$ 7.5 Hz, ^4J = 1.2 Hz, 1H, H5), 7.48 (virt. td, $^3J \approx {}^3J \approx$ 7.5 Hz, ^3J = 0.9 Hz, 1H, H6), 7.38-7.31 (m, 3H, H4, H3'), 7.31-7.26 (m, 1H, H4'), 7.26-7.22 (m, 2H, H2'), 6.58 (bs, 1H, NH), 4.81 (dd, ^3J = 9.1 Hz, ^3J = 5.1 Hz, 1H, H3), 3.25 (dd, ^2J = 13.6 Hz, ^3J = 5.1 Hz, 1H, C3-CH_a-C1'), 2.80 (dd, ^2J = 3.6 Hz, ^3J = 9.1 Hz, 1H, C3-CH_b-C1').

^{13}C-NMR (101 MHz, $CDCl_3$, 300 K): δ [ppm] = 170.5 (C1), 147.0 (C3a), 137.0, (C1') 132.1 (C5), 131.8 (C7a), 129.3 (C2', C6'), 129.0 (C3', C5'), 128.6 (C6), 127.4 (C4'), 124.1 (C7), 122.8 (C4), 58.4 (C3), 41.5 (C3-CH_a-C1', C3-CH_b-C1').

The analytical data are in accordance with the literature.[76]

12. Photochemical Deracemization Reactions

(*R*)-7-(4-(*tert*-Butyl)benzyl)-6,7-dihydro-5*H*-pyrrolo[3,4-*b*]pyrazin-5-one (2a)

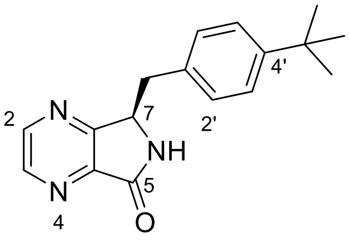

2a
$C_{17}H_{19}N_3O$
M = 281.36 g mol^{-1}

According to GP E, a degassed solution of *rac*-**2a** (7.03 mg, 25.0 µmol, 1.00 equiv.) and enantiomerically pure (+)-benzophenone **1b** (252 µg, 625 nmol, 2.5 mol%) in 10 mL α,α,α-trifluorotoluene was irradiated at λ = 366 nm for 8 h. After irradiation, the solvent was evaporated and the residue was purified by FCC (SiO_2, EtOAc) to obtain **2a** (6.33 mg, 22.5 µmol, 90%, 98% *ee*) as a colorless solid.

TLC (EtOAc): R_f = 0.21 [UV] [$KMnO_4$].

Optical Rotation: $[\alpha]_D^{25}$: +108 (*c* = 1.0, CH_2Cl_2) [98% *ee*].

Chiral HPLC: 98% *ee* (AS-H 250 × 4.6 mm, *n*-Hep/*iso*-PrOH = 50/50, 1 mL/min, λ = 210 nm); t_R = 14.84 min (minor, *ent*-**2a**), 28.56 min (major, **2a**).

(*R*)-7-Benzyl-6,7-dihydro-5*H*-pyrrolo[3,4-*b*]pyrazin-5-one (2b)

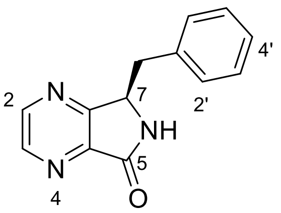

2b
$C_{13}H_{11}N_3O$
M = 225.25g mol^{-1}

According to GP E, a degassed solution of *rac*-**2b** (5.63 mg, 25.0 µmol, 1.00 equiv.) and enantiomerically pure (+)-benzophenone **1b** (252 µg, 625 nmol, 2.5 mol%) in 10 mL α,α,α-trifluorotoluene was irradiated at λ = 366 nm for 8 h. After irradiation, the solvent was evaporated and the residue was purified by flash column chromatography (SiO_2, EtOAc) to obtain **2b** (4.63 mg, 20.6 µmol, 82%, 99% *ee*) as a colorless solid.

TLC (EtOAc): R_f = 0.18 [UV] [$KMnO_4$].

Optical Rotation: $[\alpha]_D^{25}$: +46 (*c* = 1.0, CH_2Cl_2) [99% *ee*].

Chiral HPLC: 99% *ee* (AD-H 250 × 4.6 mm, *n*-Hep/*iso*-PrOH = 90/10, 1 mL/min, λ = 210 nm); t_R = 26.18 min (minor, *ent*-**2b**), 29.66 min (major, **2b**).

(*R*)-7-(4-(Trifluoromethyl)benzyl)-6,7-dihydro-5*H*-pyrrolo[3,4-*b*]pyrazin-5-one (2c)

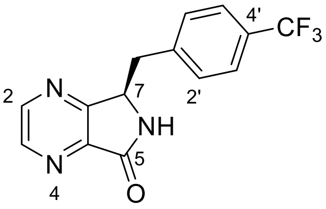

2c

$C_{14}H_{10}F_3N_3O$

M = 293.25 g mol^{-1}

According to GP E, a degassed solution of *rac*-**2c** (7.33 mg, 25.0 µmol, 1.00 equiv.) and enantiomerically pure (+)-benzophenone **1b** (252 µg, 625 nmol, 2.5 mol%) in 10 mL α,α,α-trifluorotoluene was irradiated at λ = 366 nm for 8 h. After irradiation, the solvent was evaporated and the residue was purified by flash column chromatography (SiO_2, EtOAc) to obtain **2c** (6.09 mg, 20.8 µmol, 83%, 95% *ee*) as a colorless solid.

TLC (EtOAc): R_f = 0.21 [UV] [$KMnO_4$].

Optical Rotation: $[\alpha]_D^{25}$: +98 (*c* = 1.0, CH_2Cl_2) [95% *ee*].

Chiral HPLC: 95% *ee* (AD-H 250 × 4.6 mm, *n*-Hep/*iso*-PrOH = 70/30, 1 mL/min, λ = 210 nm); t_R = 5.43 min (minor, *ent*-**2c**), 7.82 min (major, **2c**).

(*R*)-7-(4-Bromobenzyl)-6,7-dihydro-5*H*-pyrrolo[3,4-*b*]pyrazin-5-one (2d)

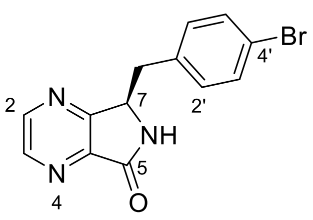

2d

$C_{13}H_{10}BrN_3O$

M = 304.15 g mol^{-1}

According to GP E, a degassed solution of *rac*-**2d** (7.60 mg, 25.0 µmol, 1.00 equiv.) and enantiomerically pure (+)-benzophenone **1b** (252 µg, 625 nmol, 2.5 mol%) in 10 mL α,α,α-trifluorotoluene was irradiated at λ = 366 nm for 8 h. After irradiation, the solvent was evaporated and the residue was purified by flash column chromatography (SiO_2, EtOAc) to obtain **2d** (5.86 mg, 22.5 µmol, 77%, 89% *ee*) as a colorless solid.

TLC (EtOAc): R_f = 0.14 [UV] [$KMnO_4$].

Optical Rotation: $[\alpha]_D^{25}$: +108 (*c* = 1.0, CH_2Cl_2) [89% *ee*].

Chiral HPLC: 89% *ee* (IC 250 × 4.6 mm, *n*-Hep/*iso*-PrOH = 50/50, 1 mL/min, λ = 210 nm); t_R = 20.53 min (major, **2d**), 28.56 min (minor, *ent*-**2d**).

(*R*)- 7-(2-Chlorobenzyl)-6,7-dihydro-5*H*-pyrrolo[3,4-*b*]pyrazin-5-one (2e)

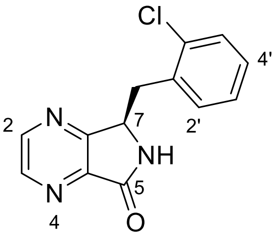

2e

$C_{13}H_{10}ClN_3O$

M = 259.69 g mol^{-1}

According to GP E, a degassed solution of *rac*-**2e** (6.49 mg, 25.0 µmol, 1.00 equiv.) and enantiomerically pure (+)-benzophenone **1b** (252 µg, 625 nmol, 2.5 mol%) in 10 mL α,α,α-trifluorotoluene was irradiated at λ = 366 nm for 8 h. After irradiation, the solvent was evaporated and the residue was purified by flash column chromatography (SiO_2, EtOAc) to obtain **2e** (5.87 mg, 22.6 µmol, 90%, 97% *ee*) as a colorless solid.

TLC (EtOAc): R_f = 0.24 [UV] [$KMnO_4$].

Optical Rotation: $[\alpha]_D^{25}$: +54 (*c* = 1.0, CH_2Cl_2) [97% *ee*].

Chiral HPLC: 97% *ee* (AD-H 250 × 4.6 mm, *n*-Hep/*iso*-PrOH = 70/30, 1 mL/min, λ = 210 nm); t_R = 8.91 min (minor, *ent*-**2e**), 9.87 min (major, **2e**).

(*R*)-7-(4-(Trifluoromethoxy)benzyl)-6,7-dihydro-5*H*-pyrrolo[3,4-*b*]pyrazin-5-one (2f)

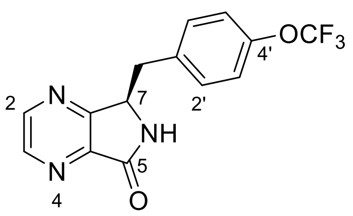

2f

$C_{14}H_{10}F_3N_3O_2$

M = 309.25 g mol^{-1}

According to GP E, a degassed solution of *rac*-**2f** (7.73 mg, 25.0 µmol, 1.00 equiv.) and enantiomerically pure (+)-benzophenone **1b** (252 µg, 625 nmol, 2.5 mol%) in 10 mL α,α,α-trifluorotoluene was irradiated at λ = 366 nm for 8 h. After irradiation, the solvent was evaporated and the residue was purified by flash column chromatography (SiO_2, EtOAc) to obtain **2f** (5.95 mg, 19.2 µmol, 77%, 99% *ee*) as a colorless solid.

TLC (EtOAc): R_f = 0.15 [UV] [$KMnO_4$].

Optical Rotation: $[\alpha]_D^{25}$: +90 (*c* = 1.0, CH_2Cl_2) [99% *ee*].

Chiral HPLC: 99% *ee* (AD-H 250 × 4.6 mm, *n*-Hep/*iso*-PrOH = 70/30, 1 mL/min, λ = 210 nm); t_R = 5.29 min (minor, *ent*-**2f**), 6.90 min (major, **2f**).

(*R*)-7-(3,5-Dimethylbenzyl)-6,7-dihydro-5*H*-pyrrolo[3,4-*b*]pyrazin-5-one (2g)

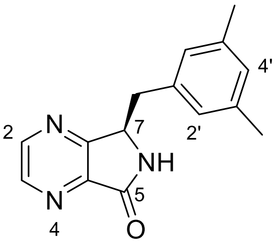

2g

$C_{15}H_{15}N_3O$

M = 253.31 g mol^{-1}

According to GP E, a degassed solution of *rac*-**2g** (6.33 mg, 25.0 µmol, 1.00 equiv.) and enantiomerically pure (+)-benzophenone **1b** (252 µg, 625 nmol, 2.5 mol%) in 10 mL α,α,α-trifluorotoluene was irradiated at λ = 366 nm for 8 h. After irradiation, the solvent was evaporated and the residue was purified by flash column chromatography (SiO_2, EtOAc) to obtain **2g** (4.57 mg, 18.0 µmol, 72%, 91% *ee*) as a colorless solid.

TLC (EtOAc): R_f = 0.26 [UV] [$KMnO_4$].

Optical Rotation: $[\alpha]_D^{25}$: +94 (*c* = 1.0, CH_2Cl_2) [91% *ee*].

Chiral HPLC: 91% *ee* (AD-H 250 × 4.6 mm, *n*-Hep/*iso*-PrOH = 70/30, 1 mL/min, λ = 210 nm); t_R = 6.21 min (major, **2g**), 7.82 min (minor, *ent*-**2g**).

***tert*-Butyl (*R*)-4-((7-oxo-6,7-dihydro-5*H*-pyrrolo[3,4-*b*]pyrazin-5-yl)methyl)benzoate (2h)**

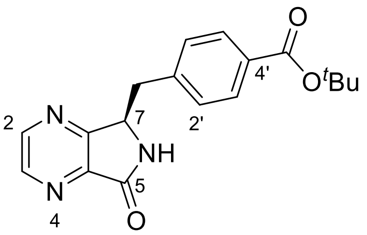

2h

$C_{18}H_{19}N_3O_3$

M = 325.37 g mol^{-1}

According to GP E, a degassed solution of *rac*-**2h** (8.13 mg, 25.0 µmol, 1.00 equiv.) and enantiomerically pure (+)-benzophenone **1b** (252 µg, 625 nmol, 2.5 mol%) in 10 mL α,α,α-trifluorotoluene was irradiated at λ = 366 nm for 8 h. After irradiation, the solvent was evaporated and the residue was purified by flash column chromatography (SiO_2, EtOAc) to obtain **2h** (6.53 mg, 20.1 µmol, 80%, 90% *ee*) as a colorless solid.

TLC (EtOAc): R_f = 0.19 [UV] [$KMnO_4$].

Optical Rotation: $[\alpha]_D^{25}$: +100 (*c* = 1.0, CH_2Cl_2) [90% *ee*].

Chiral HPLC: 90% *ee* (AS-RH 250 × 4.6 mm, MeCN/water = 20/80 → 100/0 over 30 min, 1 mL/min, λ = 210 nm); t_R = 10.27 min (minor, *ent*-**2h**), 13.14 min (major, **2h**).

(*R*)-7-((5-(Trifluoromethyl)furan-2-yl)methyl)-6,7-dihydro-5*H*-pyrrolo[3,4-*b*]pyrazin-5-one (2i)

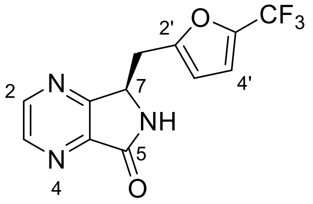

2i
$C_{12}H_8F_3N_3O_2$
M = 283.21 g mol^{-1}

According to GP E, a degassed solution of *rac*-**2i** (7.08 mg, 25.0 µmol, 1.00 equiv.) and enantiomerically pure (+)-benzophenone **1b** (252 µg, 625 nmol, 2.5 mol%) in 10 mL α,α,α-trifluorotoluene was irradiated at λ = 366 nm for 8 h. After irradiation, the solvent was evaporated and the residue was purified by flash column chromatography (SiO_2, EtOAc) to obtain **2i** (6.31 mg, 22.3 µmol, 89%, 90% *ee*) as a colorless solid.

TLC (EtOAc): R_f = 0.26 [UV] [$KMnO_4$].

Optical Rotation: $[\alpha]_D^{25}$: +98 (c = 1.0, CH_2Cl_2) [90% *ee*].

Chiral HPLC: 90% *ee* (AD-H 250 × 4.6 mm, *n*-Hep/*iso*-PrOH = 70/30, 1 mL/min, λ = 210 nm); t_R = 5.25 min (minor, ent-**2i**), 6:06 min (major, **2i**).

(*R*)-7-Ethyl-6,7-dihydro-5*H*-pyrrolo[3,4-*b*]pyrazin-5-one (2j)

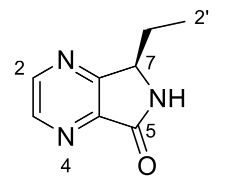

2h
$C_8H_9N_3O$
M = 163.18 g mol^{-1}

According to GP E, a degassed solution of *rac*-**2j** (4.07 mg, 25.0 µmol, 1.00 equiv.) and enantiomerically pure (+)-benzophenone **1b** (252 µg, 625 nmol, 2.5 mol%) in 10 mL α,α,α-trifluorotoluene was irradiated at λ = 366 nm for 8 h. After irradiation, the solvent was evaporated and the residue was purified by flash column chromatography (SiO_2, EtOAc) to obtain **2j** (3.18 mg, 19.5 µmol, 78%, 96% *ee*) as a colorless solid.

TLC (EtOAc): R_f = 0.14 [UV] [$KMnO_4$].

Optical Rotation: $[\alpha]_D^{25}$: +22 (c = 1.0, CH_2Cl_2) [96% *ee*].

Chiral HPLC: 98% *ee* (IC 250 × 4.6 mm, *n*-Hep/*iso*-PrOH = 50/50, 1 mL/min, λ = 210 nm); t_R = 17.55 min (minor, *ent*-**2j**), 23.63 min (major, **2j**).

(*R*)-7-Isopentyl-6,7-dihydro-5*H*-pyrrolo[3,4-*b*]pyrazin-5-one (2k)

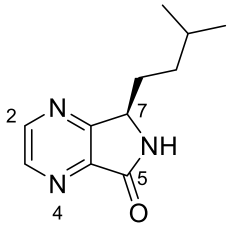

2k

$C_{11}H_{15}N_3O$

M = 205.26 g mol^{-1}

According to GP E, a degassed solution of *rac*-**2k** (5.10 mg, 25.0 µmol, 1.00 equiv.) and enantiomerically pure (+)-benzophenone **1b** (252 µg, 625 nmol, 2.5 mol%) in 10 mL α,α,α-trifluorotoluene was irradiated at λ = 366 nm for 8 h. After irradiation, the solvent was evaporated and the residue was purified by flash column chromatography (SiO_2, EtOAc) to obtain **2k** (4.22 mg, 20.7 µmol, 83%, 98% *ee*) as a colorless solid.

TLC (EtOAc): R_f = 0.26 [UV] [$KMnO_4$].

Optical Rotation: $[\alpha]_D^{25}$: +98 (*c* = 1.0, CH_2Cl_2) [98% *ee*].

Chiral HPLC: 98% *ee* (AD-H 250 × 4.6 mm, *n*-Hep/*iso*-PrOH = 90/10, 1 mL/min, λ = 210 nm); t_R = 11.04 min (major, **2k**), 14.73 min (minor, *ent*-**2k**).

(*R*)-7-(Cyclopentylmethyl)-6,7-dihydro-5*H*-pyrrolo[3,4-*b*]pyrazin-5-one (2l)

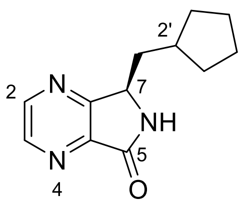

2l

$C_{12}H_{15}N_3O$

M = 217.27 g mol^{-1}

According to GP E, a degassed solution of *rac*-**2l** (4.07 mg, 25.0 µmol, 1.00 equiv.) and enantiomerically pure (+)-benzophenone **1b** (252 µg, 625 nmol, 2.5 mol%) in 10 mL α,α,α-trifluorotoluene was irradiated at λ = 366 nm for 8 h. After irradiation, the solvent was evaporated and the residue was purified by flash column chromatography (SiO_2, EtOAc) to obtain **2l** (3.18 mg, 19.5 µmol, 85%, 98% *ee*) as a colorless solid.

TLC (EtOAc): R_f = 0.31 [UV] [$KMnO_4$].

Optical Rotation: $[\alpha]_D^{25}$: +34 (*c* = 1.0, CH_2Cl_2) [98% *ee*].

Chiral HPLC: 98% *ee* (IC 250 × 4.6 mm, *n*-Hep/*iso*-PrOH = 50/50, 1 mL/min, λ = 210 nm); t_R = 15.99 min (minor, *ent*-**2l**), 26.42 min (major, **2l**).

(*R*)-7-(Cyclopropylmethyl)-6,7-dihydro-5*H*-pyrrolo[3,4-*b*]pyrazin-5-one (2m)

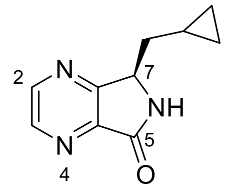

2m

$C_{10}H_{11}N_3O$

M = 189.22 g mol^{-1}

According to GP E, a degassed solution of *rac*-**2m** (4.73 mg, 25.0 µmol, 1.00 equiv.) and enantiomerically pure (+)-benzophenone **1b** (252 µg, 625 nmol, 2.5 mol%) in 10 mL α,α,α-trifluorotoluene was irradiated at λ = 366 nm for 8 h. After irradiation, the solvent was evaporated and the residue was purified by flash column chromatography (SiO_2, EtOAc) to obtain **2m** (3.87 mg, 20.5 µmol, 82%, 98% *ee*) as a colorless solid.

TLC (EtOAc): R_f = 0.26 [UV] [$KMnO_4$].

Optical Rotation: $[\alpha]_D^{25}$: +12 (*c* = 1.0, CH_2Cl_2) [98% *ee*].

Chiral HPLC: 98% *ee* (IC 250 × 4.6 mm, *n*-Hep/*iso*-PrOH = 50/50, 1 mL/min, λ = 210 nm); t_R = 18.18 min (minor, *ent*-**2m**), 25.15 min (major, **2m**).

(*R*)-7-(2-Chloroethoxy)-6,7-dihydro-5*H*-pyrrolo[3,4-*b*]pyrazin-5-one (2n)

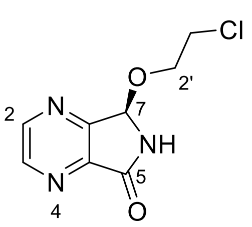

2n

$C_8H_8ClN_3O_2$

M = 213.62 g mol^{-1}

According to GP E, a degassed solution of *rac*-**2n** (5.34 mg, 25.0 µmol, 1.00 equiv.) and enantiomerically pure (+)-benzophenone **1b** (252 µg, 625 nmol, 2.5 mol%) in 10 mL α,α,α-trifluorotoluene was irradiated at λ = 366 nm for 8 h. After irradiation, the solvent was evaporated and the residue was purified by flash column chromatography (SiO_2, EtOAc) to obtain **2n** (5.19 mg, 24.4 µmol, 98%, 96% *ee*) as a colorless solid.

TLC (EtOAc): R_f = 0.38 [UV] [$KMnO_4$].

Optical Rotation: $[\alpha]_D^{25}$: +68 (*c* = 1.0, CH_2Cl_2) [96% *ee*].

Chiral HPLC: 96% *ee* (IC 250 × 4.6 mm, *n*-Hep/*iso*-PrOH = 50/50, 1 mL/min, λ = 210 nm); t_R = 16.81 min (minor, *ent*-**2n**), 22.00 min (major, **2n**).

(*R*)-7-Isobutoxy-6,7-dihydro-5*H*-pyrrolo[3,4-*b*]pyrazin-5-one (2o)

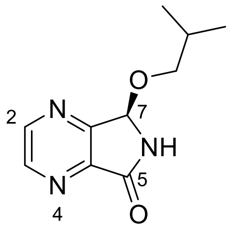

2o

$C_{10}H_{13}N_3O_2$

M = 207.23 g mol^{-1}

According to GP E, a degassed solution of *rac*-**2o** (5.18 mg, 25.0 µmol, 1.00 equiv.) and enantiomerically pure (+)-benzophenone **1b** (252 µg, 625 nmol, 2.5 mol%) in 10 mL α,α,α-trifluorotoluene was irradiated at λ = 366 nm for 8 h. After irradiation, the solvent was evaporated and the residue was purified by flash column chromatography (SiO_2, EtOAc) to obtain **2o** (4.46 mg, 20.5 µmol, 86%, 96% *ee*) as a colorless solid.

TLC (EtOAc): R_f = 0.43 [UV] [$KMnO_4$].

Optical Rotation: $[\alpha]_D^{25}$: +78 (*c* = 1.0, CH_2Cl_2) [96% *ee*].

Chiral HPLC: 96% *ee* (IC 250 × 4.6 mm, *n*-Hep/*iso*-PrOH = 50/50, 1 mL/min, λ = 210 nm); t_R = 9.91 min (minor, *ent*-**2o**), 16.68 min (major, **2o**).

(*R*)-7-(But-3-en-1-yloxy)-6,7-dihydro-5*H*-pyrrolo[3,4-*b*]pyrazin-5-one (2p)

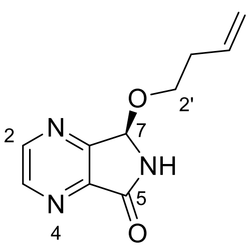

2p

$C_{10}H_{11}N_3O_2$

M = 205.22 g mol^{-1}

According to GP E, a degassed solution of *rac*-**2p** (5.13 mg, 25.0 µmol, 1.00 equiv.) and enantiomerically pure (+)-benzophenone **1b** (252 µg, 625 nmol, 2.5 mol%) in 10 mL α,α,α-trifluorotoluene was irradiated at λ = 366 nm for 8 h. After irradiation, the solvent was evaporated and the residue was purified by flash column chromatography (SiO_2, EtOAc) to obtain **2p** (5.02 mg, 24.5 µmol, 98%, 91% *ee*) as a colorless solid.

TLC (EtOAc): R_f = 0.58 [UV] [$KMnO_4$].

Optical Rotation: $[\alpha]_D^{25}$: +44 (*c* = 1.0, CH_2Cl_2) [91% *ee*].

Chiral HPLC: 91% *ee* (IC 250 × 4.6 mm, *n*-Hep/*iso*-PrOH = 50/50, 1 mL/min, λ = 210 nm); t_R = 12.66 min (minor, *ent*-**2p**), 19.42 min (major, **2p**).

(*R*)-7-Isopropoxy-6,7-dihydro-5*H*-pyrrolo[3,4-*b*]pyrazin-5-one (2q)

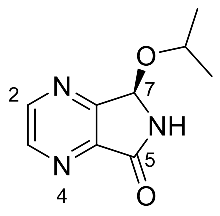

2q

$C_9H_{11}N_3O_2$

M = 193.21 g mol^{-1}

According to GP E, a degassed solution of *rac*-**2q** (4.83 mg, 25.0 µmol, 1.00 equiv.) and enantiomerically pure (+)-benzophenone **1b** (252 µg, 625 nmol, 2.5 mol%) in 10 mL α,α,α-trifluorotoluene was irradiated at λ = 366 nm for 8 h. After irradiation, the solvent was evaporated and the residue was purified by flash column chromatography (SiO_2, EtOAc) to obtain **2q** (3.56 mg, 18.4 µmol, 74%, 97% *ee*) as a colorless solid.

TLC (EtOAc): R_f = 0.45 [UV] [$KMnO_4$].

Optical Rotation: $[\alpha]_D^{25}$: +30 (*c* = 1.0, CH_2Cl_2) [97% *ee*].

Chiral HPLC: 97% *ee* (IC 250 × 4.6 mm, *n*-Hep/*iso*-PrOH = 50/50, 1 mL/min, λ = 210 nm); t_R = 14.32 min (minor, *ent*-**2q**), 22.22 min (major, **2q**).

(*R*)-7-(3-Methoxy-3-methylbutoxy)-6,7-dihydro-5*H*-pyrrolo[3,4-*b*]pyrazin-5-one (2r)

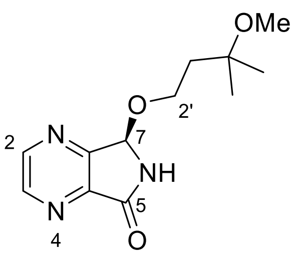

2r

$C_{12}H_{17}N_3O_3$

M = 251.29 g mol^{-1}

According to GP E, a degassed solution of *rac*-**2r** (6.28 mg, 25.0 µmol, 1.00 equiv.) and enantiomerically pure (+)-benzophenone **1b** (252 µg, 625 nmol, 2.5 mol%) in 10 mL α,α,α-trifluorotoluene was irradiated at λ = 366 nm for 8 h. After irradiation, the solvent was evaporated and the residue was purified by flash column chromatography (SiO_2, EtOAc) to obtain **2r** (4.78 mg, 19.0 µmol, 76%, 86% *ee*) as a colorless solid.

TLC (EtOAc): R_f = 0.24 [UV] [$KMnO_4$].

Optical Rotation: $[\alpha]_D^{25}$: +64 (*c* = 1.0, CH_2Cl_2) [86% *ee*].

Chiral HPLC: 86% *ee* (IC 250 × 4.6 mm, *n*-Hep/*iso*-PrOH = 50/50, 1 mL/min, λ = 210 nm); t_R = 21.66 min (major, **2r**), 23.34 min (minor, *ent*-**2r**).

(*R*)-7-(2,2,2-Trifluoroethoxy)-6,7-dihydro-5*H*-pyrrolo[3,4-*b*]pyrazin-5-one (2s)

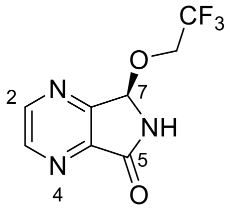

2s
$C_8H_6F_3N_3O_2$
M = 233.15 g mol^{-1}

According to GP E, a degassed solution of *rac*-**2s** (5.82 mg, 25.0 µmol, 1.00 equiv.) and enantiomerically pure (+)-benzophenone **1b** (252 µg, 625 nmol, 2.5 mol%) in 10 mL α,α,α-trifluorotoluene was irradiated at λ = 366 nm for 8 h. After irradiation, the solvent was evaporated and the residue was purified by flash column chromatography (SiO_2, EtOAc) to obtain **2s** (5.12 mg, 22.0 µmol, 88%, 92% *ee*) as a colorless solid.

TLC (EtOAc): R_f = 0.64 [UV] [$KMnO_4$].

Optical Rotation: $[\alpha]_D^{25}$: +54 (*c* = 1.0, CH_2Cl_2) [92% *ee*].

Chiral HPLC: 92% *ee* (IC 250 × 4.6 mm, *n*-Hep/*iso*-PrOH = 70/30, 1 mL/min, λ = 210 nm); t_R = 7.60 min (minor, *ent*-**2s**), 9.60 min (major, **2s**).

(*R*)-7-((*tert*-Butyldimethylsilyl)oxy)-6,7-dihydro-5*H*-pyrrolo[3,4-*b*]pyrazin-5-one (2t)

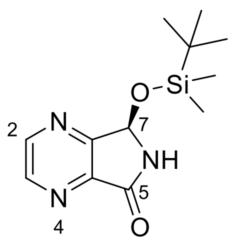

2t
$C_{12}H_{19}N_3O_2Si$
M = 265.39 g mol^{-1}

According to GP E, a degassed solution of *rac*-**2t** (6.63 mg, 25.0 µmol, 1.00 equiv.) and enantiomerically pure (+)-benzophenone **1b** (252 µg, 625 nmol, 2.5 mol%) in 10 mL α,α,α-trifluorotoluene was irradiated at λ = 366 nm for 8 h. After irradiation, the solvent was evaporated and the residue was purified by flash column chromatography (SiO_2, EtOAc) to obtain **2r** (5.59 mg, 21.1 µmol, 84%, 99% *ee*) as a colorless solid.

TLC (Pn:EtOAc = 1:2): R_f = 0.47 [UV] [$KMnO_4$].

Optical Rotation: $[\alpha]_D^{25}$: +48 (*c* = 1.0, CH_2Cl_2) [99% *ee*].

Chiral HPLC: 99% *ee* (IC 250 × 4.6 mm, *n*-Hep/*iso*-PrOH = 50/50, 1 mL/min, λ = 210 nm); t_R = 7.74 min (minor, *ent*-**2t**), 10.59 min (major, **2t**).

1 mmol scale:

According to GP E, a degassed solution of *rac*-**2t** (265 mg, 1.00 mmol, 1.00 equiv.) and enantiomerically pure (+)-benzophenone **1b** (10.1 mg, 25.0 µmol, 2.5 mol%) in 200 mL α,α,α-trifluorotoluene was irradiated at λ = 366 nm for 15 h. After irradiation, the solvent was evaporated and the residue was purified by flash column chromatography (SiO_2, EtOAc) to obtain **2t** (209 mg, 788 µmol, 79%, 98% *ee*) as a colorless solid.

TLC (Pn:EtOAc = 1:2): R_f = 0.47 [UV] [$KMnO_4$].

Optical Rotation: $[\alpha]_D^{25}$: +48 (c = 1.0, CH_2Cl_2) [98% *ee*].

Chiral HPLC: 98% *ee* (IC 250 × 4.6 mm, *n*-Hep/*iso*-PrOH = 50/50, 1 mL/min, λ = 210 nm); t_R = 7.72 min (minor, *ent*-**2t**), 10.55 min (major, **2t**).

(*R*)-7-((Triethylsilyl)oxy)-6,7-dihydro-5*H*-pyrrolo[3,4-*b*]pyrazin-5-one (2u)

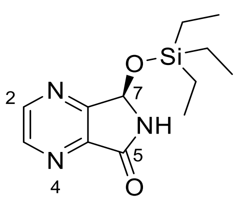

2u
$C_{12}H_{19}N_3O_2Si$
M = 265.39 g mol^{-1}

According to GP E, a degassed solution of *rac*-**2u** (6.63 mg, 25.0 µmol, 1.00 equiv.) and enantiomerically pure (+)-benzophenone **1b** (252 µg, 625 nmol, 2.5 mol%) in 10 mL α,α,α-trifluorotoluene was irradiated at λ = 366 nm for 8 h. After irradiation, the solvent was evaporated and the residue was purified by flash column chromatography (SiO_2, EtOAc) to obtain **2u** (6.24 mg, 23.5 µmol, 94%, 97% *ee*) as a colorless solid.

TLC (Pn:EtOAc = 1:1): R_f = 0.32 [UV] [$KMnO_4$].

Optical Rotation: $[\alpha]_D^{25}$: +62 (c = 1.0, CH_2Cl_2) [97% *ee*].

Chiral HPLC: 98% *ee* (IC 250 × 4.6 mm, *n*-Hep/*iso*-PrOH = 50/50, 1 mL/min, λ = 210 nm); t_R = 9.98 min (minor, *ent*-**2u**), 28.56 min (major, **2u**).

_(R)_-7-Phenyl-6,7-dihydro-5*H*-pyrrolo[3,4-*b*]pyrazin-5-one (*rac*-SI-7)

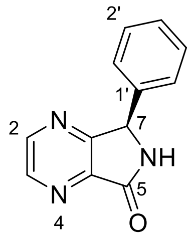

SI-7

$C_{12}H_9N_3O$

M = 211.22 g mol^{-1}

A degassed solution of *rac*-**SI-7** (5.28 mg, 25.0 µmol, 1.00 equiv.) and enantiomerically pure (+)-benzophenone **1b** (252 µg, 625 nmol, 2.5 mol%) in a mixture of 9 mL α,α,α-trifluorotoluene and 1 mL MeCN was irradiated at λ = 366 nm for 8 h. After irradiation, the solvent was evaporated and the residue was purified by flash column chromatography (SiO_2, EtOAc) to obtain **2u** (3.35 mg, 15.9 µmol, 63%, 94% *ee*) as a colorless solid.

TLC (EtOAc): R_f = 0.40 [UV] [$KMnO_4$].

Optical Rotation: $[\alpha]_D^{25}$: +90 (*c* = 1.0, CH_2Cl_2) [94% *ee*].

Chiral HPLC: 94% *ee* AD-H 250 × 4.6 mm, *n*-Hep/*iso*-PrOH = 70/30, 1 mL/min, λ = 210 nm); t_R = 6.99 min (major, **SI-7**), 8.46 min (minor, *ent*-**SI-7**).

13. Downstream Synthetic Transformations

***tert*-Butyl (*R*)-5-(cyclopentylmethyl)-7-oxo-5,7-dihydro-6*H*-pyrrolo[3,4-*b*]pyrazine-6-carboxylate (6)**

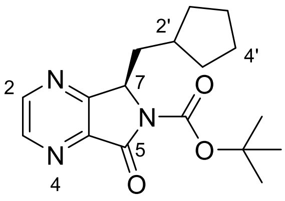

6
$C_{17}H_{23}N_3O_3$
M = 317.39 g mol^{-1}

According to a modified procedure,[77] to a solution of substrate **2l** (40.0 mg, 184 µmol, 98% *ee*, 1.00 equiv.) in CH_2Cl_2 (200 µL) was added *N,N*-Diisopropylethylamine (160 µL, 119 mg, 920 µmol, 5.00 equiv.), di-*tert*-butyl dicarbonate (213 µL, 217 mg, 994 µmol, 5.40 equiv.) followed by DMAP (11.3 mg, 92.1 µmol, 0.50 equiv.) at room temperature. The resulting mixture was stirred for 20 min at the same temperature. Afterwards, the reaction was quenched by addition of sat. NH_4Cl (1 mL) and distilled water (1 mL). The aqueous layer was extracted with CH_2Cl_2 (3 × 15 mL), and the combined organic layers were washed with brine (10 mL), dried over Na_2SO_4. After filtration, the solvent was removed under reduced pressure and the residual crude product was subjected to FCC (SiO_2, Pn:EtOAc = 2:1) to yield the desired product **6** (56 mg, 176 µmol, 96%, 98% *ee*) as a colorless oil which solidifies upon standing.

TLC (Pn:EtOAc = 2:1): R_f = 0.42 [UV] [$KMnO_4$].

M.p.: 102 °C.

^{1}H-NMR (400 MHz, $CDCl_3$, 300 K): δ [ppm] = 8.81 (d, ^3J = 2.4 Hz, 1H, H2), 8.75 (d, ^3J = 2.4 Hz, 1H, H3), 5.17 (dd, ^3J = 7.4 Hz, ^3J = 2.9 Hz, 1H, H7), 2.35 – 2.31 (m, 1H, H_a1'), 2.31 – 2.24 (m, 1H, H_a1'), 2.26 – 2.17 (m, 2H, H_a2', H_a3'/ H_a6'), 1.62 [s, 9H, $(CH_3)_3$], 1.61 – 1.50 (m, 2H, H_a4' , H_a5'), 1.49 – 1.35 (m, 3H, H_b4', H_b5', H_a6'/ H_a3'), 1.14 – 1.03 (m, 1H, H_b3'/ H_b6'), 0.99 – 0.89 (m, 1H, H_b6'/ H_b3').

^{13}C-NMR (101 MHz, $CDCl_3$, 300 K): δ [ppm] = 163.2 (C5), 160.0 (C7a), 149.8 (COO), 148.1 (C3), 146.8 (C2), 142.8 (C4a), 84.3 [$C(CH_3)_3$] 59.3 (C7), 37.5 (C1'), 35.3 (C2'), 33.8 (C3'/C6'), 32.9 (C6'/C3'), 28.3 [$C(CH_3)_3$], 25.1 (C4', C5').

HRMS (ESI) m/z: Calculated for [M+Na]$^+$: 340.1632; found: 340.1632.

IR (film): $\tilde{v}$max/cm^{-1} = 2943 (m, CH_{aliph}), 2866 (m, CH_{aliph}), 1777 (s, C=O), 1546 (m, C=Car), 1357 (s, CN), 1289 (s, CO).

Optical Rotation: $[\alpha]_D^{25}$: +26 (c = 1.0, CH_2Cl_2) [98% *ee*].

Chiral HPLC: 98% *ee* AD-H 250 × 4.6 mm, *n*-Hep/*iso*-PrOH = 90/10, 1 mL/min, λ = 210 nm); t_R = 14.29 min (minor, *ent*-**6**), 15.71 min (major, **6**).

(*R*)-3-(1-((*tert*-Butoxycarbonyl)amino)-2-cyclopentylethyl)pyrazine-2-carboxylic acid (7)

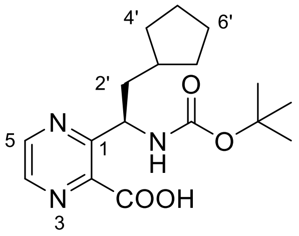

7

$C_{17}H_{25}N_3O_4$

M = 335.40 g mol^{-1}

According to a modified procedure,[78] to a solution of substrate **6** (45.0 mg, 142 µmol, 98% *ee*, 1.00 equiv.) in THF (707 µL) was added 1 M aqueous lithium hydroxide solution (8.48 mg, 354µL, 354 mmol, 2.50 equiv.) at room temperature. The resulting mixture was stirred vigorously for 10 min at the same temperature. Afterwards, the reaction was quenched by the addition of sat. NH_4Cl (1 mL). The pH value of the mixture was adjusted to 2-3 by addition of aqueous 1 M HCl and the mixture was extracted with CH_2Cl_2 (3 × 15 mL). The combined organic layers were washed with brine (10 mL) and dried over Na_2SO_4. After filtration, the solvent was removed under reduced pressure to yield the desired product **7** (44.9 mg, 134 µmol, 94%) as a colorless oil.

^{1}H-NMR (400 MHz, DMSO-d6, 300 K): δ [ppm] = 13.67 (bs, 1H, COOH), 8.75 (d, ^3J = 2.4 Hz, 1H, H5), 8.58 (d, ^3J = 2.4 Hz, 1H, H4), 7.15 (d, ^3J = 8.2 Hz, 1H, NH), 5.27 – 5.19 (m, 1H, H1'), 1.94 – 1.84 (m, 1H, H3'), 1.81 – 1.64 (m, 3H, H_a2', H_a4', H_a7'), 1.62 – 1.54 (m, 3H, H_b2',H_a5', H_b5'), 1.45 (m, 2H, H_a6', H_b6'), 1.31 [s, 9H, $(CH_3)_3$], 1.19 – 1.13 (m, 1H, H_b4'), 1.14 – 1.00 (m, 1H, H_b7').

^{13}C-NMR (101 MHz, DMSO-d6, 300 K): δ [ppm] = 166.7 (COOH), 157.0 (C1), 155.2 (NCOO), 145.8 (C5), 143.5 (C2), 142.0 (C4), 77.8 [*C*$(CH_3)_3$], 51.4 (C1'), 40.6 (C2'), 36.6 (C3'), 32.6 (C7'), 31.2 (C4'), 28.1 [C(*C*$H_3)_3$], 24.6 (C5'), 24.6 (C6').

HRMS (ESI) m/z: Calculated for $[M+H]^+$: 336.1918; found: 336.1919.

IR (film): $\tilde{v}$max/cm^{-1} = 3241 (bs, COOH), 2953 (m, CH_{aliph}), 2860 (m, CH_{aliph}), 1702 (s, C=O), 1529 (m, C=Car), 1366 (s, CN), 1159 (s, CO).

***tert*-Butyl (*R*)-(2-cyclopentyl-1-(pyrazin-2-yl)ethyl)carbamate (8)**

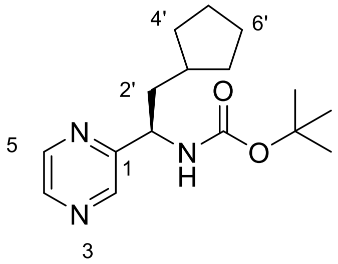

8

$C_{16}H_{25}N_3O_2$

M = 291.40 g mol^{-1}

According to a modified procedure,[79] a solution of substrate **7** (30.0 mg, 89.5 μmol, 1.00 equiv.) in *p*-xylene (330 μL) was refluxed for 60 min. Afterwards, the reaction mixture was allowed to cool to room temperature. The pH value of the mixture was adjusted to 11-12 by addition of 1 M NaOH and the mixture was extracted with CH_2Cl_2 (3 × 15 mL). The combined organic layers were washed with brine (10 mL) and dried over Na_2SO_4. After filtration, the solvent was removed under reduced pressure and the residual crude product was subjected to FCC (SiO_2, Pn:EtOAc = 4:1) to yield the desired product **8** (16.0 mg, 54.9 μmol, 61%, 96% *ee*) as a white solid.

TLC (Pn:EtOAc = 4:1): R_f = 0.23 [UV] [$KMnO_4$].

M.p.: 69 °C.

^{1}H-NMR (400 MHz, $CDCl_3$, 300 K): δ [ppm] = 8.55 (s, 1H, H2), 8.51 (dd, ^3J = 2.5, 1.5 Hz, 1H, H4), 8.46 (d, ^3J = 2.5 Hz, 1H, H5), 5.31 (bs, 1H, NH), 4.83 (m, 1H, H1’), 1.85 – 1.68 (m, 5H, H_a2’, H_b2’, H3’, H_a4’, H_a7’), 1.64 – 1.54 (m, 2H, H_a5’, H_b5’), 1.52 – 1.45 (m, 2H, H_a6’, H_b6’), 1.42 [s, 9H, $(CH_3)_3$], 1.22 – 1.04 (m, 2H, H_b4’, H_b7’).

^{13}C-NMR (101 MHz, $CDCl_3$, 300 K): δ [ppm] = 157.3 (C1), 155.4 (NCOO), 144.3 (C4), 143.7 (C2), 143.5 (C5), 79.8 [$C(CH_3)_3$], 53.2 (C1’), 42.8 (C2’), 36.8 (C3’), 33.0 (C4’/C7’)), 32.8 (C4’/C7’), 28.5 [$C(CH_3)_3$], 25.3 (C5’/C6’), 25.2 (C5’/C6’)).

HRMS (ESI) m/z: Calculated for $[M+H]^+$: 292.2020; found: 292.2018.

IR (film): $\tilde{v}$max/cm^{-1} = 3241 (bs, COOH), 2953 (m, CH_{aliph}), 2860 (m, CH_{aliph}), 1702 (s, C=O), 1529 (m, C=Car), 1364 (s, CN), 1162 (s, CO).

Optical Rotation: $[\alpha]_D^{25}$: +38 (*c* = 1.0, CH_2Cl_2) [96% *ee*].

Chiral HPLC: 96% *ee* AD-H 250 × 4.6 mm, *n*-Hep/*iso*-PrOH = 90/10, 1 mL/min, λ = 210 nm); t_R = 8.80 min (minor, *ent*-**8**), 11.23 min (major, **8**).

Methyl (*R*)-3-(1-((*tert*-butoxycarbonyl)amino)-2-cyclopentylethyl)pyrazine-2-carboxylate (9)

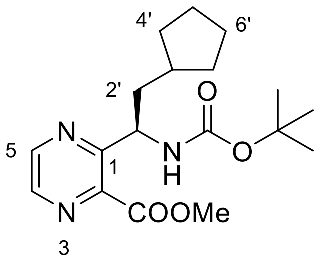

9

$C_{18}H_{27}N_3O_4$

M = 349.43 g mol^{-1}

According to a modified procedure,[80] to a solution of substrate **7** (15.0 mg, 44.7 µmol, 1.00 equiv.) in CH_2Cl_2:MeOH (5:1, 515 µL) was added a 2 M solution of (diazomethyl)trimethylsilane (10.2 mg, 44.7 µL, 89.5 µmol, 2.00 equiv.) in hexane at 0 °C. The resulting mixture was stirred for 1 h at the same temperature. Subsequently, the mixture was allowed to warm to room temperature and was stirred another 1 h at the same temperature. Afterwards, the reaction was quenched by addition of 1 drop acetic acid. The pH value of the mixture was adjusted to 7 by addition of a sat. bicarb solution and then was extracted with CH_2Cl_2 (3 × 15 mL). The combined organic layers were washed with brine (10 mL) and dried over Na_2SO_4. After filtration, the solvent was removed under reduced pressure and the residual crude product was subjected to FCC (SiO_2, Pn:EtOAc = 2:1) to yield the desired product **9** (15.1 mg, 43.2 µmol, 97%, 97% *ee*) as a white solid.

TLC (Pn:EtOAc = 2:1): R_f = 0.54 [UV] [$KMnO_4$].

M.p.: 122 °C.

^{1}H-NMR (400 MHz, $CDCl_3$, 300 K): δ [ppm] = 8.66 (d, ^3J = 2.3 Hz, 1H), 8.55 (d, ^3J = 2.3 Hz, 1H), 5.60 (s, 1H, H1'), 5.46 b(s, 1H, NH), 4.03 (s, 3H, CH_3), 2.00 – 1.86 (m, 2H, H3', H_a4'), 1.83 – 1.73 (m, 3H, H_a2', H_b2', H_a7'), 1.68 – 1.47 (m, 4H, H_a5', H_b5',H_a6', H_b6'), 1.40 (s, 9H), 1.37 – 1.23 (m, 1H, H_b4'), 1.19 – 1.05 (m, 1H, H_b7').

^{13}C-NMR (101 MHz, $CDCl_3$, 300 K): δ [ppm] = 165.3 (COO), 158.8 (C1), 155.4 (NCOO), 146.3 (C5), 142.3 (C2, C4), 79.5 [*C*(CH_3)$_3$], 53.3 (CH_3), 51.9 (C1'), 43.1 (C2'), 37.2 (C3'), 33.3 (C7'), 32.3 (C4'), 28.5 [C(*C*H_3)$_3$, 25.3 (C4'), 25.2 (C5').

HRMS (ESI) m/z: Calculated for $[M+H]^+$: 350.2074; found: 350.2073.

IR (film): $\tilde{v}$max/cm^{-1} = 3255 (bs, COO), 2954 (m, CH_{aliph}), 2853 (m, CH_{aliph}), 1745 (s, C=O), 1703 (s, C=O), 1531 (m, $C=C_{arom}$), 1162 (s, CN), 1021 (s, CO).

Optical Rotation: $[\alpha]_D^{25}$: +38 (c = 1.0, CH_2Cl_2) [97% *ee*].

Chiral HPLC: 97% *ee* IC 250 × 4.6 mm, *n*-Hep/*iso*-PrOH = 70/30, 1 mL/min, λ = 210 nm); t_R = 6.79 min (major, **9**), 18.74 min (minor, *ent*-**9**).

(*R*)-6,7-Dihydrooxazolo[3',2':1,2]pyrrolo[3,4-*b*]pyrazin-9(4*bH*)-one (10)

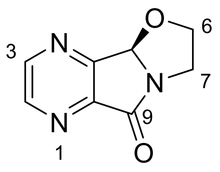

10
$C_8H_7N_3O_2$
M = 177.16 g mol^{-1}

According to a modified procedure,[3] to a solution of substrate **2n** (17.5 mg, 81.9 µmol, 96% *ee*, 1.00 equiv.) in PhH (1.6 mL) was added *N*-Methyl-*N*,*N*-dioctyl-1-octanaminiumchlorid (3.31 mg, 8.19 µmol, 0.10 equiv.) and sodium hydroxide (41.0 mg, 1.02 mmol, 12.5 equiv.) at room temperature. The resulting mixture was stirred for 30 min at the same temperature. Subsequently, CH_2Cl_2 (2 mL) was added, and the mixture was filtrated. The resulting filtrate was dried over Na_2SO_4. After filtration, the solvent was removed under reduced pressure and the residual crude product was subjected to FCC (SiO_2, EtOAc) to yield the desired product **10** (13.1 mg, 73.9 µmol, 90%, 96% *ee*) as a white solid.

TLC (EtOAc): R_f = 0.42 [UV] [$KMnO_4$].

M.p.: 137 °C.

^{1}H-NMR (400 MHz, $CDCl_3$, 300 K): δ [ppm] = 8.85 (d, ^3J = 2.6 Hz, 1H, H3), 8.73 (d, ^3J = 2.6 Hz, 1H, H2), 5.86 (s, 1H, H4b), 4.41 (ddd, ^2J = 8.7 Hz, ^3J = 7.7 Hz, ^3J = 5.2 Hz, 1H, H_a5), 4.34 (ddd, ^2J = 8.7, ^3J = 7.7 Hz, ^3J = 6.6 Hz, 1H, H_b5), 4.14 (ddd, ^2J = 11.0 Hz, ^3J = 7.7 Hz, ^3J = 6.6 Hz, 1H, H_a6), 3.57 (ddd, ^2J = 11.0 Hz, ^3J = 7.7 Hz, ^3J = 5.2 Hz, 1H, H_b6).

^{13}C-NMR (101 MHz, $CDCl_3$, 300 K): δ [ppm] = 169.2 (C9), 156.8 (C4a), 148.0 (C3), 147.3 (C2), 146.2 (C9a), 89.0 (C4b), 71.0 (C6), 43.0 (C7).

HRMS (ESI) m/z: Calculated for $[M+H]^+$: 178.0611; found: 178.0612.

IR (film): $\tilde{\nu}$max/cm^{-1} = 3066 (m, CH_{arom}), 2923 (m, CH_{aliph}), 1721 (s, C=O), 1382 (s, CN), 1053 (s, CO).

Optical Rotation: $[\alpha]_D^{25}$: +26 (c = 1.0, CH_2Cl_2) [96% *ee*].

Chiral HPLC: 96% *ee* AD-H 250 × 4.6 mm, *n*-Hep/*iso*-PrOH = 90/10, 1 mL/min, λ = 210 nm); t_R = 15.63 min (major, **10**), 11.23 min (minor, *ent*-**10**).

(*R*)-7-((*tert*-Butyldimethylsilyl)oxy)-6-(5-chloropyridin-2-yl)-6,7-dihydro-5*H*-pyrrolo[3,4-*b*]pyrazin-5-one (11)

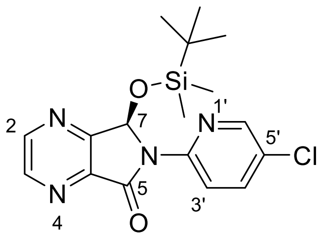

11
$C_{17}H_{21}ClN_4O_2Si$
M = 376.92 g mol^{-1}

According to a modified procedure,[81] to a solution of substrate **2t** (30.0 mg, 113 µmol, 98% *ee*, 1.00 equiv.), copper(I) iodide (10.7 mg, 56.5 µmol, 0.50 equiv.), K_3PO_4 (47.9 mg, 226 µmol, 2.00 equiv.) and 5-chloro-2-iodopyridine (29.7 mg, 124 µmol, 1.10 equiv.) in $PhCH_3$ (754 µL) was added *N,N'*-Dimethylethane-1,2-diamine (3.98 mg, 4.87 µL, 45.2 µmol, 0.40 equiv.).The resulting mixture was heated to 60 °C for 50 min. Afterwards, the mixture was allowed to cool to room temperature. Subsequently, the solvent was removed, and the residue was subjected to FCC (SiO_2, Pn:EtOAc = 6:1 → Pn:EtOAc = 2:1) to yield the desired product **11** (28.0 mg, 74.3 µmol, 66%, 97% *ee*) as a white solid.

TLC (Pn:EtOAc = 6:1): R_f = 0.24 [UV] [$KMnO_4$].

M.p.: >260°C.

^{1}H-NMR (400 MHz, $CDCl_3$, 300 K): δ [ppm] = 8.83 (d, ^3J = 2.6 Hz, 1H, H2), 8.77 (d, ^3J = 2.6 Hz, 1H, H3), 8.42 (dd, ^3J = 2.6 Hz, ^5J = 0.7 Hz, 1H, H3’), 8.35 (dd, ^4J = 8.8 Hz, ^5J = 0.7 Hz, 1H, H6’), 7.78 (dd, ^4J = 8.8 Hz, ^3J = 2.6 Hz, 1H, H4’), 7.05 (s, 1H, H7), 0.78 [s, 9H, $C(CH_3)_3$], 0.34 (s, 3H, Si-CH_3CH$_3$), 0.11 (s, 3H, Si-CH$_3$CH_3).

^{13}C-NMR (101 MHz, $CDCl_3$, 300 K): δ [ppm] = 163.0 (C5), 157.9 (C4a), 148.0 (C2’), 147.9 (C3), 147.3 (C2), 146.6 (C3’), 143.7 (C7a), 138.0 (C4’), 128.4 (C5’), 117.8 (C6’), 80.0 (C7), 25.7 [C(CH$_3$)$_3$], 18.3 [C(CH$_3$)$_3$], –4.2 (Si-CH$_3$CH$_3$), –4.4 (Si-CH$_3$$CH_3$).

HRMS (ESI) m/z: Calculated for $[M+H]^+$: 377.1195; found: 377.1192.

Optical Rotation: $[\alpha]_D^{25}$: +22 (c = 1.0, CH_2Cl_2) [97% *ee*].

Chiral HPLC: 97% *ee* IC 250 × 4.6 mm, *n*-Hep/*iso*-PrOH = 90/10, 1 mL/min, λ = 210 nm); t_R = 27.63 min (major, **11**), 32.22 min (minor, *ent*-**11**).

14. NMR Spectra

(2-Hydroxy-3-nitrophenyl)(phenyl)methanone (SI-1)

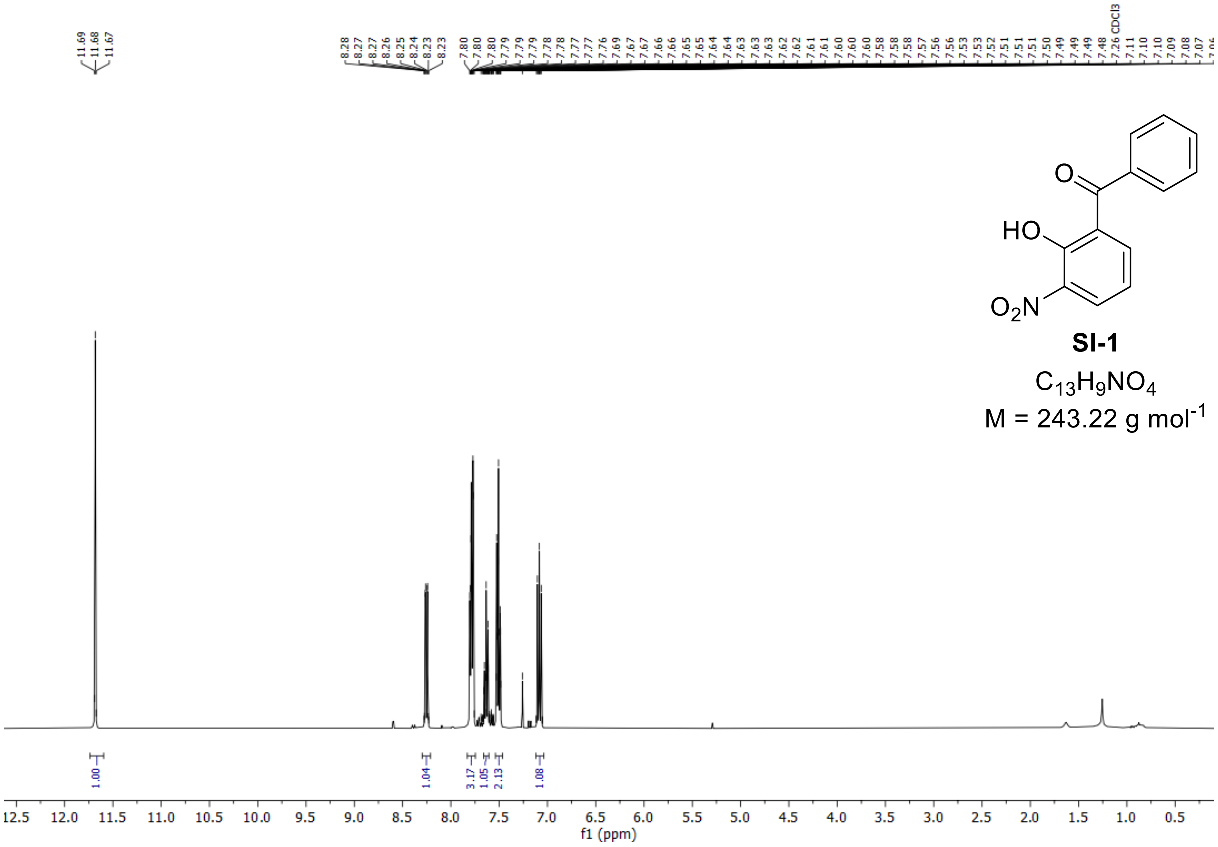

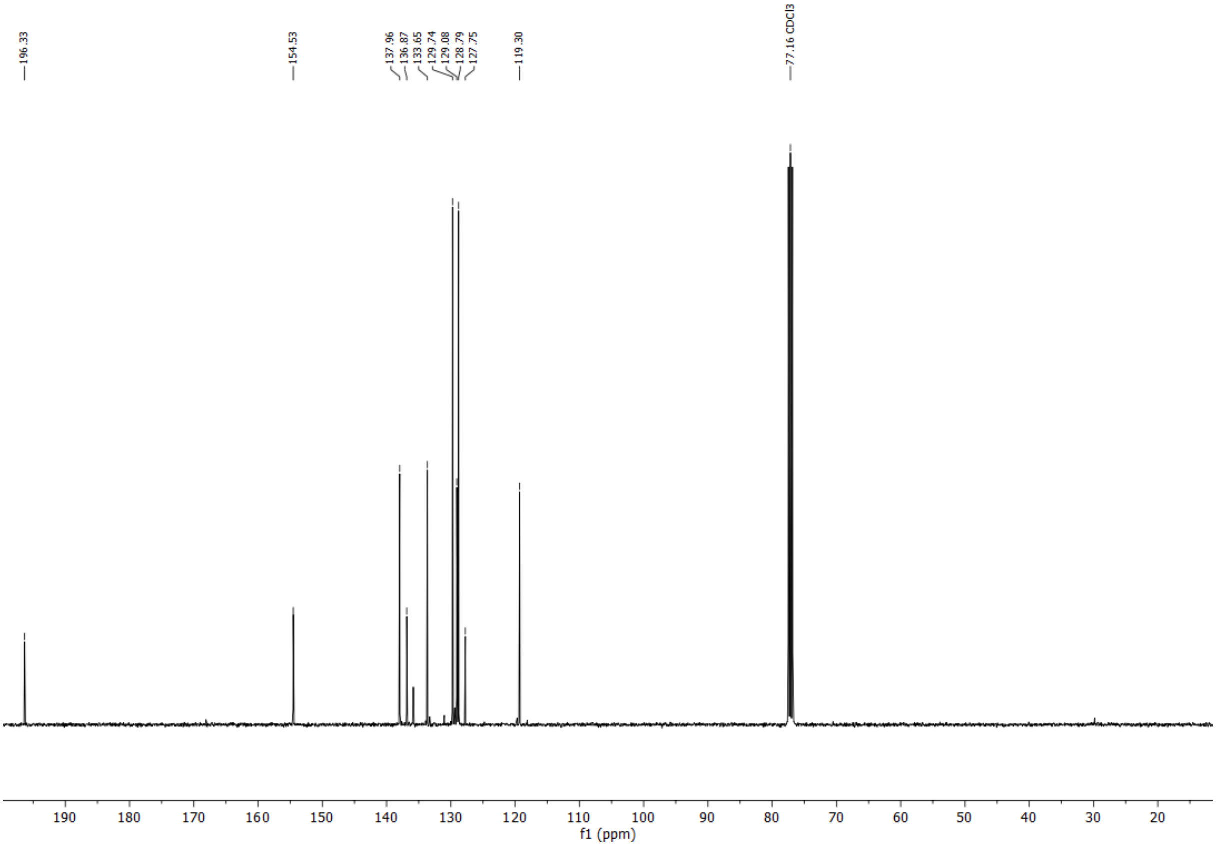

(3-Amino-2-hydroxyphenyl)(phenyl)methanone (SI-2)

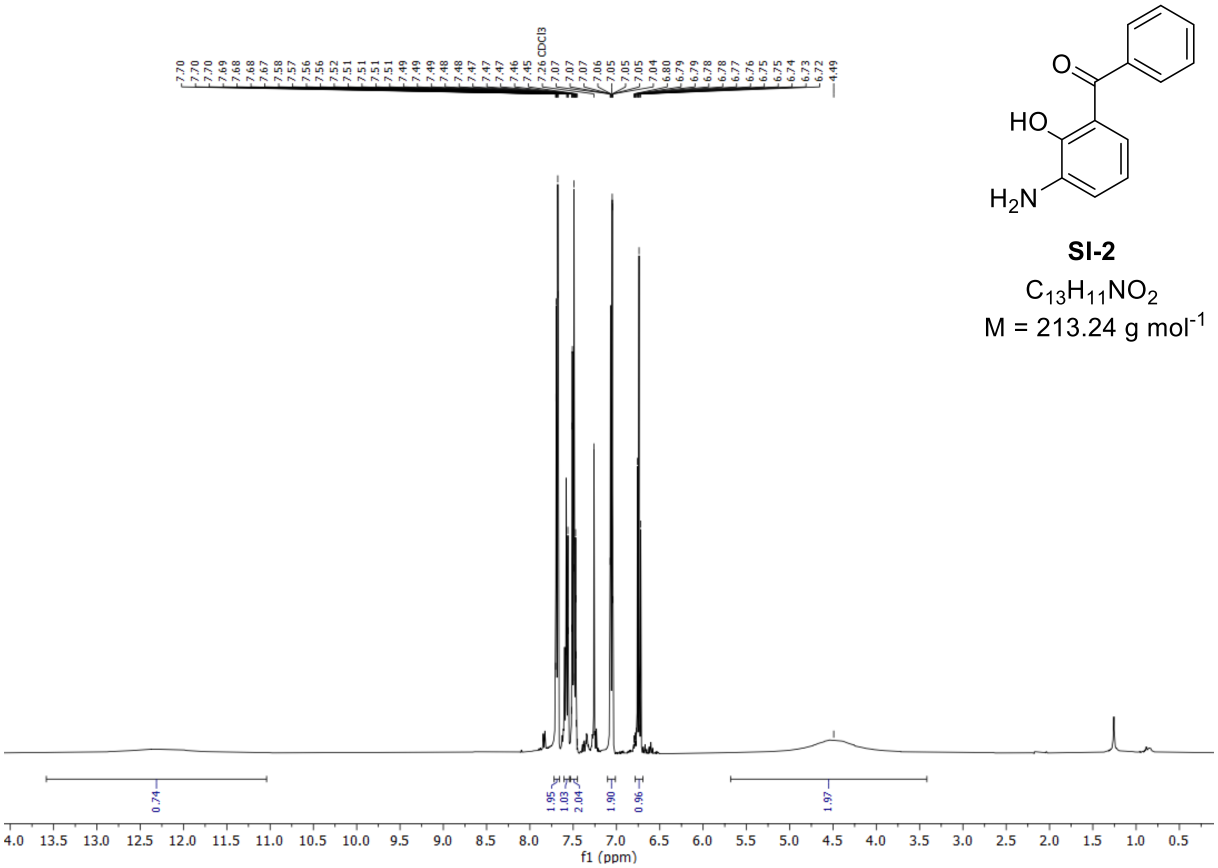

SI-2
$C_{13}H_{11}NO_2$
M = 213.24 g mol^{-1}

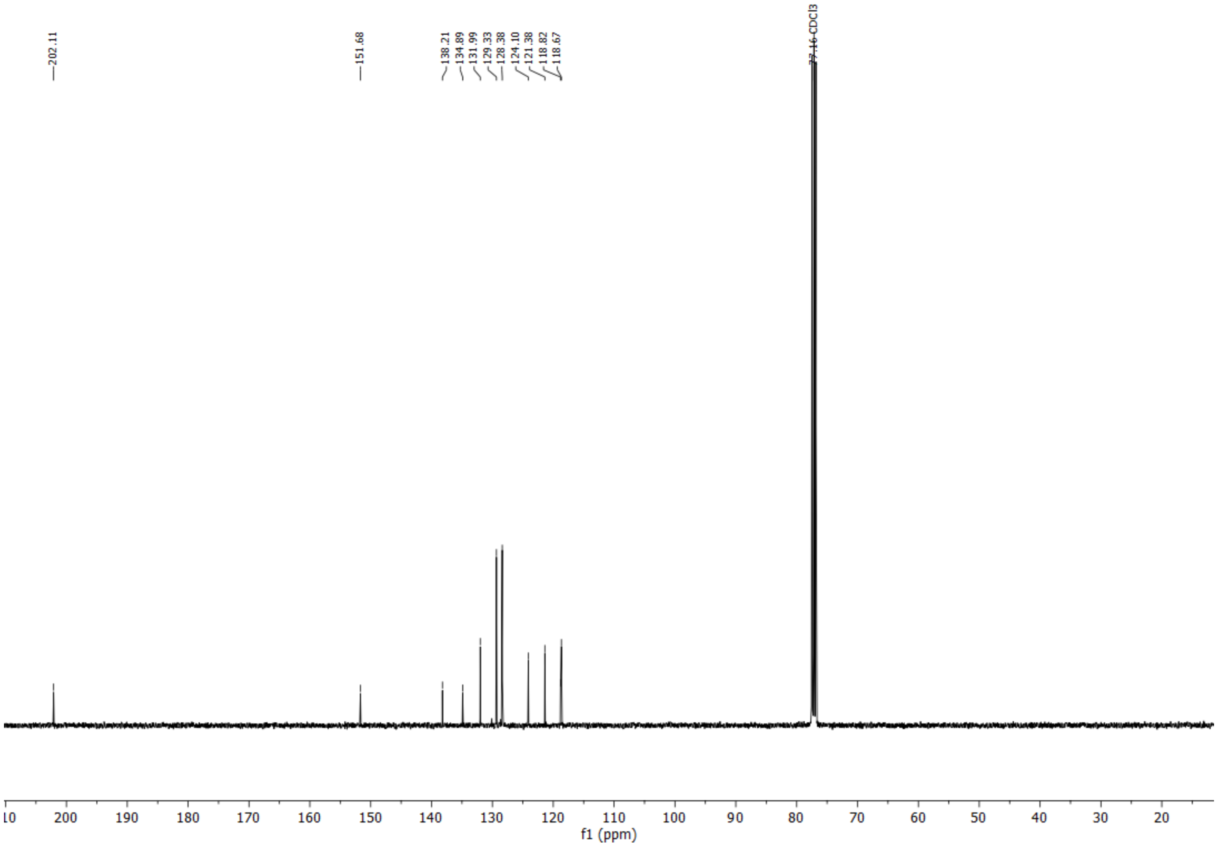

2-Amino-6-benzoylphenyl (1*SR*,5*RS*,7*RS*)-1,5,7-trimethyl-2-oxo-3-azabicyclo[3.3.1]nonane-7-carboxylate (*rac*-SI-4)

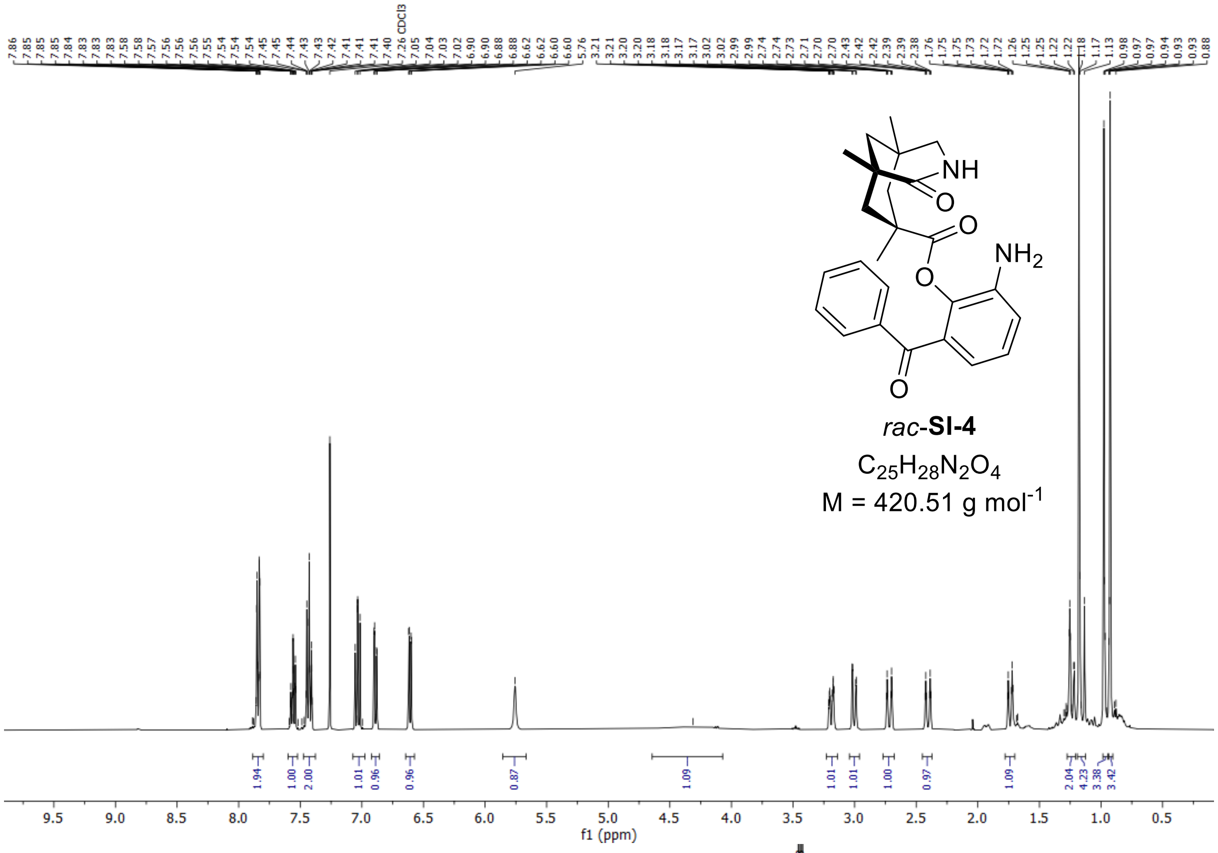

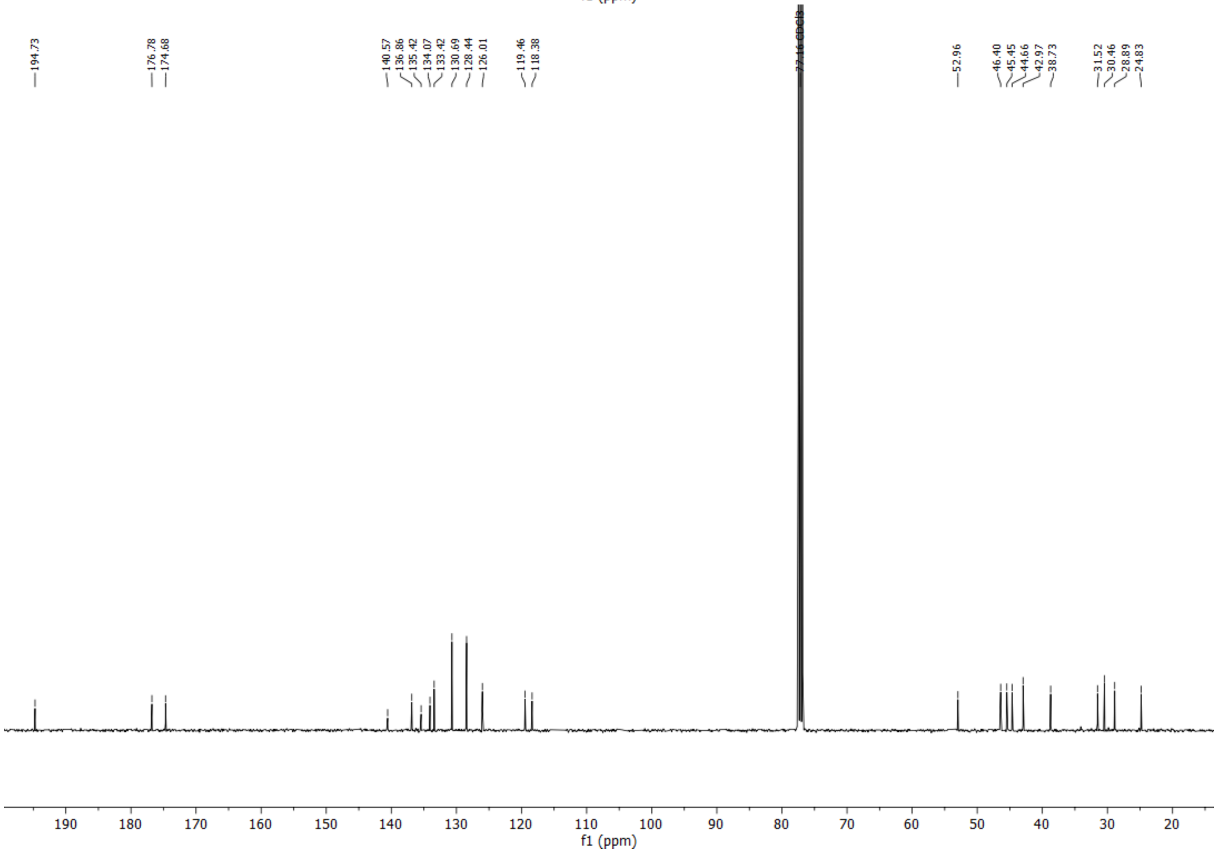

(1*SR*,5*SR*,7*RS*)-7-(7-Benzoylbenzo[d]oxazol-2-yl)-1,5,7-trimethyl-3-azabicyclo[3.3.1]nonan-2-one (*rac*-1b)

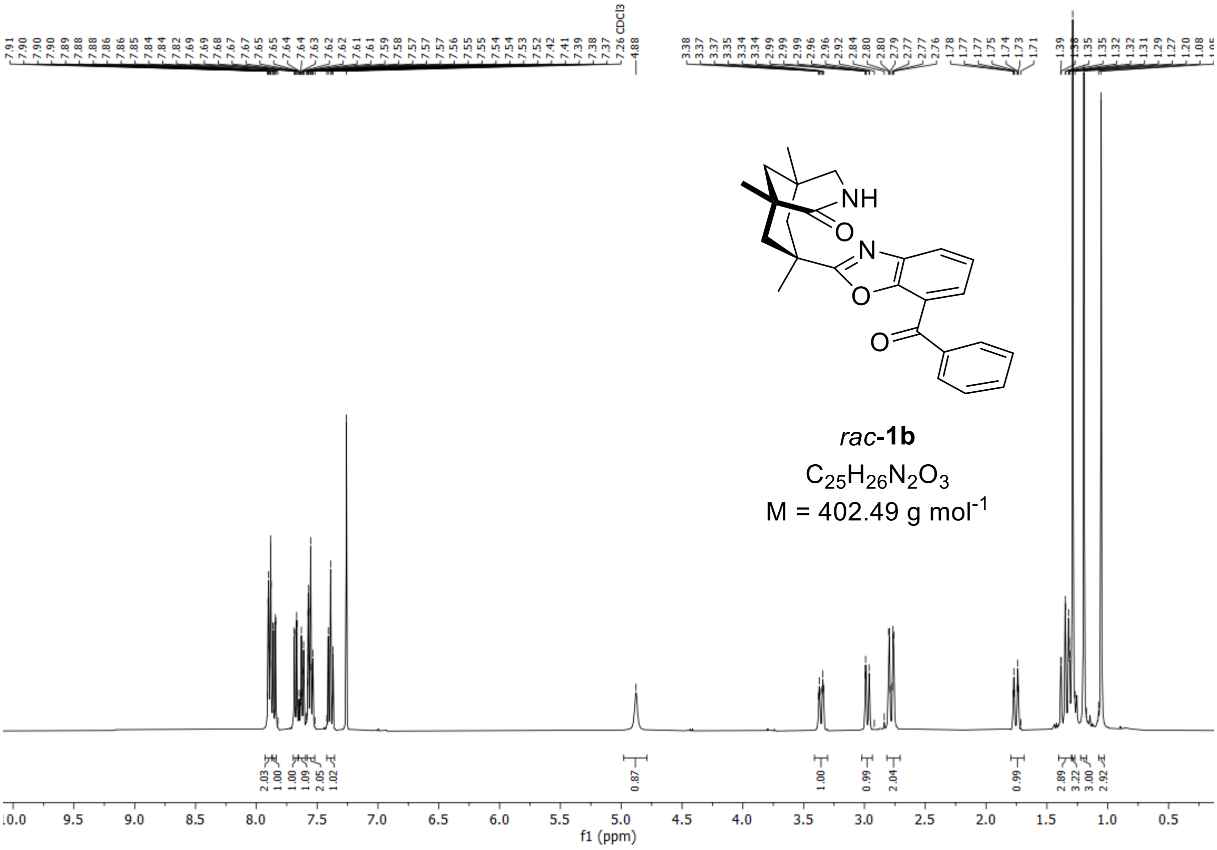

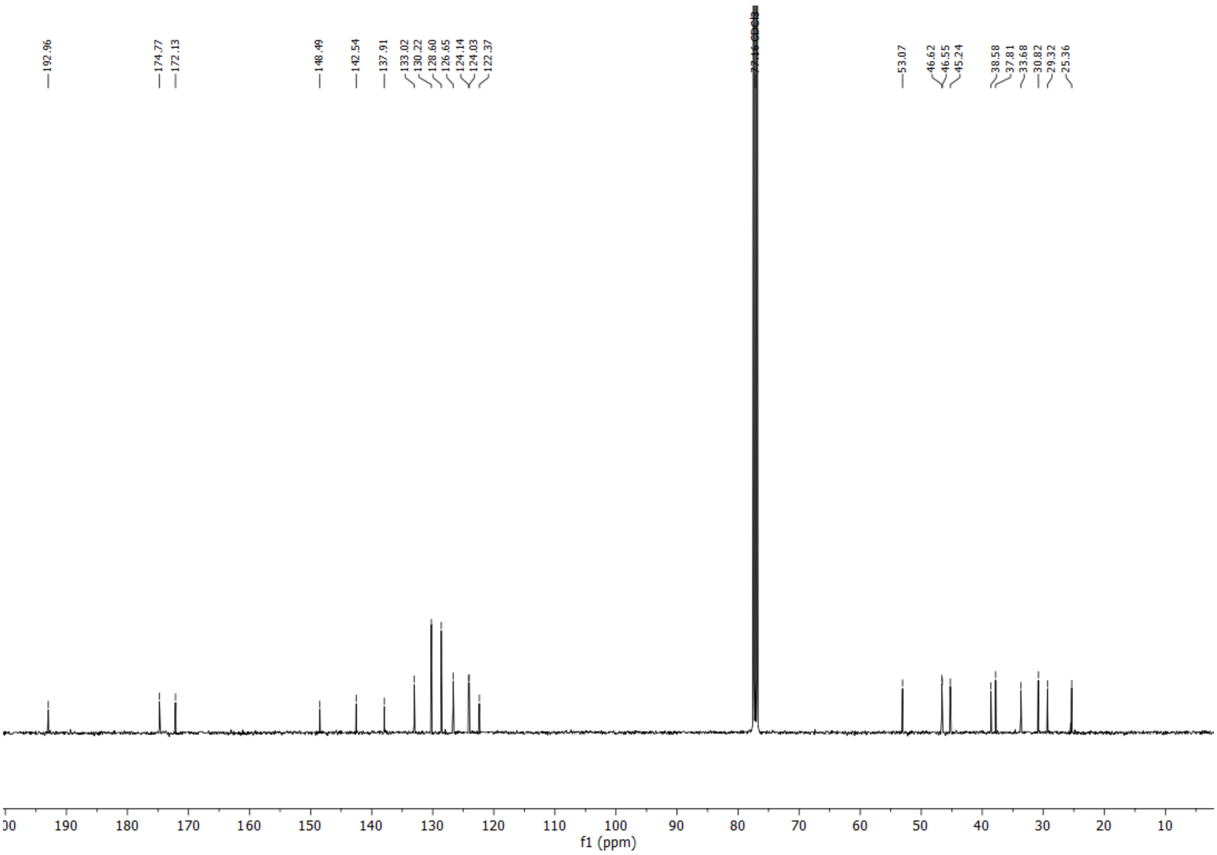

Methyl 3-(bromomethyl)pyrazine-2-carboxylate (SI-8)

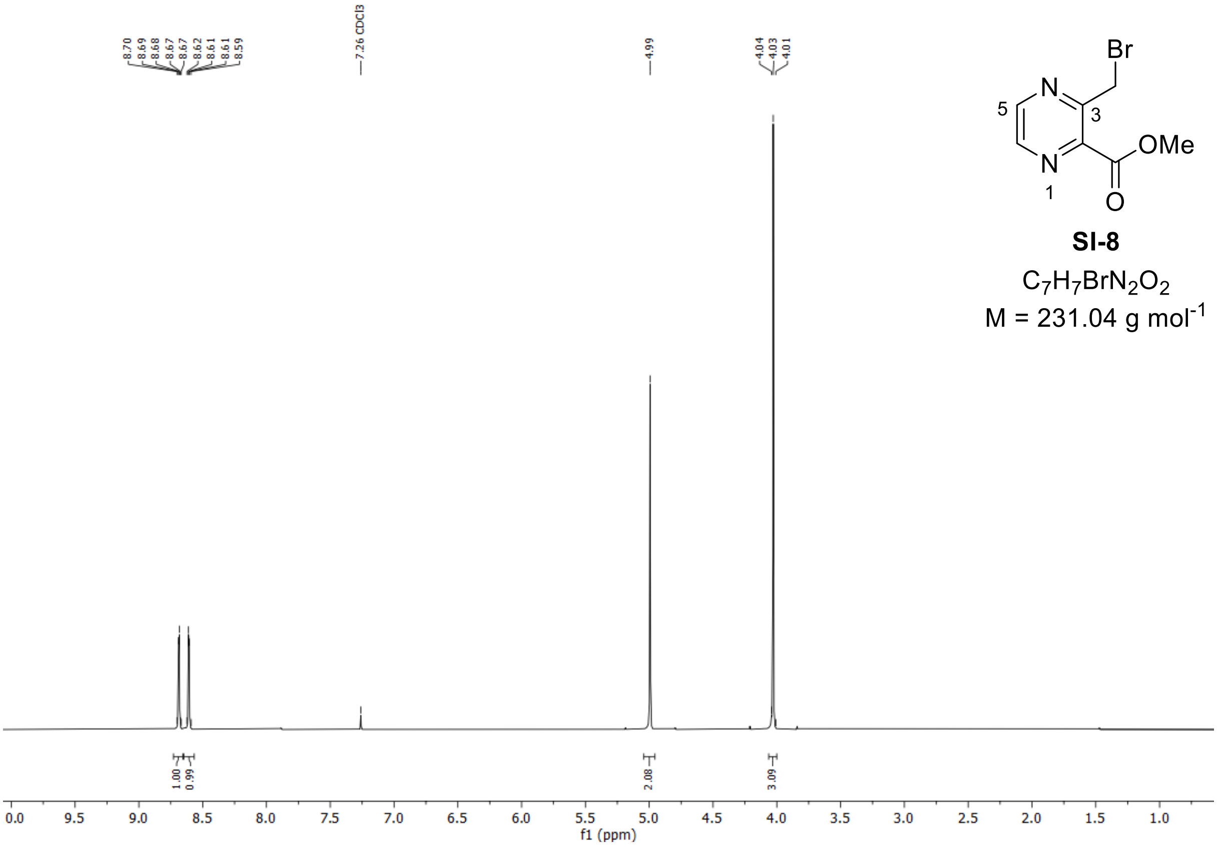

SI-8

$C_7H_7BrN_2O_2$

M = 231.04 g mol^{-1}

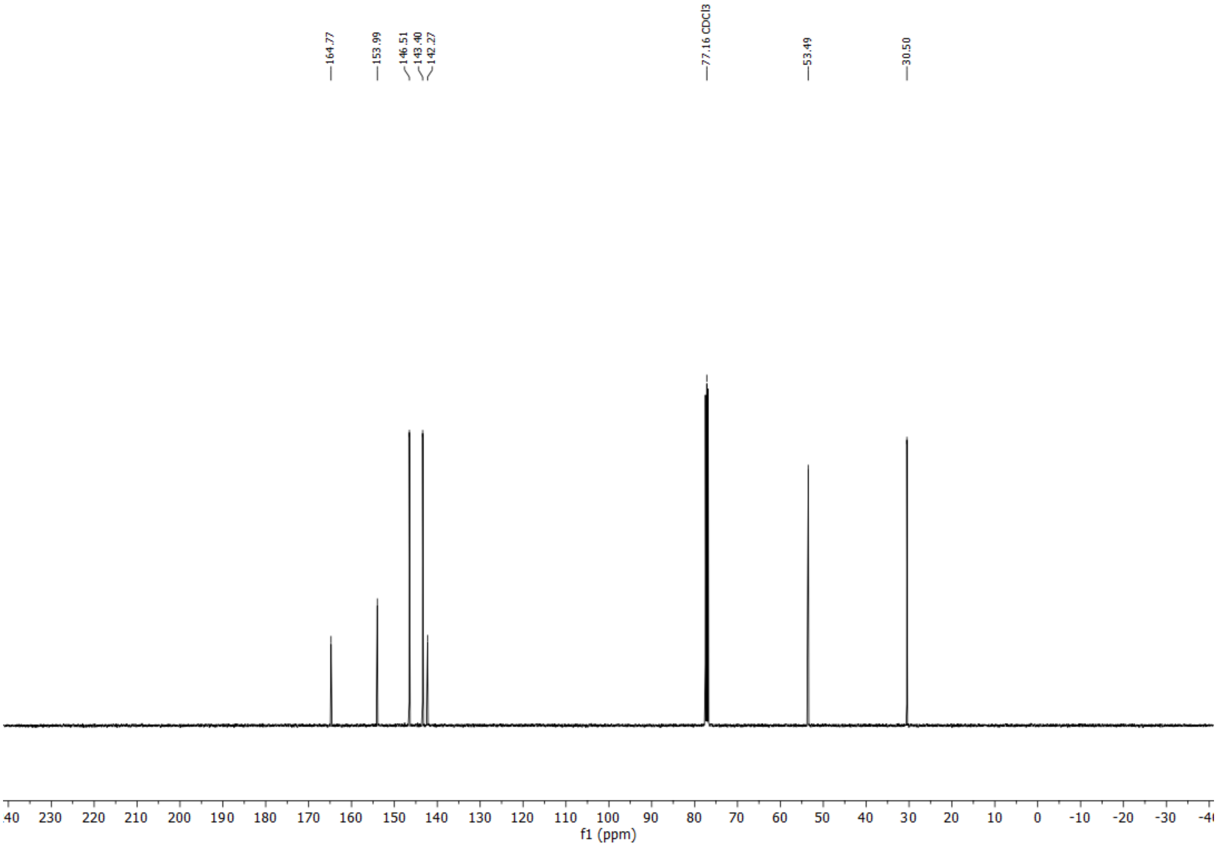

6-(4-Methoxybenzyl)-6,7-dihydro-5*H*-pyrrolo[3,4-*b*]pyrazin-5-one (SI-9)

SI-9

$C_{14}H_{13}N_3O_2$

M = 255.27 g mol^{-1}

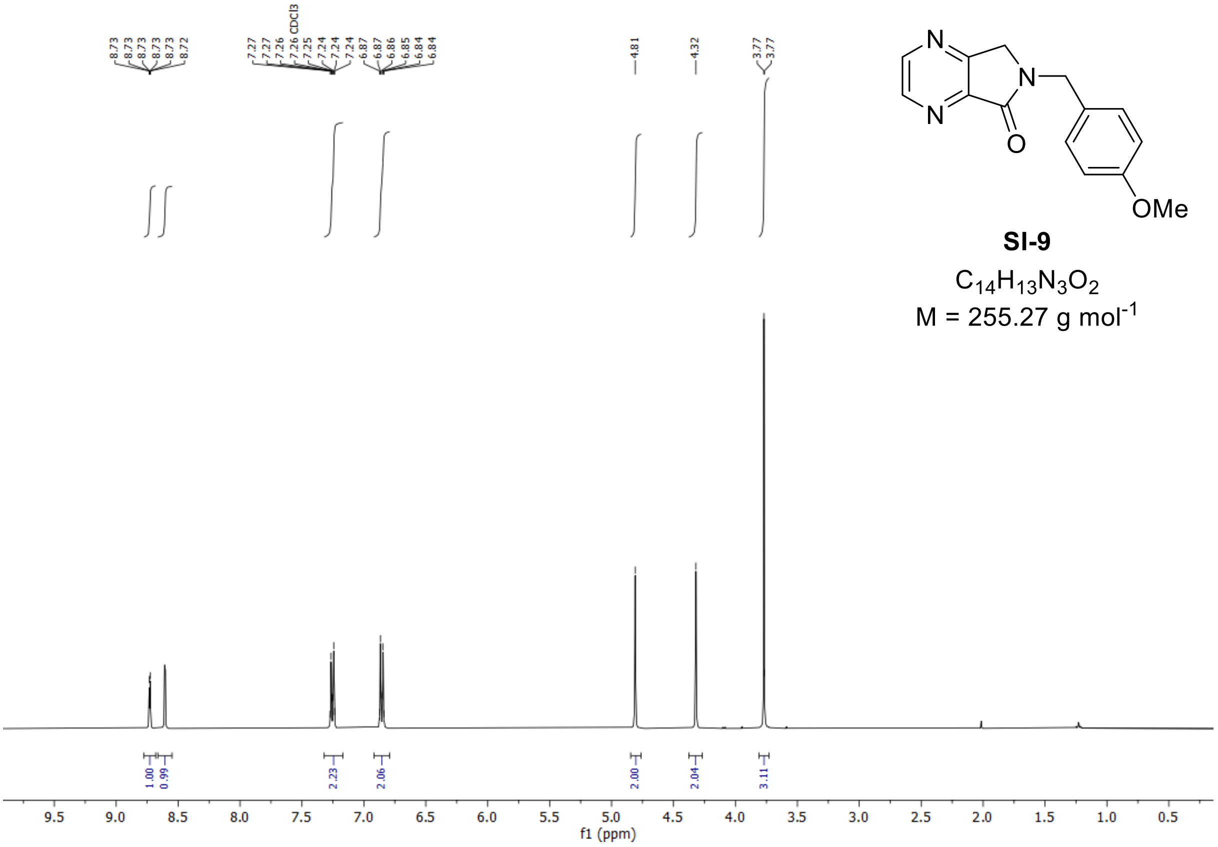

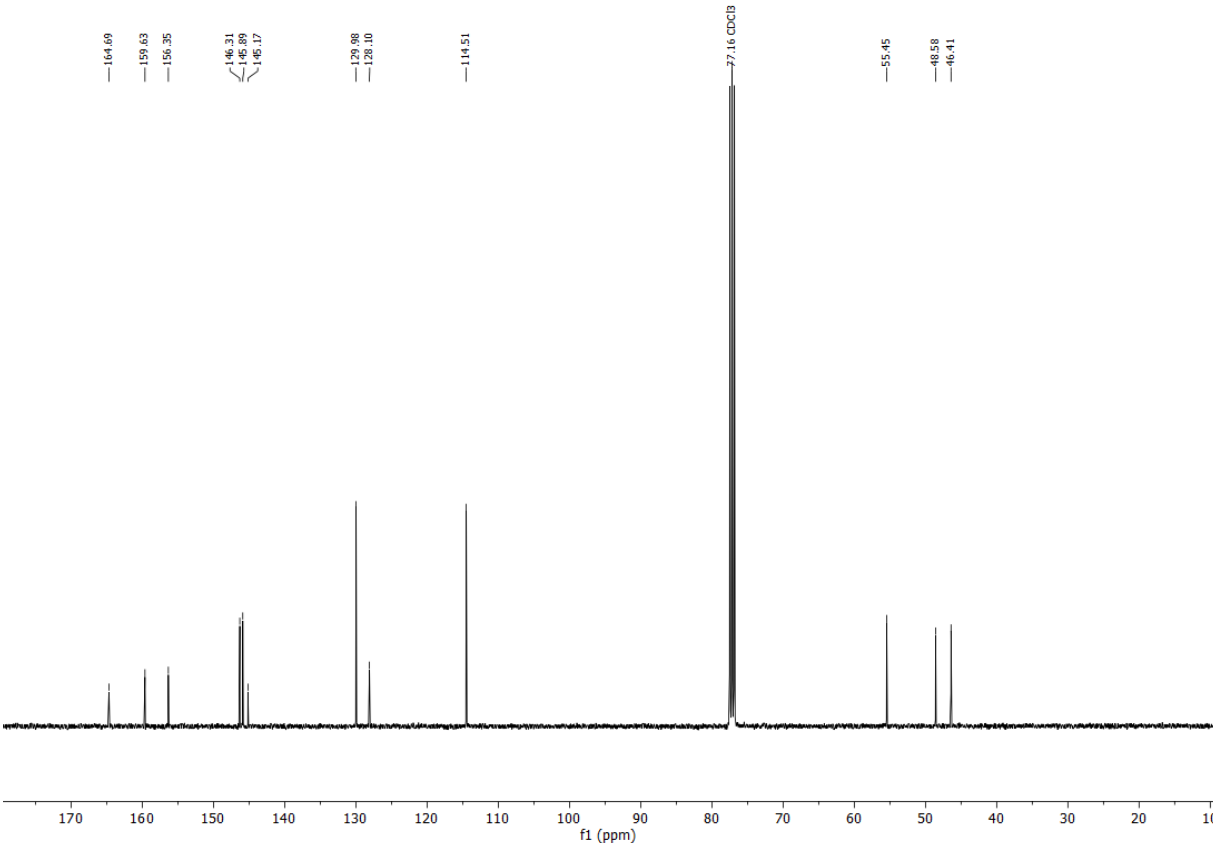

7-(4-(*tert*-Butyl)benzyl)-6,7-dihydro-5*H*-pyrrolo[3,4-*b*]pyrazin-5-one (*rac*-2a)

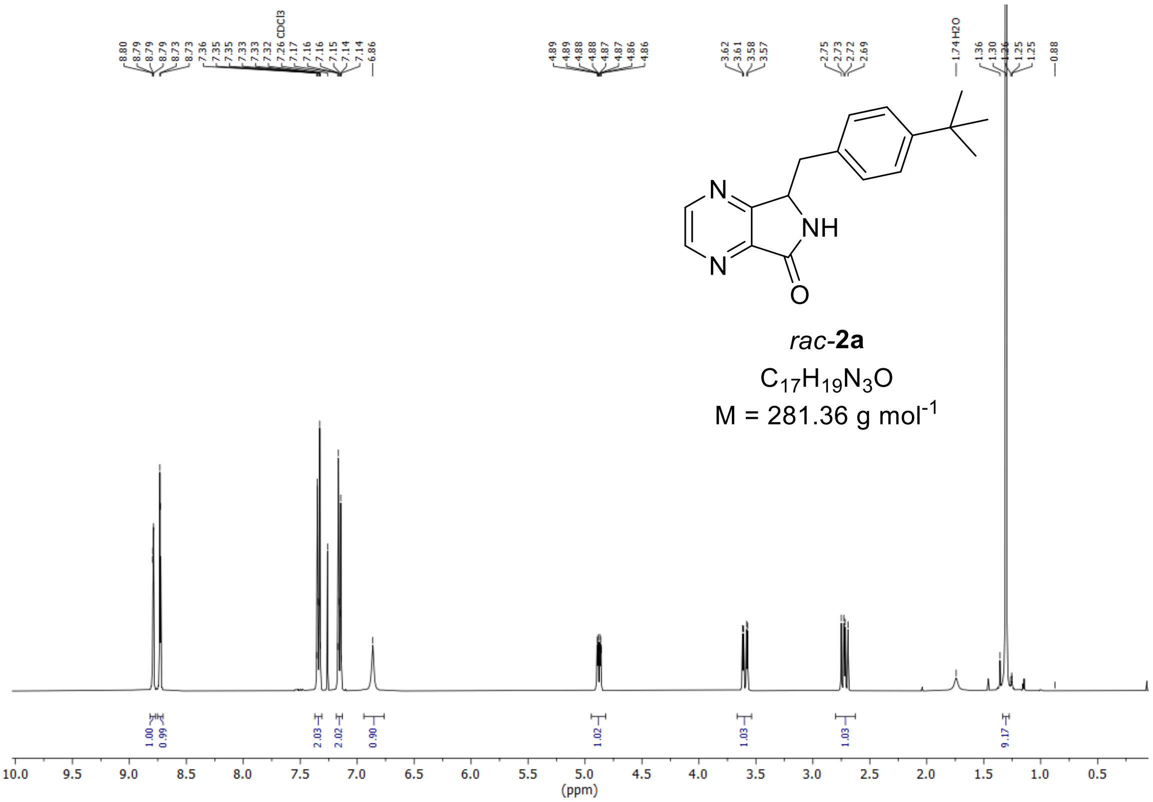

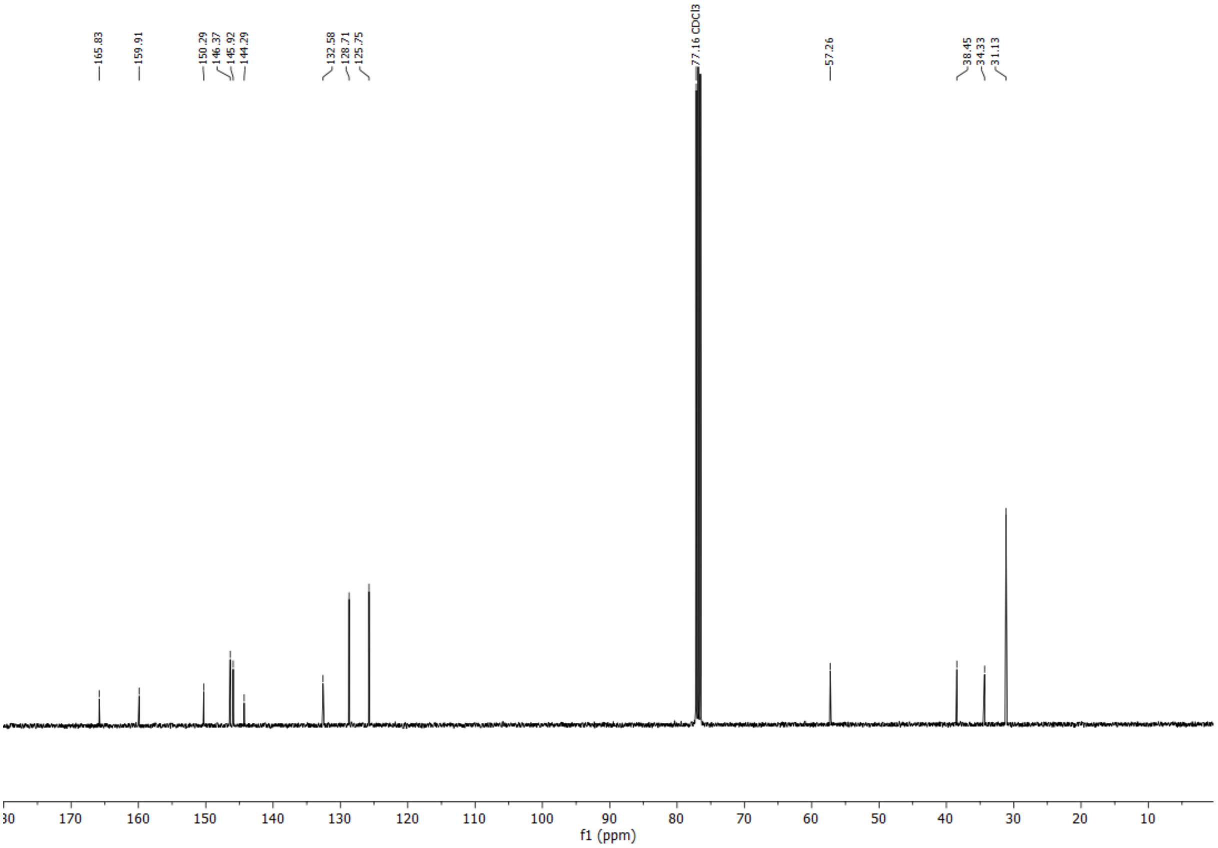

7-Benzyl-6,7-dihydro-5*H*-pyrrolo[3,4-*b*]pyrazin-5-one (*rac*-2b)

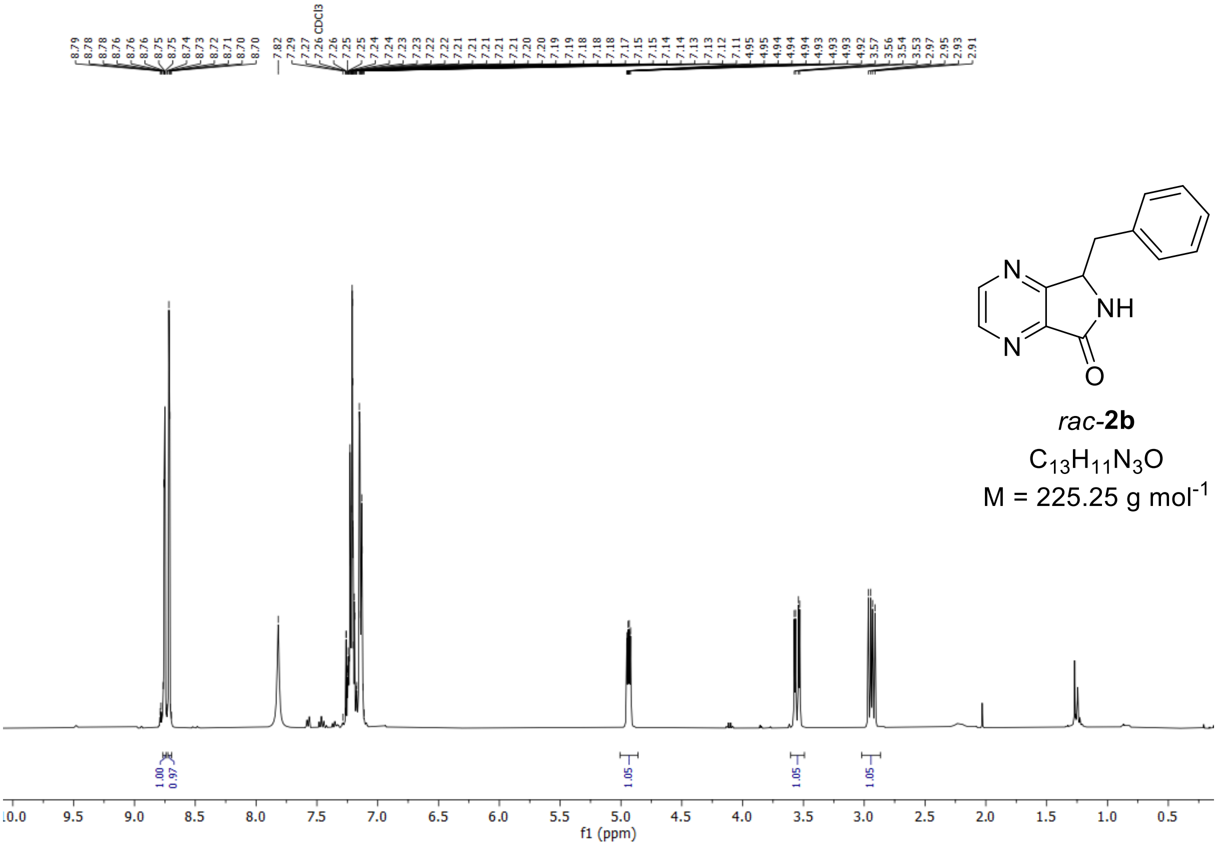

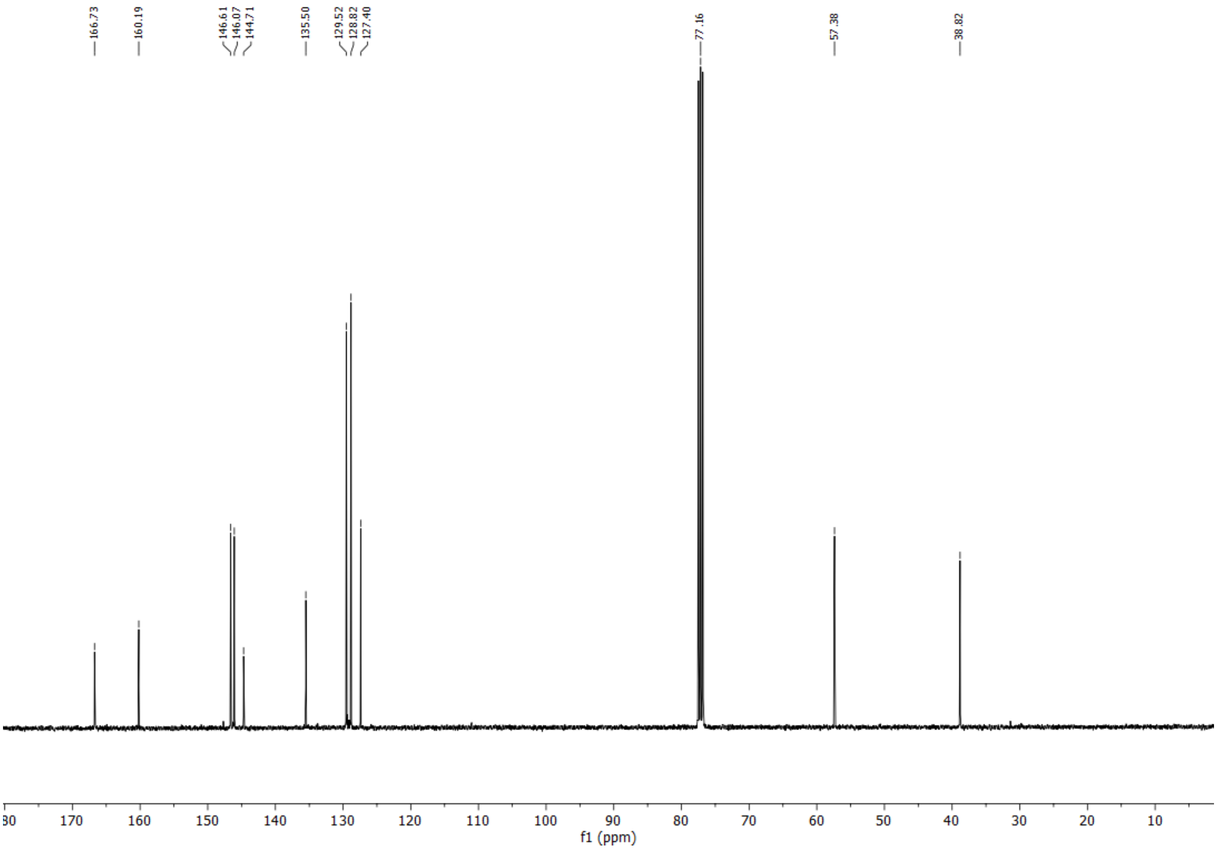

7-(4-(Trifluoromethyl)benzyl)-6,7-dihydro-5*H*-pyrrolo[3,4-*b*]pyrazin-5-one (*rac*-2c)

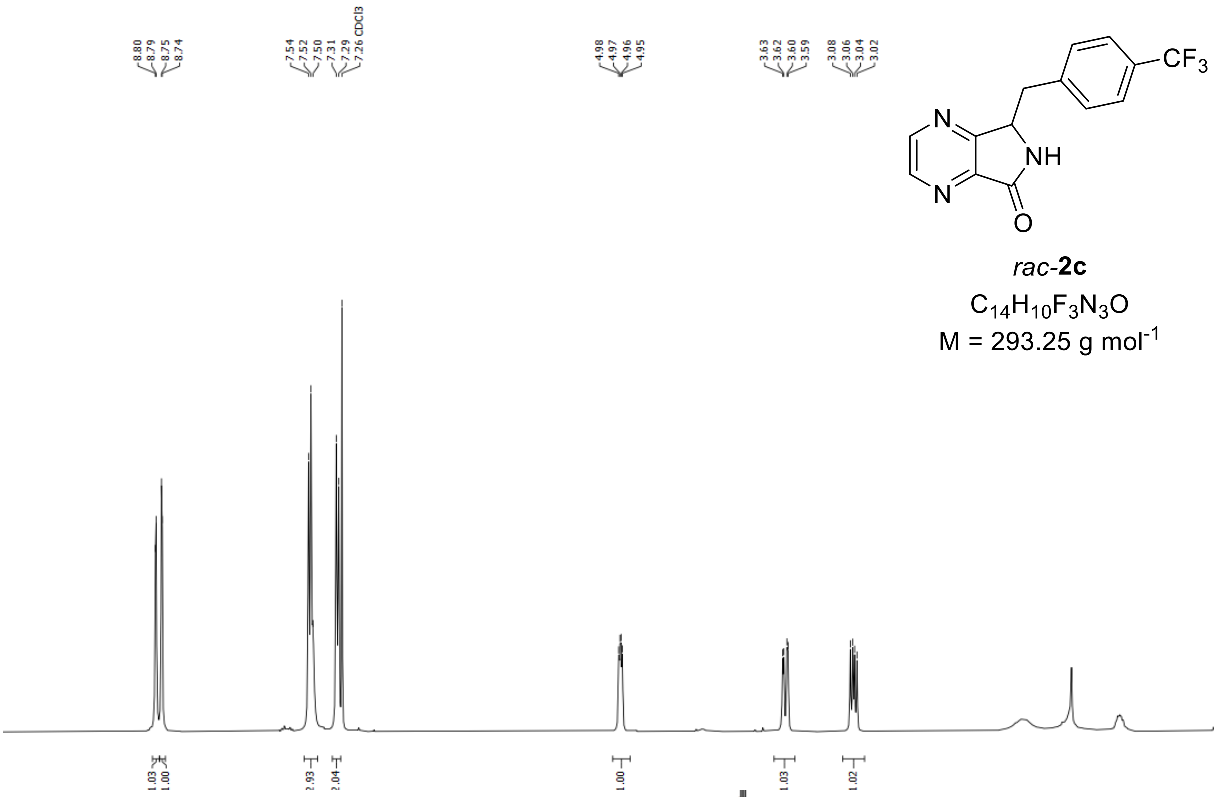

rac-**2c**

$C_{14}H_{10}F_3N_3O$

M = 293.25 g mol^{-1}

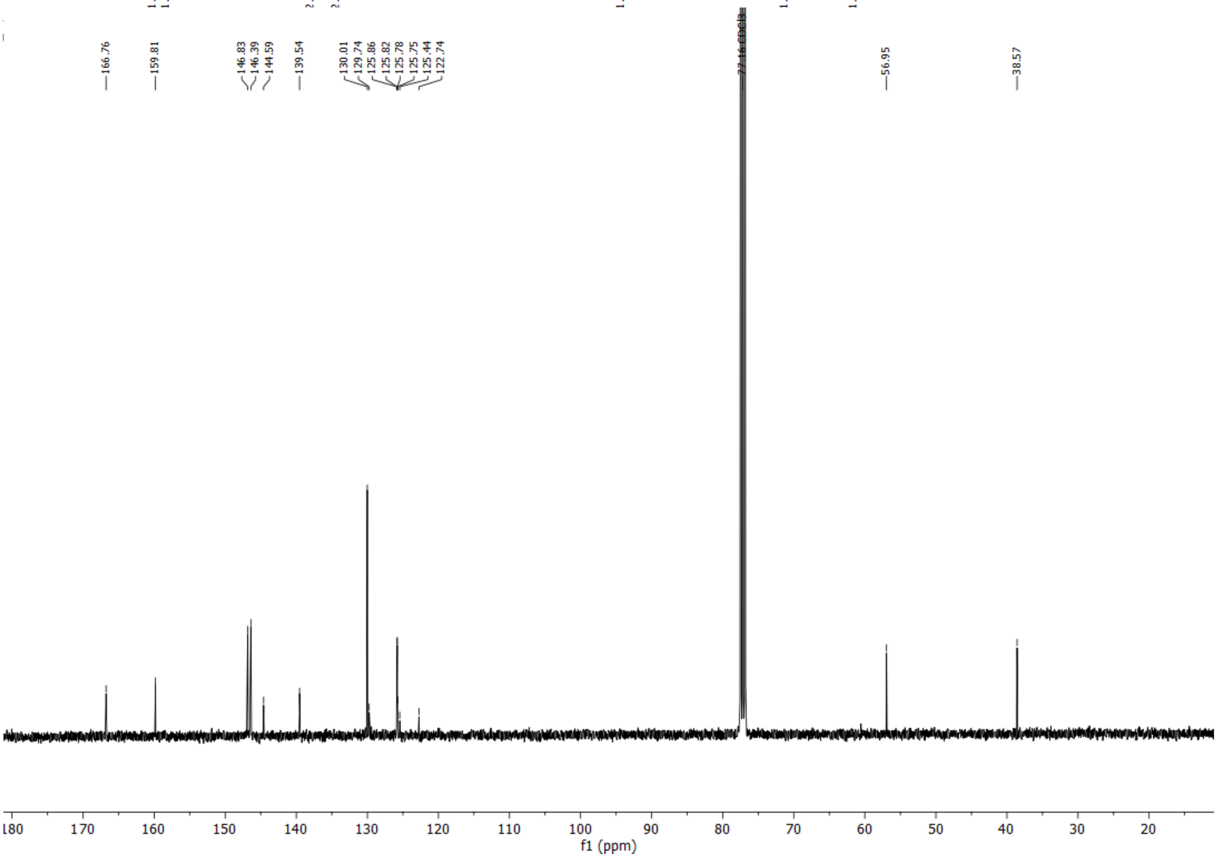

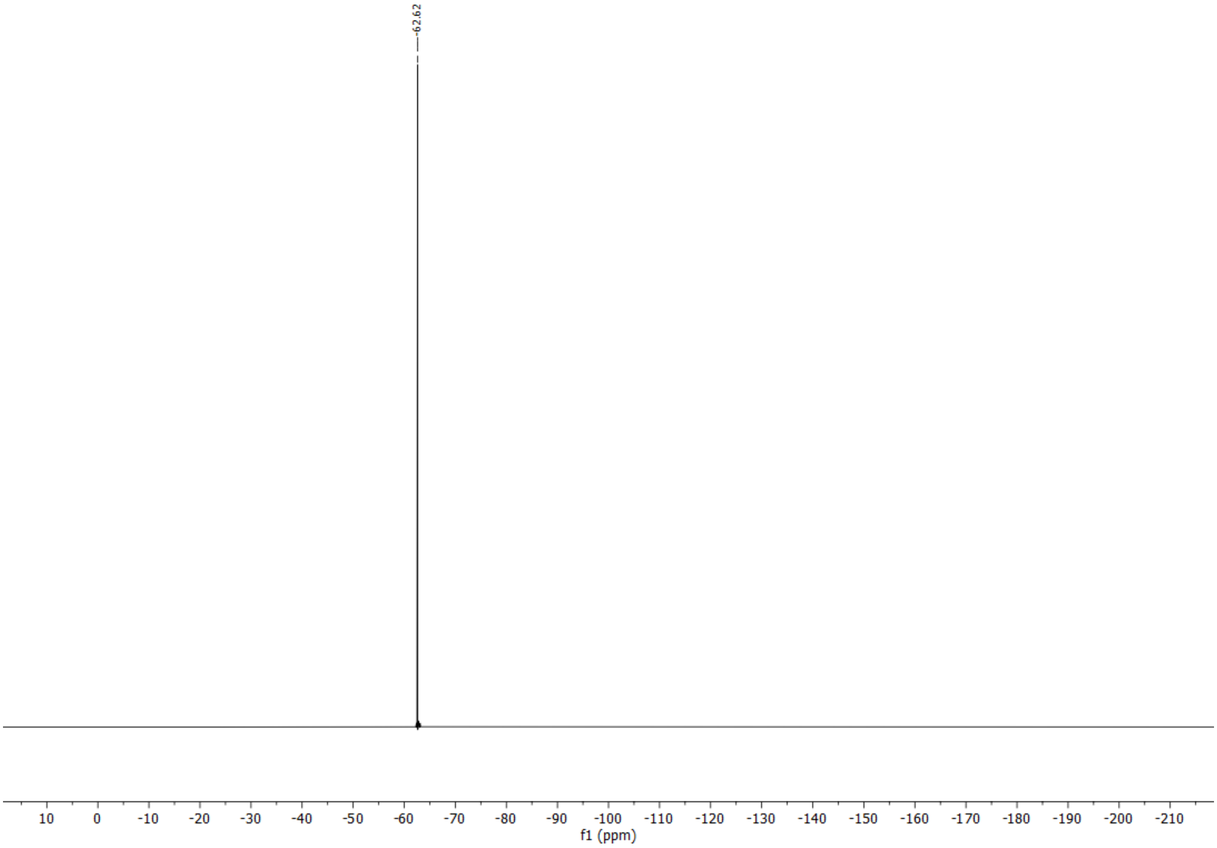

-62.62
10
0
-10
-20
-30
-40
-50
-60
-70
-80
-90
-100
-110
-120
-130
-140
-150
-160
-170
-180
-190
-200
-210
f1 (ppm)

7-(4-Bromobenzyl)-6,7-dihydro-5*H*-pyrrolo[3,4-*b*]pyrazin-5-one (*rac*-2d)

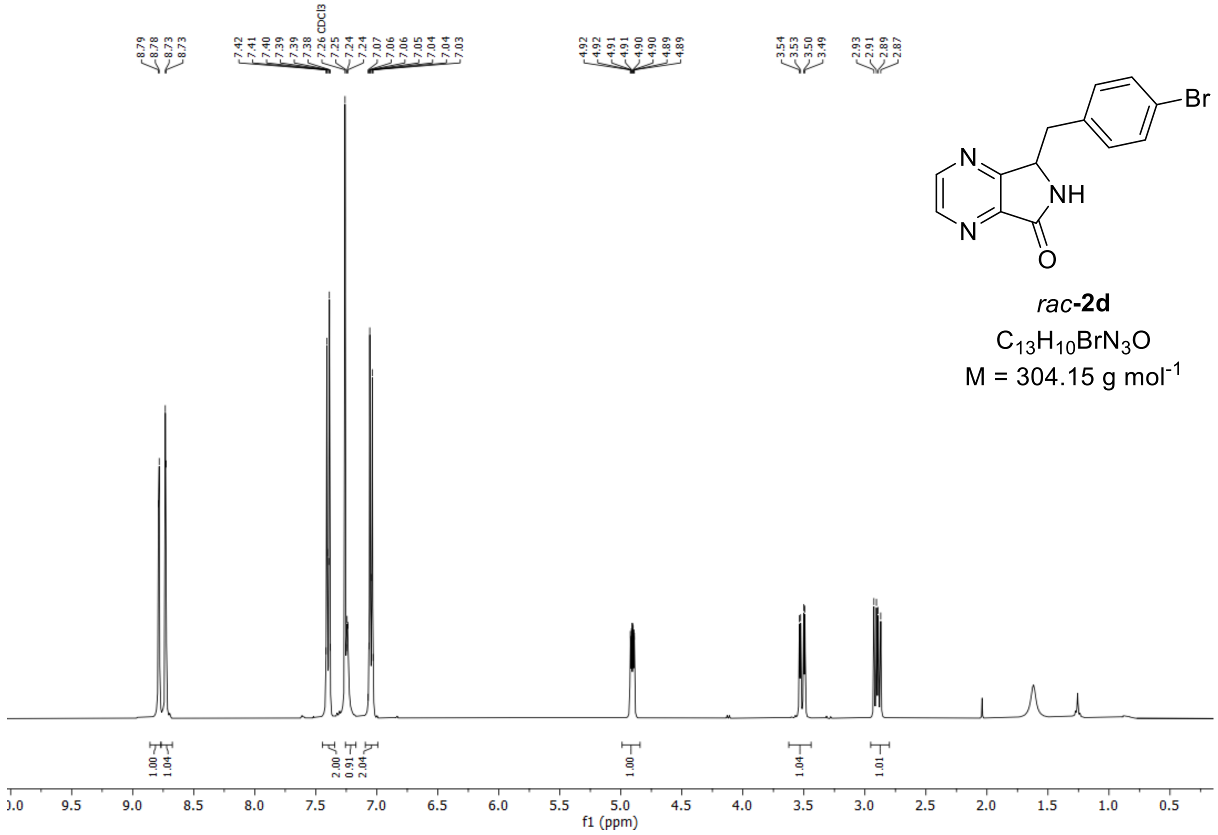

rac-**2d**

$C_{13}H_{10}BrN_3O$

M = 304.15 g mol^{-1}

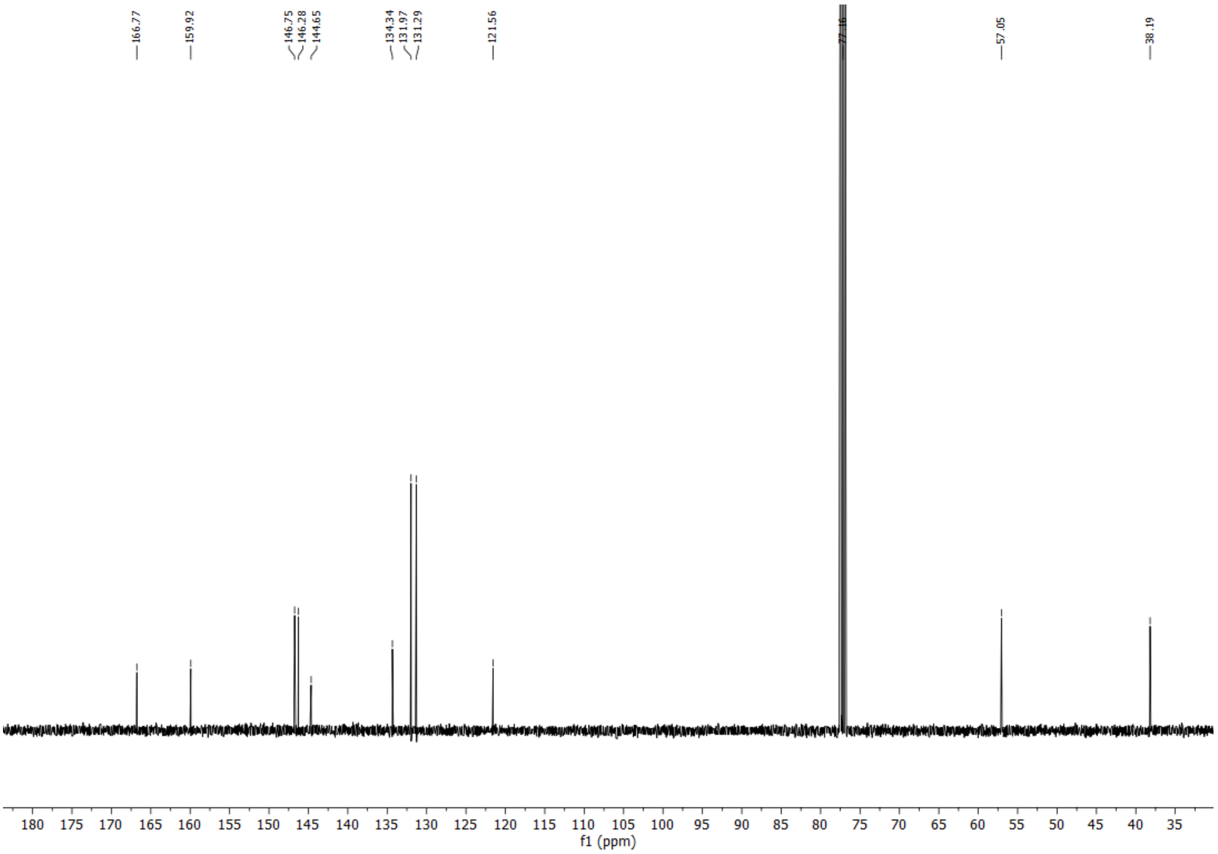

7-(2-Chlorobenzyl)-6,7-dihydro-5*H*-pyrrolo[3,4-*b*]pyrazin-5-one (*rac*-2e)

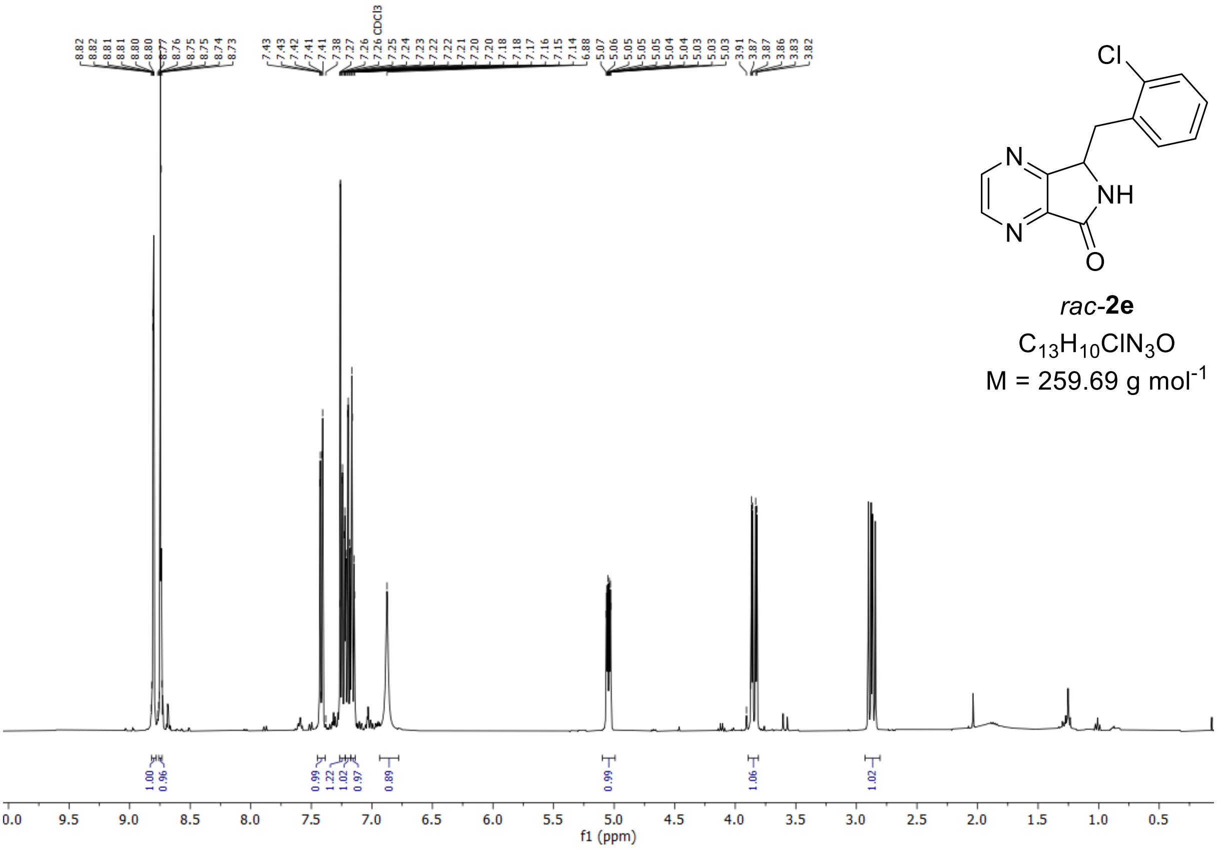

rac-**2e**

$C_{13}H_{10}ClN_3O$

M = 259.69 g mol^{-1}

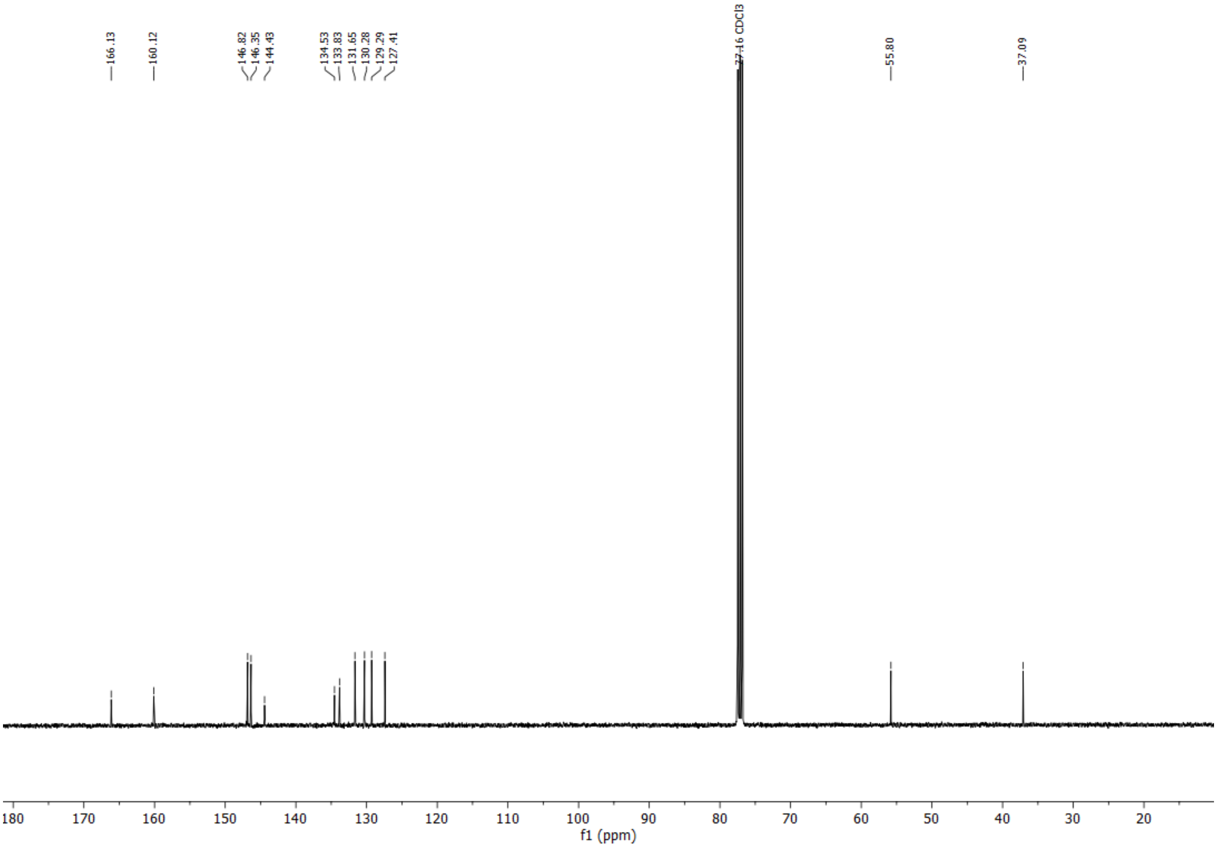

7-(4-(Trifluoromethoxy)benzyl)-6,7-dihydro-5*H*-pyrrolo[3,4-*b*]pyrazin-5-one (*rac*-2f)

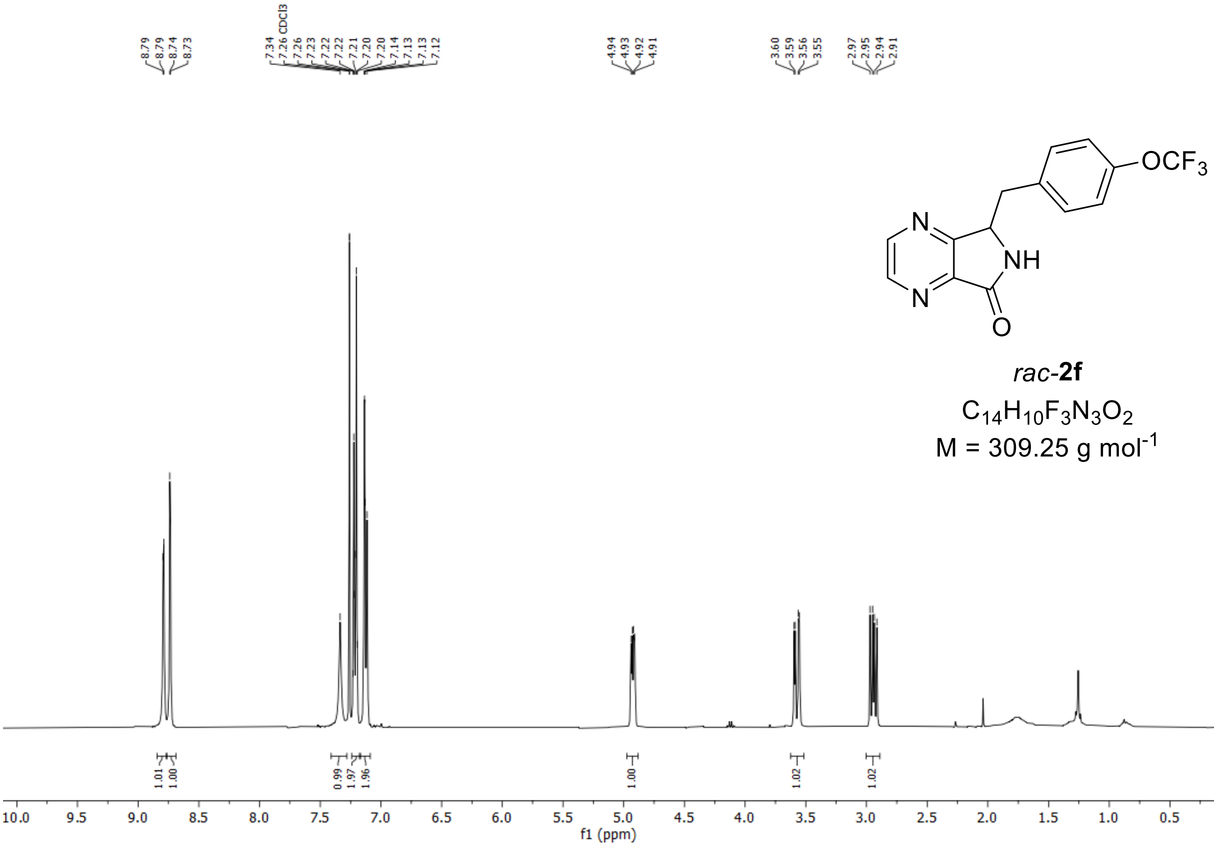

rac-**2f**

$C_{14}H_{10}F_3N_3O_2$

M = 309.25 g mol^{-1}

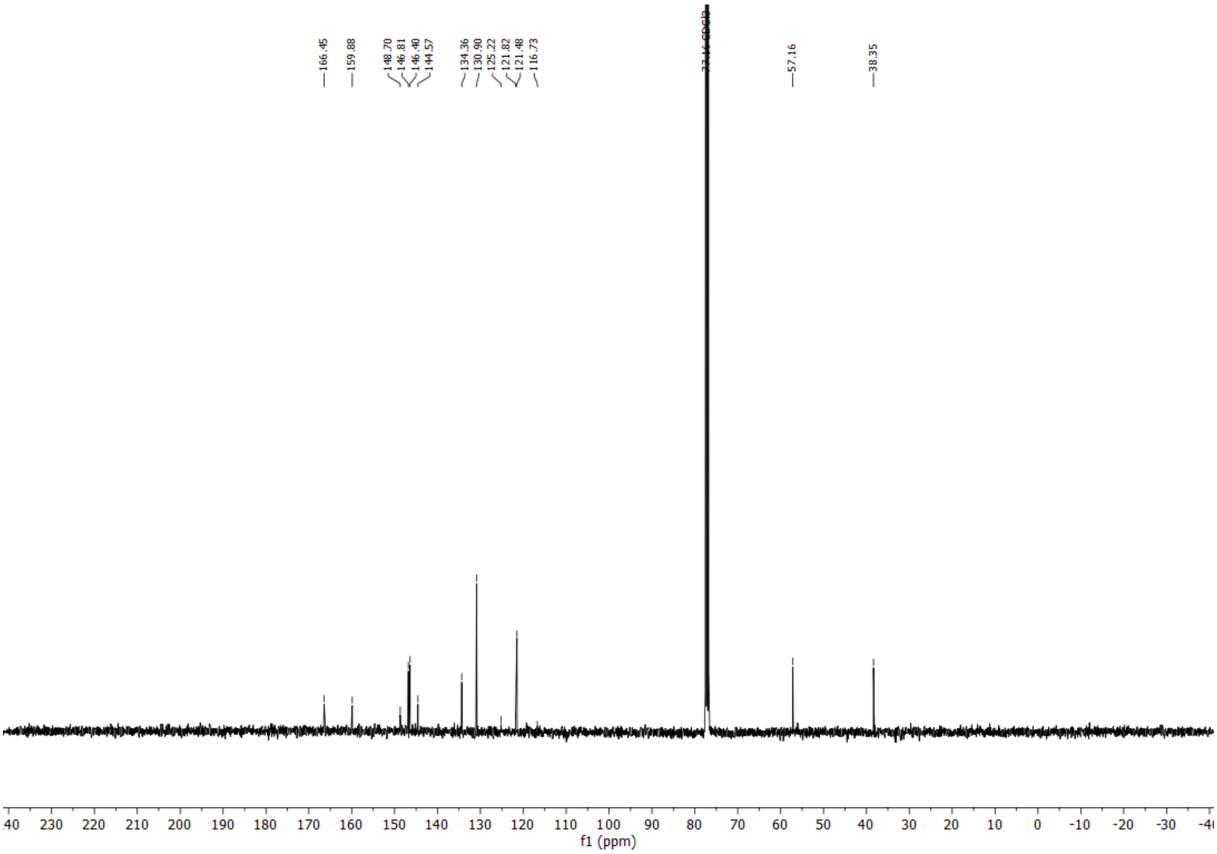

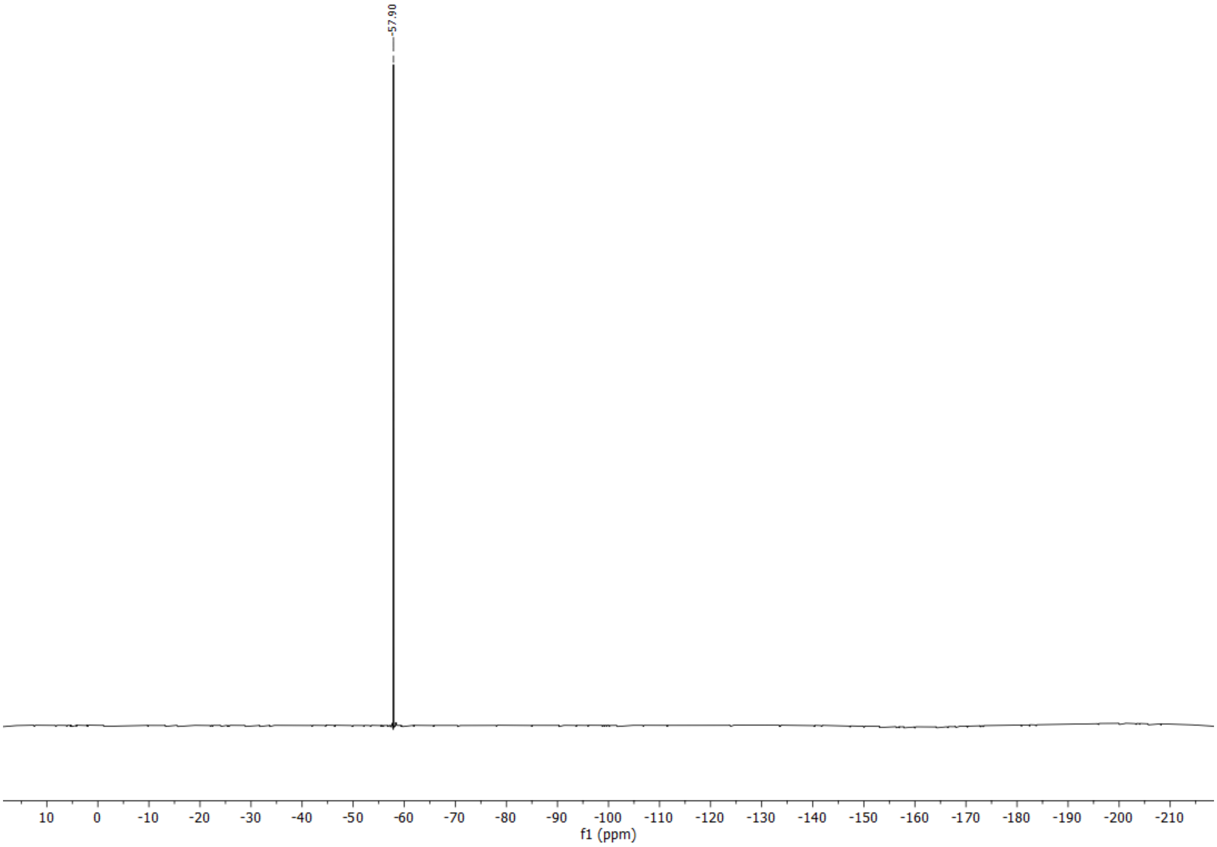
-57.90
10 0 -10 -20 -30 -40 -50 -60 -70 -80 -90 -100 -110 -120 -130 -140 -150 -160 -170 -180 -190 -200 -210
f1 (ppm)

7-(3,5-Dimethylbenzyl)-6,7-dihydro-5*H*-pyrrolo[3,4-*b*]pyrazin-5-one (*rac*-2g)

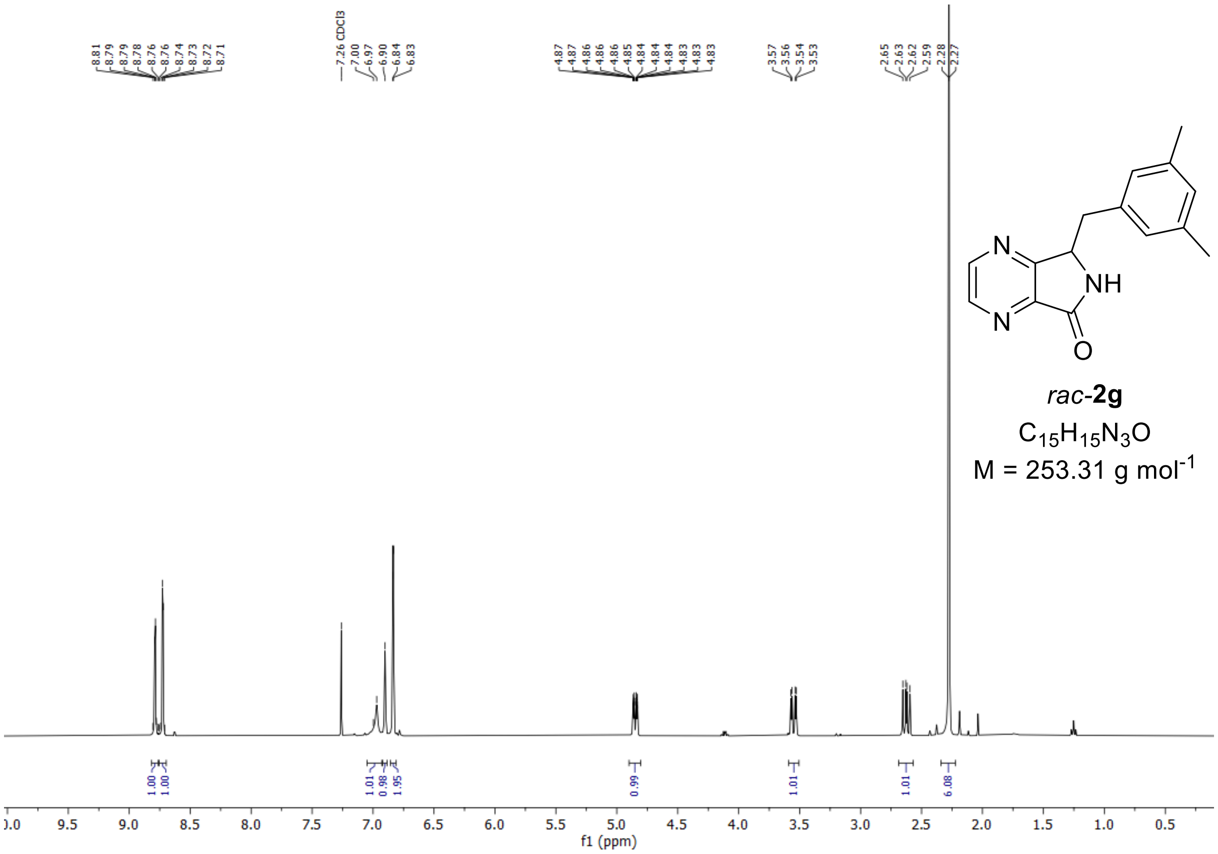

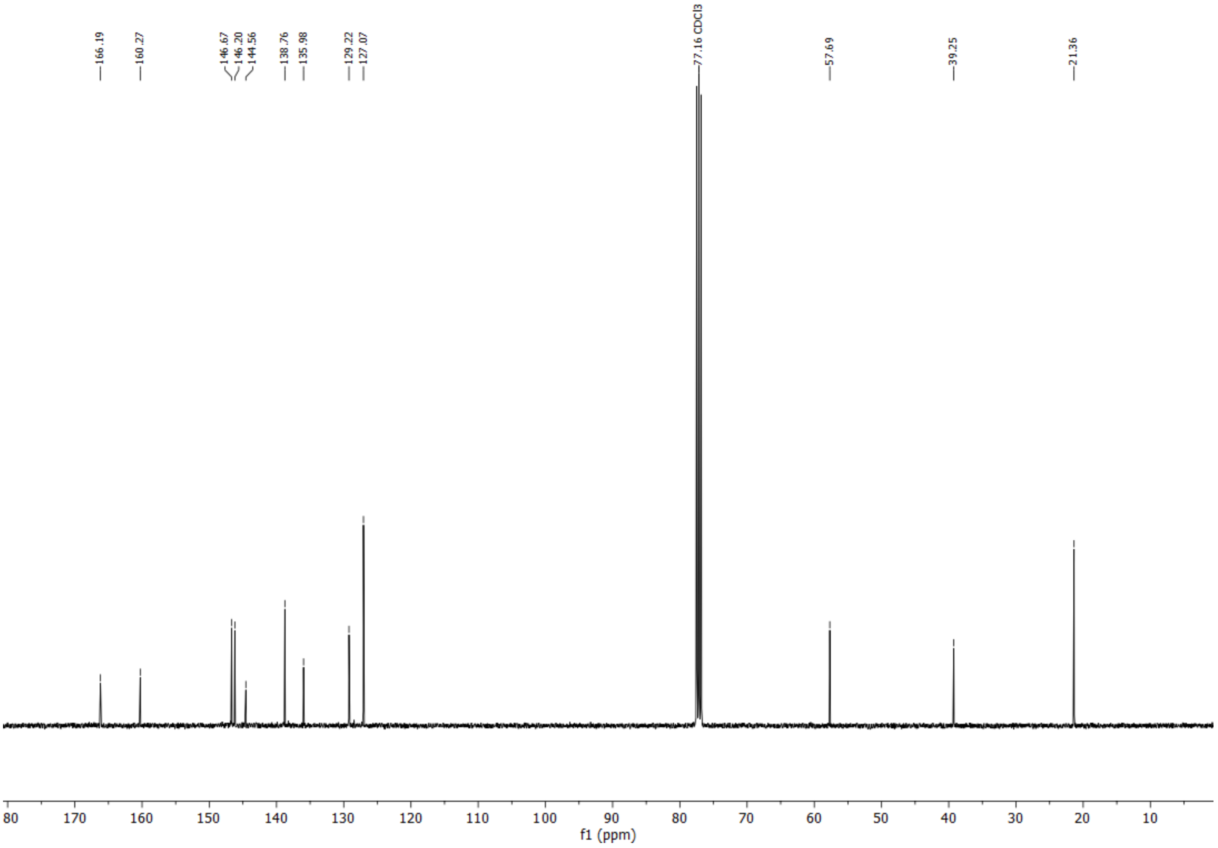

***tert*-Butyl 4-((7-oxo-6,7-dihydro-5*H*-pyrrolo[3,4-*b*]pyrazin-5-yl)methyl)benzoate (*rac*-2h)**

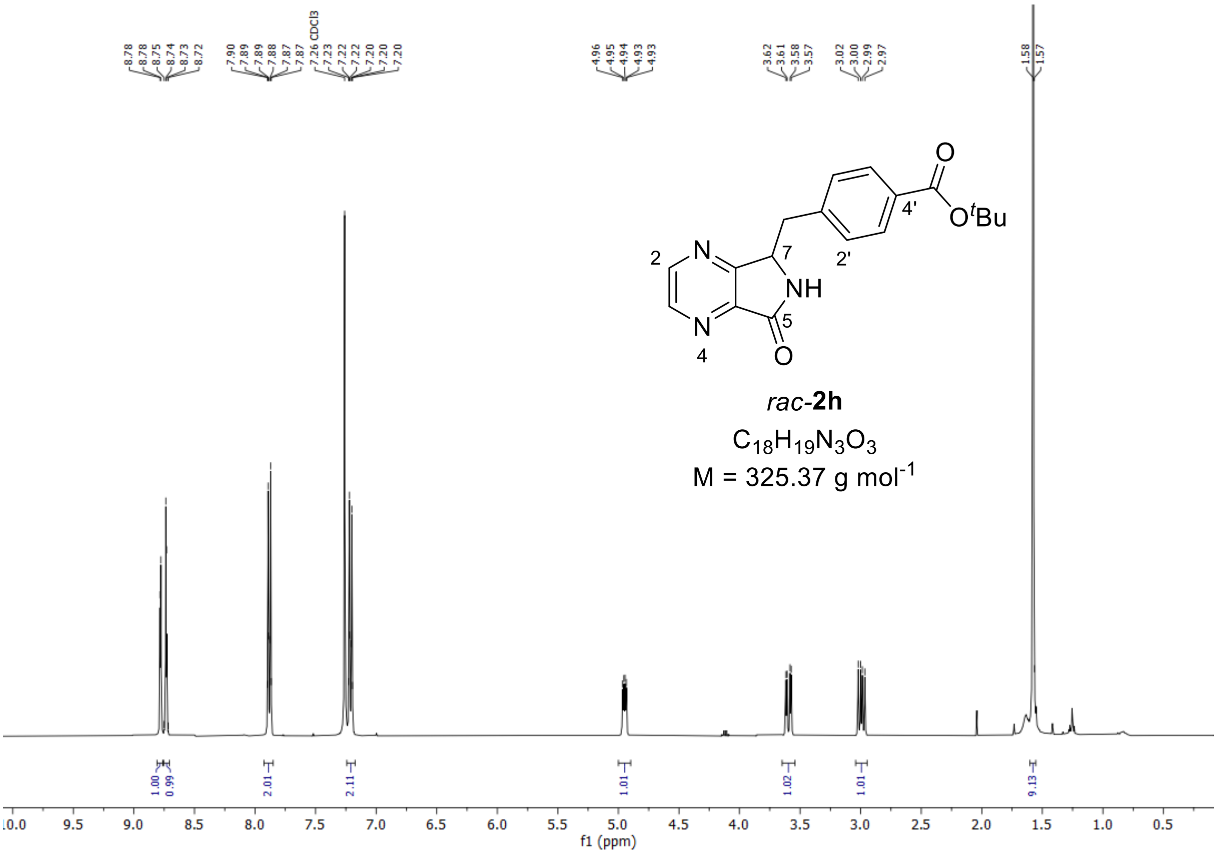

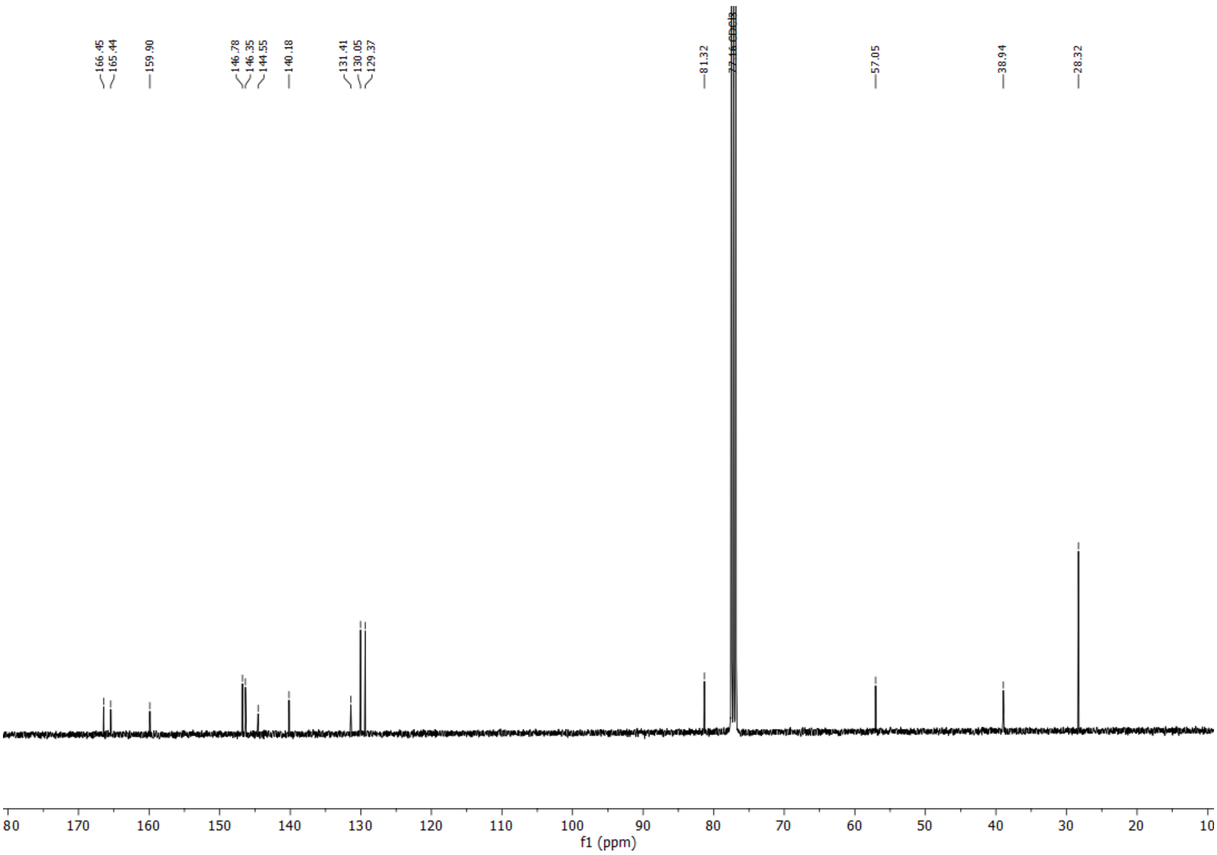

7-((5-(Trifluoromethyl)furan-2-yl)methyl)-6,7-dihydro-5*H*-pyrrolo[3,4-*b*]pyrazin-5-one (*rac*-2i)

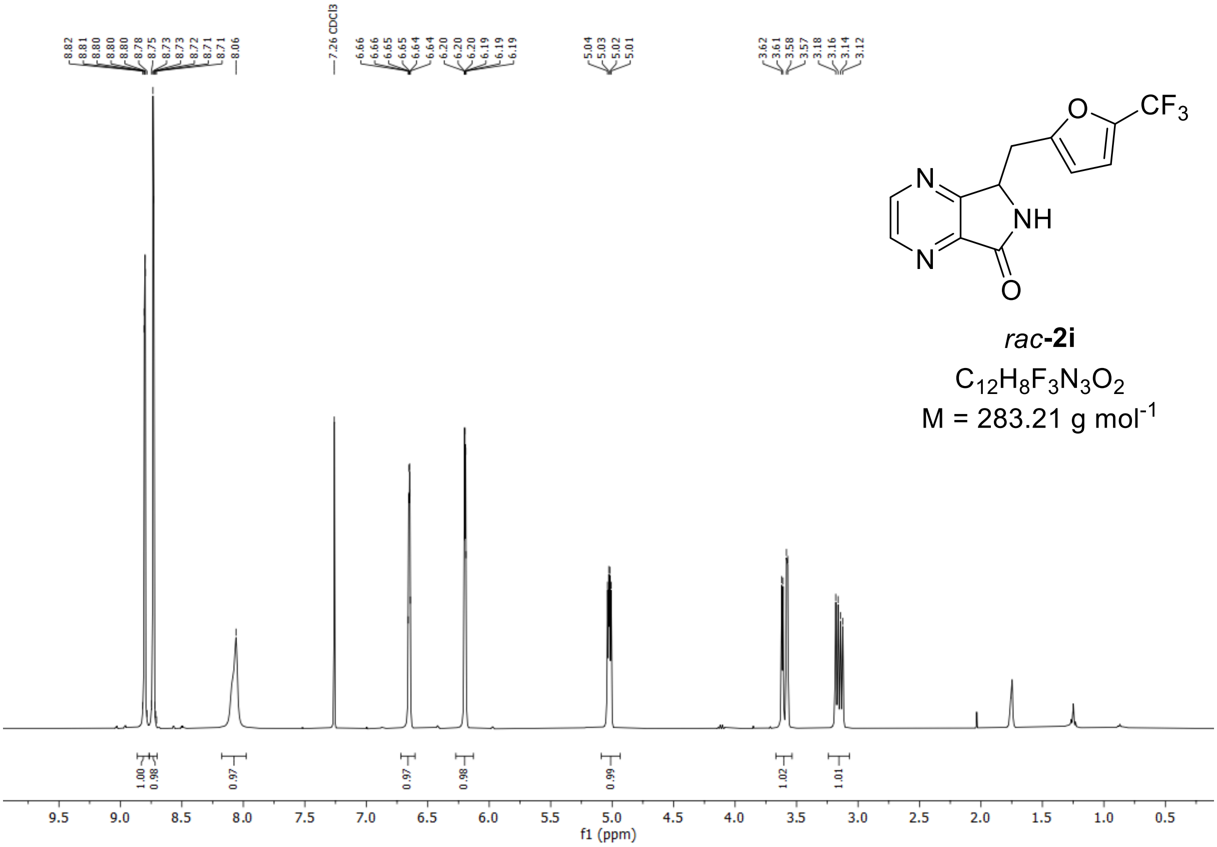

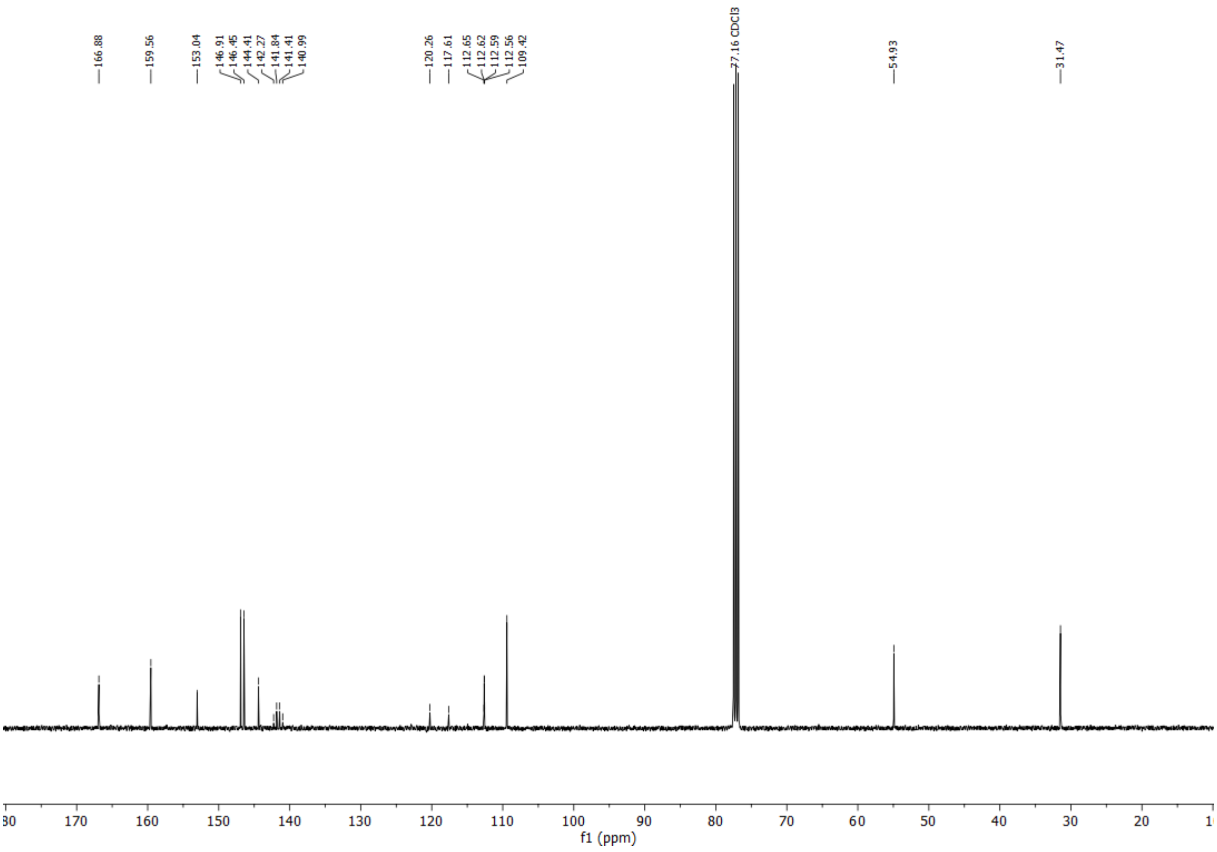

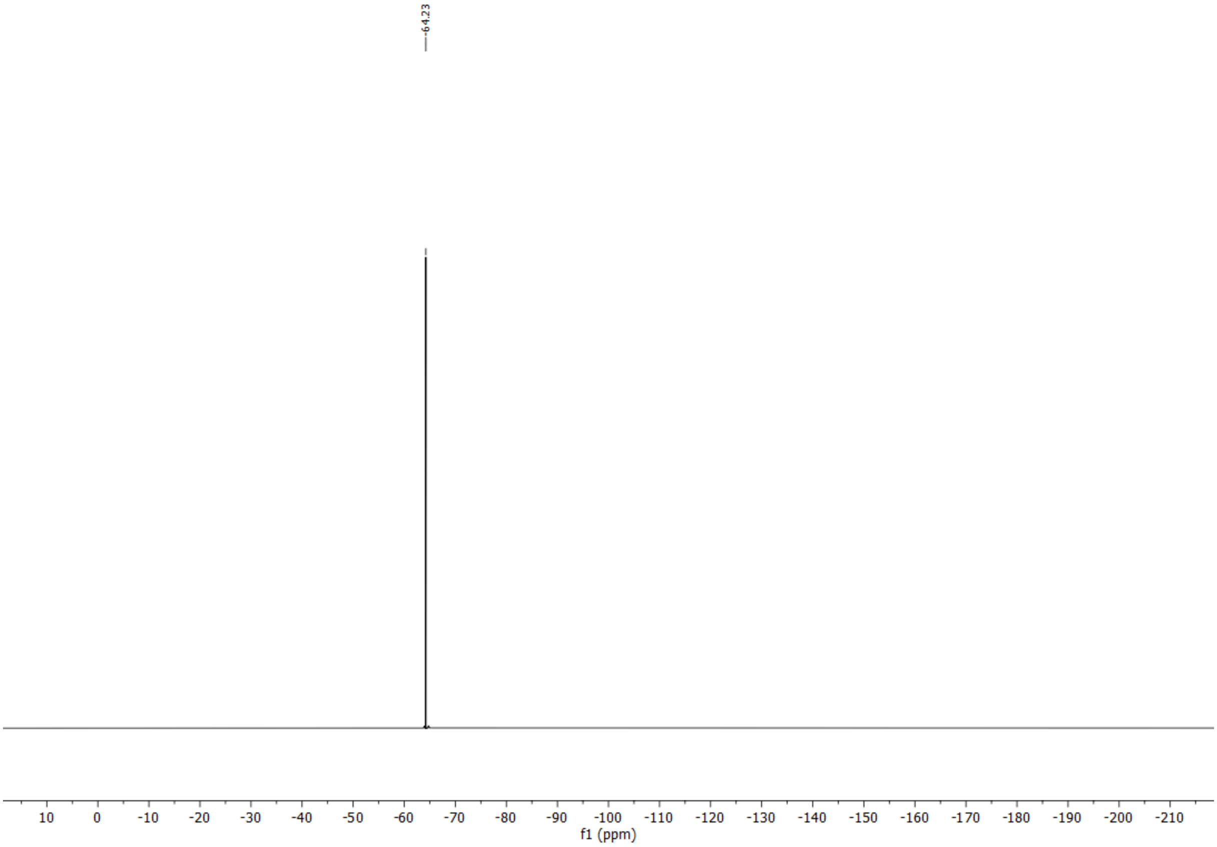
-64.23
10
0
-10
-20
-30
-40
-50
-60
-70
-80
-90
-100
-110
-120
-130
-140
-150
-160
-170
-180
-190
-200
-210
f1 (ppm)

7-Ethyl-6,7-dihydro-5*H*-pyrrolo[3,4-*b*]pyrazin-5-one (*rac*-2j)

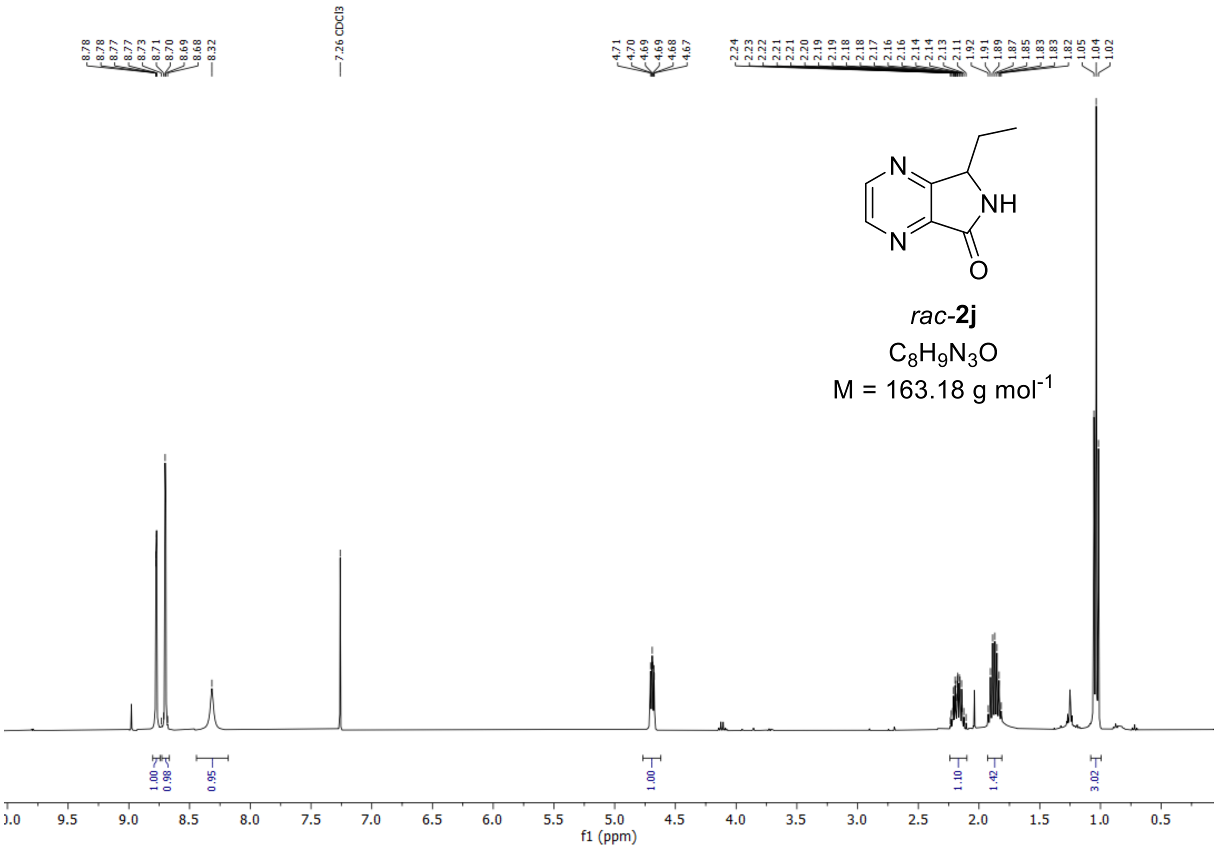

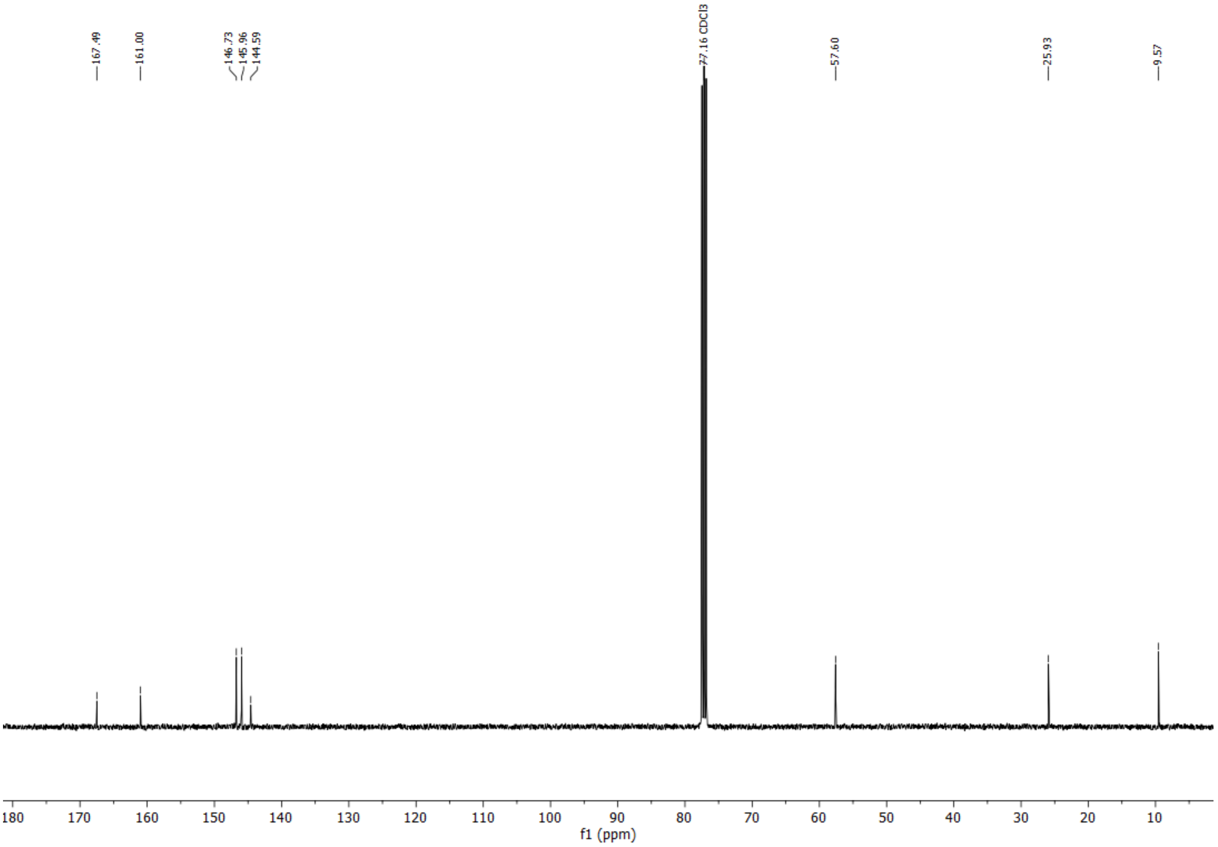

7-Isopentyl-6,7-dihydro-5*H*-pyrrolo[3,4-*b*]pyrazin-5-one (*rac*-2k)

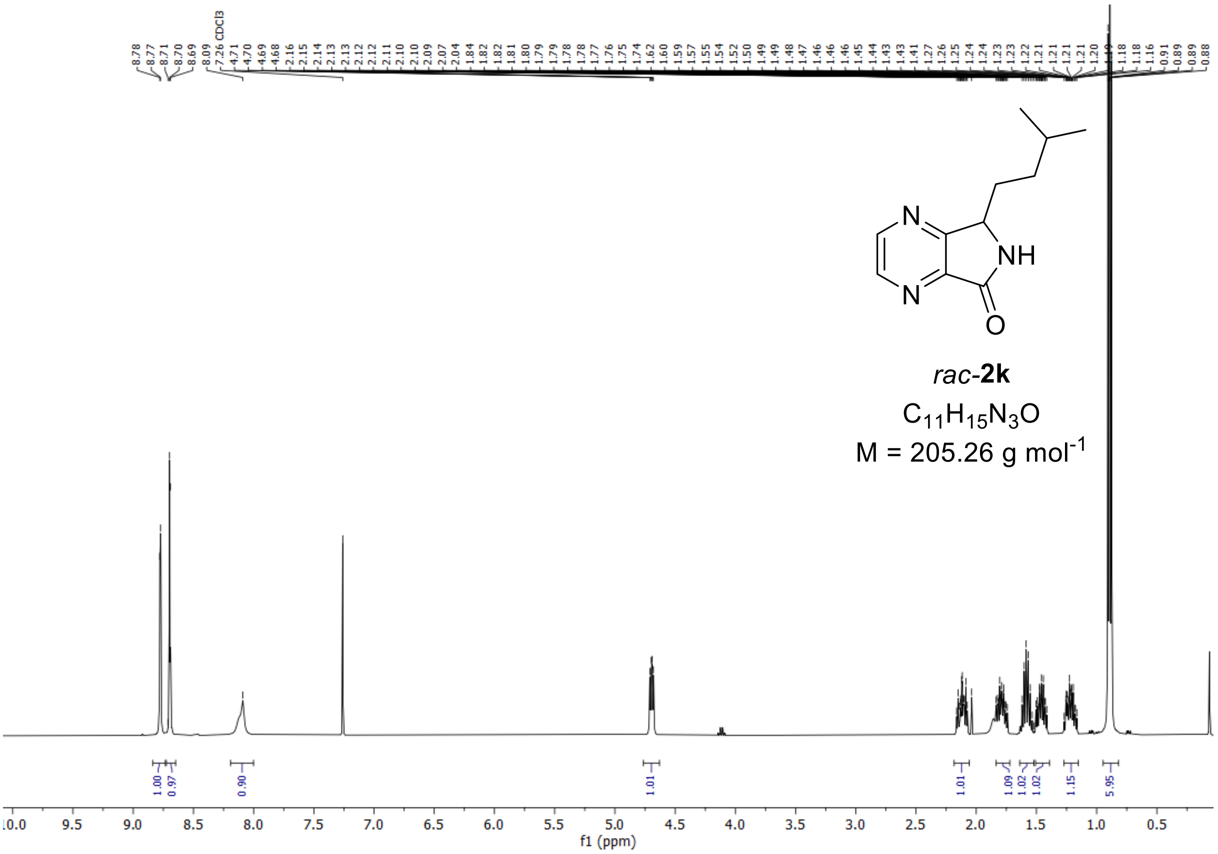

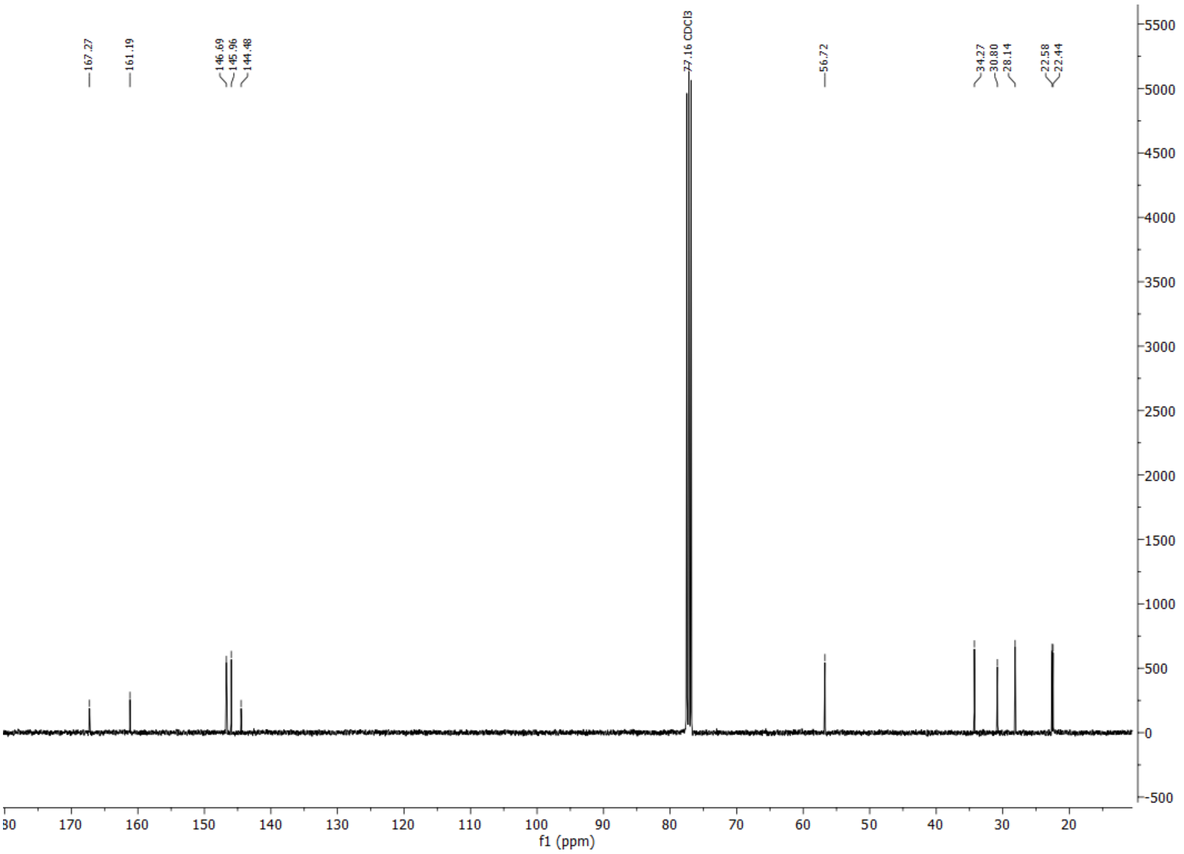

7-(Cyclopentylmethyl)-6,7-dihydro-5*H*-pyrrolo[3,4-*b*]pyrazin-5-one (*rac*-2l)

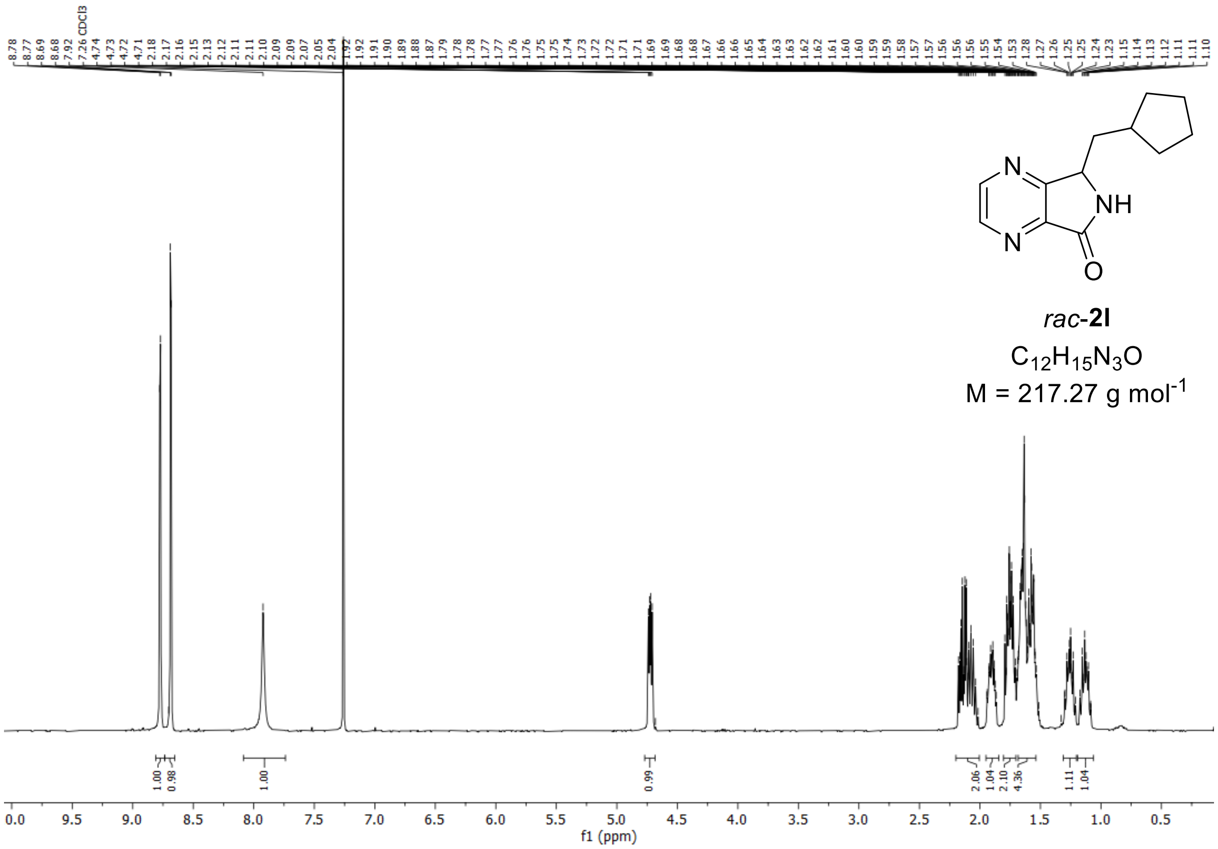

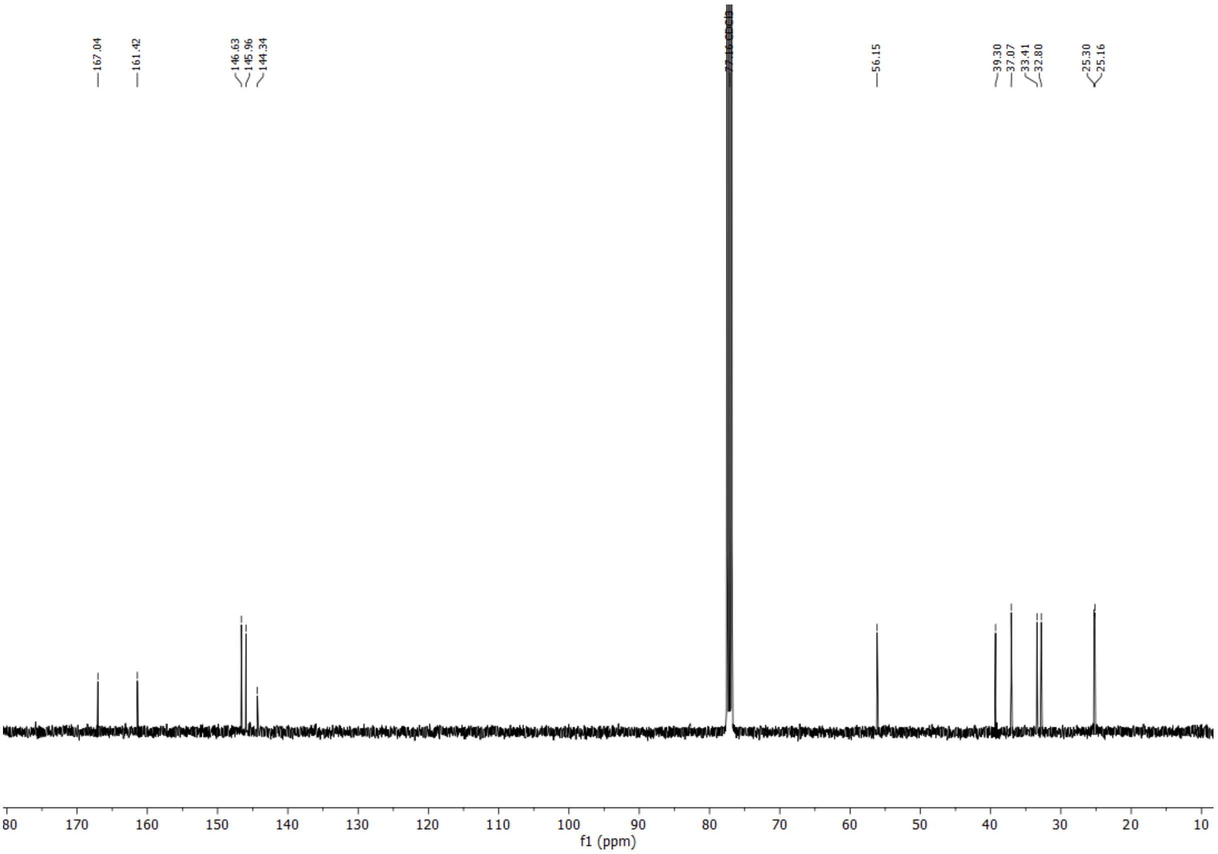

7-(Cyclopropylmethyl)-6,7-dihydro-5*H*-pyrrolo[3,4-*b*]pyrazin-5-one (*rac*-2m)

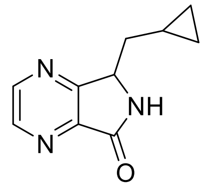

rac-**2m**

$C_{10}H_{11}N_3O$

M = 189.22 g mol^{-1}

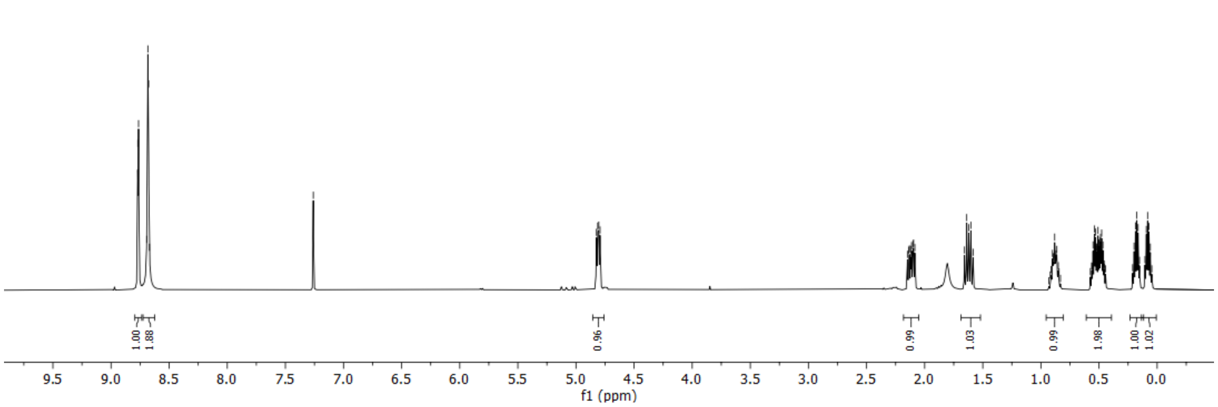

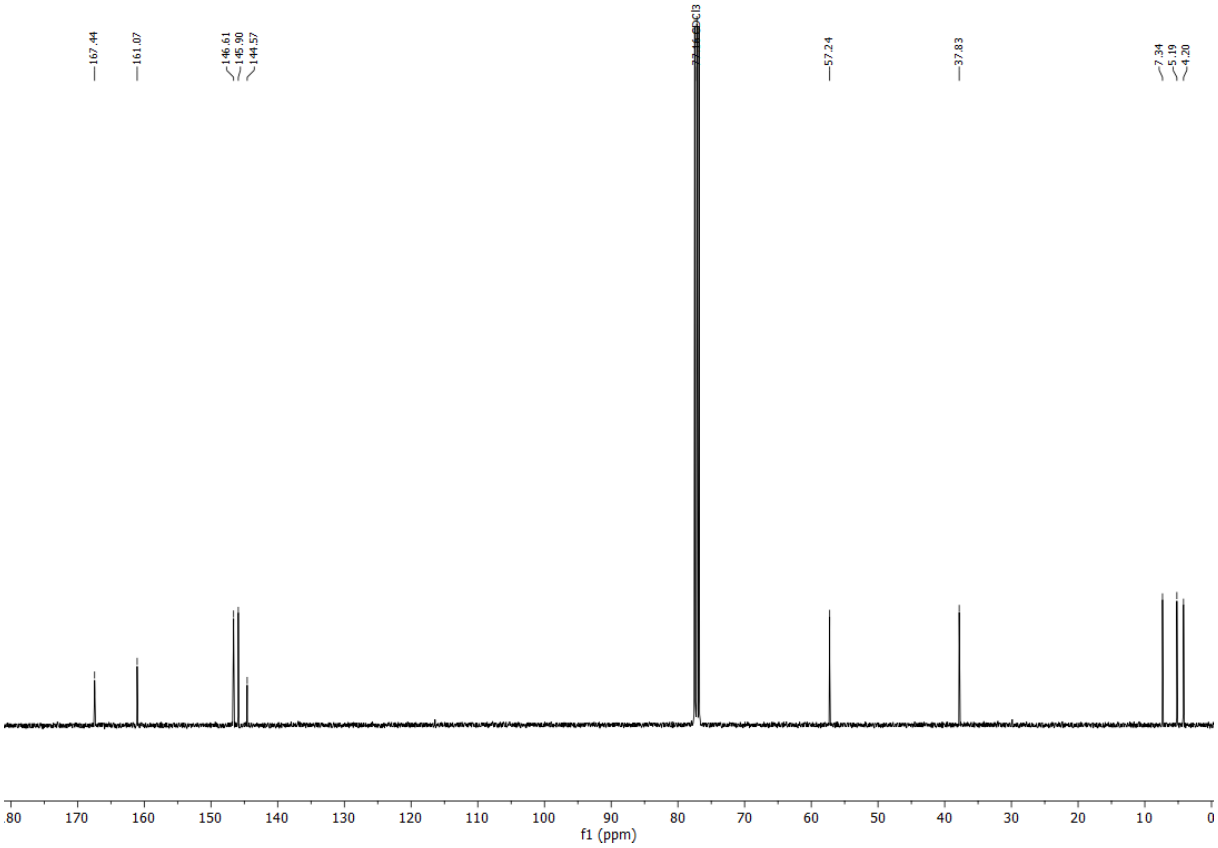

Pyrazine-2,3-dicarboximide (SI-10)

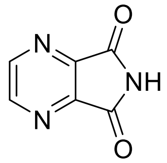

SI-10

$C_6H_3N_3O_2$

M = 149.11 g mol^{-1}

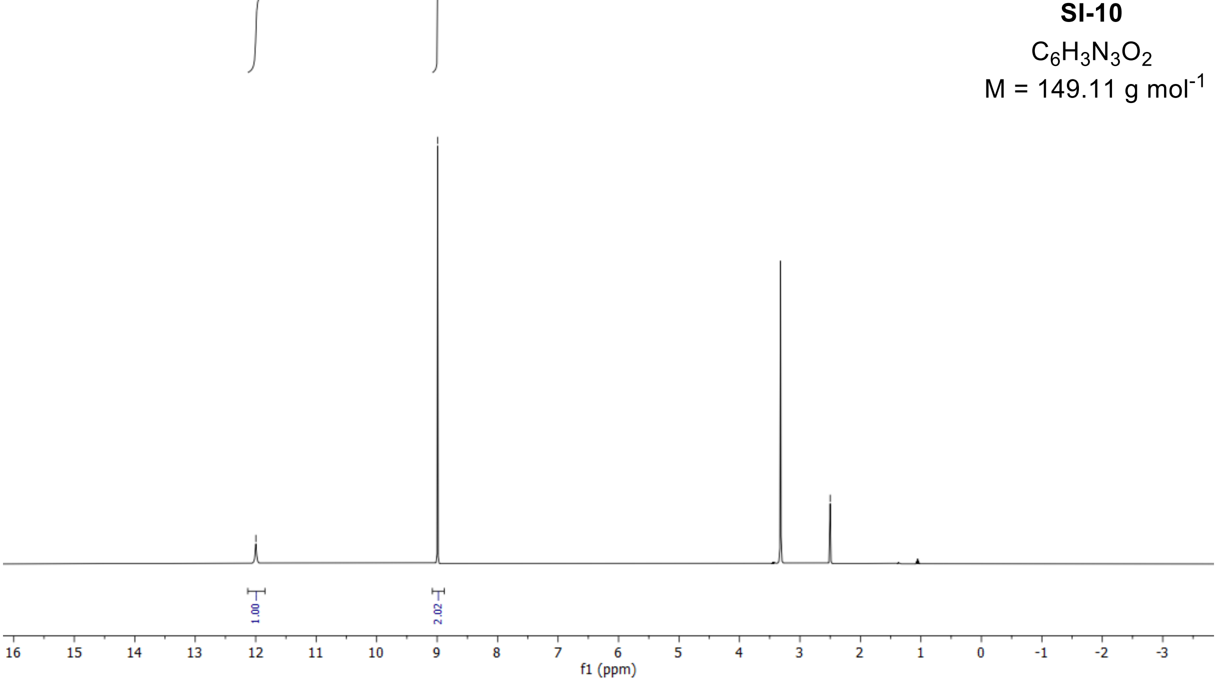

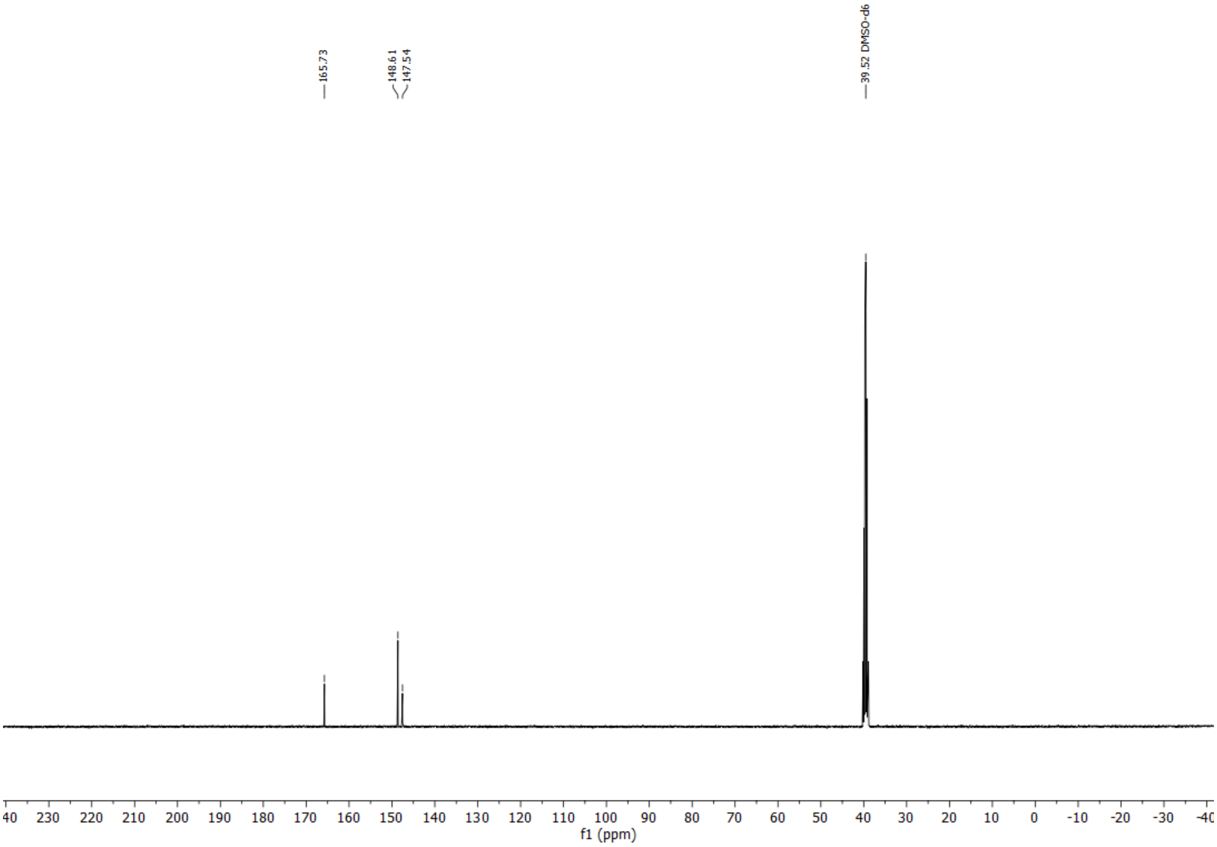

7-Hydroxy-6,7-dihydro-5*H*-pyrrolo[3,4-*b*]pyrazin-5-one (SI-11)

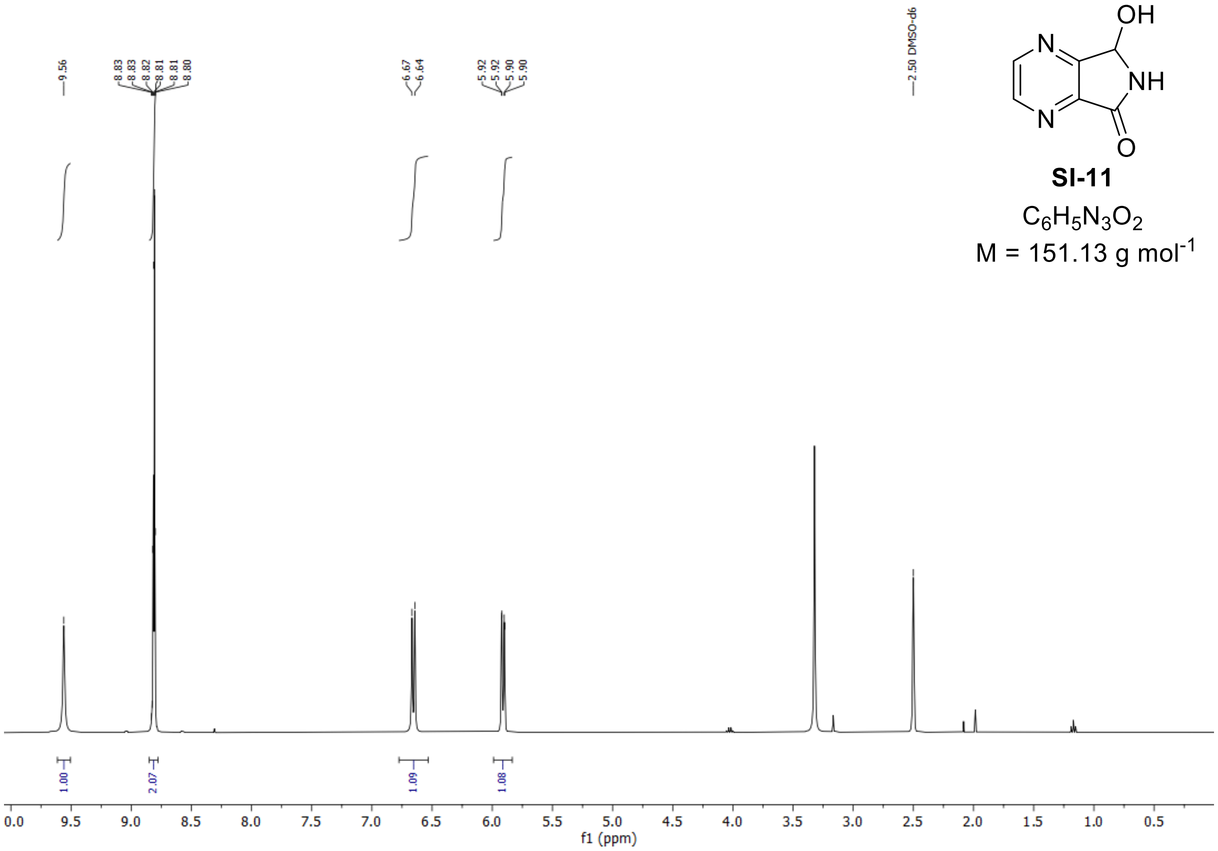

SI-11
$C_6H_5N_3O_2$
M = 151.13 g mol^{-1}

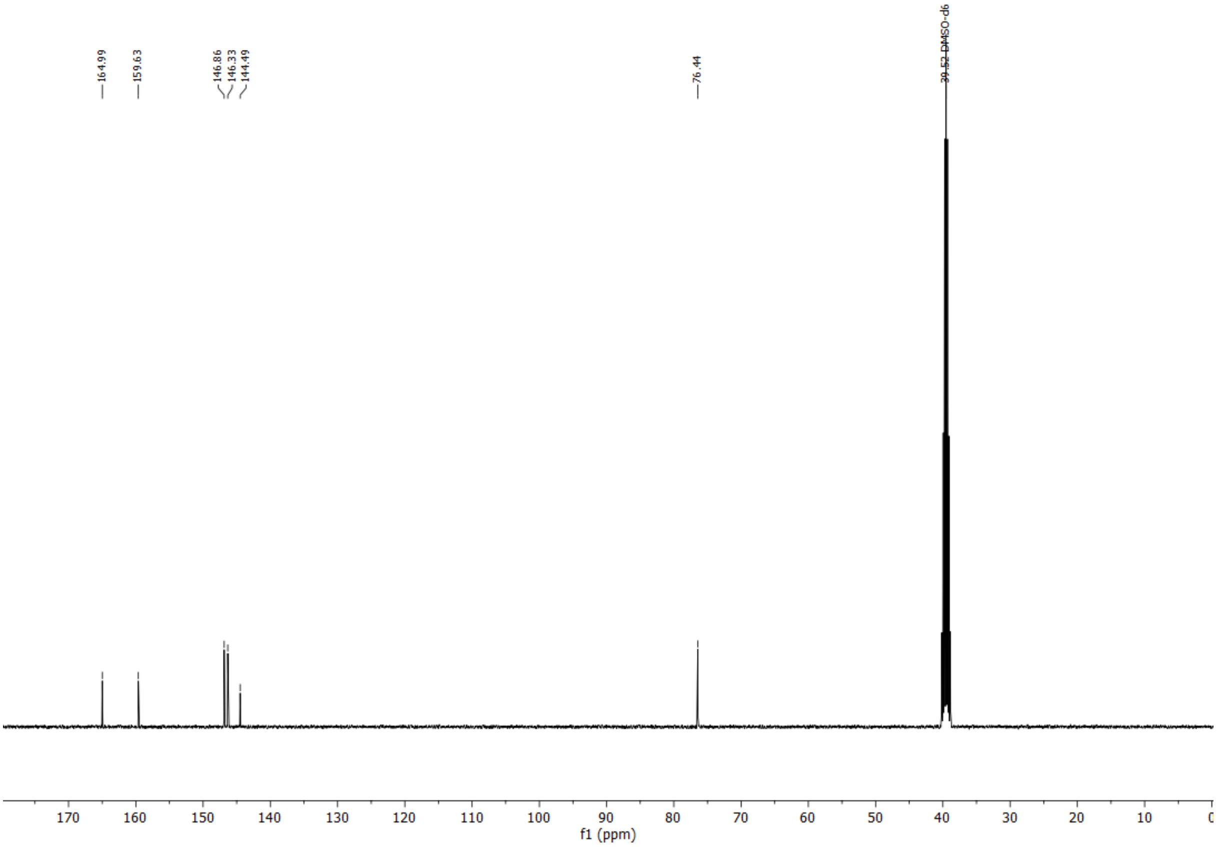

7-(2-Chloroethoxy)-6,7-dihydro-5*H*-pyrrolo[3,4-*b*]pyrazin-5-one (*rac*-2n)

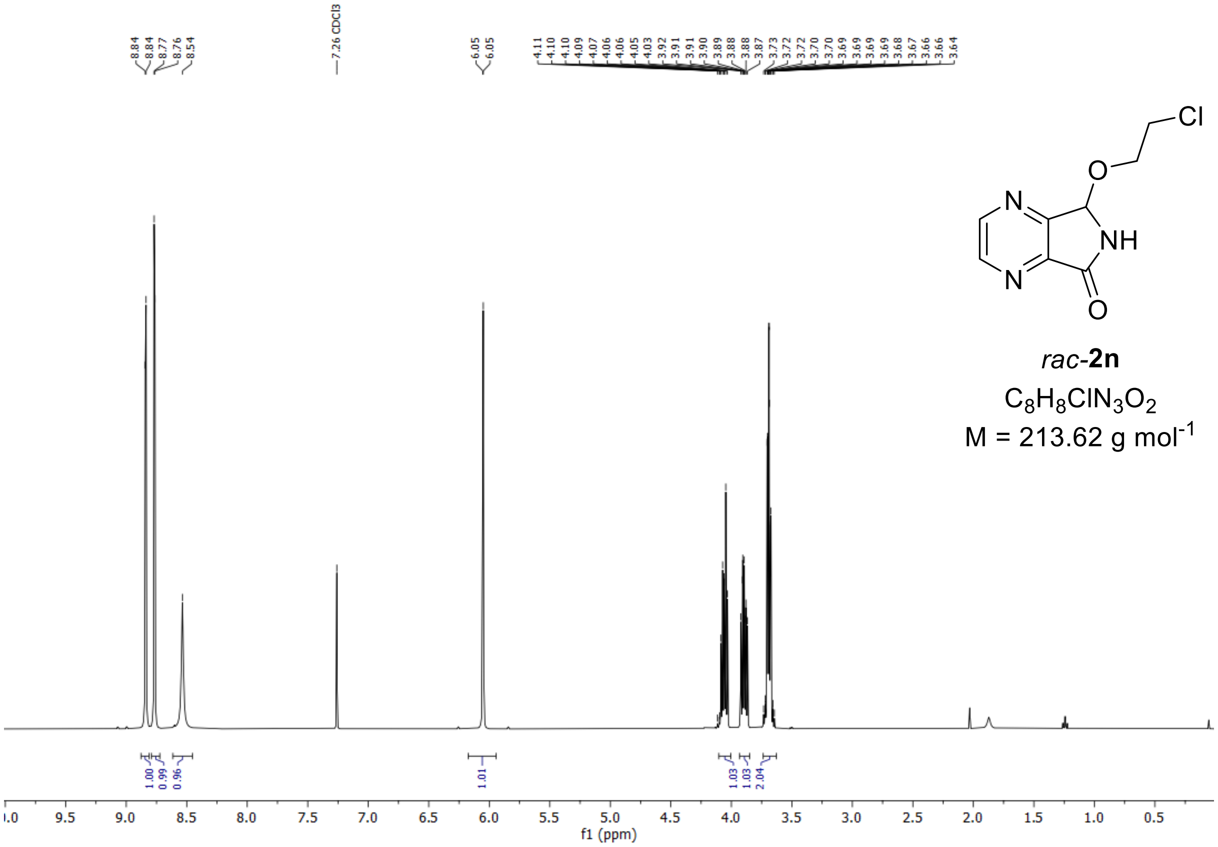

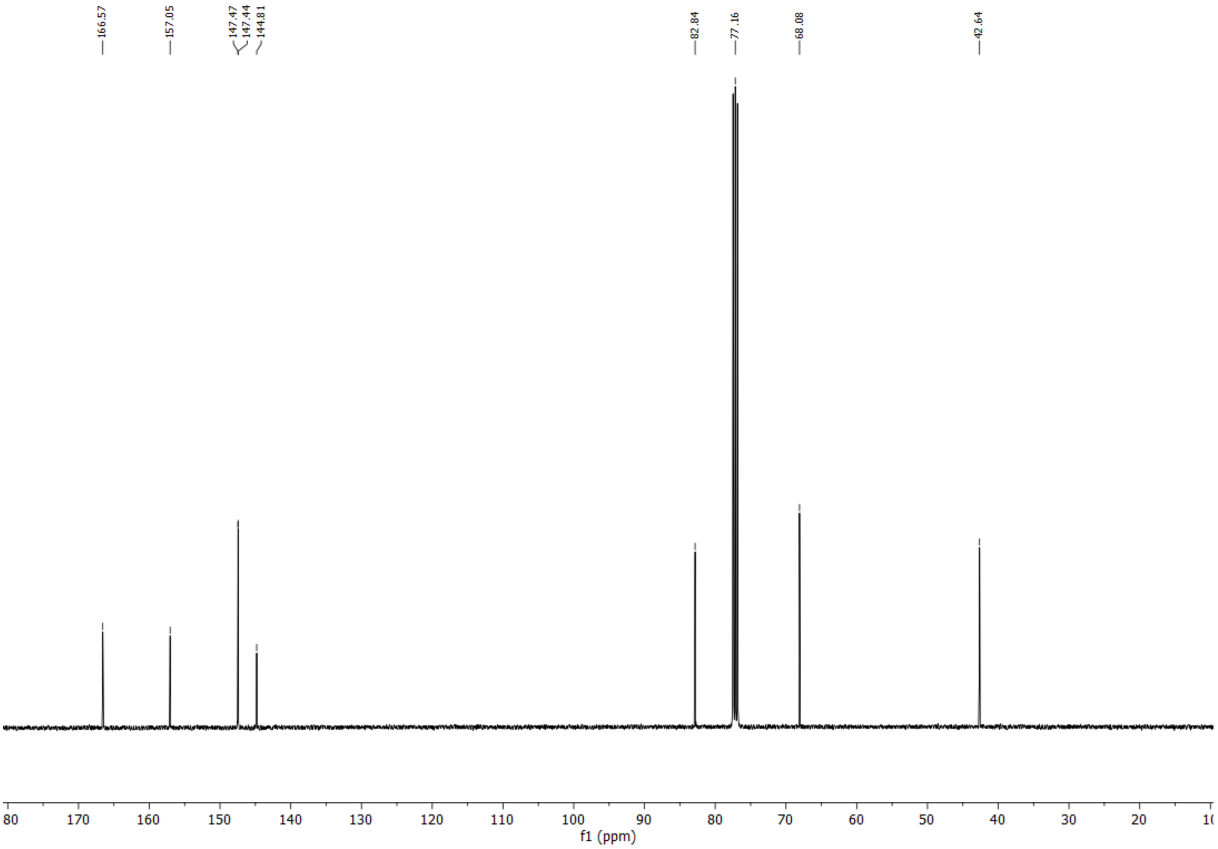

7-Isobutoxy-6,7-dihydro-5*H*-pyrrolo[3,4-*b*]pyrazin-5-one (*rac*-2o)

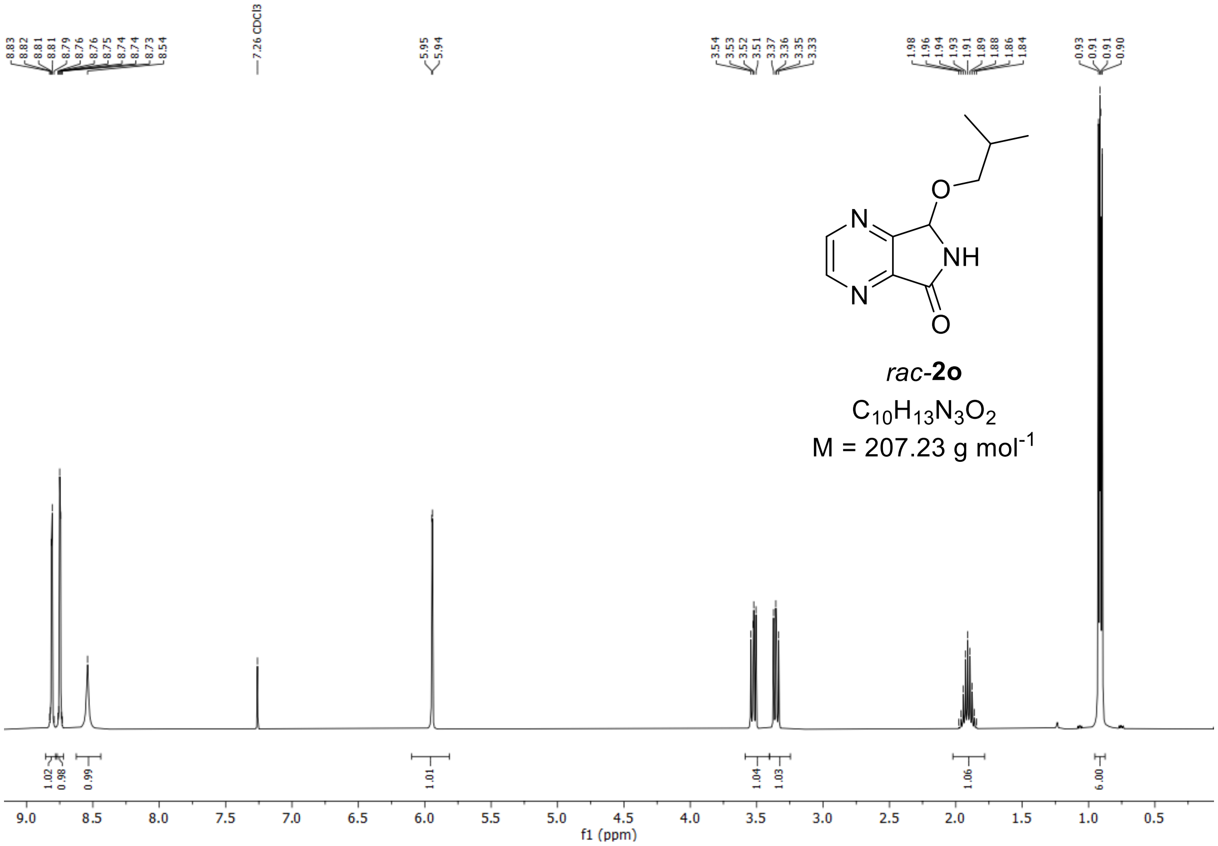

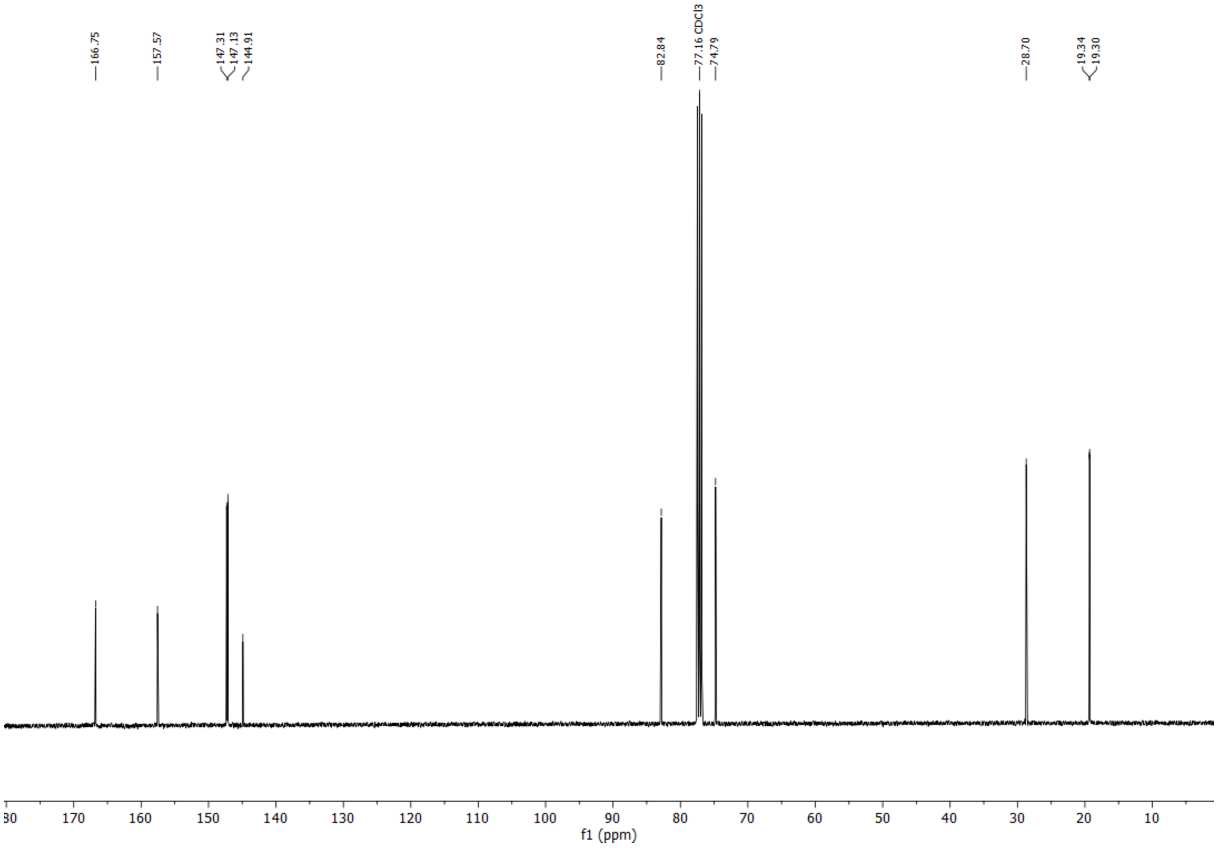

7-(But-3-en-1-yloxy)-6,7-dihydro-5*H*-pyrrolo[3,4-*b*]pyrazin-5-one (*rac*-2p)

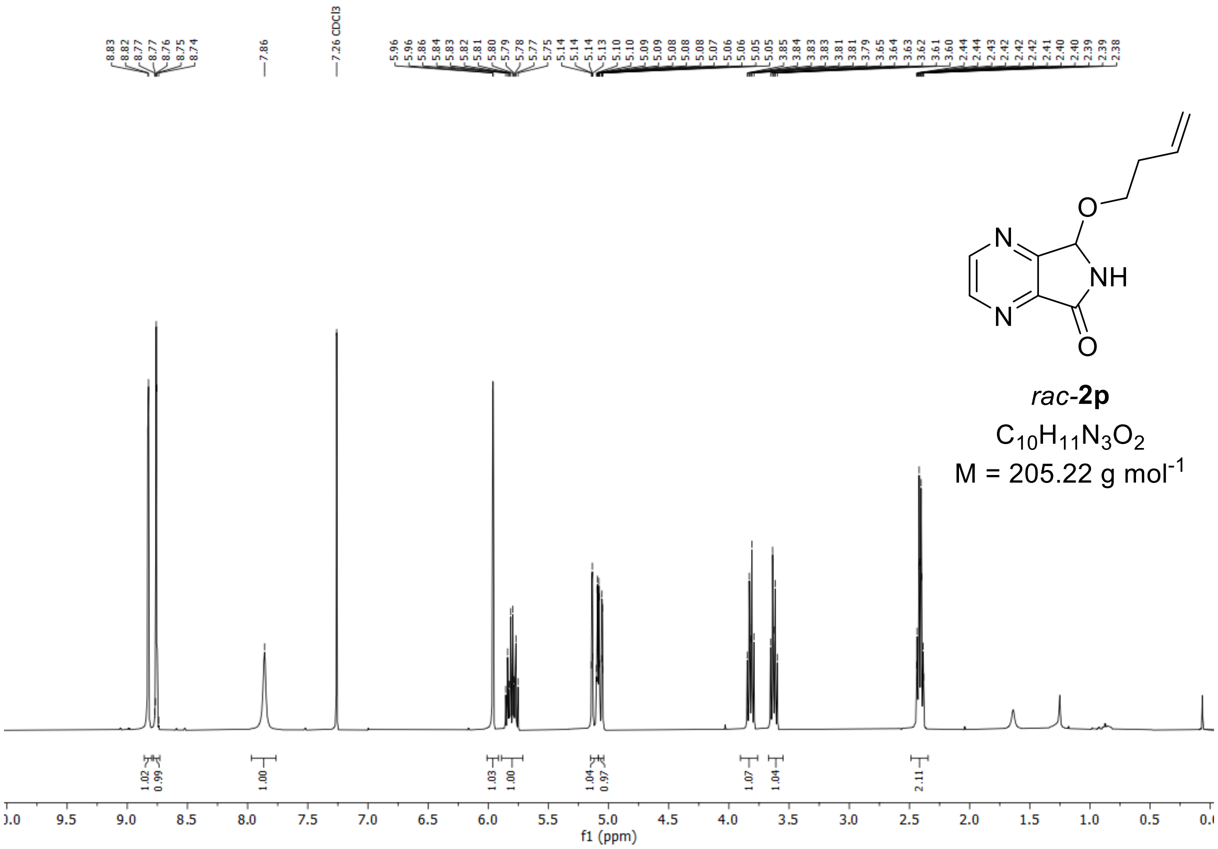

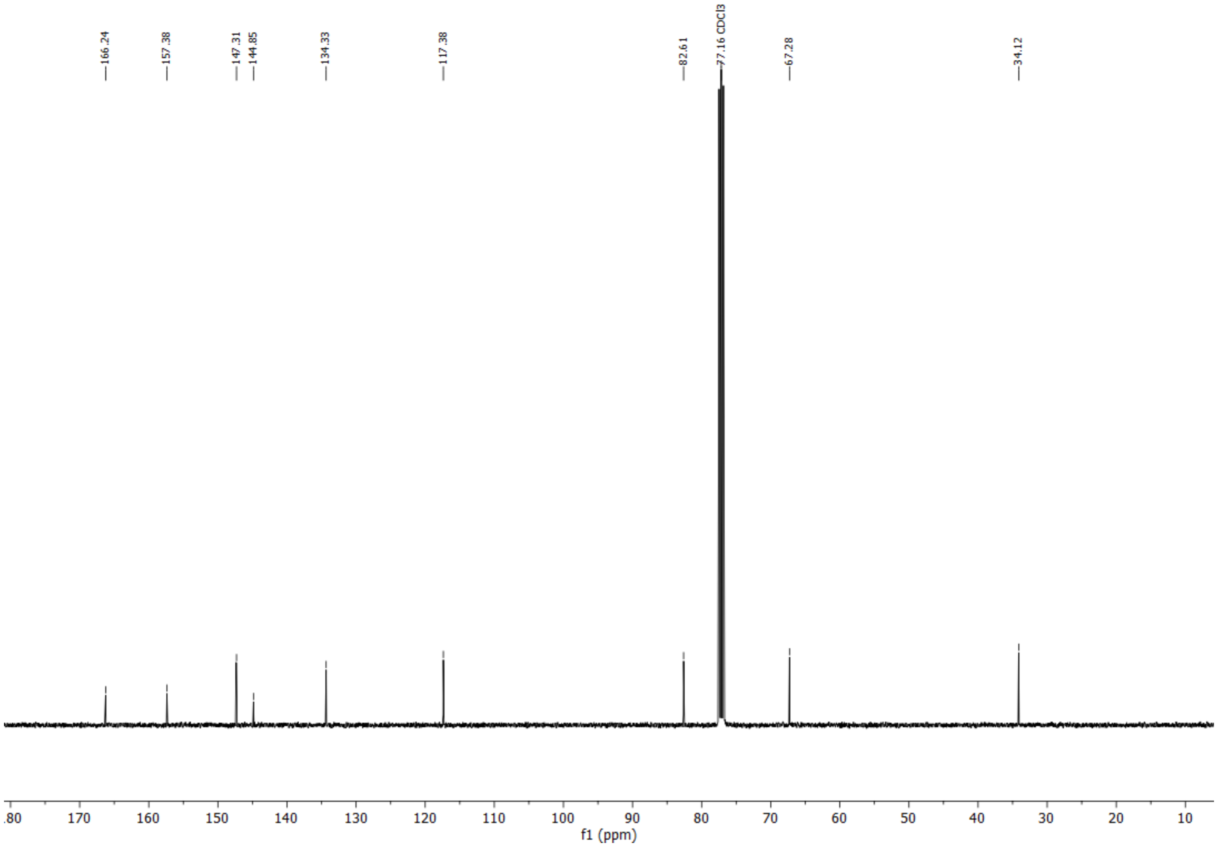

7-Isopropoxy-6,7-dihydro-5*H*-pyrrolo[3,4-*b*]pyrazin-5-one (*rac*-2q)

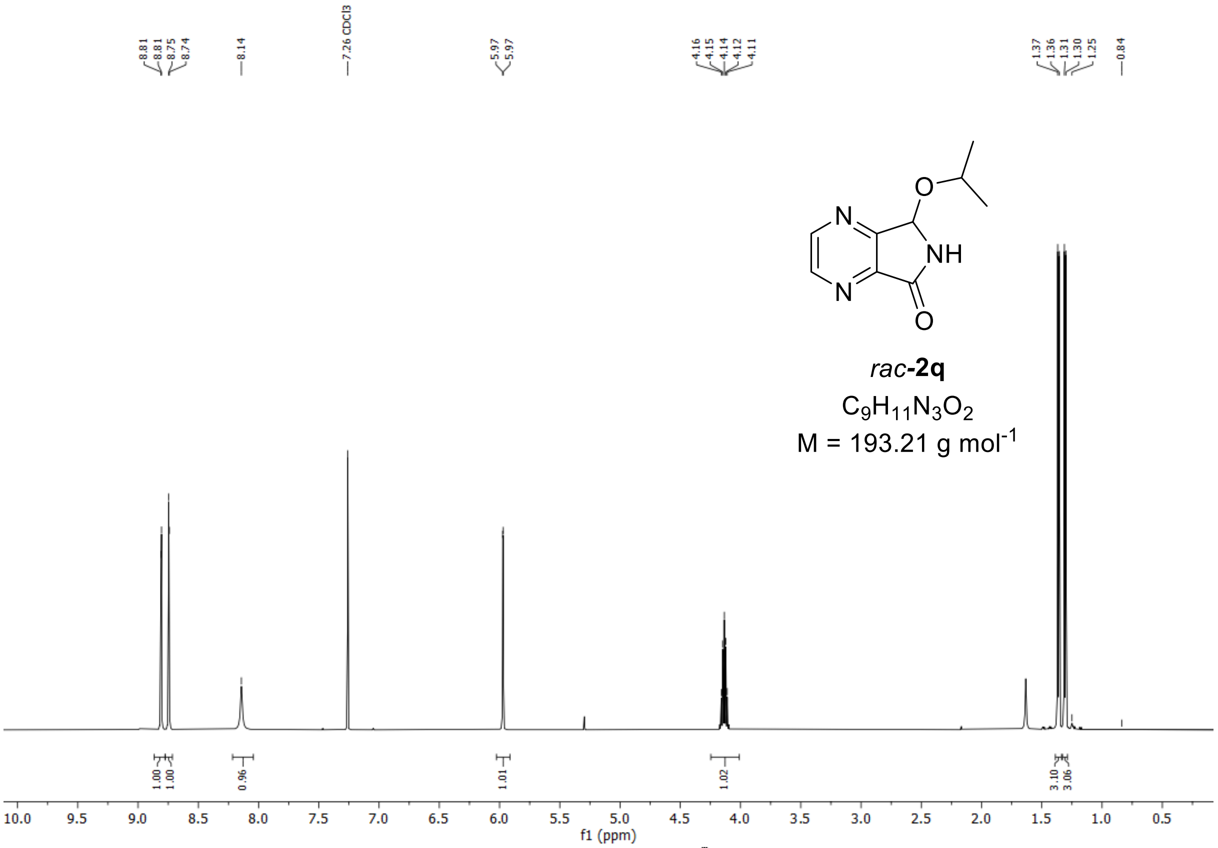

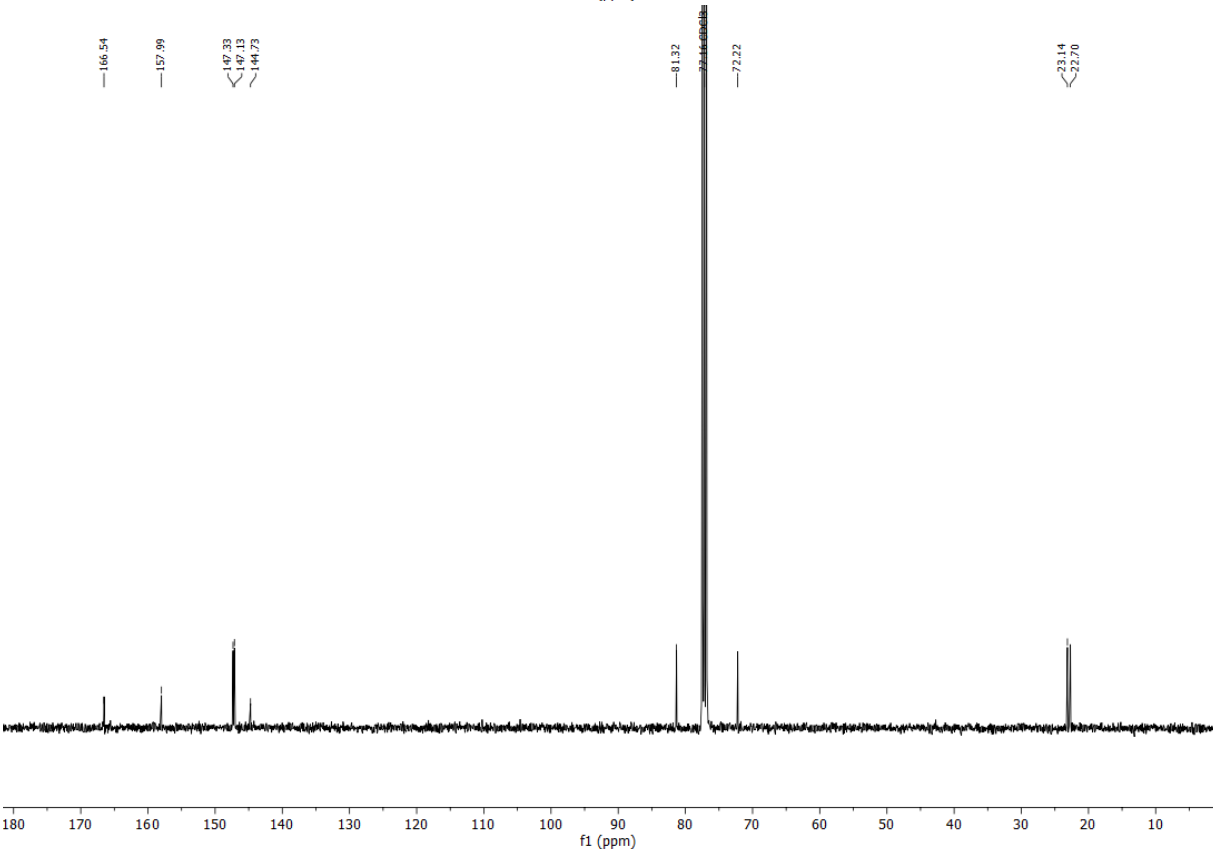

7-(3-Methoxy-3-methylbutoxy)-6,7-dihydro-5*H*-pyrrolo[3,4-*b*]pyrazin-5-one (*rac*-2r)

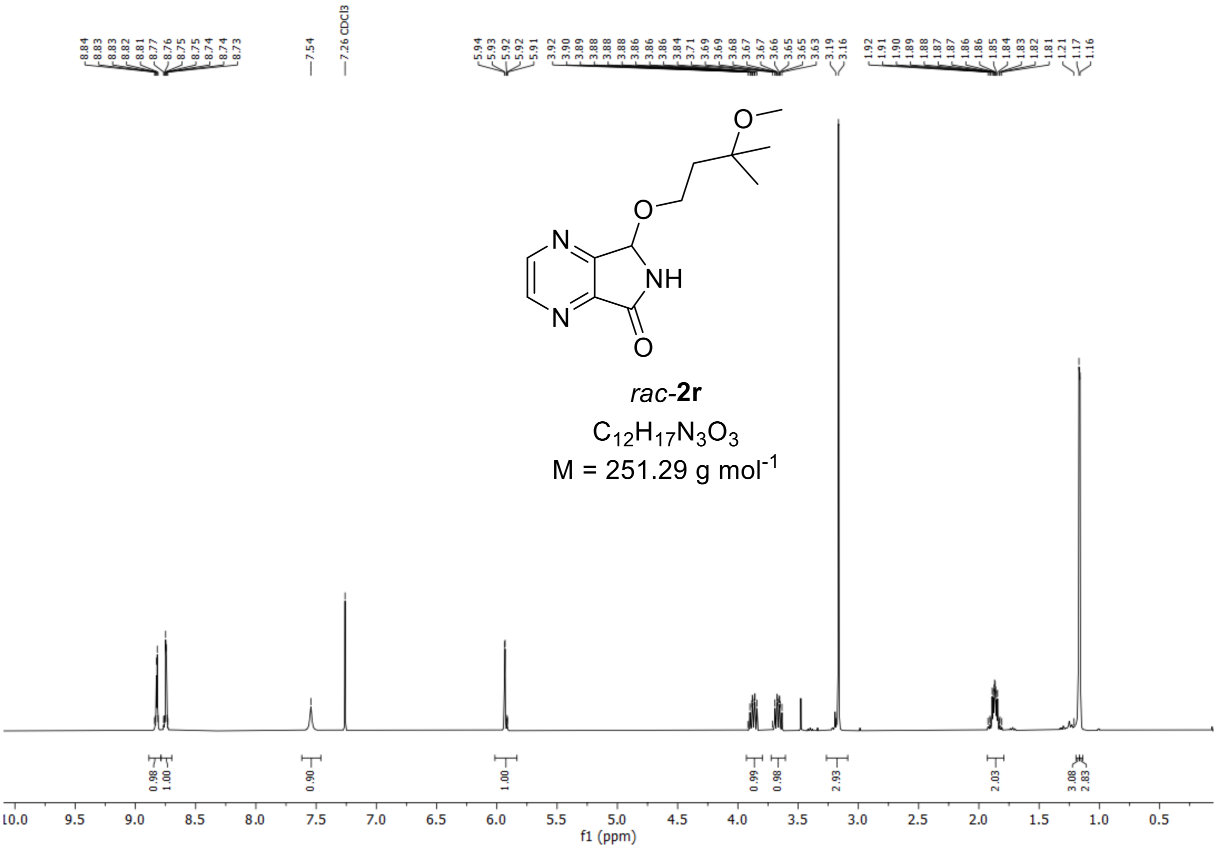

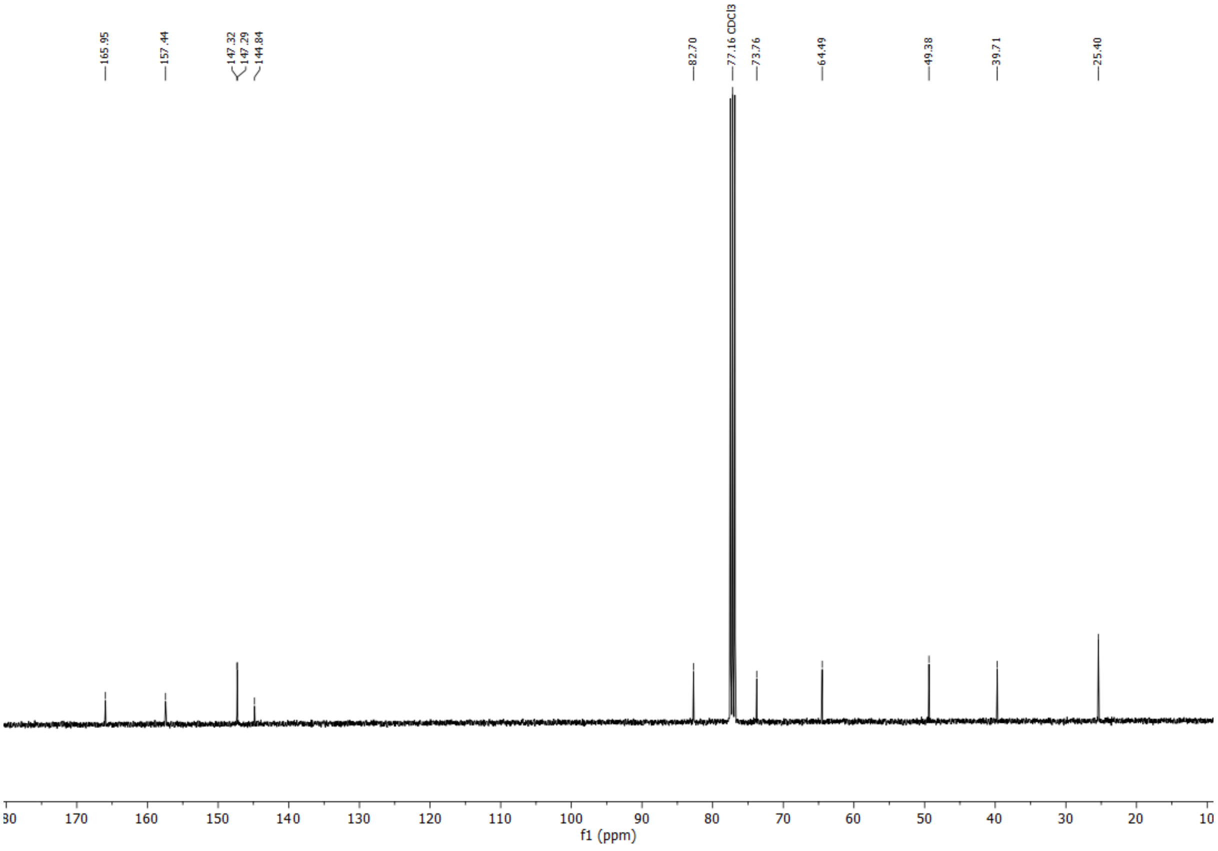

7-(2,2,2-Trifluoroethoxy)-6,7-dihydro-5*H*-pyrrolo[3,4-*b*]pyrazin-5-one (*rac*-2s)

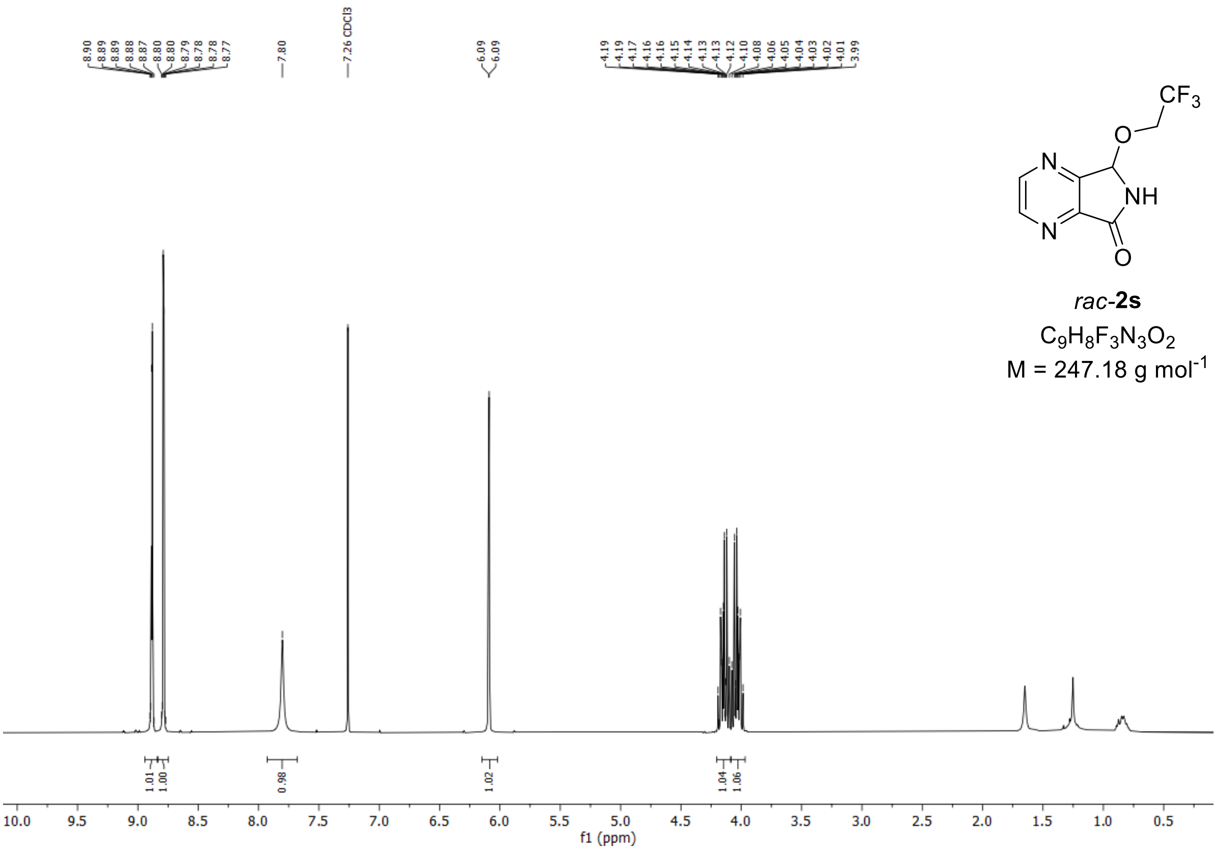

rac-**2s**

$C_9H_8F_3N_3O_2$

M = 247.18 g mol^{-1}

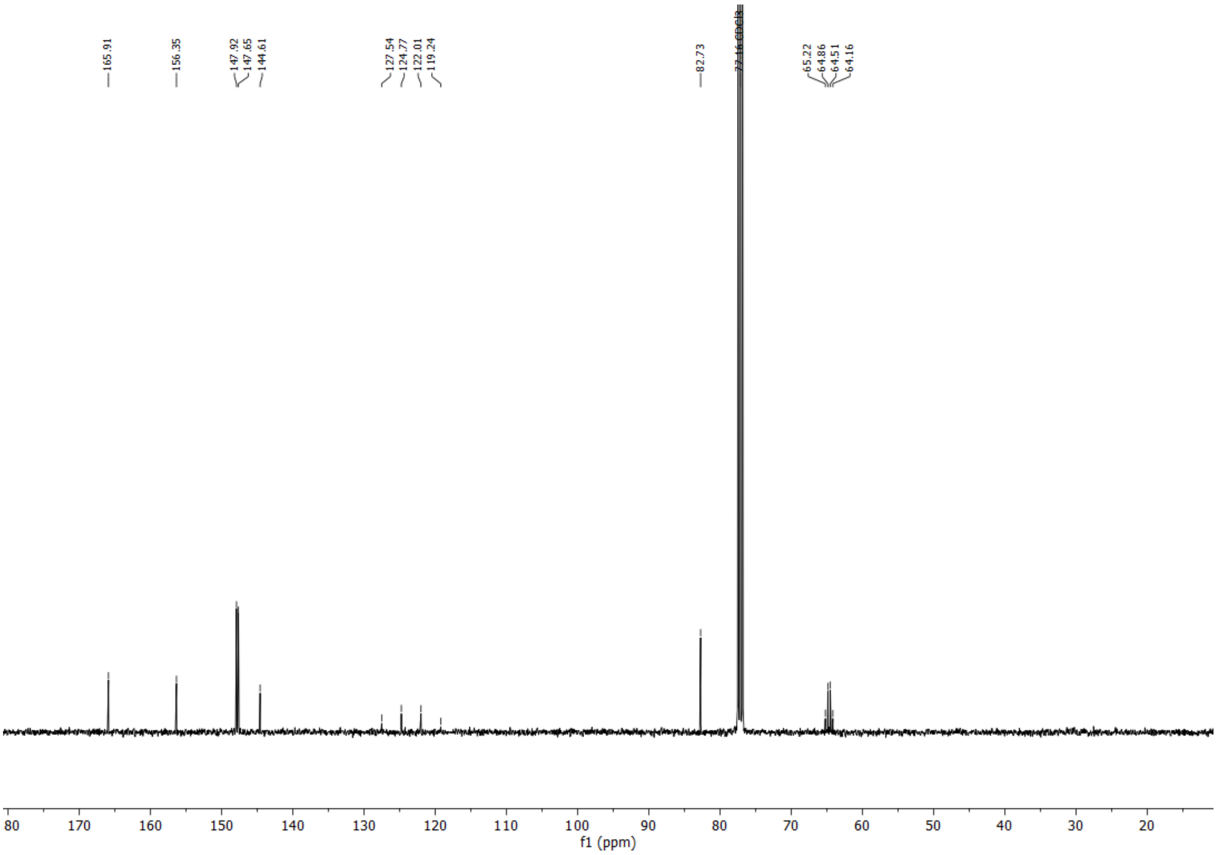

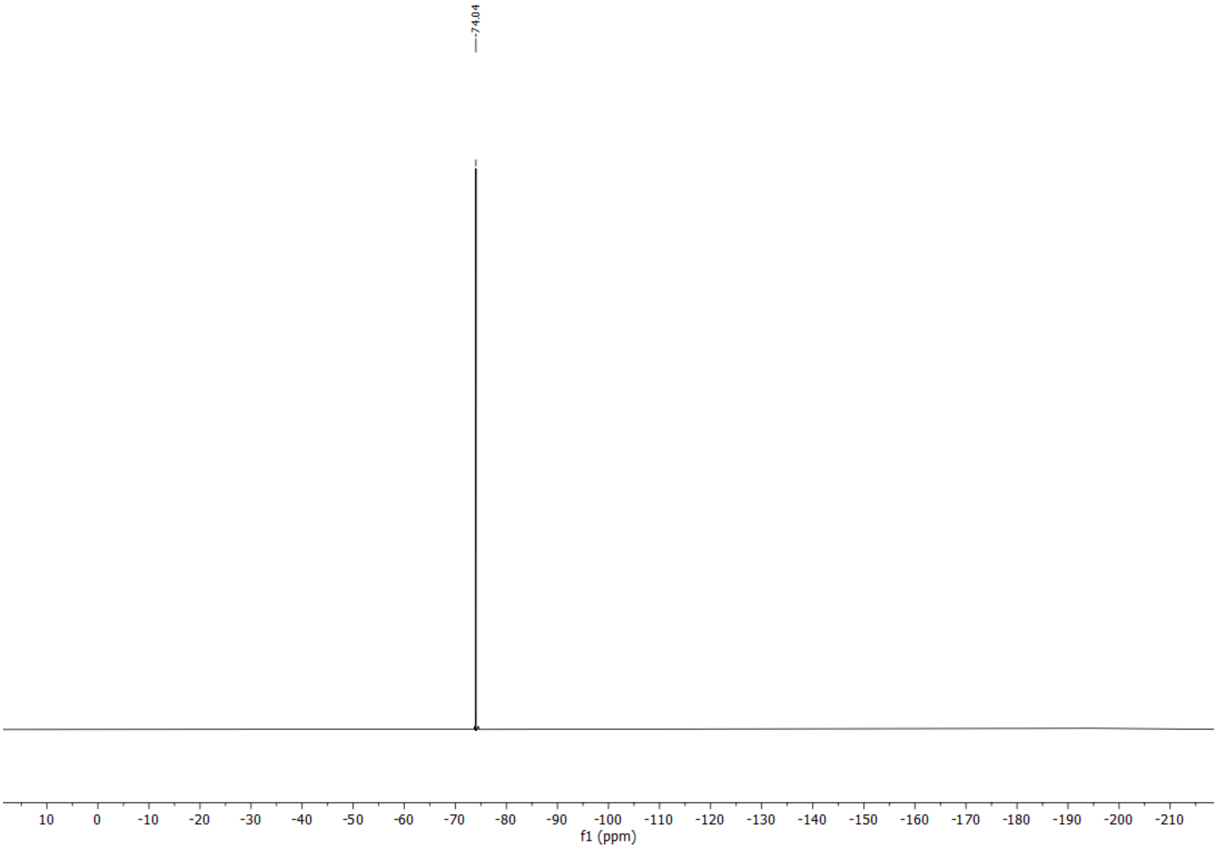

-74.04
10
0
-10
-20
-30
-40
-50
-60
-70
-80
-90
-100
-110
-120
-130
-140
-150
-160
-170
-180
-190
-200
-210
f1 (ppm)

7-((*tert*-Butyldimethylsilyl)oxy)-6,7-dihydro-5*H*-pyrrolo[3,4-*b*]pyrazin-5-one (*rac*-2t)

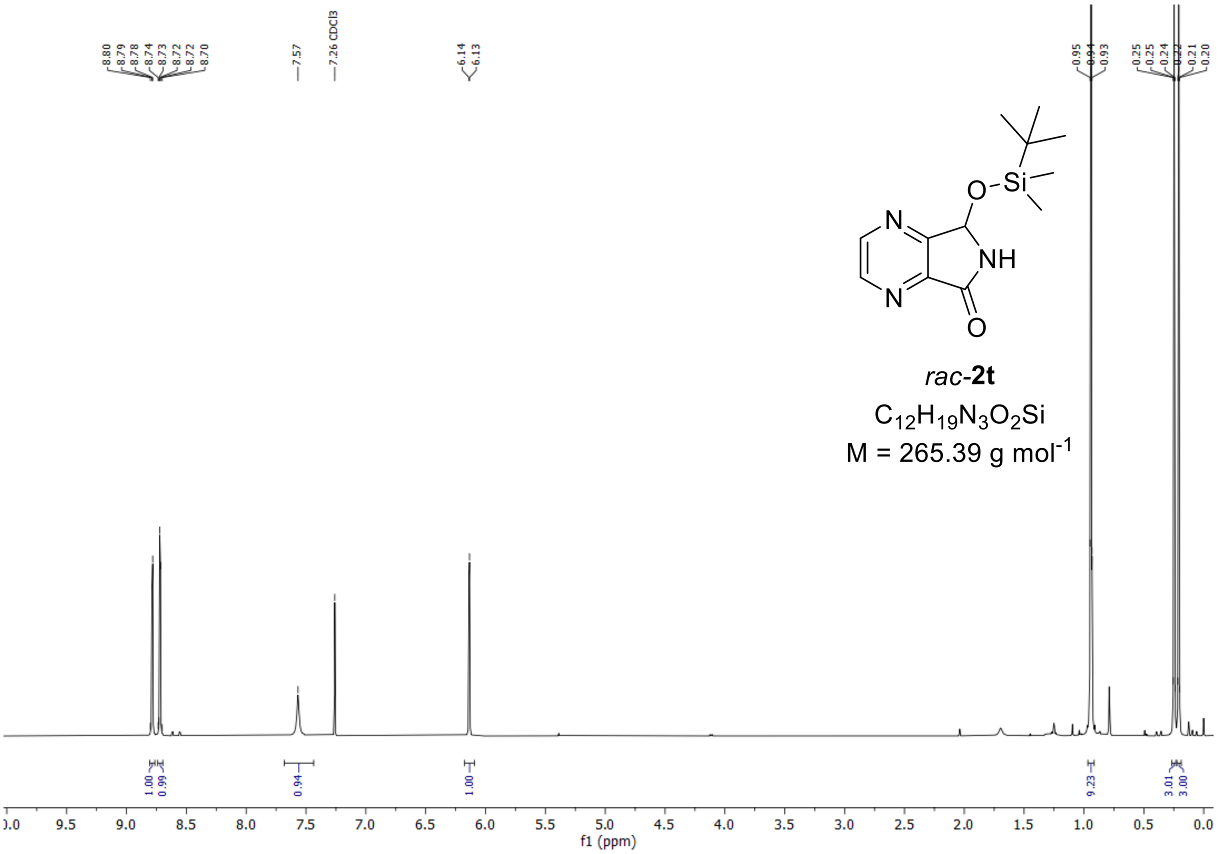

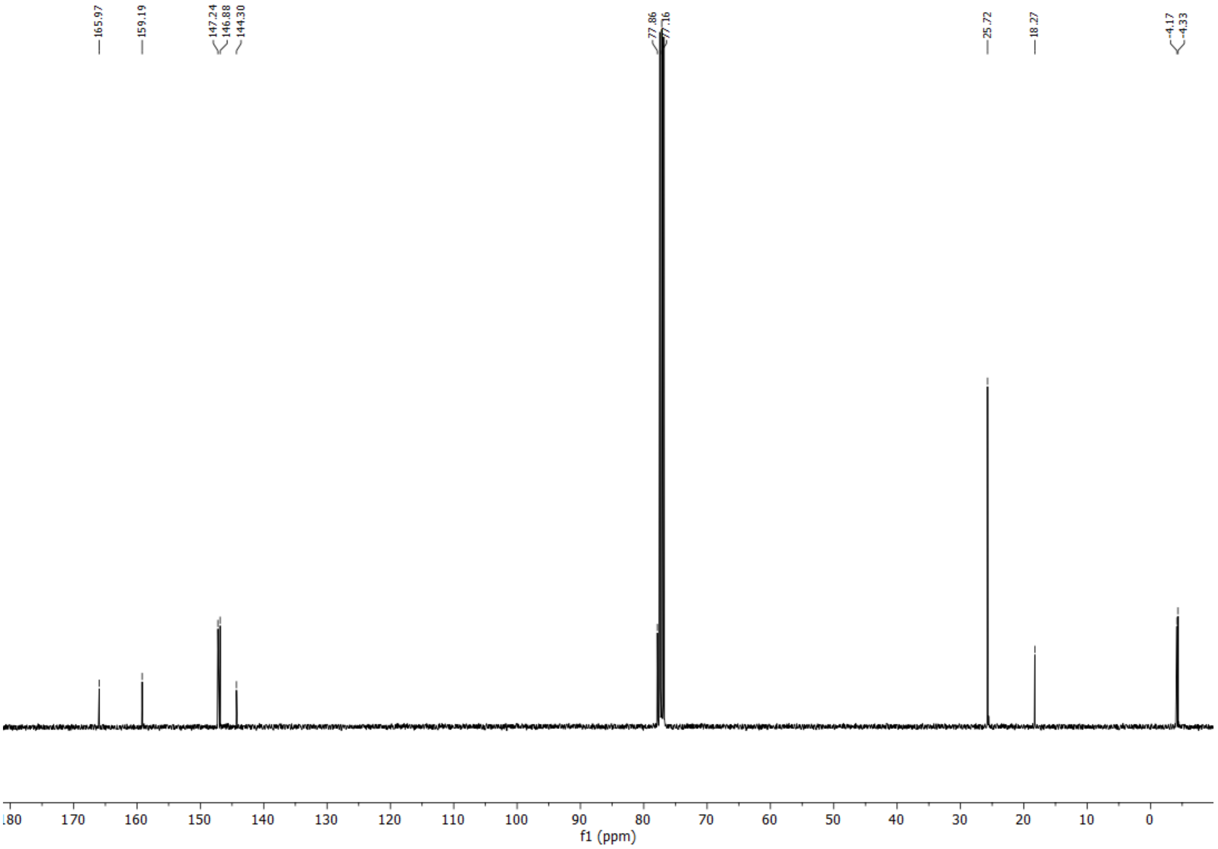

7-((Triethylsilyl)oxy)-6,7-dihydro-5*H*-pyrrolo[3,4-*b*]pyrazin-5-one (*rac*-2u)

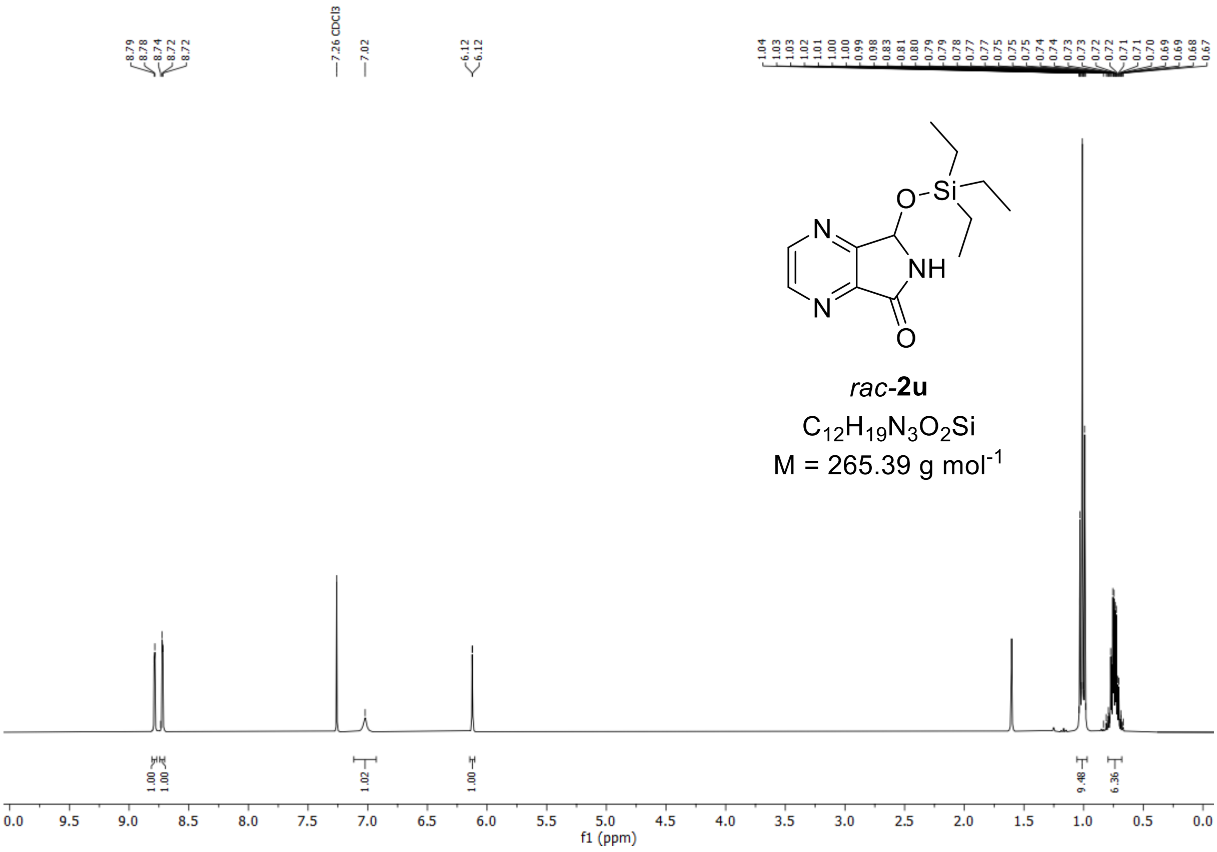

rac-**2u**

$C_{12}H_{19}N_3O_2Si$

M = 265.39 g mol^{-1}

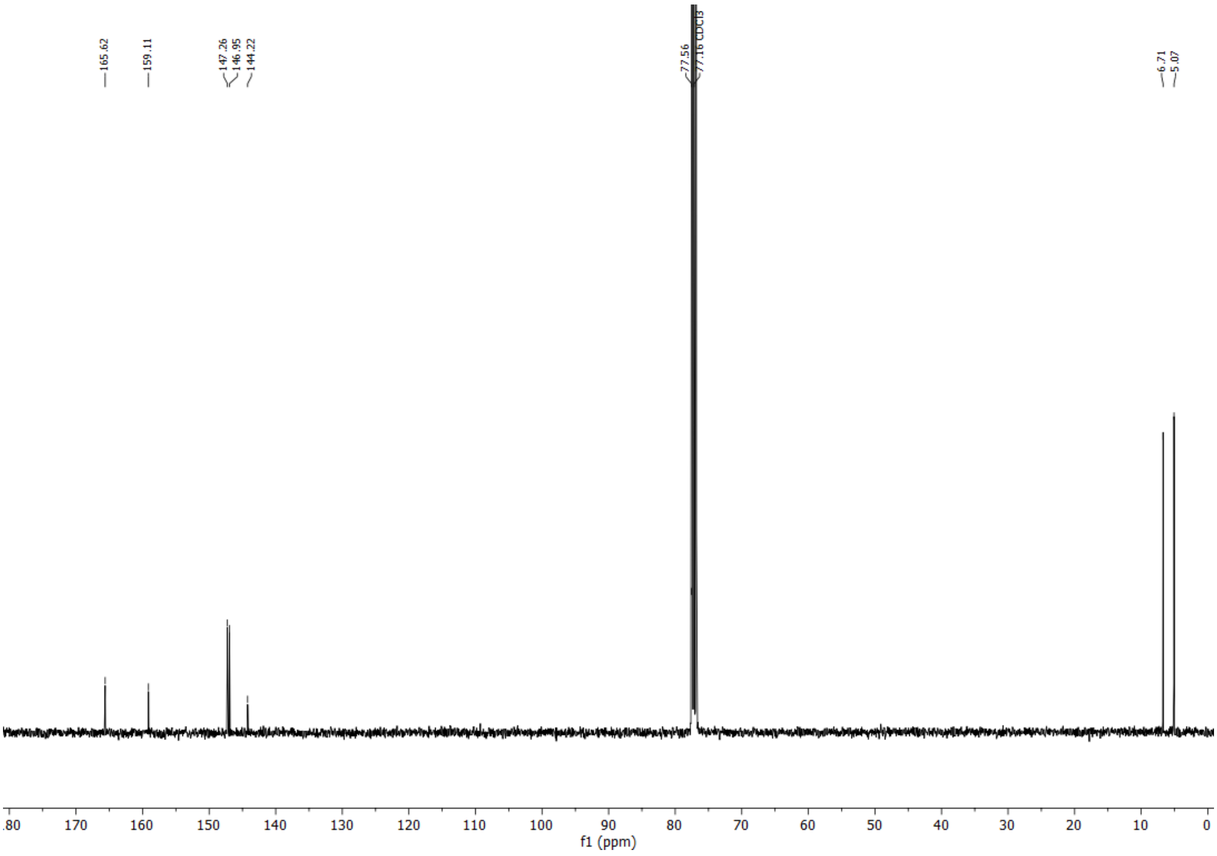

7-(4-(*tert*-Butyl)benzyl)-6-methyl-6,7-dihydro-5*H*-pyrrolo[3,4-*b*]pyrazin-5-one (*rac*-**2a**-Me)

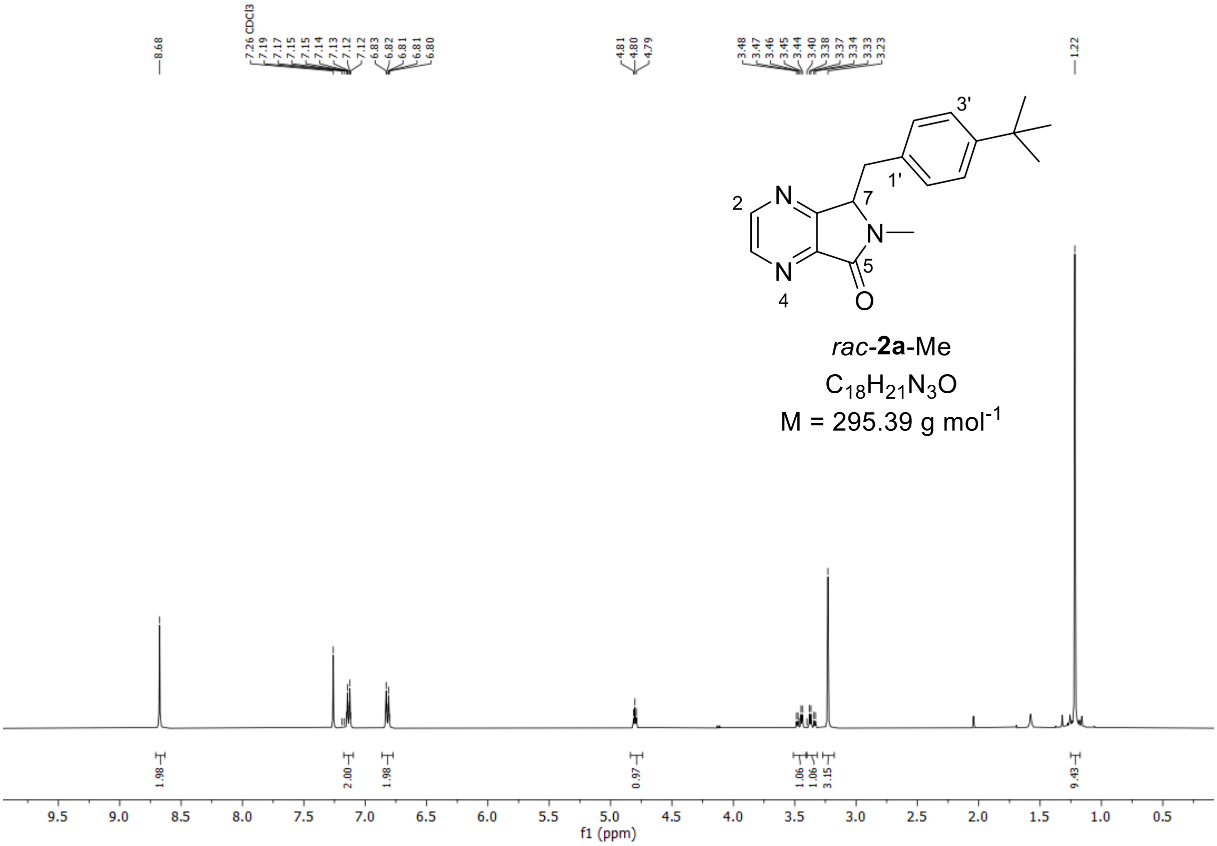

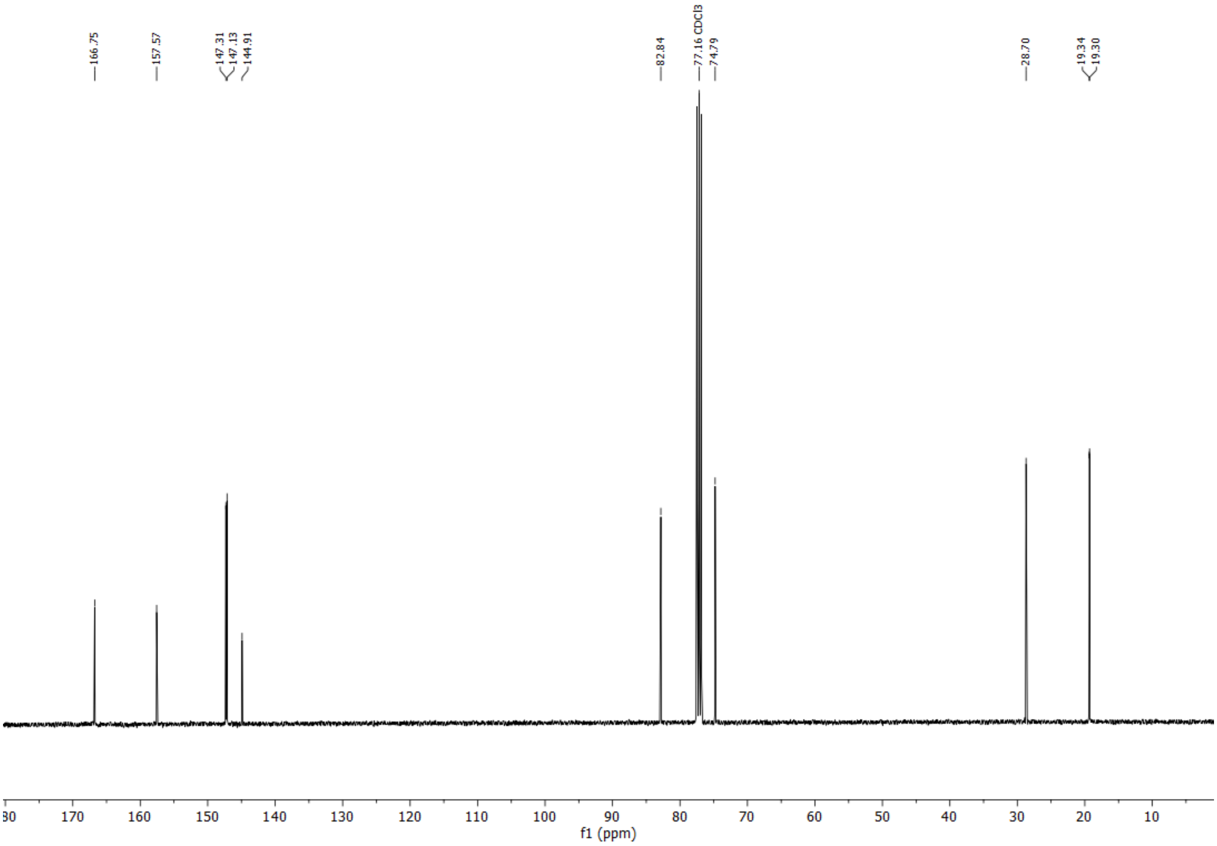

7-(4-chlorobenzyl)-6,7-dihydro-5H-pyrrolo[3,4-b]pyrazin-5-one (*rac*-SI-6)

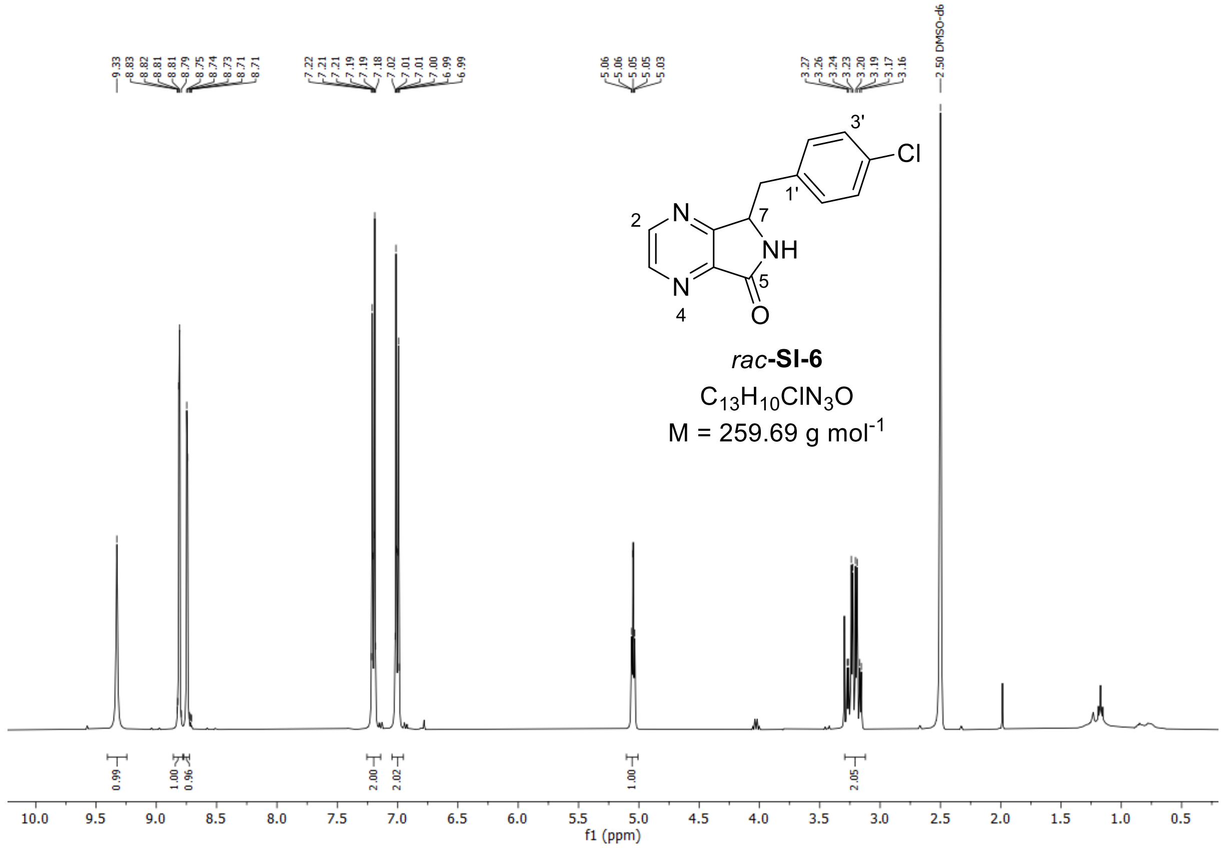

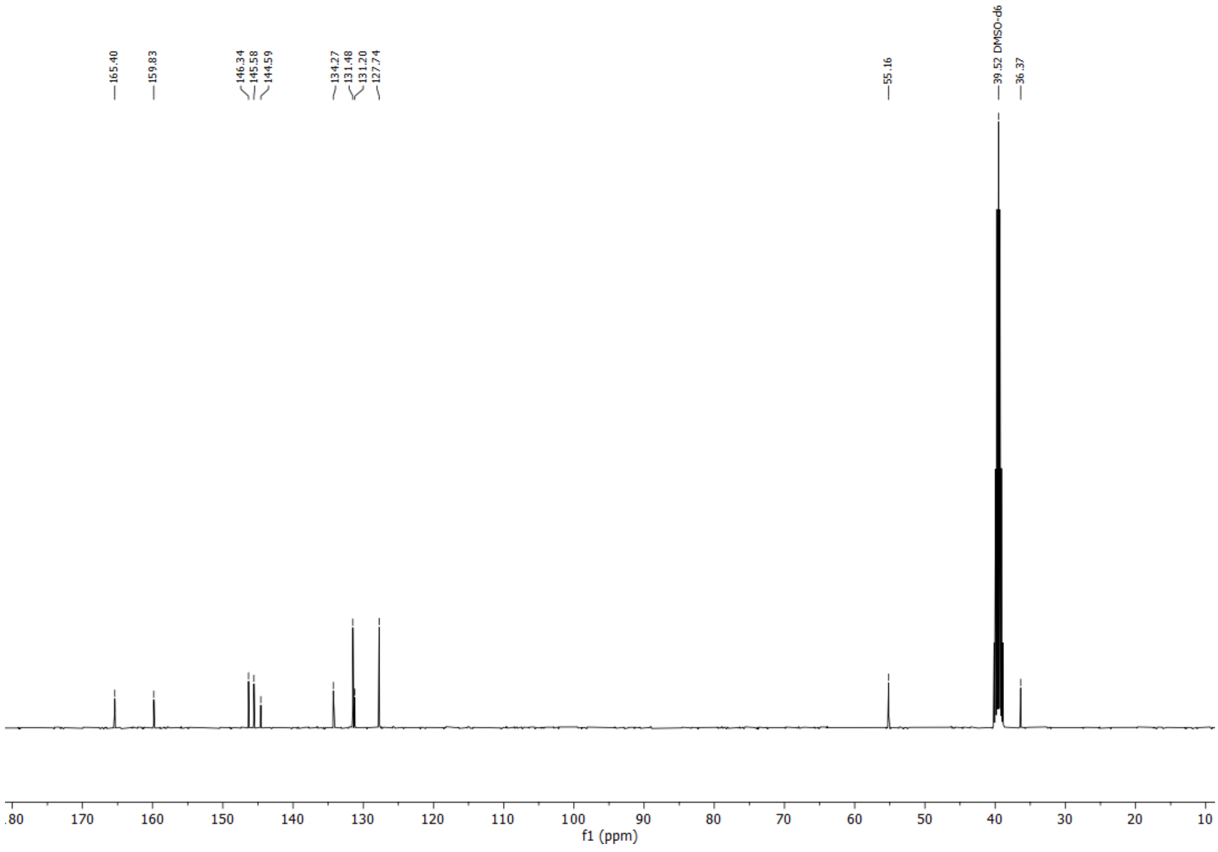

7-Phenyl-6,7-dihydro-5*H*-pyrrolo[3,4-*b*]pyrazin-5-one

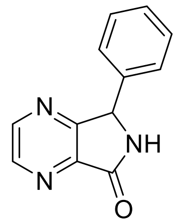

rac-**SI-7**

$C_{12}H_9N_3O$

M = 211.22 g mol^{-1}

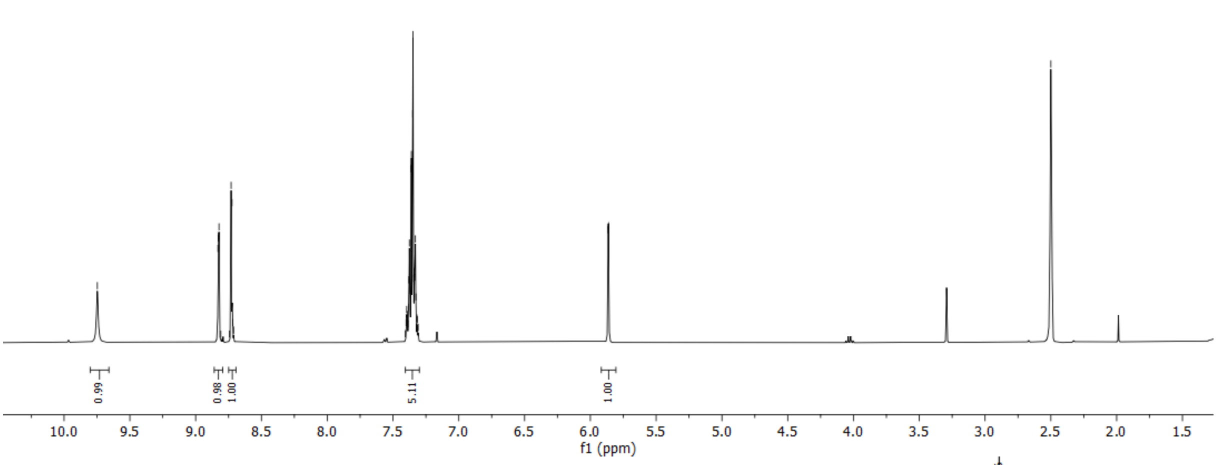

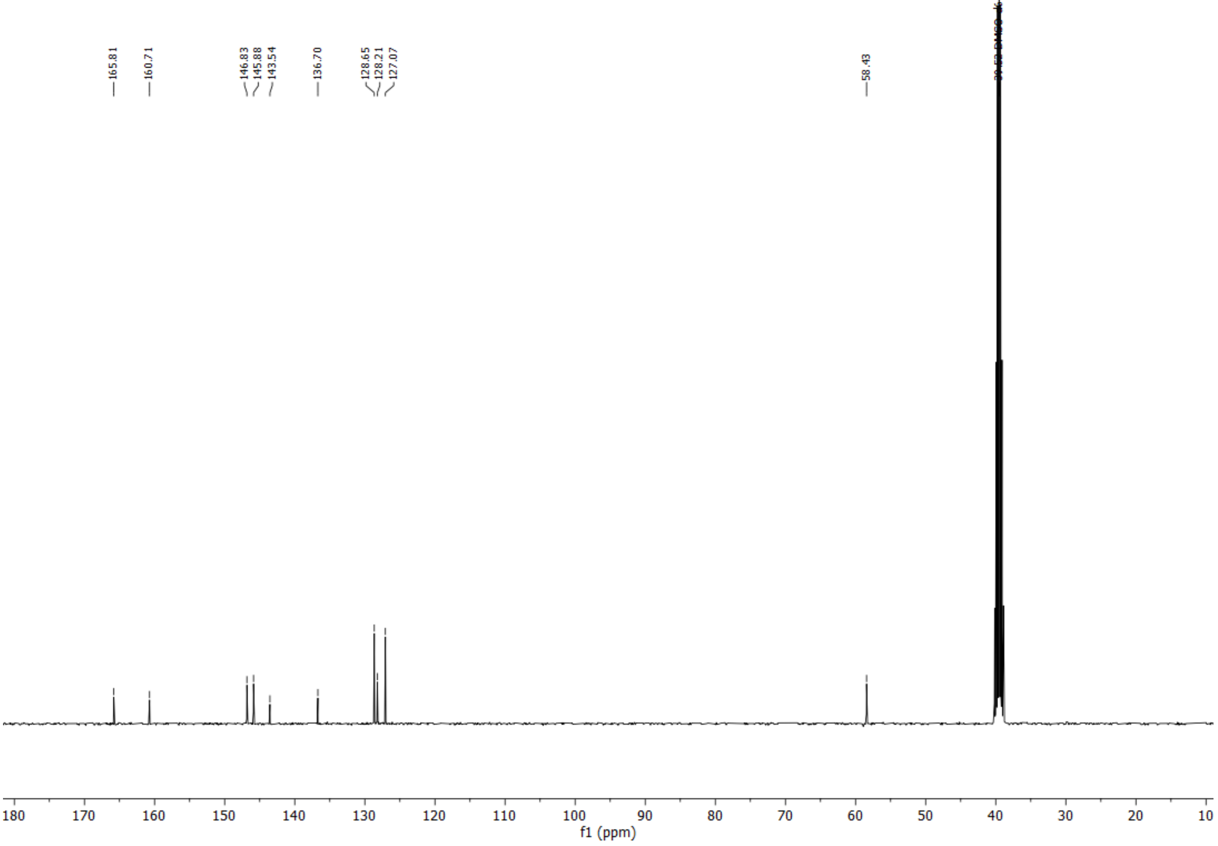

tert-Butyl (_R_)-5-(cyclopentylmethyl)-7-oxo-5,7-dihydro-6_H_-pyrrolo[3,4-_b_]pyrazine-6-carboxylate (6)

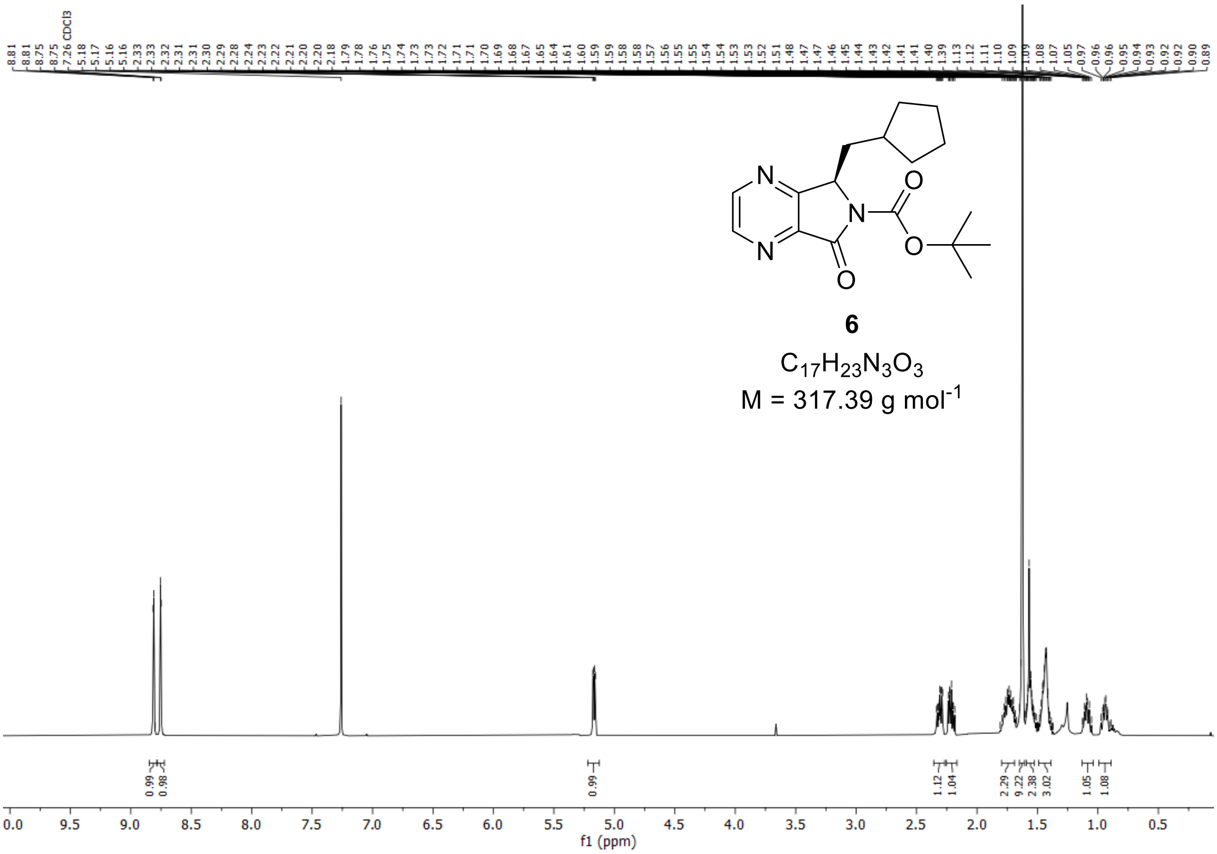

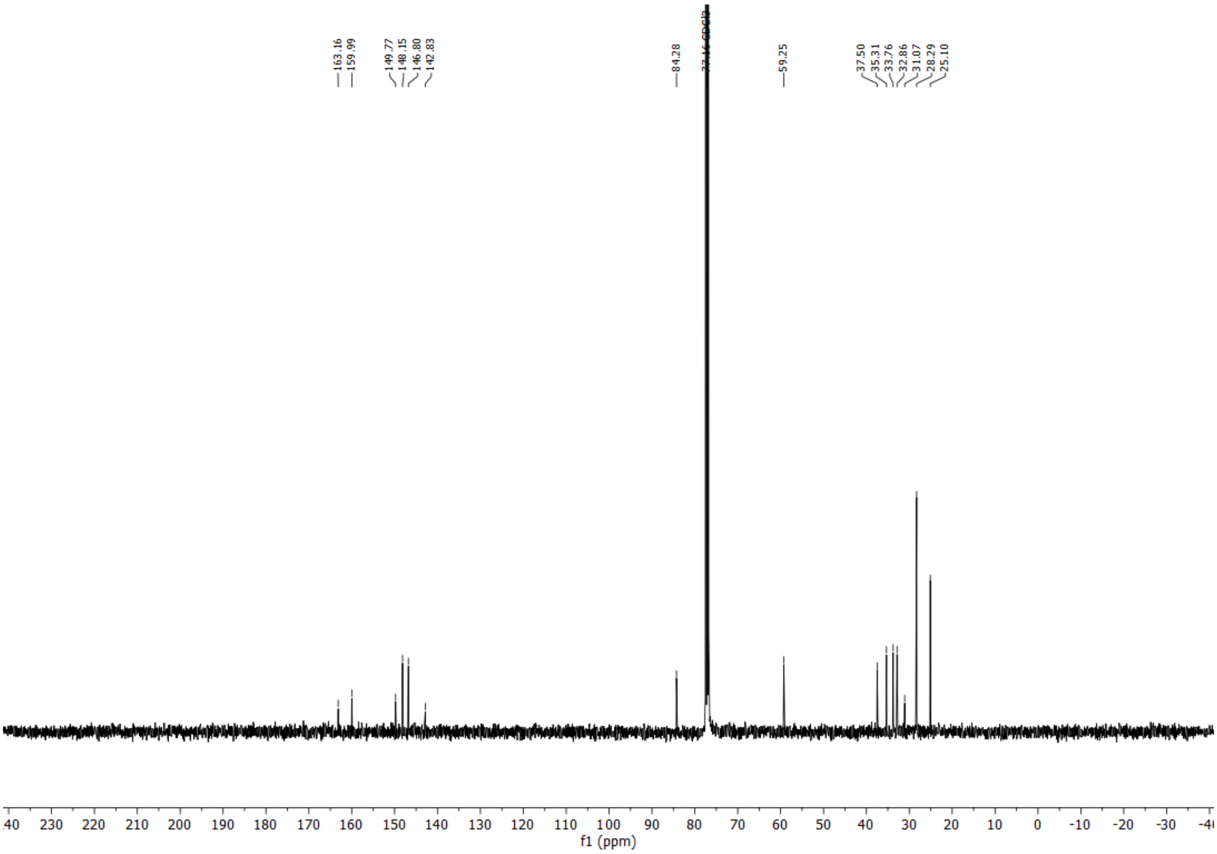

(*R*)-3-(1-((*tert*-Butoxycarbonyl)amino)-2-cyclopentylethyl)pyrazine-2-carboxylic acid (7)

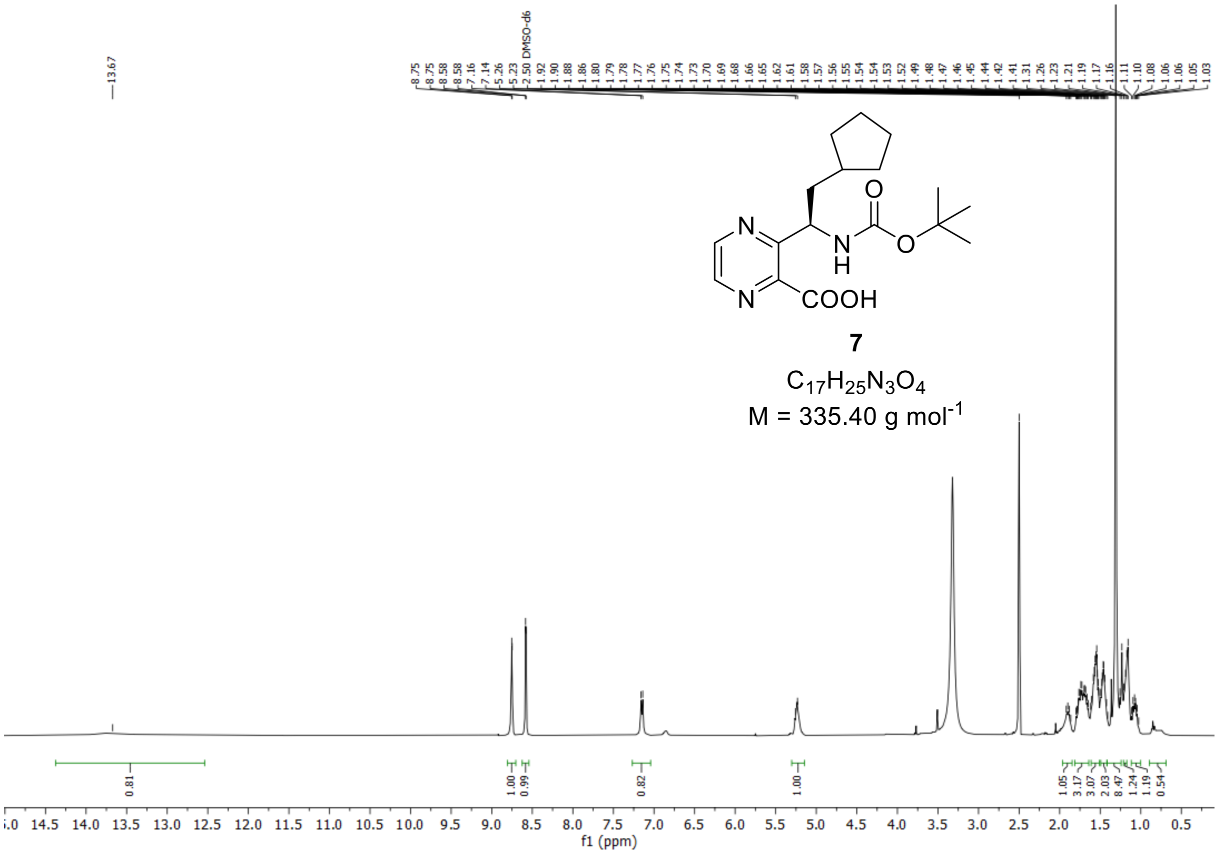

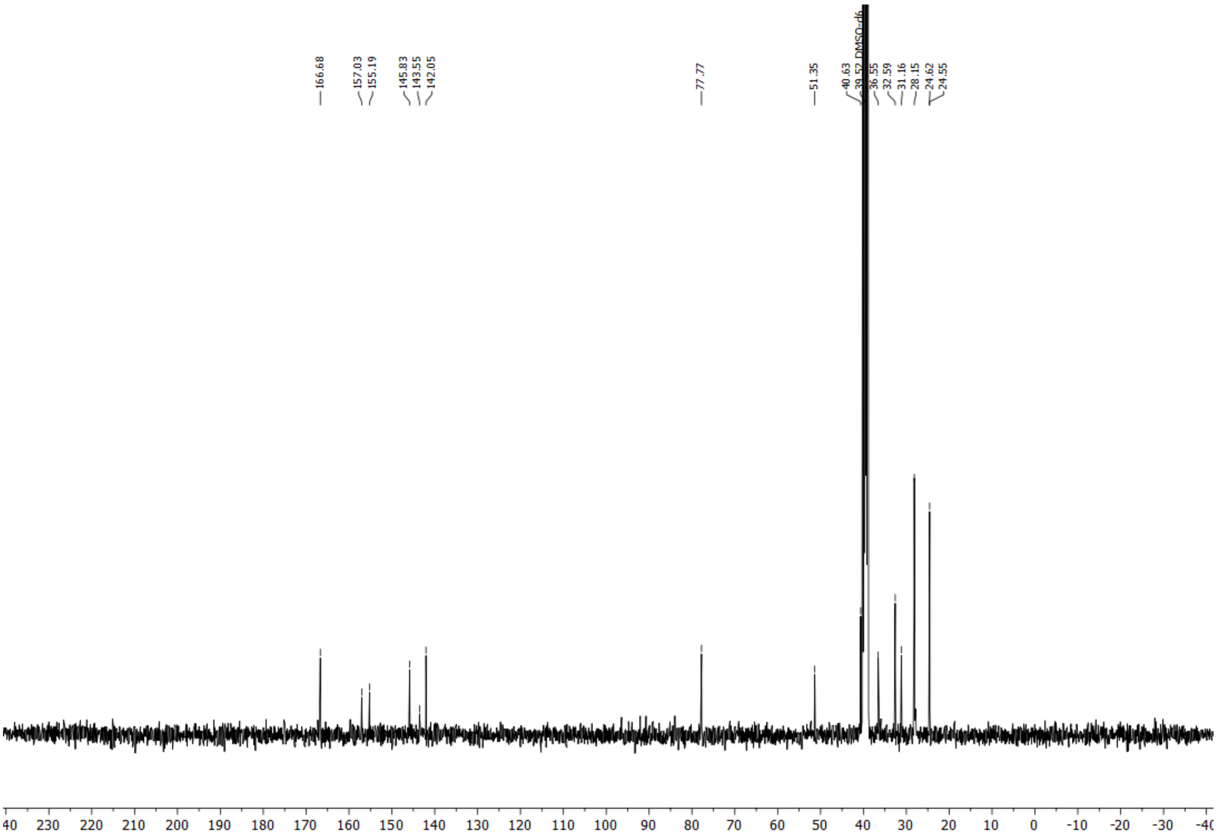

tert-Butyl (R)-(2-cyclopentyl-1-(pyrazin-2-yl)ethyl)carbamate (8)

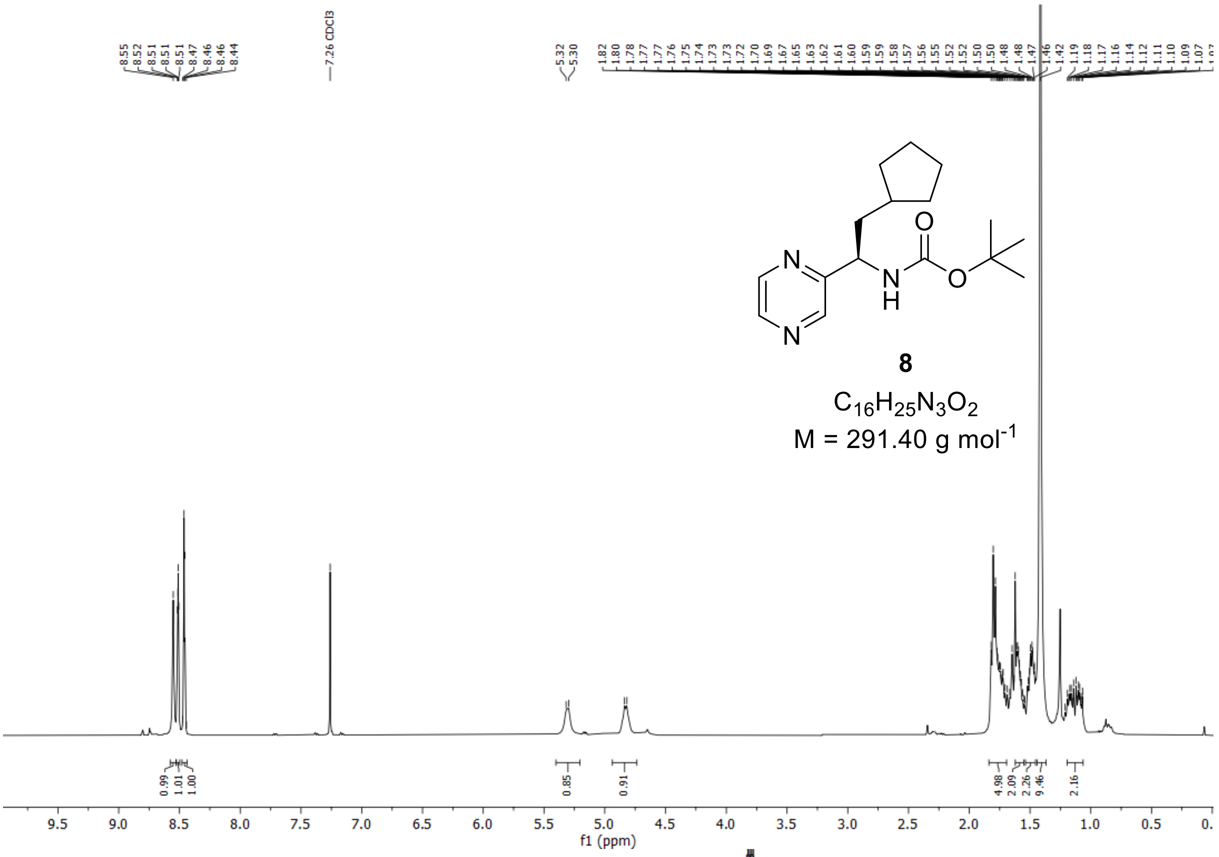

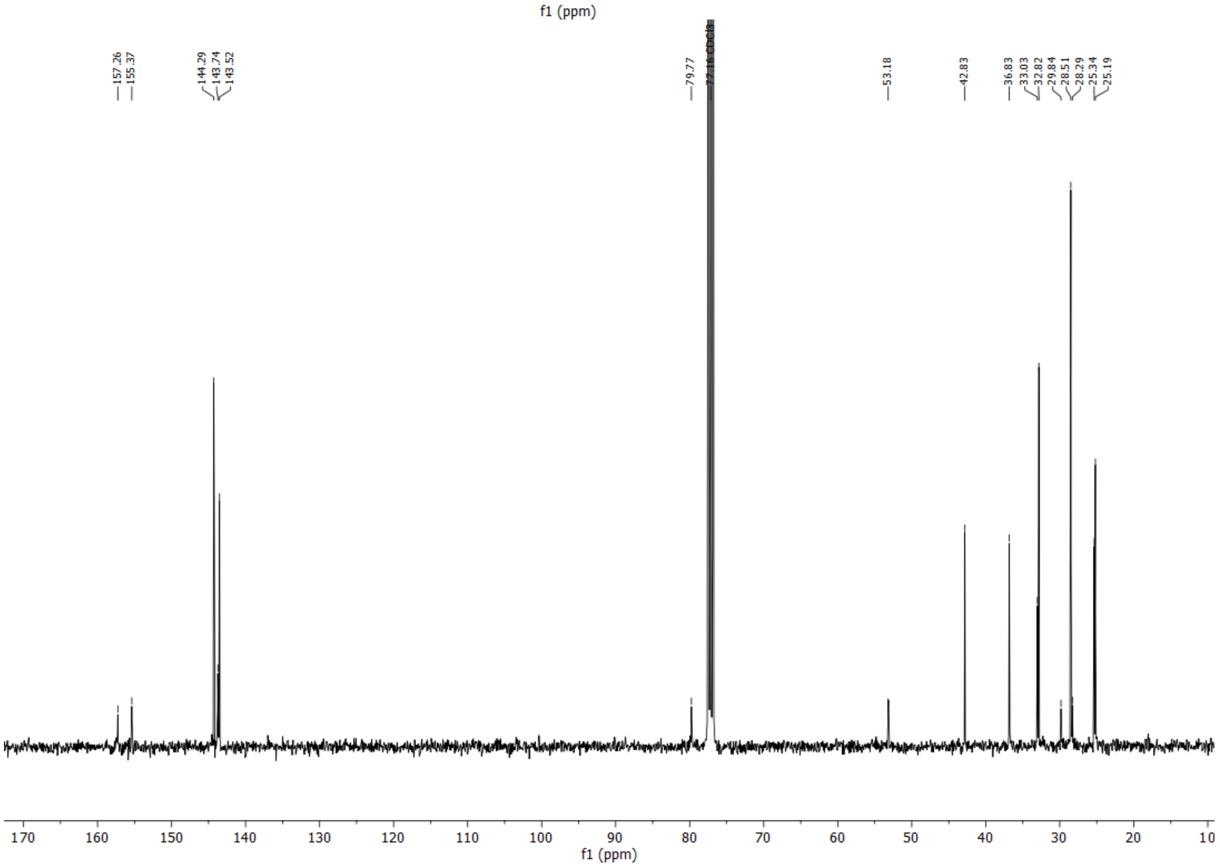

Methyl (*R*)-3-(1-((*tert*-butoxycarbonyl)amino)-2-cyclopentylethyl)pyrazine-2-carboxylate (9)

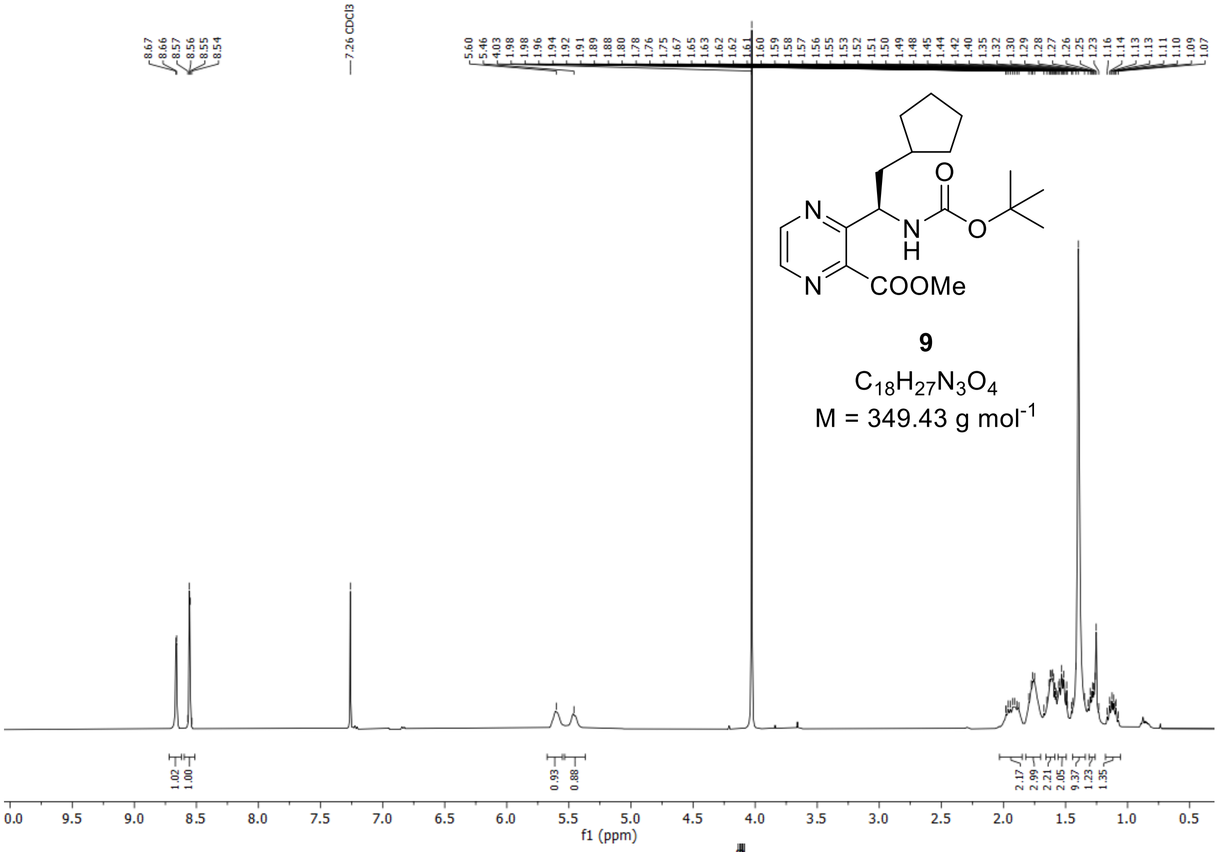

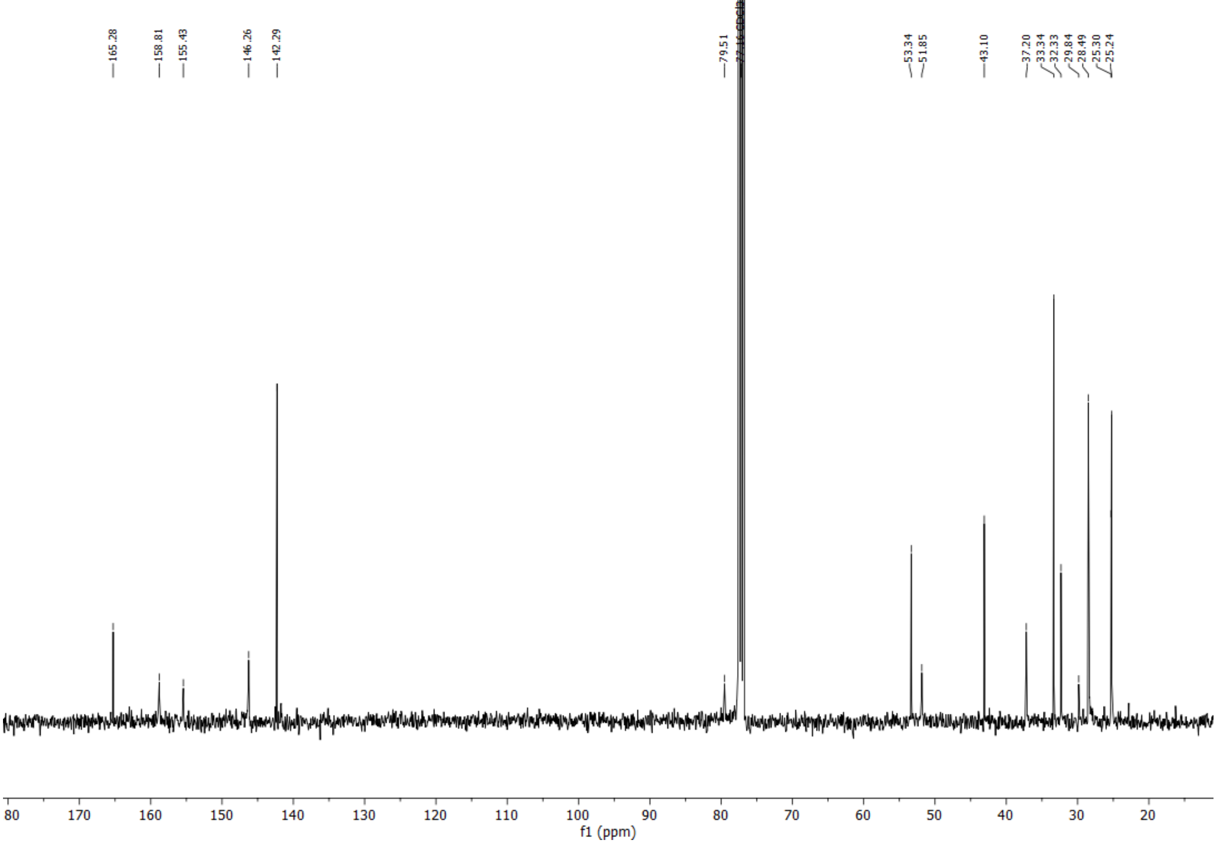

(*R*)-6,7-Dihydrooxazolo[3',2':1,2]pyrrolo[3,4-*b*]pyrazin-9(4*bH*)-one (10)

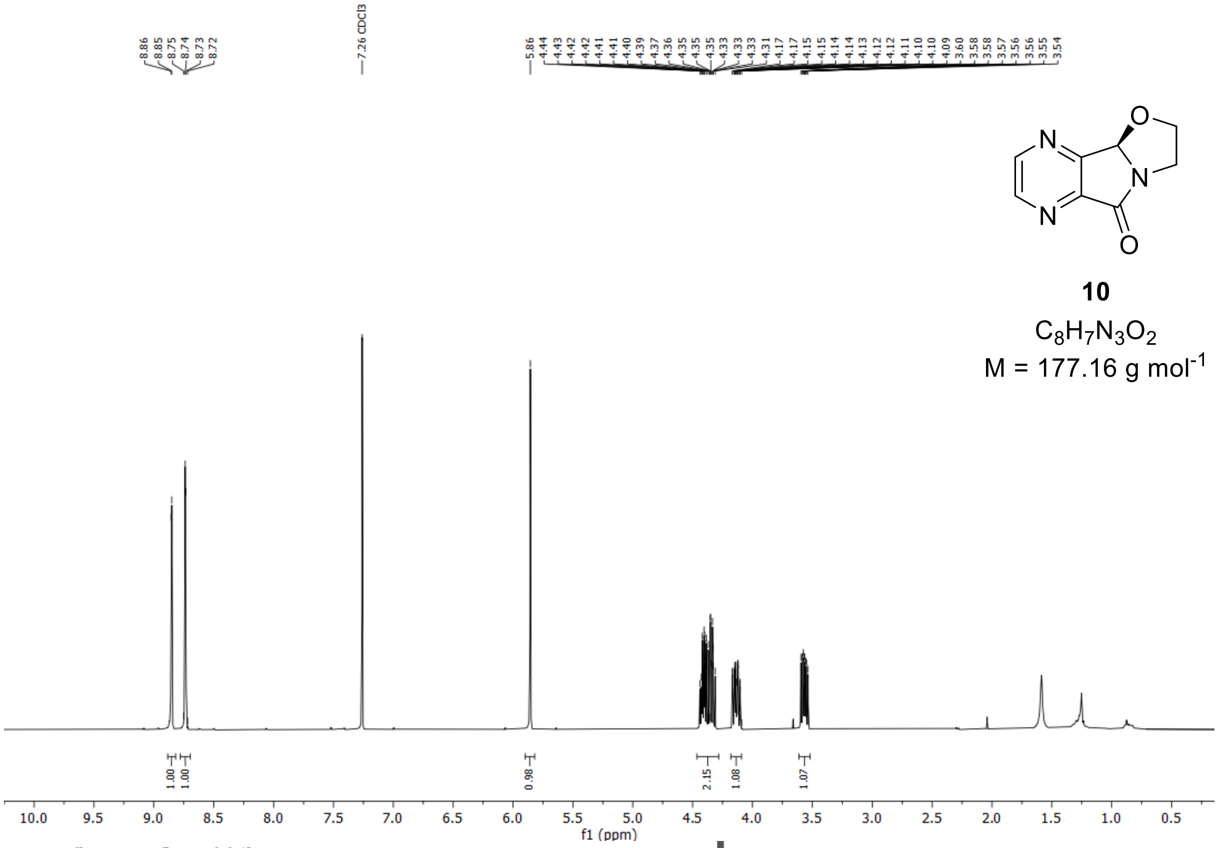

10

$C_8H_7N_3O_2$

M = 177.16 g mol^{-1}

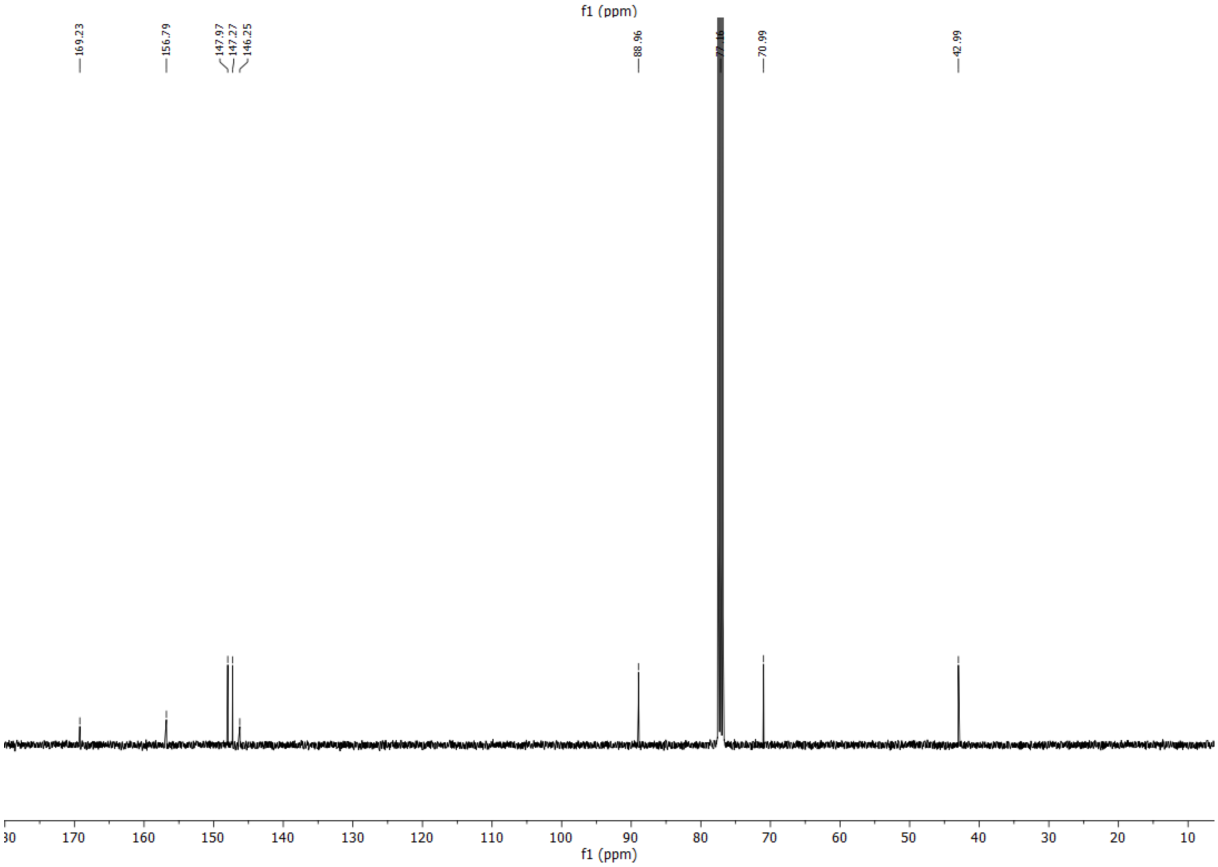

(*R*)-7-((*tert*-Butyldimethylsilyl)oxy)-6-(5-chloropyridin-2-yl)-6,7-dihydro-5*H*-pyrrolo[3,4-*b*]pyrazin-5-one (11)

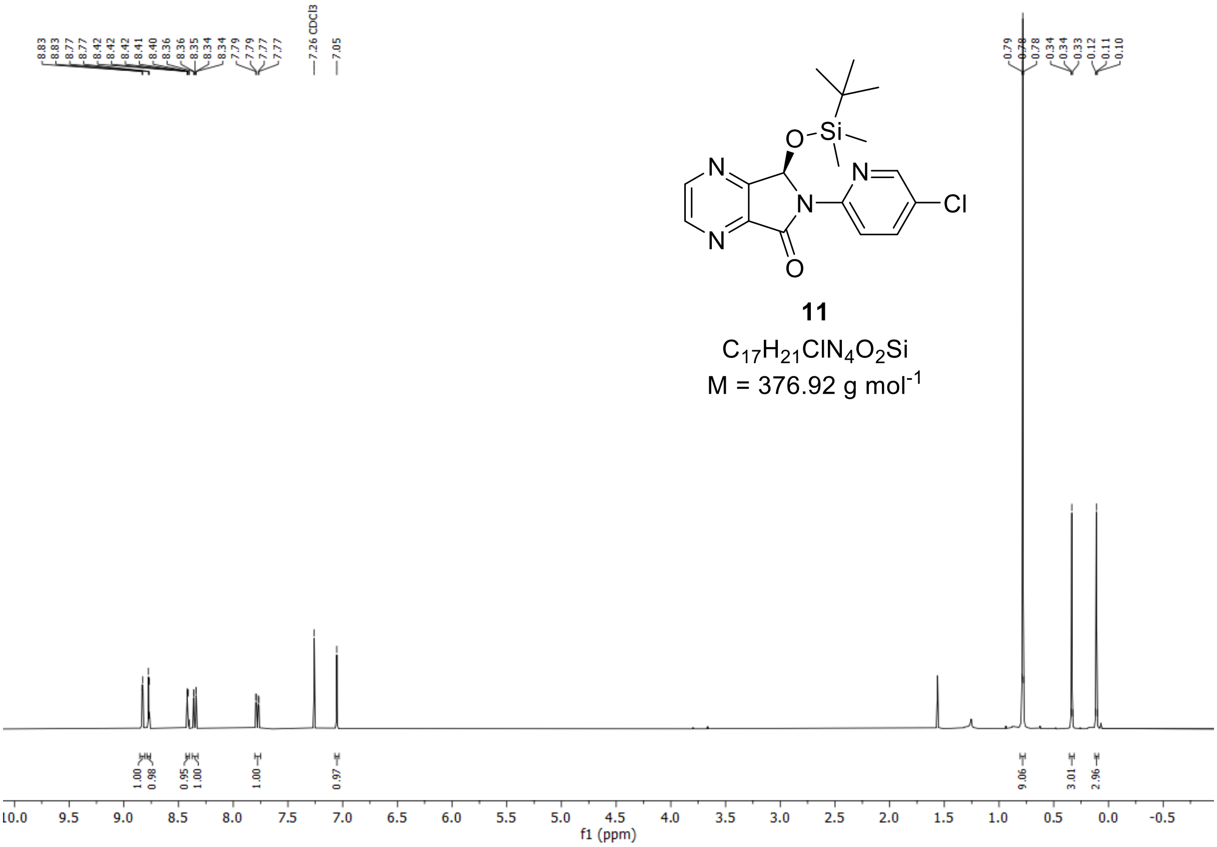

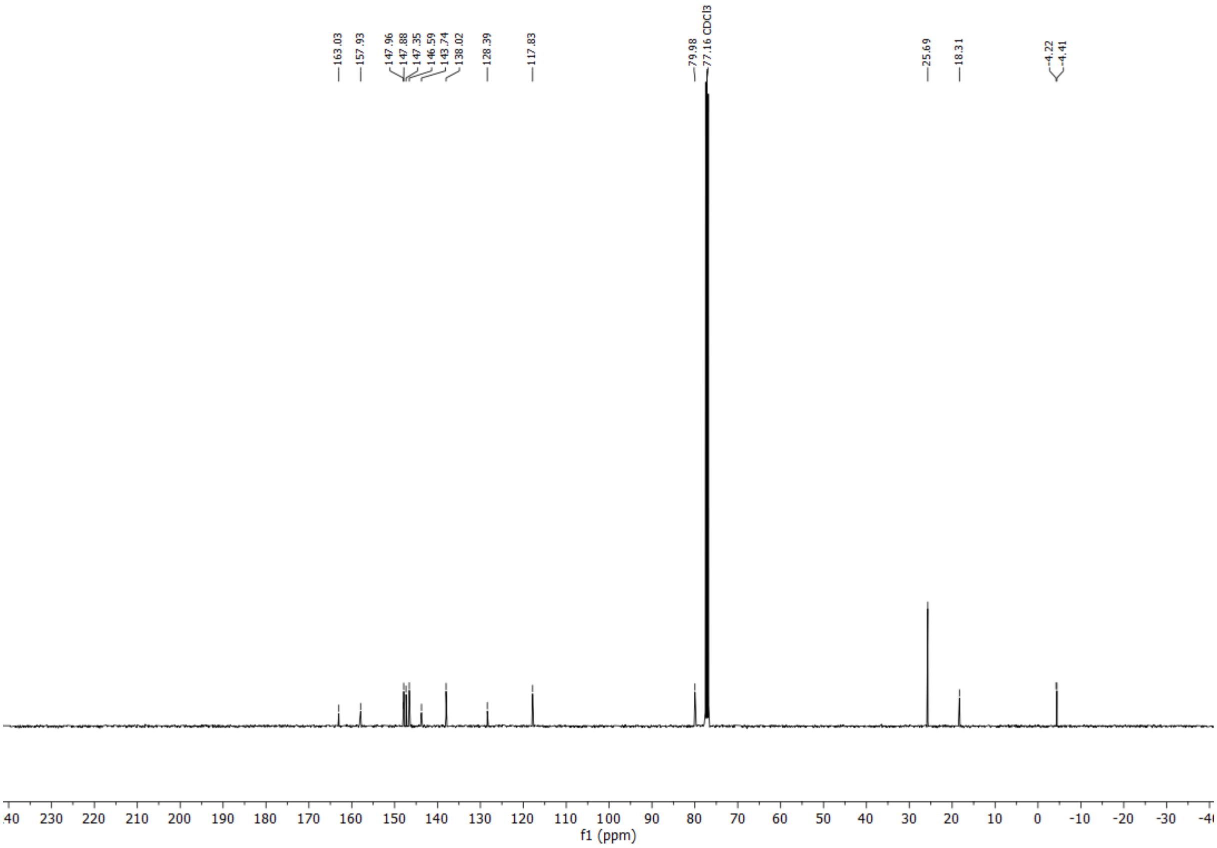

15. Chiral HPLC Traces

(*R*)-7-(4-(*tert*-Butyl)benzyl)-6,7-dihydro-5*H*-pyrrolo[3,4-*b*]pyrazin-5-one (2a)

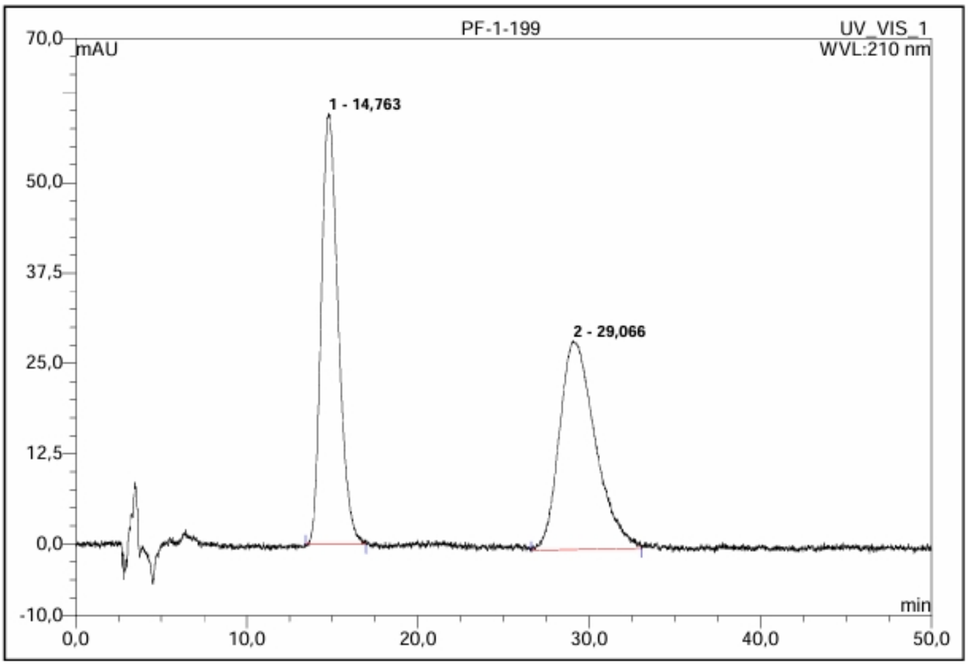

No.	Ret.Time min	Peak Name	Height mAU	Area mAU*min	Rel.Area %	Amount	Type
1	14,76	n.a.	59,723	68,629	49,40	n.a.	BMB*
2	29,07	n.a.	28,974	70,288	50,60	n.a.	BMB*
Total:			88,697	138,917	100,00	0,000	

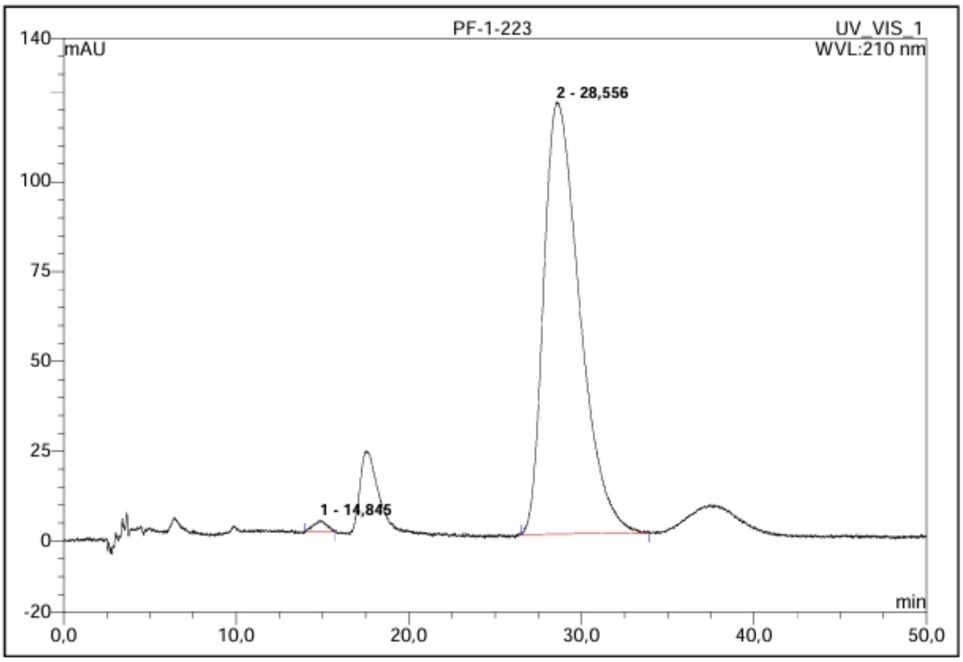

No.	Ret.Time min	Peak Name	Height mAU	Area mAU*min	Rel.Area %	Amount	Type
1	14,84	n.a.	3,358	2,918	0,99	n.a.	BMB*
2	28,56	n.a.	120,368	290,428	99,01	n.a.	BMB*
Total:			123,726	293,345	100,00	0,000	

(*R*)-7-Benzyl-6,7-dihydro-5*H*-pyrrolo[3,4-*b*]pyrazin-5-one (2b)

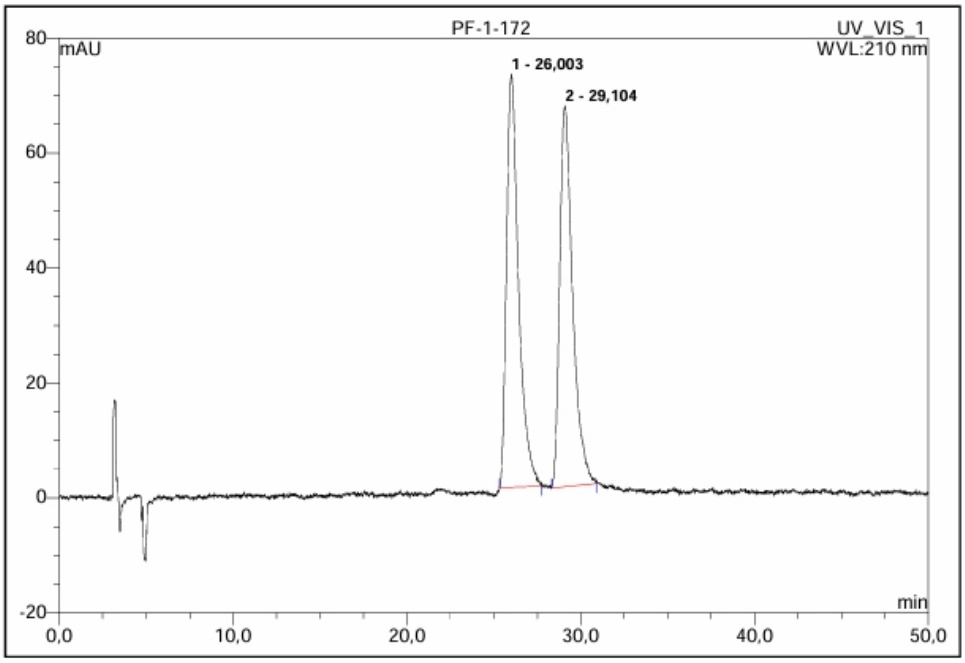

No.	Ret.Time min	Peak Name	Height mAU	Area mAU*min	Rel.Area %	Amount	Type
1	26,00	n.a.	71,846	57,681	50,24	n.a.	BMB*
2	29,10	n.a.	66,252	57,134	49,76	n.a.	BMB*
Total:			138,097	114,814	100,00	0,000	

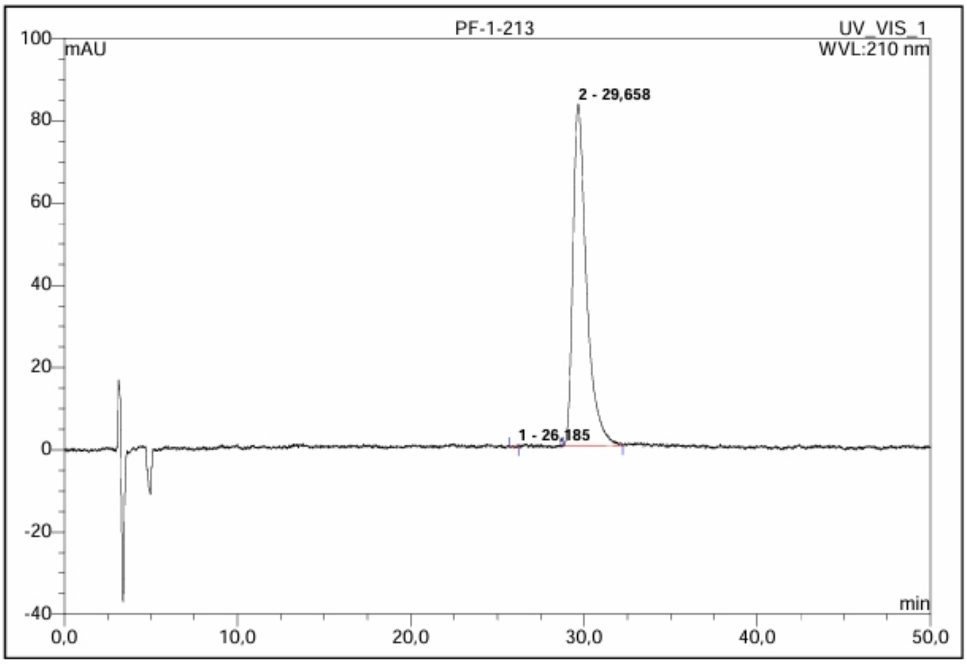

No.	Ret.Time min	Peak Name	Height mAU	Area mAU*min	Rel.Area %	Amount	Type
1	26,18	n.a.	0,464	0,022	0,03	n.a.	BMB*
2	29,66	n.a.	83,096	75,545	99,97	n.a.	BMB*
Total:			83,560	75,567	100,00	0,000	

(*R*)-7-(4-(Trifluoromethyl)benzyl)-6,7-dihydro-5*H*-pyrrolo[3,4-*b*]pyrazin-5-one (2c)

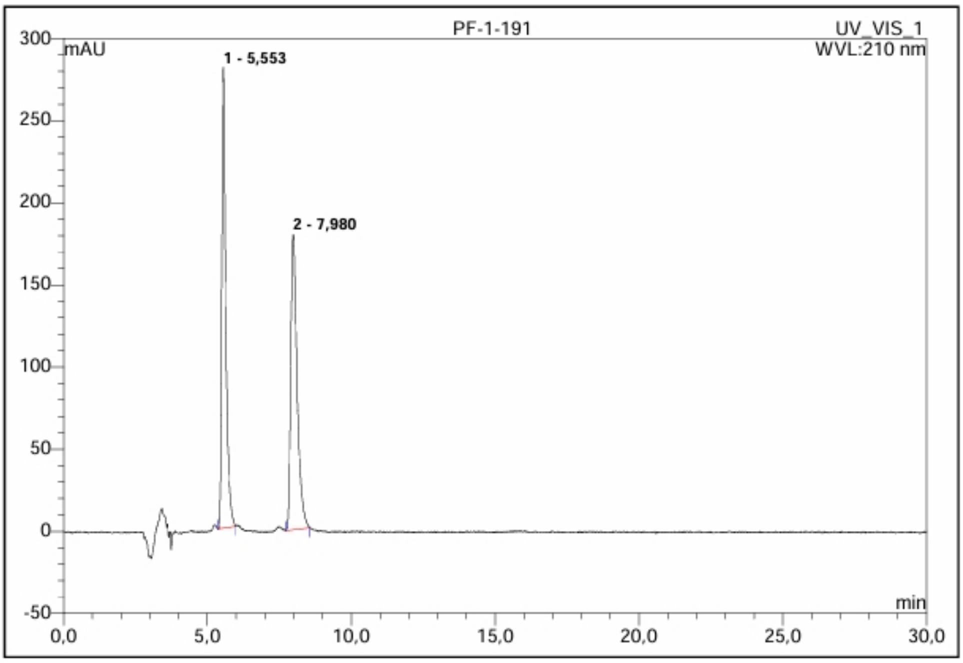

No.	Ret.Time min	Peak Name	Height mAU	Area mAU*min	Rel.Area %	Amount	Type
1	5,55	n.a.	280,663	45,537	49,77	n.a.	BMB
2	7,98	n.a.	179,802	45,952	50,23	n.a.	BMB
Total:			460,465	91,489	100,00	0,000	

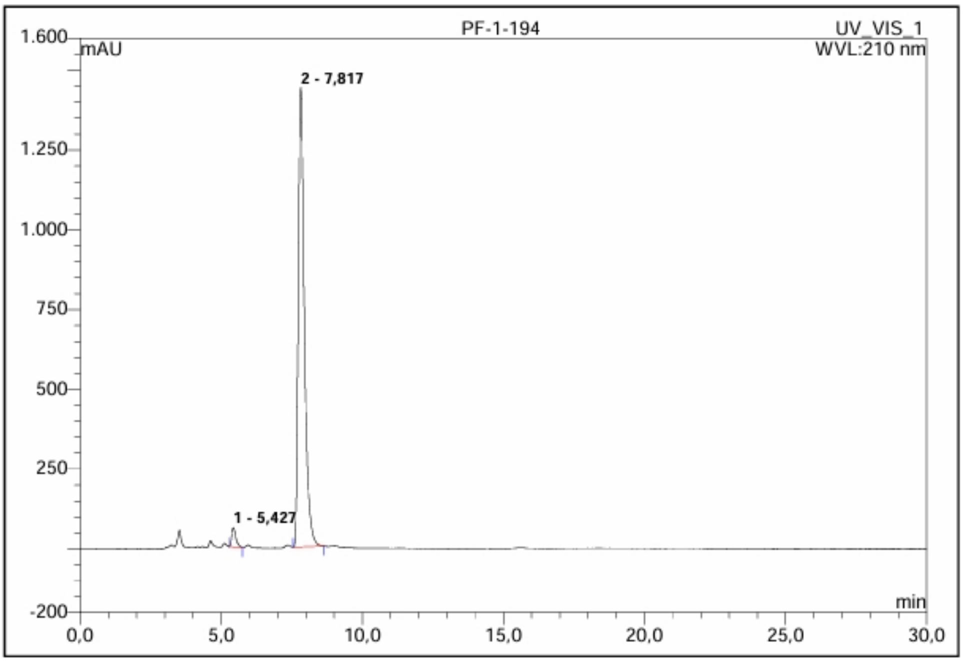

No.	Ret.Time min	Peak Name	Height mAU	Area mAU*min	Rel.Area %	Amount	Type
1	5,43	n.a.	60,405	10,407	2,79	n.a.	BMB
2	7,82	n.a.	1440,933	362,071	97,21	n.a.	BMB
Total:			1501,338	372,478	100,00	0,000	

(*R*)-7-(4-Bromobenzyl)-6,7-dihydro-5*H*-pyrrolo[3,4-*b*]pyrazin-5-one (2d)

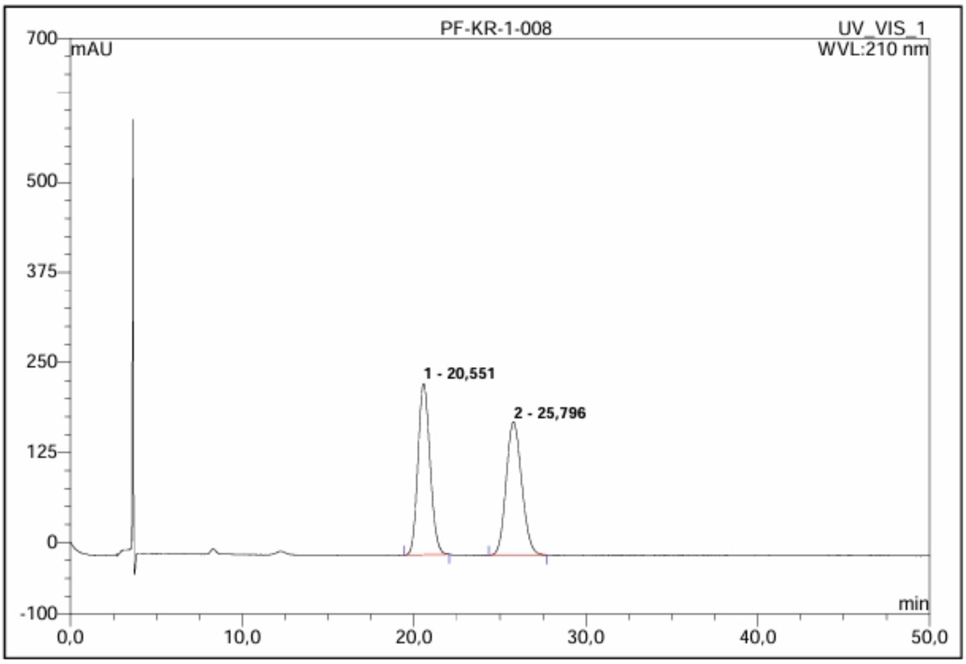

No.	Ret.Time min	Peak Name	Height mAU	Area mAU*min	Rel.Area %	Amount	Type
1	20,55	n.a.	237,676	193,561	49,88	n.a.	BMB*
2	25,80	n.a.	185,163	194,489	50,12	n.a.	BMB*
Total:			422,839	388,050	100,00	0,000	

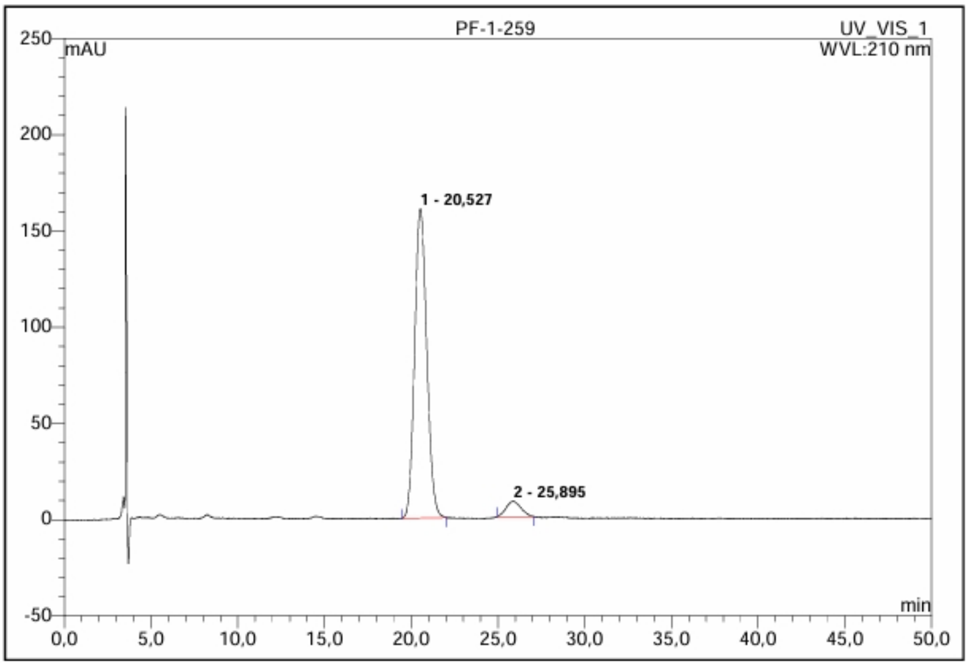

No.	Ret.Time min	Peak Name	Height mAU	Area mAU*min	Rel.Area %	Amount	Type
1	20,53	n.a.	160,710	129,883	94,31	n.a.	BMB*
2	25,90	n.a.	8,001	7,837	5,69	n.a.	BMB*
Total:			168,712	137,720	100,00	0,000	

Trace of compound 2d for SC-XRD structure report

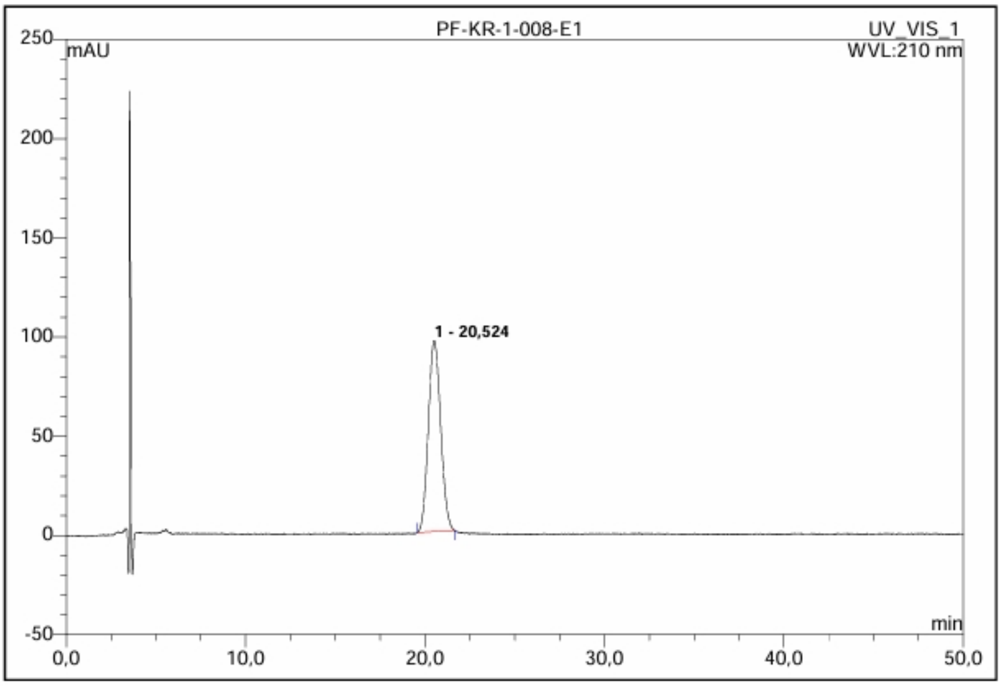

No.	Ret.Time min	Peak Name	Height mAU	Area mAU*min	Rel.Area %	Amount	Type
1	20,52	n.a.	96,206	76,694	100,00	n.a.	BMB
Total:			96,206	76,694	100,00	0,000	

(*R*)- 7-(2-Chlorobenzyl)-6,7-dihydro-5*H*-pyrrolo[3,4-*b*]pyrazin-5-one (2e)

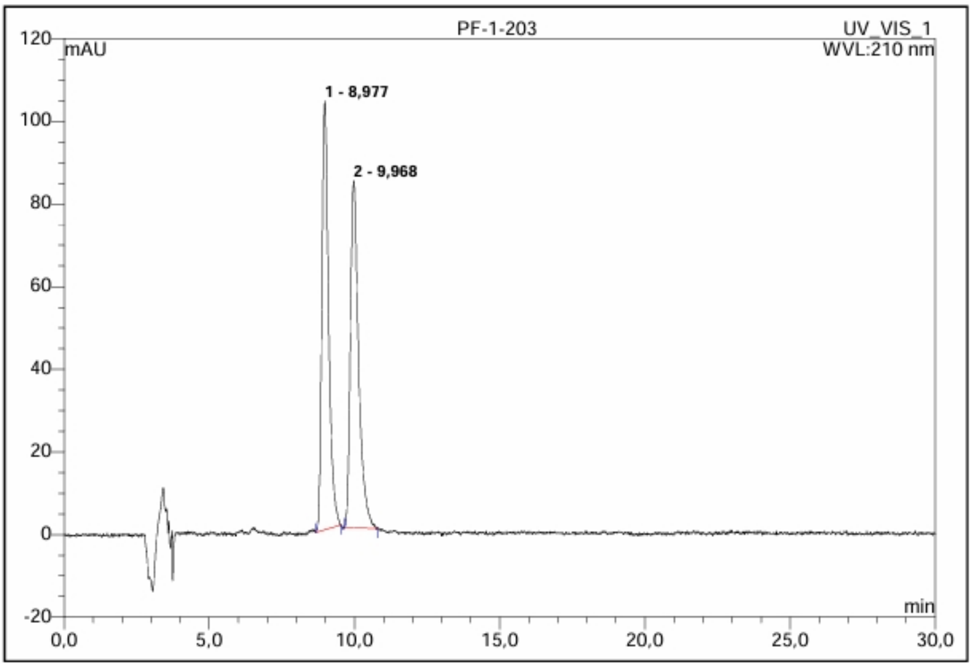

No.	Ret.Time min	Peak Name	Height mAU	Area mAU*min	Rel.Area %	Amount	Type
1	8,98	n.a.	103,733	27,769	50,19	n.a.	BMB*
2	9,97	n.a.	83,897	27,560	49,81	n.a.	BMB*
Total:			187,631	55,329	100,00	0,000	

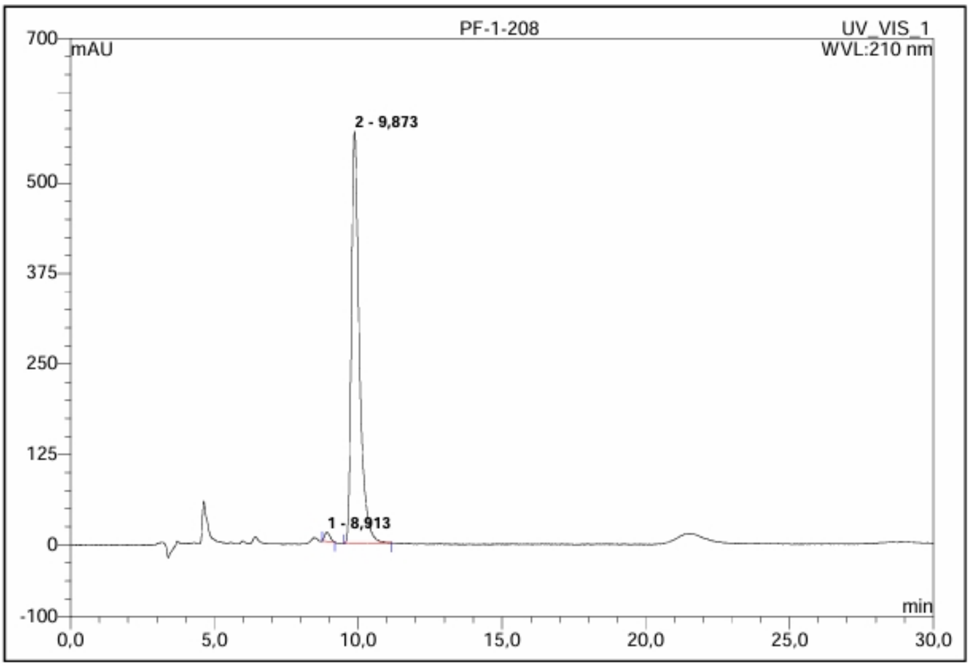

No.	Ret.Time min	Peak Name	Height mAU	Area mAU*min	Rel.Area %	Amount	Type
1	8,91	n.a.	12,631	2,694	1,45	n.a.	BMB*
2	9,87	n.a.	569,687	183,403	98,55	n.a.	BMB*
Total:			582,318	186,097	100,00	0,000	

(*R*)-7-(4-(Trifluoromethoxy)benzyl)-6,7-dihydro-5*H*-pyrrolo[3,4-*b*]pyrazin-5-one (2f)

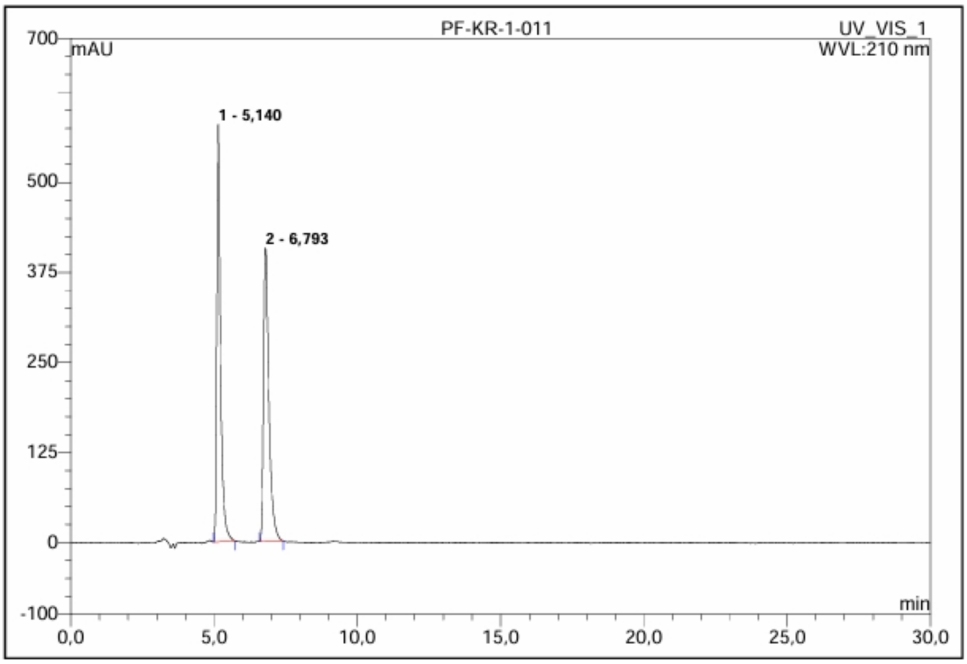

No.	Ret.Time min	Peak Name	Height mAU	Area mAU*min	Rel.Area %	Amount	Type
1	5,14	n.a.	579,430	89,267	50,11	n.a.	BMB
2	6,79	n.a.	407,856	88,874	49,89	n.a.	BMB
Total:			987,287	178,140	100,00	0,000	

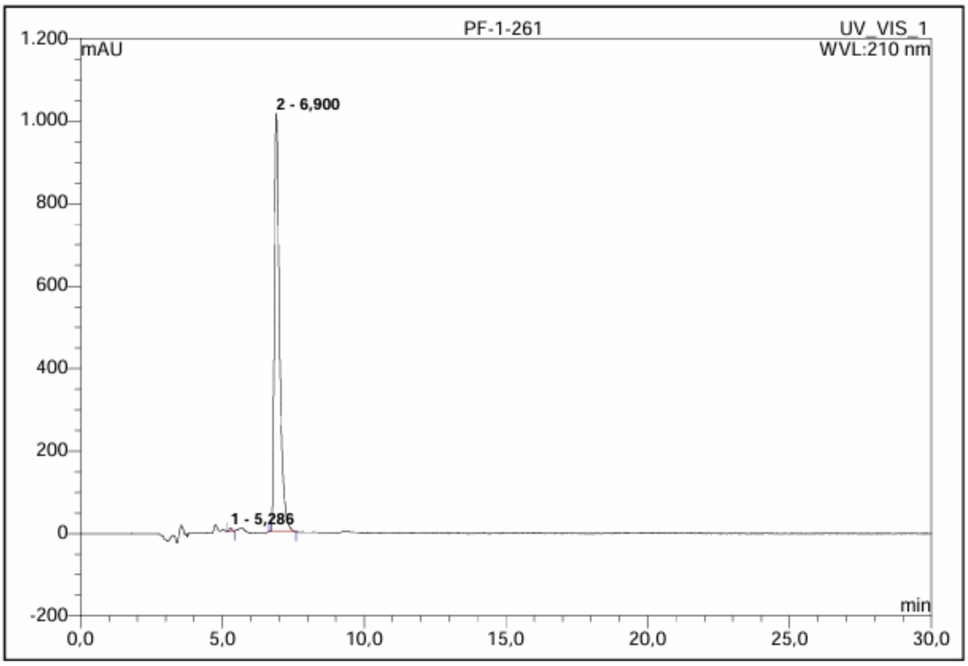

No.	Ret.Time min	Peak Name	Height mAU	Area mAU*min	Rel.Area %	Amount	Type
1	5,29	n.a.	8,221	0,884	0,41	n.a.	BMB*
2	6,90	n.a.	1014,384	216,929	99,59	n.a.	BMB*
Total:			1022,605	217,813	100,00	0,000	

(*R*)-7-(3,5-Dimethylbenzyl)-6,7-dihydro-5*H*-pyrrolo[3,4-*b*]pyrazin-5-one (2g)

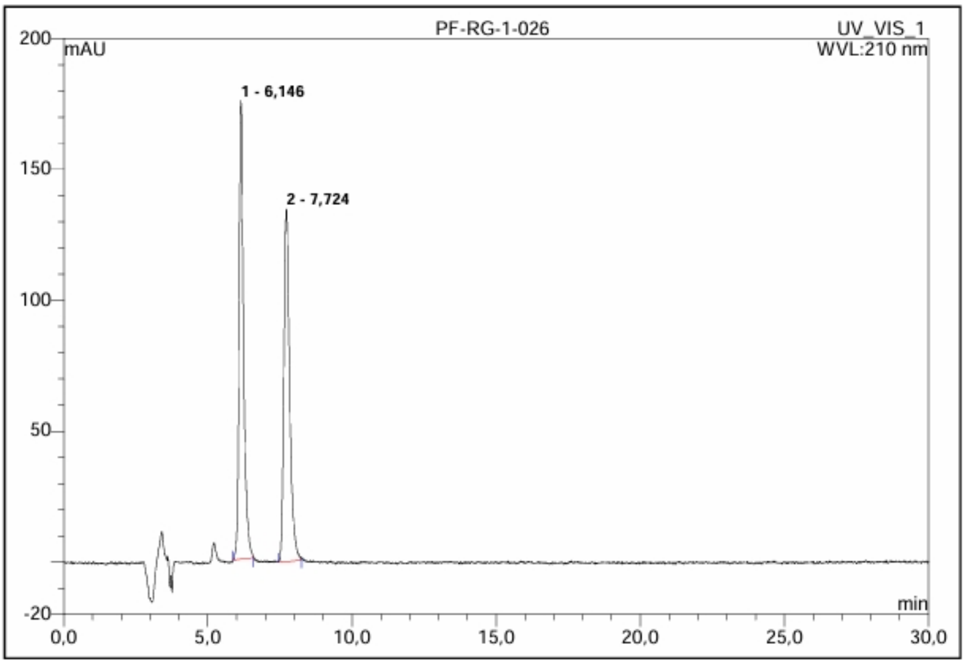

No.	Ret.Time min	Peak Name	Height mAU	Area mAU*min	Rel.Area %	Amount	Type
1	6,15	n.a.	175,132	31,850	50,45	n.a.	BMB*
2	7,72	n.a.	134,581	31,278	49,55	n.a.	BM *
Total:			309,713	63,128	100,00	0,000	

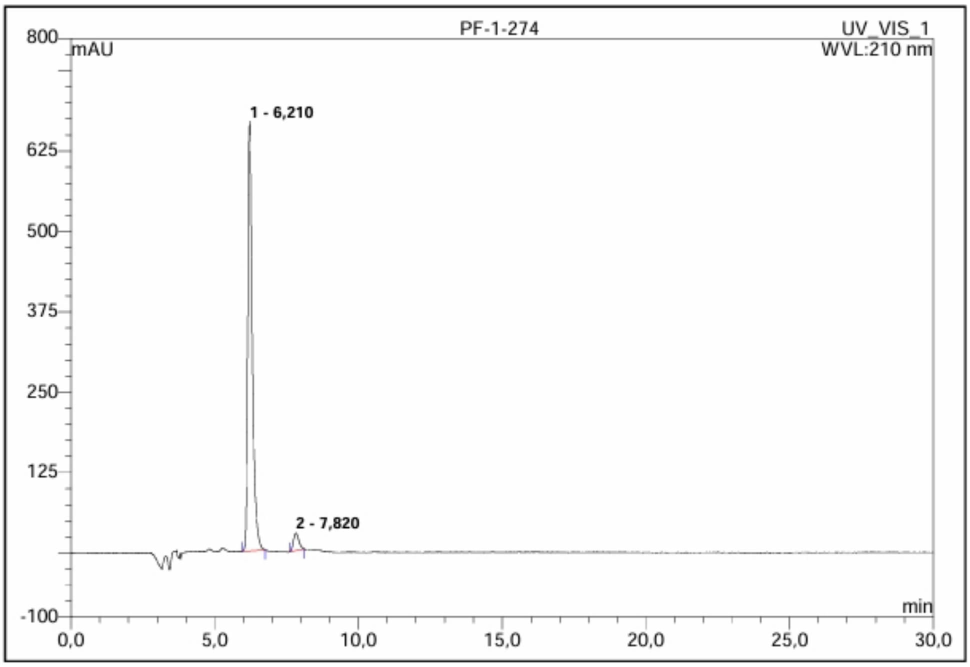

No.	Ret.Time min	Peak Name	Height mAU	Area mAU*min	Rel.Area %	Amount	Type
1	6,21	n.a.	668,253	120,616	95,33	n.a.	BMB*
2	7,82	n.a.	27,415	5,911	4,67	n.a.	BMB*
Total:			695,667	126,526	100,00	0,000	

***tert*-Butyl (*R*)-4-((7-oxo-6,7-dihydro-5*H*-pyrrolo[3,4-*b*]pyrazin-5-yl)methyl)benzoate (2h)**

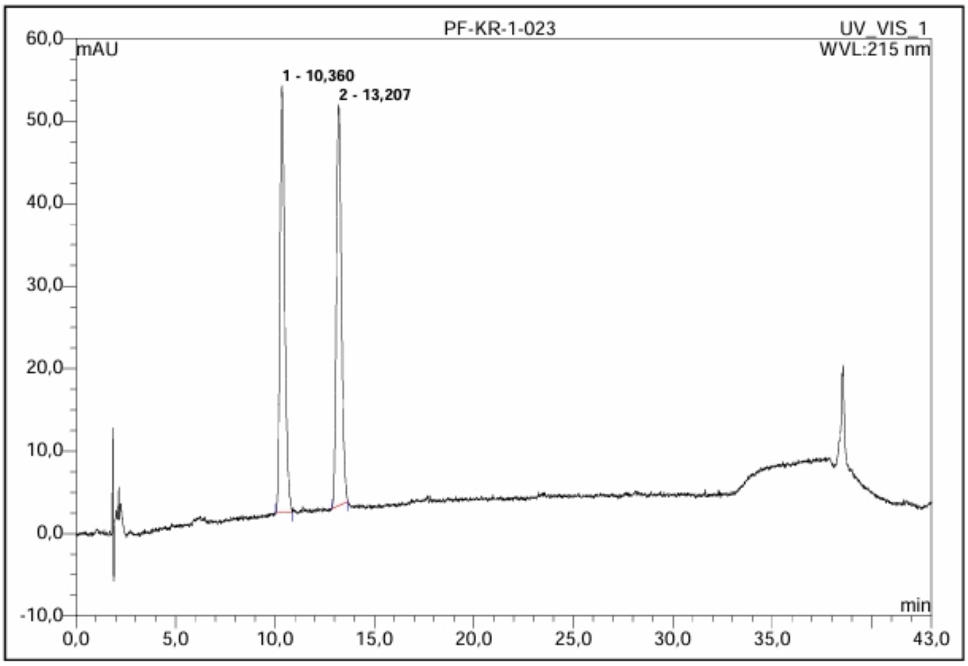

No.	Ret.Time min	Peak Name	Height mAU	Area mAU*min	Rel.Area %	Amount	Type
1	10,36	n.a.	51,735	14,506	50,54	n.a.	BMB
2	13,21	n.a.	48,617	14,196	49,46	n.a.	BMB
Total:			100,352	28,702	100,00	0,000	

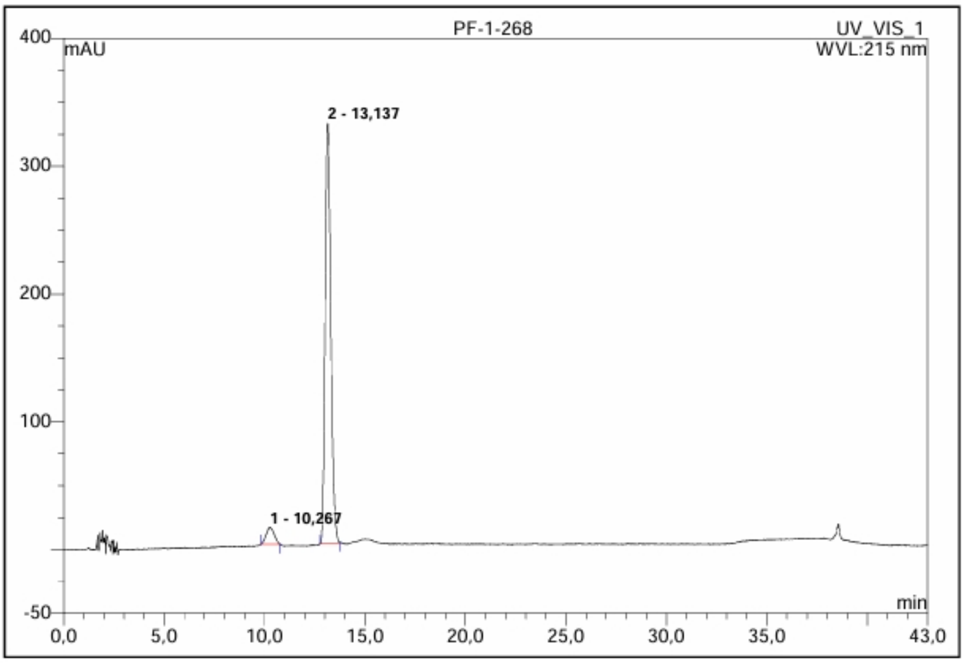

No.	Ret.Time min	Peak Name	Height mAU	Area mAU*min	Rel.Area %	Amount	Type
1	10,27	n.a.	13,202	5,979	5,23	n.a.	BMB*
2	13,14	n.a.	329,167	108,279	94,77	n.a.	BM *
Total:			342,368	114,257	100,00	0,000	

(*R*)-7-((5-(Trifluoromethyl)furan-2-yl)methyl)-6,7-dihydro-5*H*-pyrrolo[3,4-*b*]pyrazin-5-one (2i)

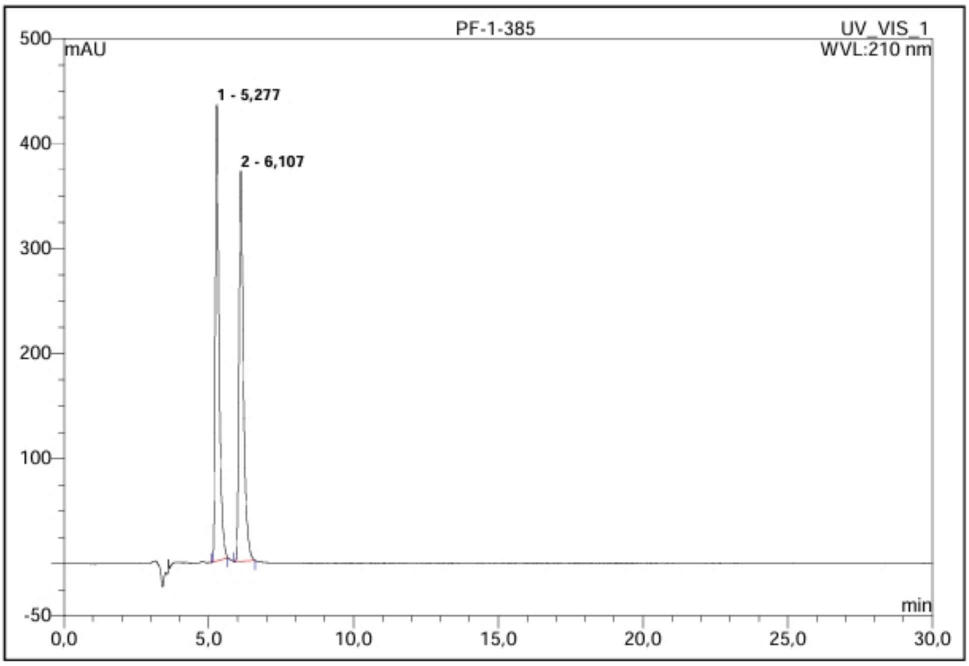

No.	Ret.Time min	Peak Name	Height mAU	Area mAU*min	Rel.Area %	Amount	Type
1	5,28	n.a.	434,646	65,521	49,75	n.a.	BMB*
2	6,11	n.a.	371,904	66,167	50,25	n.a.	BMB*
Total:			806,550	131,688	100,00	0,000	

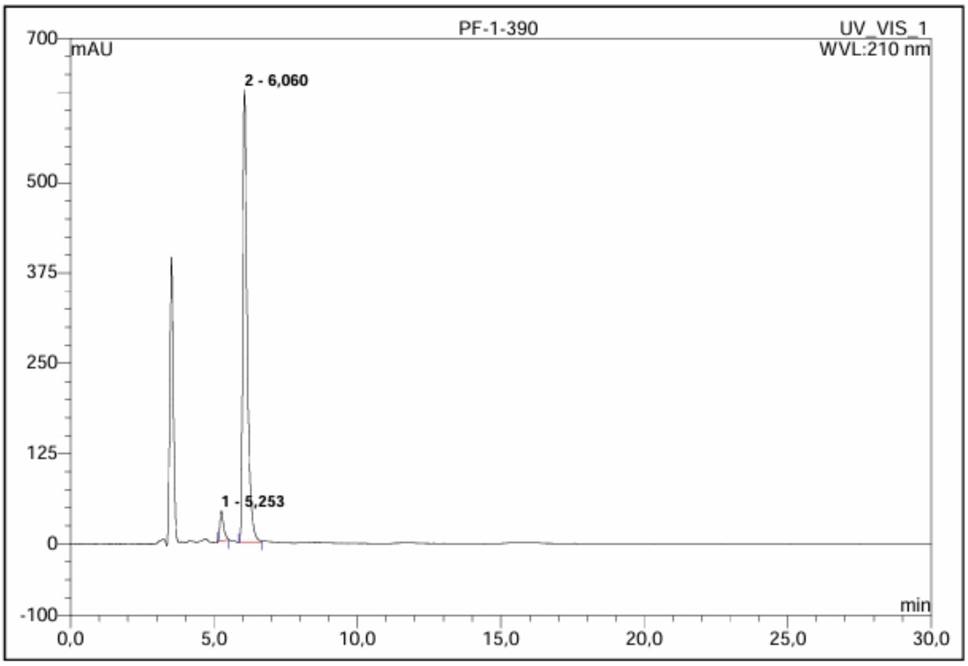

No.	Ret.Time min	Peak Name	Height mAU	Area mAU*min	Rel.Area %	Amount	Type
1	5,25	n.a.	41,678	6,187	5,20	n.a.	BMB*
2	6,06	n.a.	626,649	112,897	94,80	n.a.	BM *
Total:			668,327	119,083	100,00	0,000	

(*R*)-7-Ethyl-6,7-dihydro-5*H*-pyrrolo[3,4-*b*]pyrazin-5-one (2j)

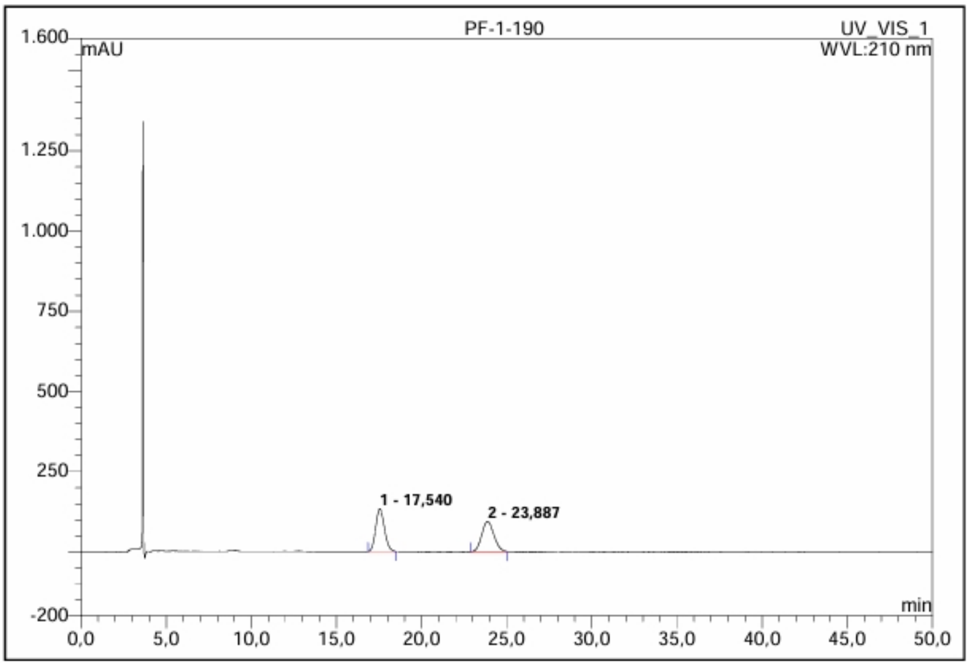

No.	Ret.Time min	Peak Name	Height mAU	Area mAU*min	Rel.Area %	Amount	Type
1	17,54	n.a.	133,042	78,967	50,34	n.a.	BMB*
2	23,89	n.a.	93,919	77,898	49,66	n.a.	BMB*
Total:			226,961	156,864	100,00	0,000	

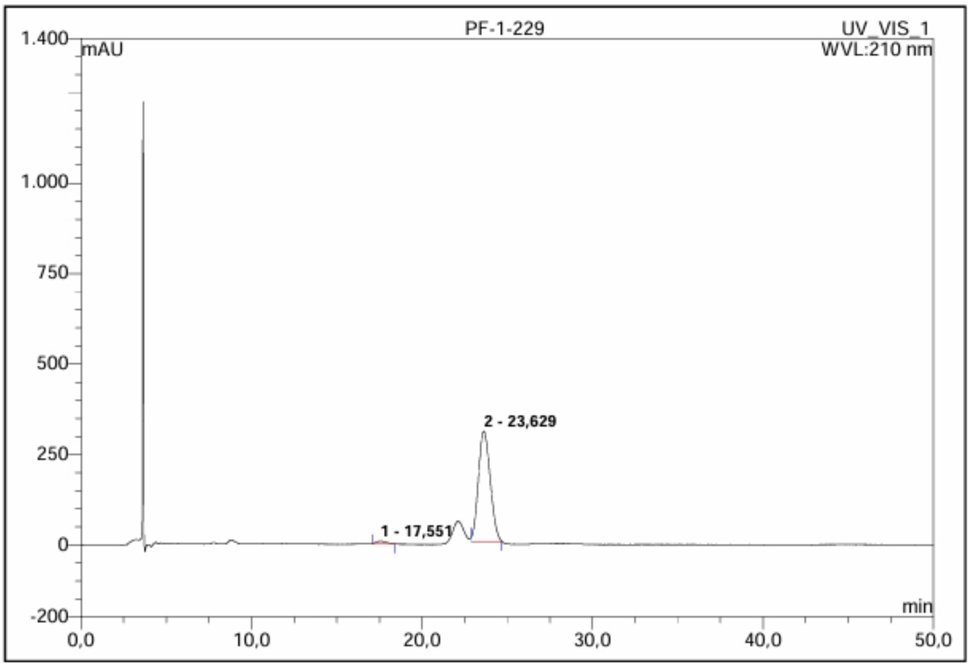

No.	Ret.Time min	Peak Name	Height mAU	Area mAU*min	Rel.Area %	Amount	Type
1	17,55	n.a.	7,608	5,285	2,10	n.a.	BMB*
2	23,63	n.a.	305,441	245,973	97,90	n.a.	MB*
Total:			313,049	251,259	100,00	0,000	

(*R*)-7-Isopentyl-6,7-dihydro-5*H*-pyrrolo[3,4-*b*]pyrazin-5-one (2k)

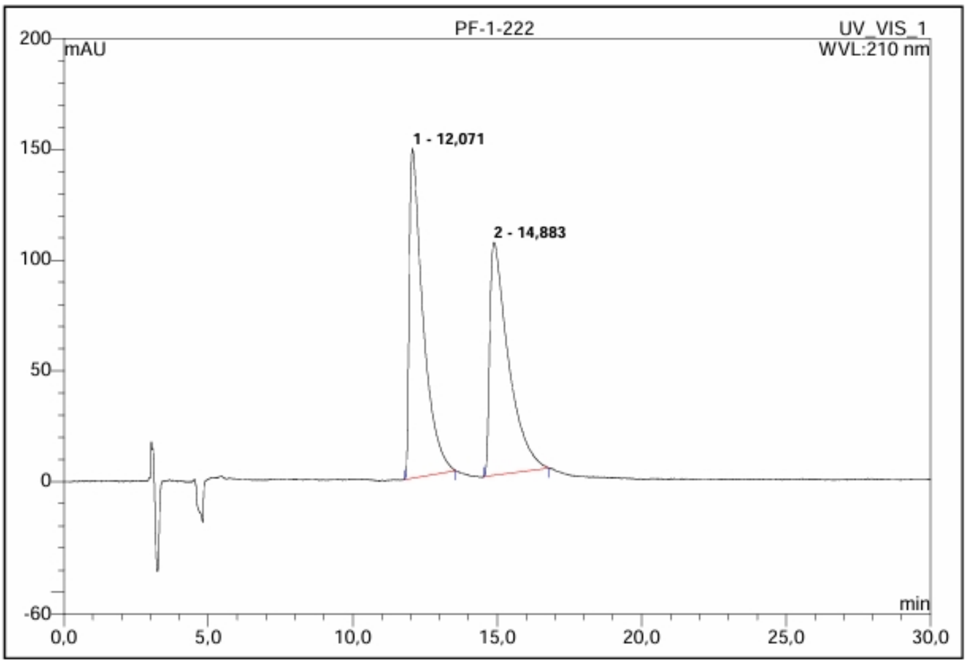

No.	Ret.Time min	Peak Name	Height mAU	Area mAU*min	Rel.Area %	Amount	Type
1	12,07	n.a.	148,756	82,334	51,06	n.a.	BMB*
2	14,88	n.a.	105,086	78,908	48,94	n.a.	BMB
Total:			253,842	161,242	100,00	0,000	

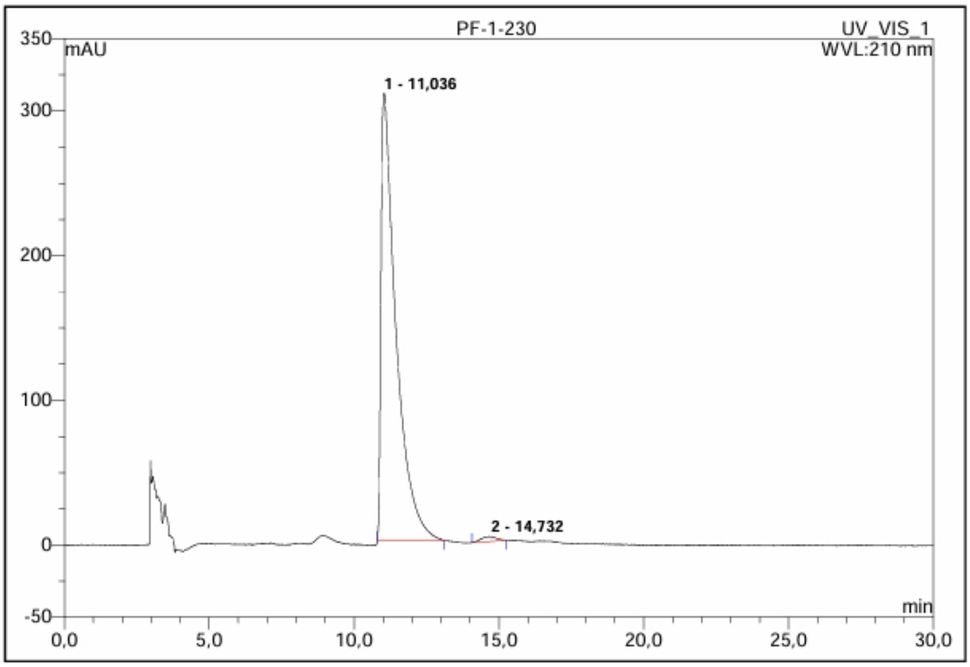

No.	Ret.Time min	Peak Name	Height mAU	Area mAU*min	Rel.Area %	Amount	Type
1	11,04	n.a.	309,201	184,612	98,89	n.a.	BMB*
2	14,73	n.a.	3,212	2,071	1,11	n.a.	BMB*
Total:			312,413	186,682	100,00	0,000	

(*R*)-7-(Cyclopentylmethyl)-6,7-dihydro-5*H*-pyrrolo[3,4-*b*]pyrazin-5-one (2l)

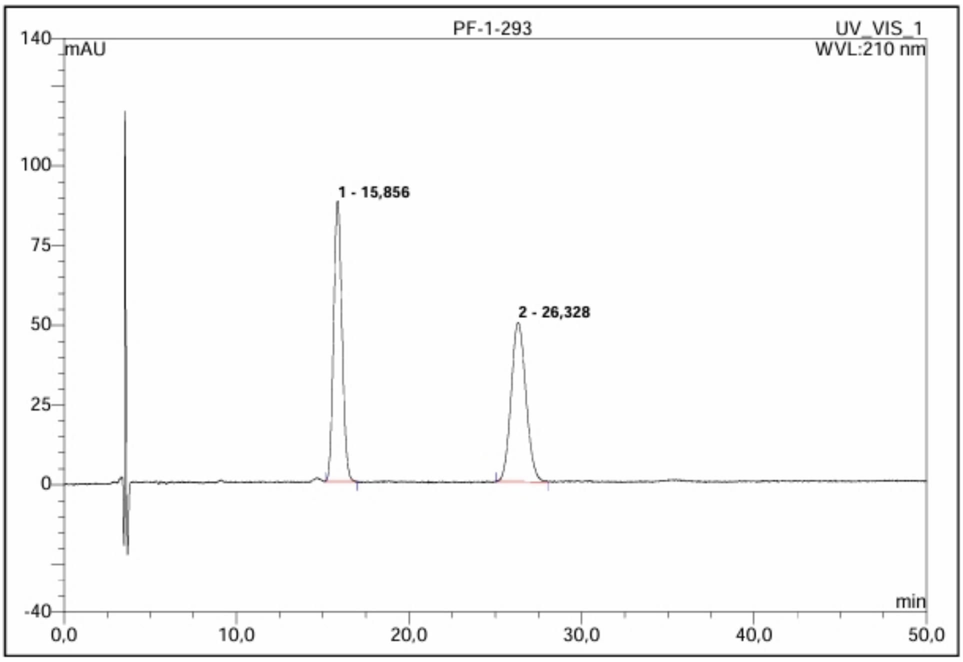

No.	Ret.Time min	Peak Name	Height mAU	Area mAU*min	Rel.Area %	Amount	Type
1	15,86	n.a.	87,968	49,621	49,87	n.a.	BMB*
2	26,33	n.a.	50,003	49,870	50,13	n.a.	BMB*
Total:			137,971	99,490	100,00	0,000	

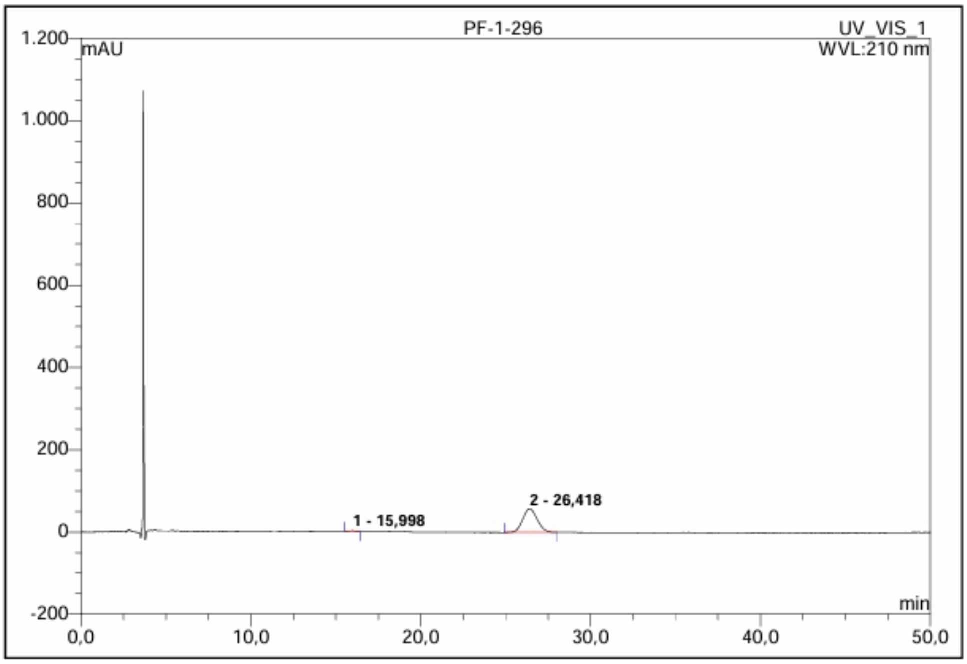

No.	Ret.Time min	Peak Name	Height mAU	Area mAU*min	Rel.Area %	Amount	Type
1	16,00	n.a.	1,440	0,520	0,91	n.a.	BM *
2	26,42	n.a.	57,059	56,386	99,09	n.a.	BMB*
Total:			58,500	56,907	100,00	0,000	

(*R*)-7-(Cyclopropylmethyl)-6,7-dihydro-5*H*-pyrrolo[3,4-*b*]pyrazin-5-one (2m)

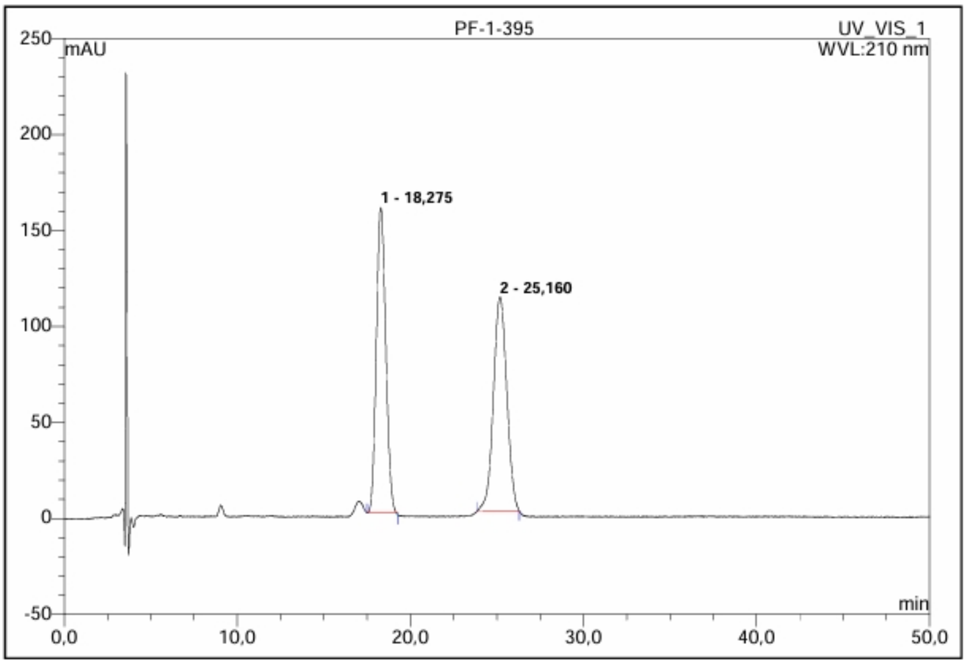

No.	Ret.Time min	Peak Name	Height mAU	Area mAU*min	Rel.Area %	Amount	Type
1	18,27	n.a.	158,933	99,877	49,94	n.a.	BM *
2	25,16	n.a.	111,586	100,107	50,06	n.a.	BMB*
Total:			270,519	199,984	100,00	0,000	

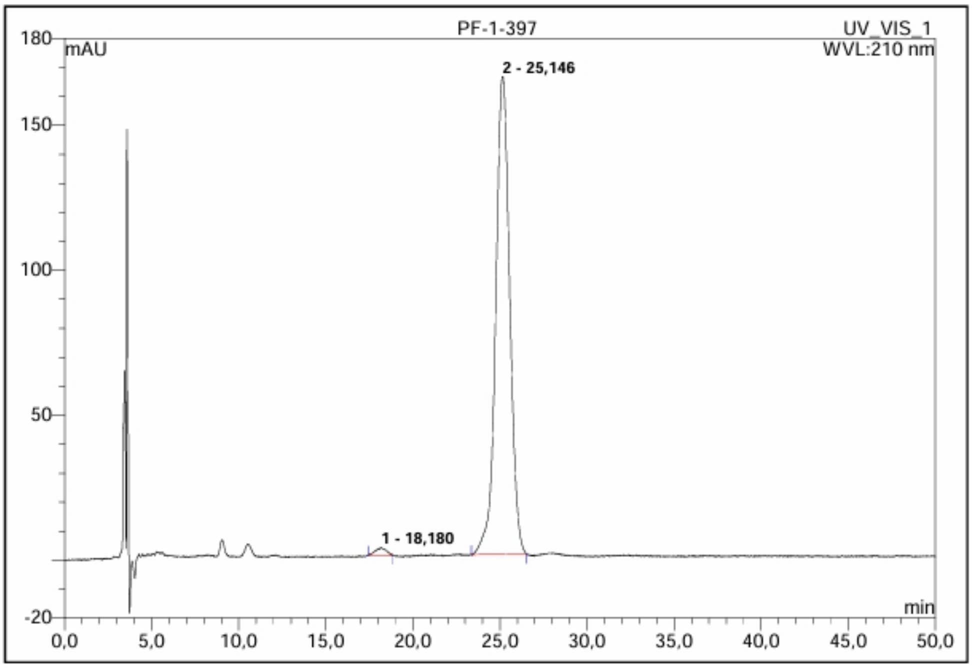

No.	Ret.Time min	Peak Name	Height mAU	Area mAU*min	Rel.Area %	Amount	Type
1	18,18	n.a.	2,666	1,867	1,20	n.a.	BM *
2	25,15	n.a.	164,812	153,846	98,80	n.a.	BMB*
Total:			167,478	155,712	100,00	0,000	

(*R*)-7-(2-Chloroethoxy)-6,7-dihydro-5*H*-pyrrolo[3,4-*b*]pyrazin-5-one (2n)

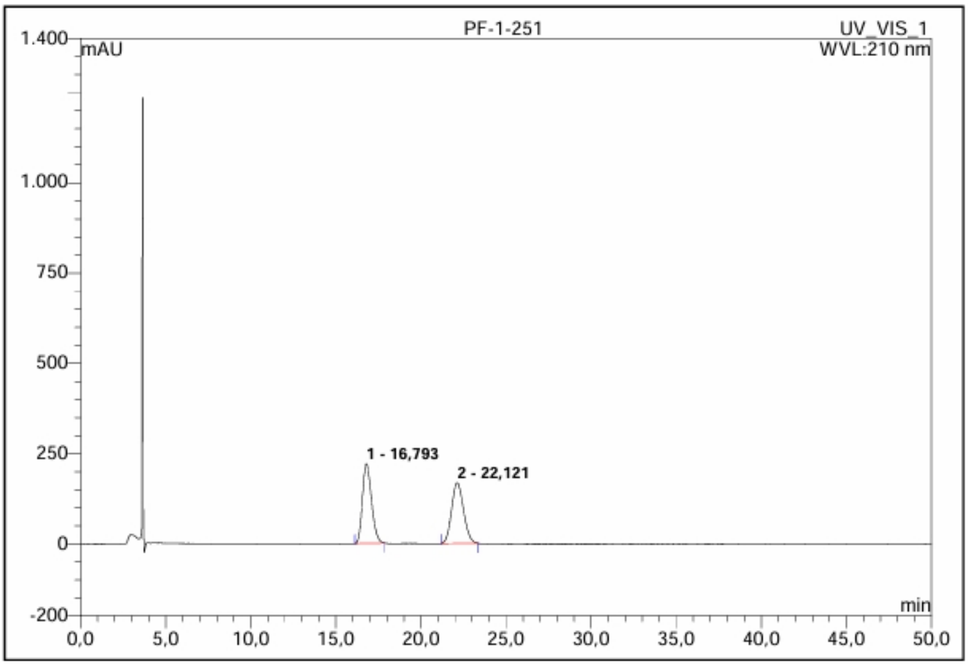

No.	Ret.Time min	Peak Name	Height mAU	Area mAU*min	Rel.Area %	Amount	Type
1	16,79	n.a.	221,048	136,932	49,97	n.a.	BMB
2	22,12	n.a.	168,377	137,080	50,03	n.a.	BMB
Total:			389,425	274,012	100,00	0,000	

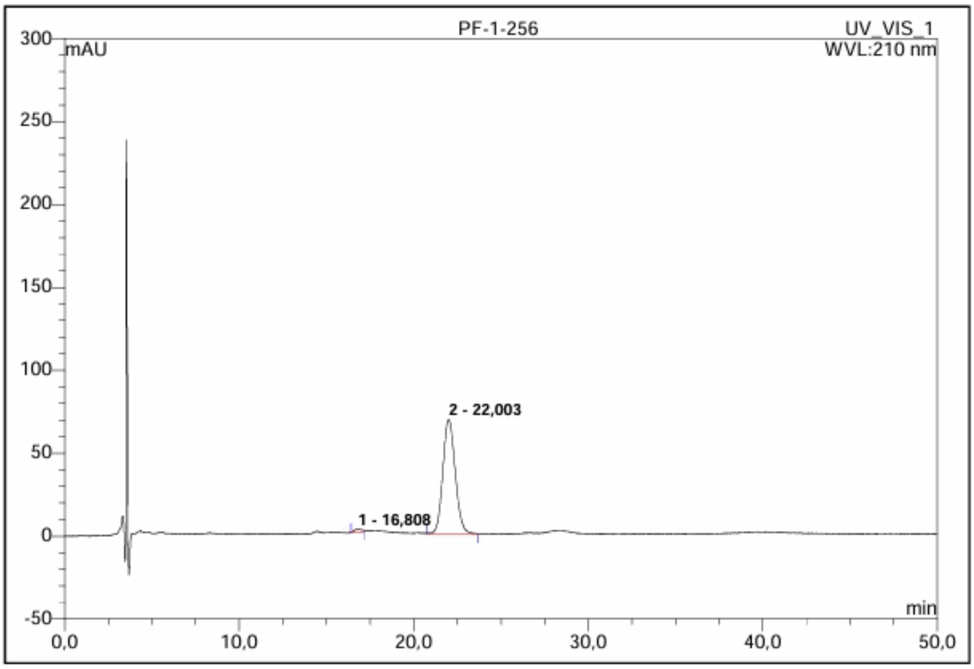

No.	Ret.Time min	Peak Name	Height mAU	Area mAU*min	Rel.Area %	Amount	Type
1	16,81	n.a.	2,216	1,146	2,00	n.a.	BM *
2	22,00	n.a.	68,790	56,079	98,00	n.a.	BMB*
Total:			71,006	57,225	100,00	0,000	

(*R*)-7-Isobutoxy-6,7-dihydro-5*H*-pyrrolo[3,4-*b*]pyrazin-5-one (2o)

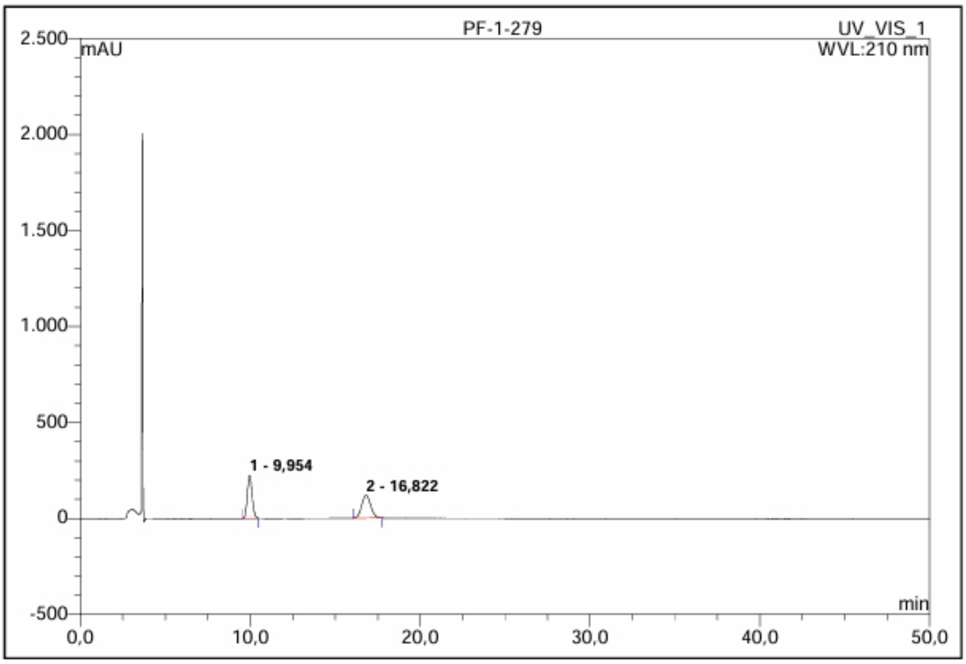

No.	Ret.Time min	Peak Name	Height mAU	Area mAU*min	Rel.Area %	Amount	Type
1	9,95	n.a.	223,916	72,544	50,03	n.a.	BMB
2	16,82	n.a.	119,134	72,446	49,97	n.a.	BMB
Total:			343,050	144,990	100,00	0,000	

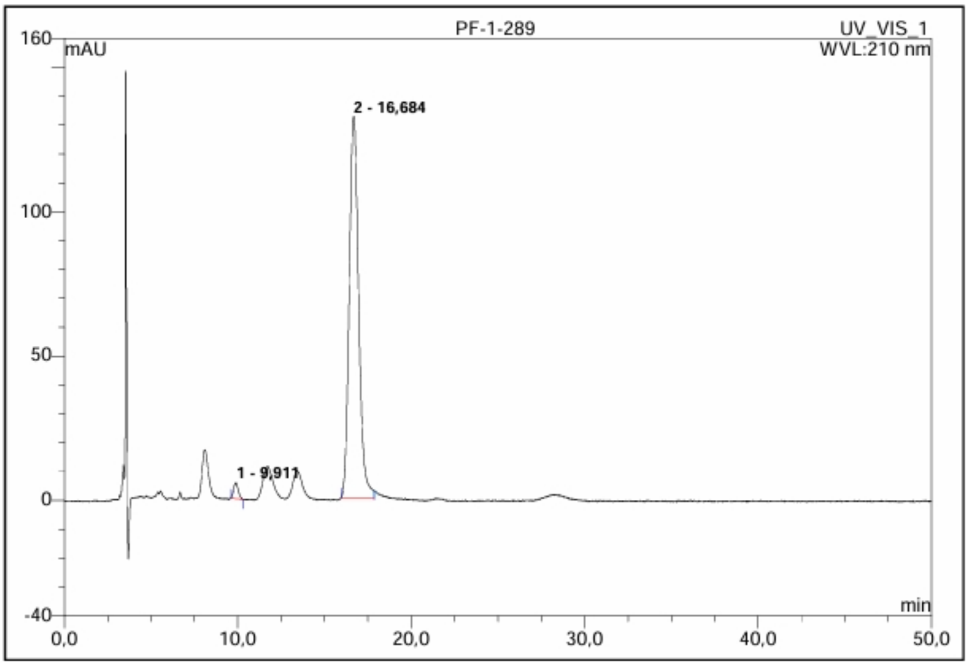

No.	Ret.Time min	Peak Name	Height mAU	Area mAU*min	Rel.Area %	Amount	Type
1	9,91	n.a.	5,554	1,709	2,03	n.a.	BMB*
2	16,68	n.a.	132,018	82,535	97,97	n.a.	BM *
Total:			137,572	84,244	100,00	0,000	

(*R*)-7-(But-3-en-1-yloxy)-6,7-dihydro-5*H*-pyrrolo[3,4-*b*]pyrazin-5-one (2p)

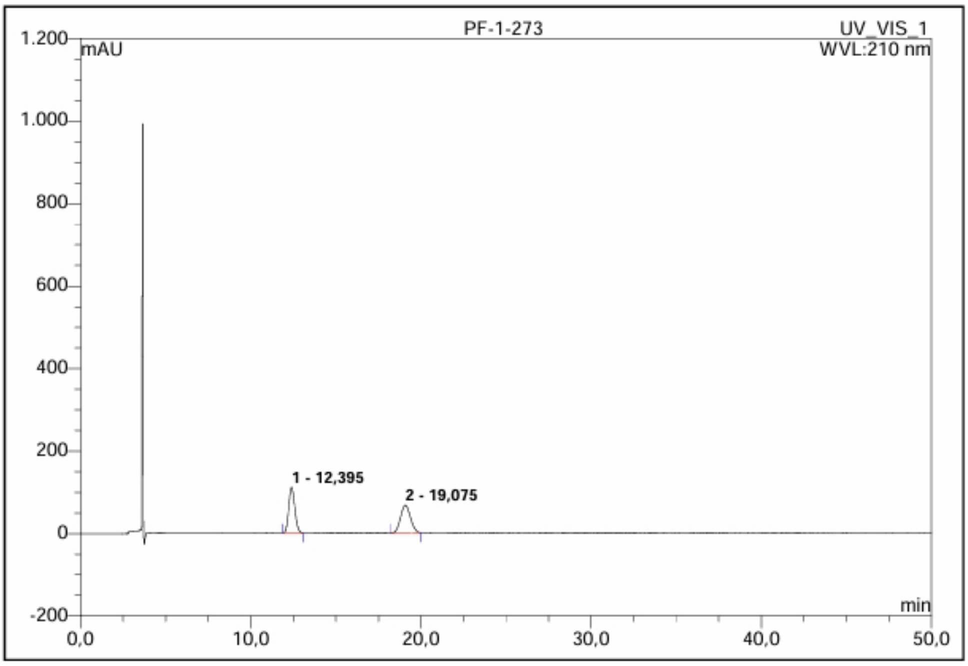

No.	Ret.Time min	Peak Name	Height mAU	Area mAU*min	Rel.Area %	Amount	Type
1	12,39	n.a.	111,147	46,006	50,25	n.a.	BMB
2	19,08	n.a.	67,371	45,542	49,75	n.a.	BMB
Total:			178,518	91,548	100,00	0,000	

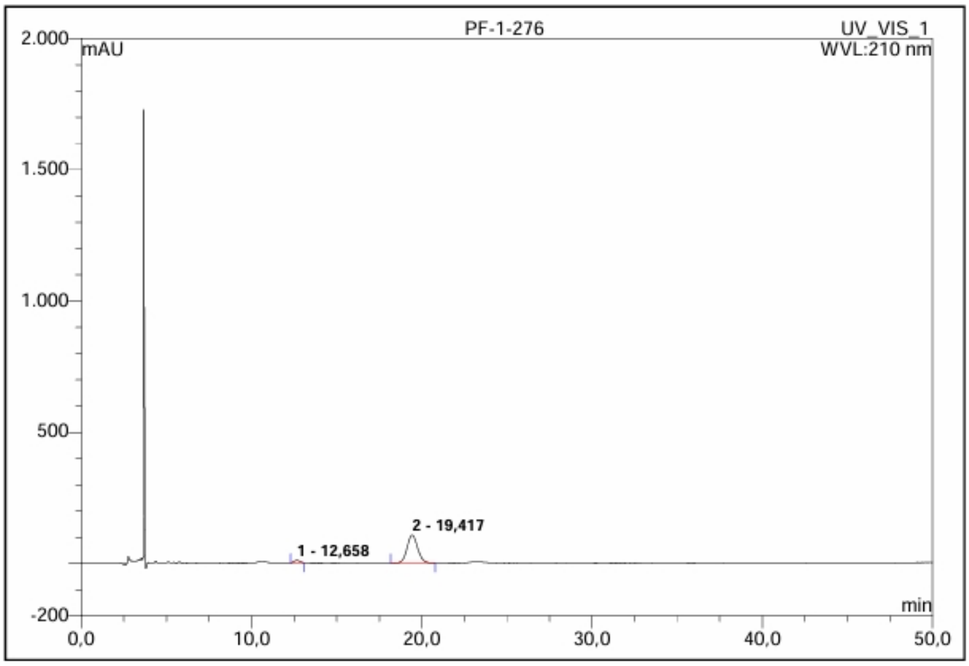

No.	Ret.Time min	Peak Name	Height mAU	Area mAU*min	Rel.Area %	Amount	Type
1	12,66	n.a.	9,490	3,637	4,50	n.a.	BMB*
2	19,42	n.a.	106,871	77,142	95,50	n.a.	BMB*
Total:			116,361	80,779	100,00	0,000	

(*R*)-7-Isopropoxy-6,7-dihydro-5*H*-pyrrolo[3,4-*b*]pyrazin-5-one (2q)

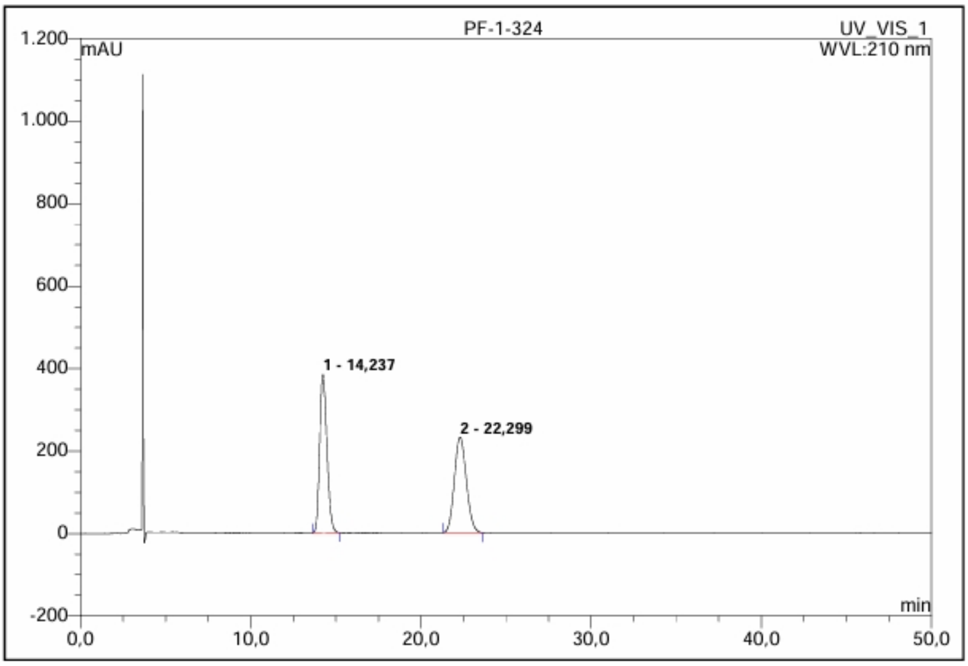

No.	Ret.Time min	Peak Name	Height mAU	Area mAU*min	Rel.Area %	Amount	Type
1	14,24	n.a.	383,578	189,535	50,14	n.a.	BMB
2	22,30	n.a.	231,282	188,480	49,86	n.a.	BMB
Total:			614,860	378,015	100,00	0,000	

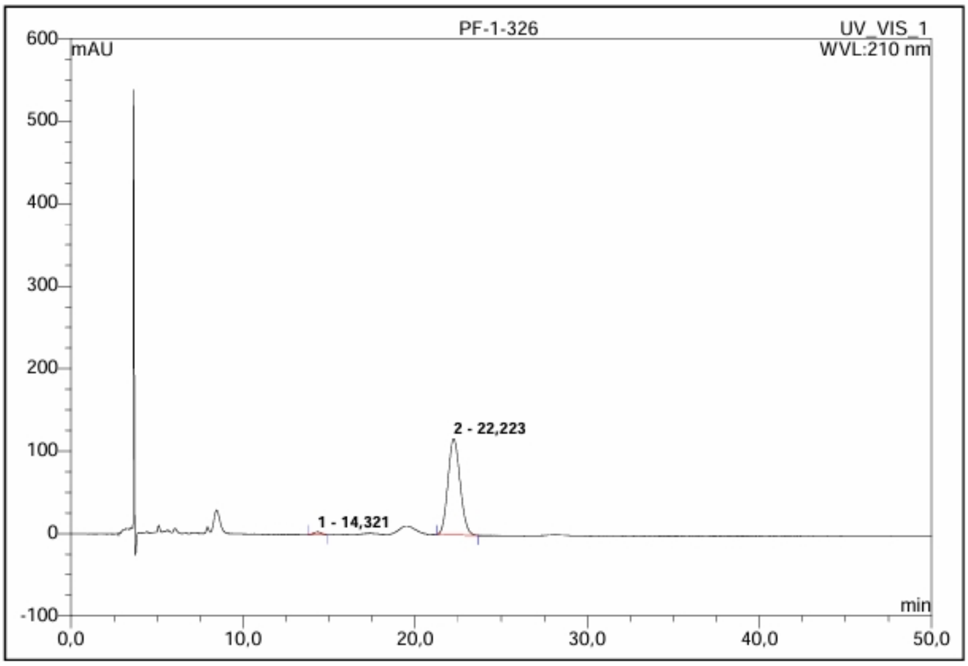

No.	Ret.Time min	Peak Name	Height mAU	Area mAU*min	Rel.Area %	Amount	Type
1	14,32	n.a.	3,066	1,449	1,50	n.a.	BMB*
2	22,22	n.a.	116,485	95,261	98,50	n.a.	BMB*
Total:			119,551	96,711	100,00	0,000	

(*R*)-7-(3-Methoxy-3-methylbutoxy)-6,7-dihydro-5*H*-pyrrolo[3,4-*b*]pyrazin-5-one (2r)

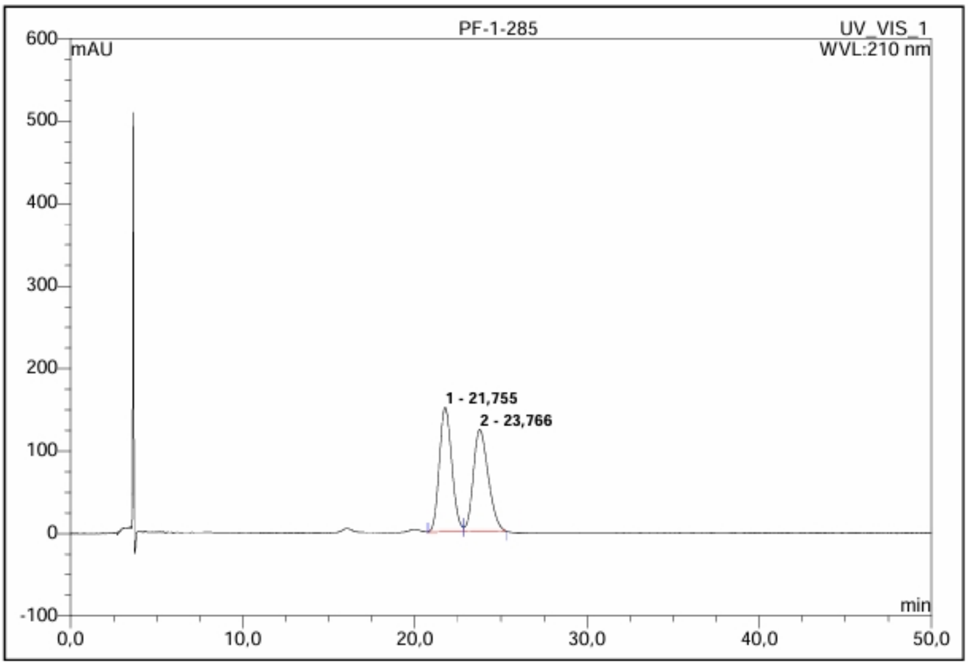

No.	Ret.Time min	Peak Name	Height mAU	Area mAU*min	Rel.Area %	Amount	Type
1	21,75	n.a.	150,663	126,160	50,37	n.a.	BM
2	23,77	n.a.	123,531	124,287	49,63	n.a.	MB
Total:			274,194	250,447	100,00	0,000	

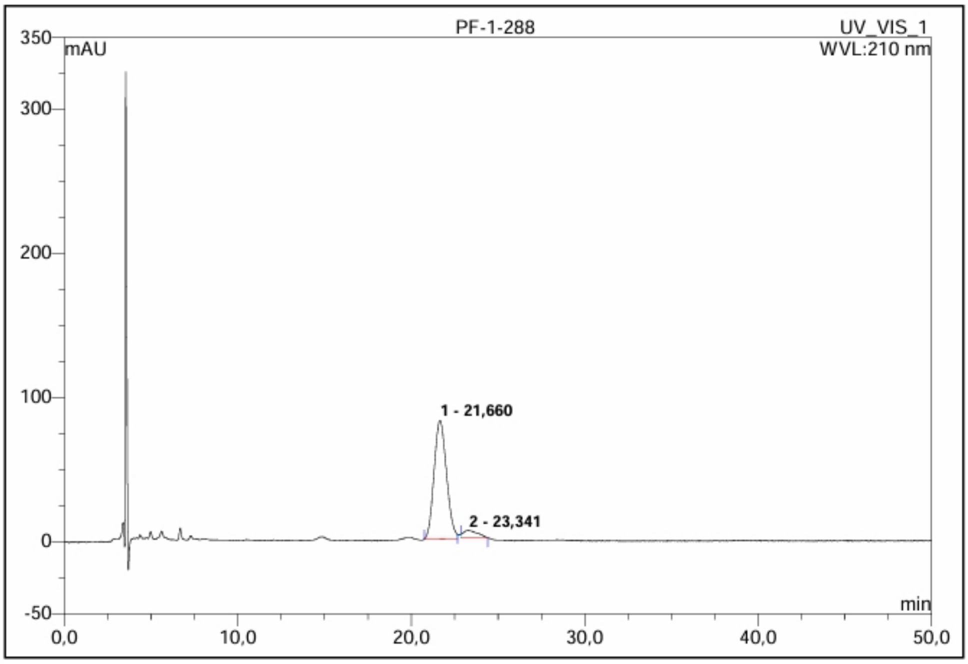

No.	Ret.Time min	Peak Name	Height mAU	Area mAU*min	Rel.Area %	Amount	Type
1	21,66	n.a.	82,113	68,057	92,87	n.a.	BM *
2	23,34	n.a.	5,282	5,226	7,13	n.a.	M *
Total:			87,394	73,283	100,00	0,000	

(*R*)-7-(2,2,2-Trifluoroethoxy)-6,7-dihydro-5*H*-pyrrolo[3,4-*b*]pyrazin-5-one (2s)

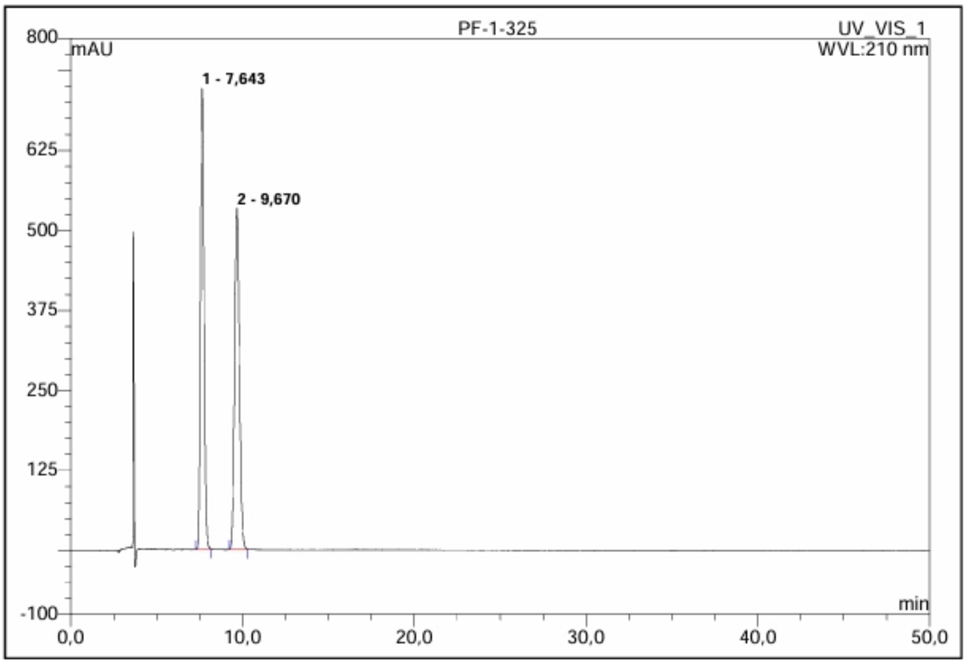

No.	Ret.Time min	Peak Name	Height mAU	Area mAU*min	Rel.Area %	Amount	Type
1	7,64	n.a.	720,004	172,304	49,91	n.a.	BMB
2	9,67	n.a.	532,692	172,944	50,09	n.a.	BMB
Total:			1252,696	345,248	100,00	0,000	

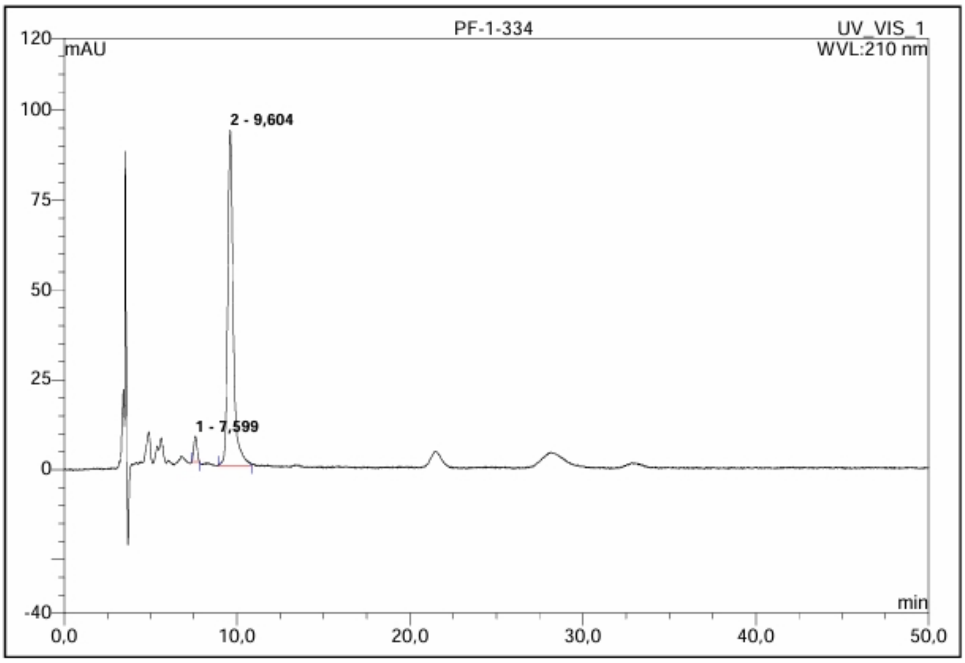

No.	Ret.Time min	Peak Name	Height mAU	Area mAU*min	Rel.Area %	Amount	Type
1	7,60	n.a.	7,085	1,539	4,23	n.a.	MB*
2	9,60	n.a.	93,317	34,818	95,77	n.a.	BM *
Total:			100,402	36,357	100,00	0,000	

(*R*)-7-((*tert*-Butyldimethylsilyl)oxy)-6,7-dihydro-5*H*-pyrrolo[3,4-*b*]pyrazin-5-one (2t)

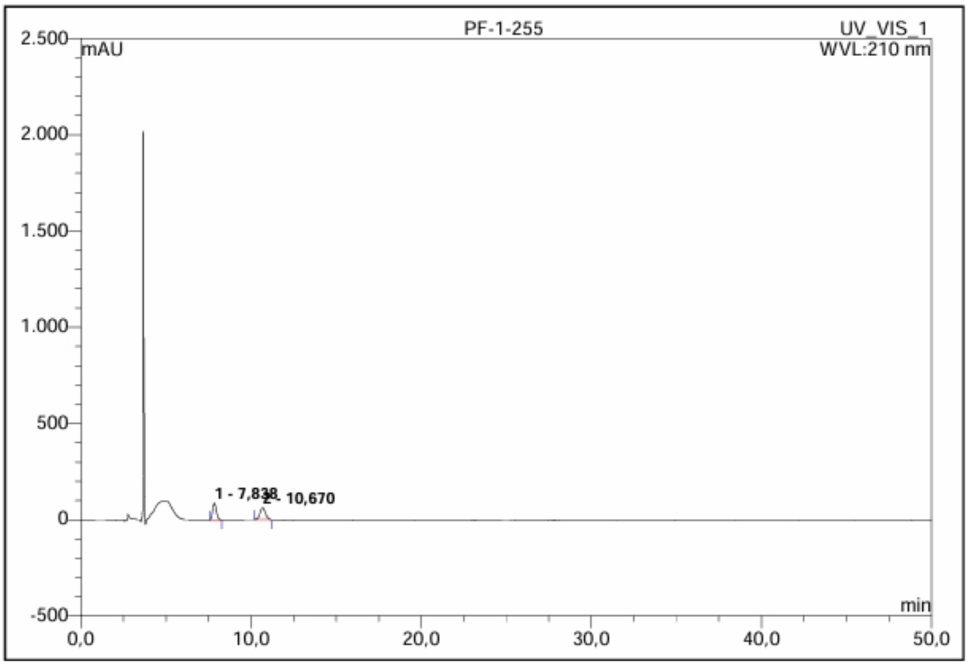

No.	Ret.Time min	Peak Name	Height mAU	Area mAU*min	Rel.Area %	Amount	Type
1	7,84	n.a.	87,885	22,266	49,54	n.a.	BMB
2	10,67	n.a.	60,984	22,682	50,46	n.a.	BMB
Total:			148,869	44,948	100,00	0,000	

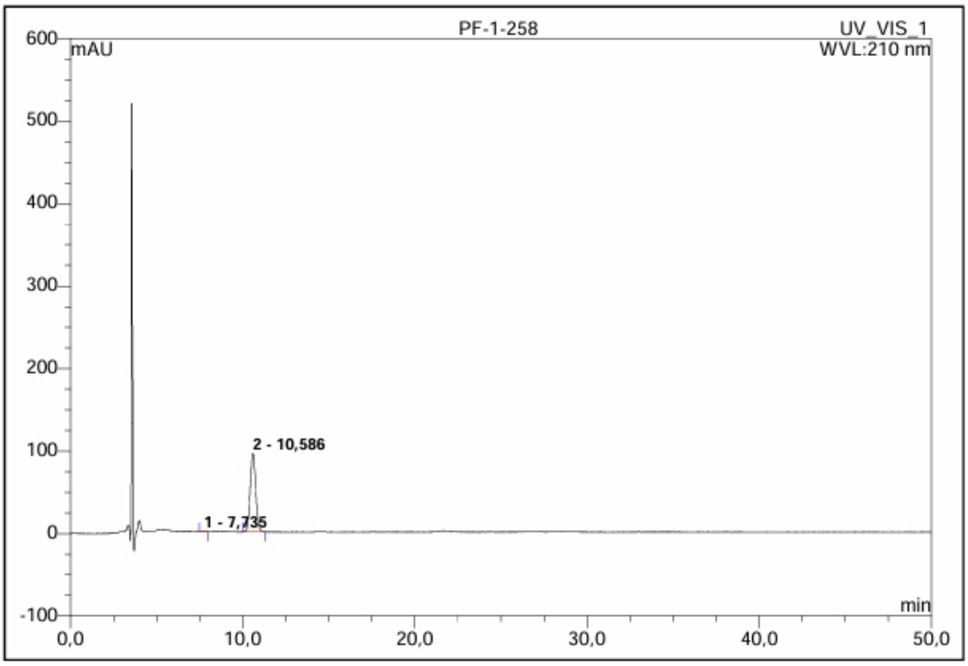

No.	Ret.Time min	Peak Name	Height mAU	Area mAU*min	Rel.Area %	Amount	Type
1	7,74	n.a.	1,113	0,245	0,68	n.a.	BMB*
2	10,59	n.a.	95,112	35,491	99,32	n.a.	BMB*
Total:			96,225	35,736	100,00	0,000	

1 mmol scale

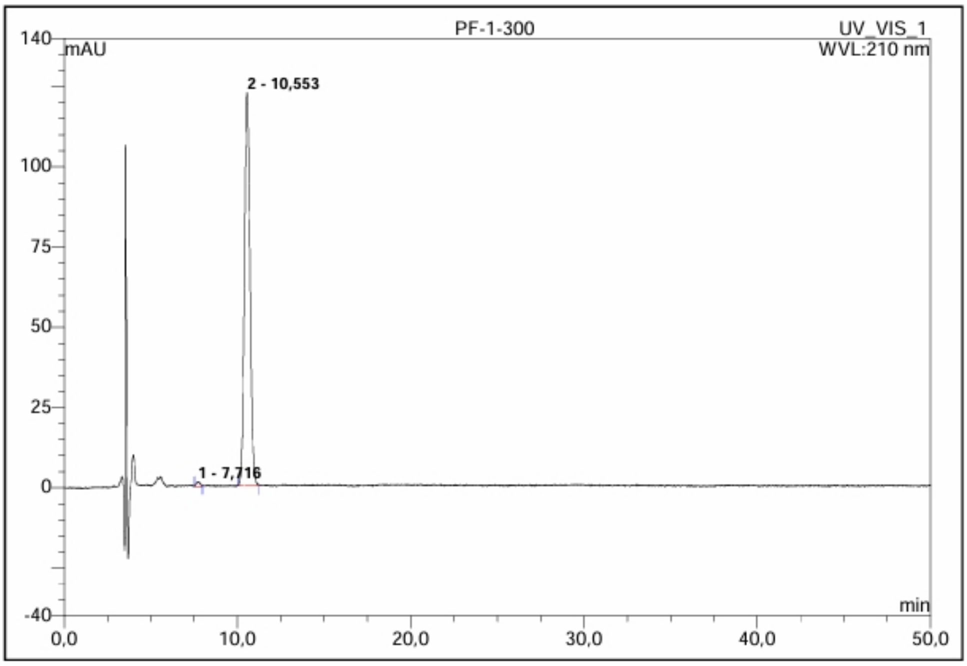

No.	Ret.Time min	Peak Name	Height mAU	Area mAU*min	Rel.Area %	Amount	Type
1	7,72	n.a.	1,434	0,355	0,77	n.a.	M *
2	10,55	n.a.	122,457	45,657	99,23	n.a.	BMB*
Total:			123,890	46,012	100,00	0,000	

(*R*)-7-((Triethylsilyl)oxy)-6,7-dihydro-5*H*-pyrrolo[3,4-*b*]pyrazin-5-one (2u)

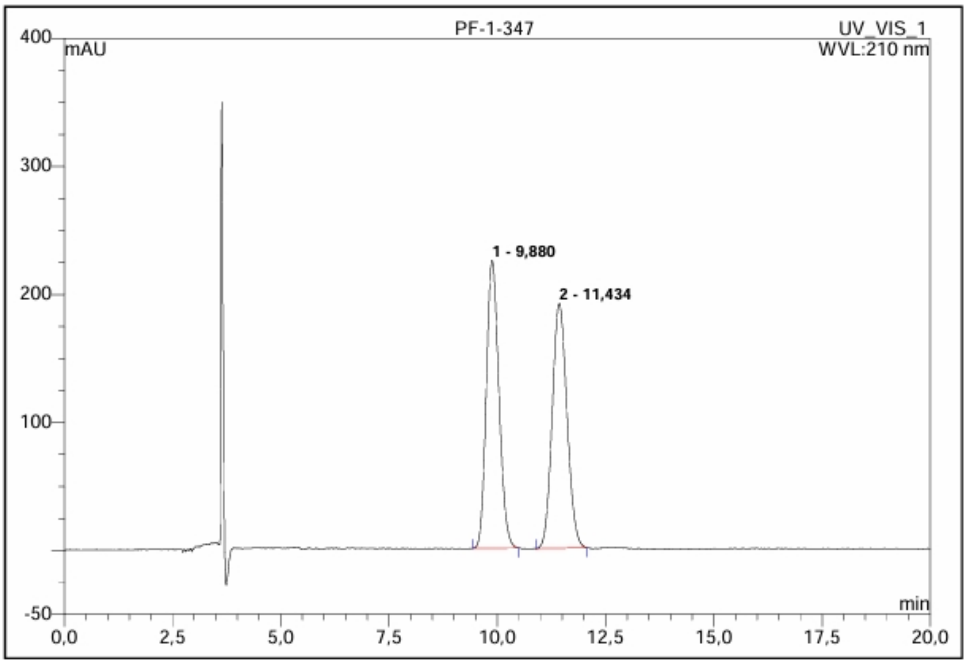

No.	Ret.Time min	Peak Name	Height mAU	Area mAU*min	Rel.Area %	Amount	Type
1	9,88	n.a.	224,810	75,923	50,05	n.a.	BMB
2	11,43	n.a.	191,128	75,758	49,95	n.a.	BMB
Total:			415,938	151,681	100,00	0,000	

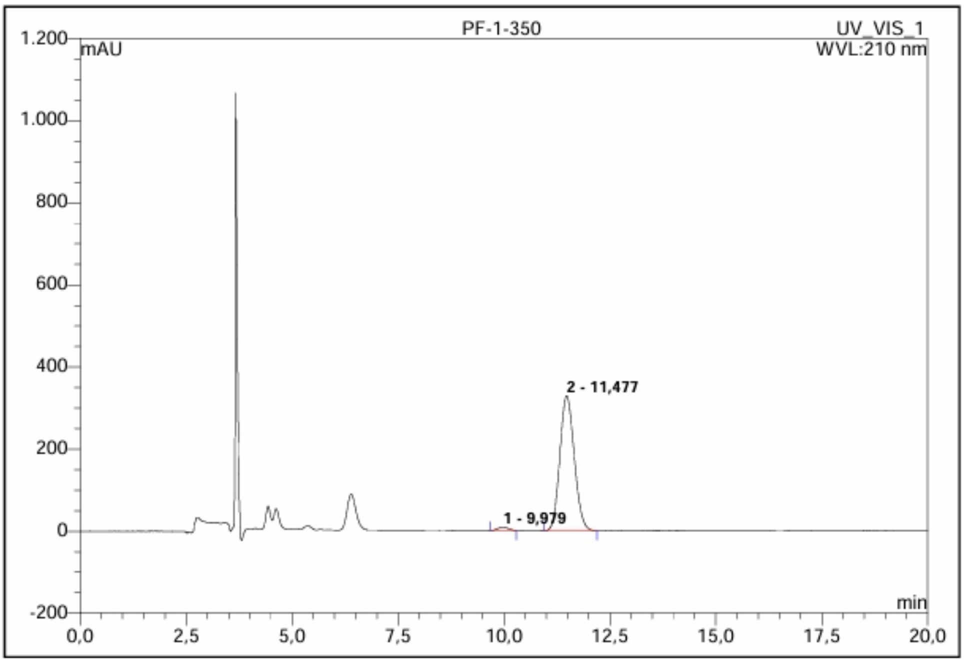

No.	Ret.Time min	Peak Name	Height mAU	Area mAU*min	Rel.Area %	Amount	Type
1	9,98	n.a.	8,288	2,501	1,88	n.a.	BMB*
2	11,48	n.a.	328,569	130,296	98,12	n.a.	BMB
Total:			336,856	132,797	100,00	0,000	

7-(4-Chlorobenzyl)-6,7-dihydro-5*H*-pyrrolo[3,4-*b*]pyrazin-5-one (SI-6)

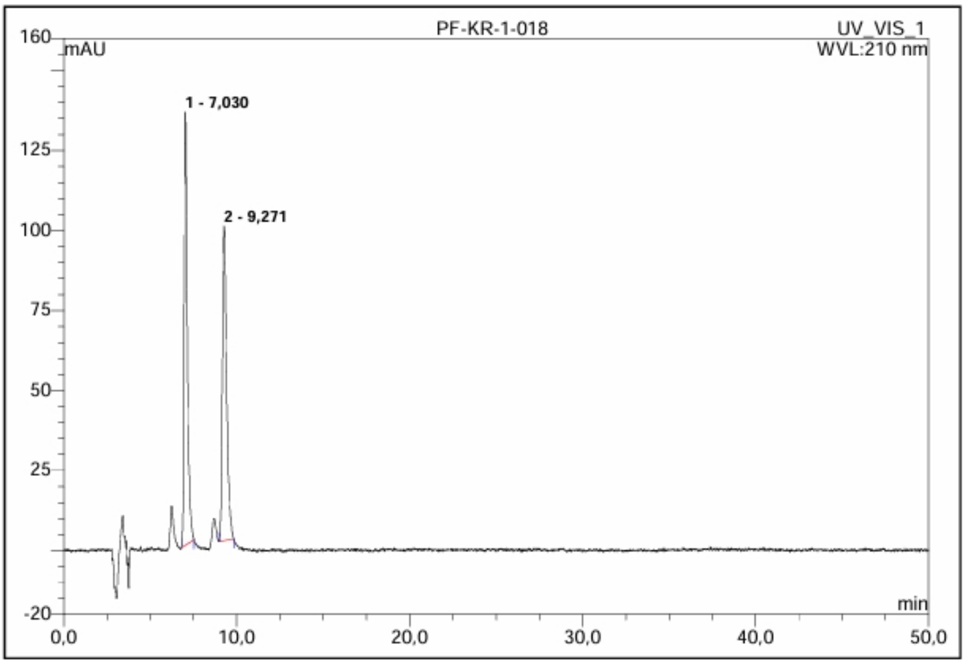

No.	Ret.Time min	Peak Name	Height mAU	Area mAU*min	Rel.Area %	Amount	Type
1	7,03	n.a.	135,363	28,743	50,78	n.a.	BMB
2	9,27	n.a.	98,249	27,861	49,22	n.a.	BMB
Total:			233,612	56,604	100,00	0,000	

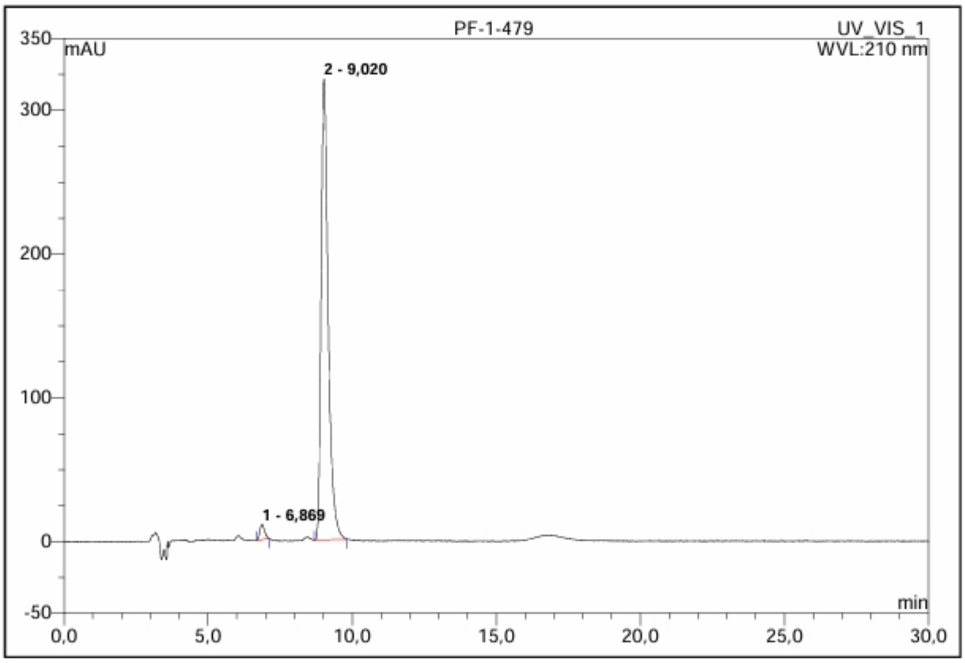

No.	Ret.Time min	Peak Name	Height mAU	Area mAU*min	Rel.Area %	Amount	Type
1	6,87	n.a.	10,494	2,030	2,19	n.a.	BMB*
2	9,02	n.a.	320,444	90,810	97,81	n.a.	BMB
Total:			330,939	92,840	100,00	0,000	

7-Phenyl-6,7-dihydro-5*H*-pyrrolo[3,4-*b*]pyrazin-5-one (SI-7)

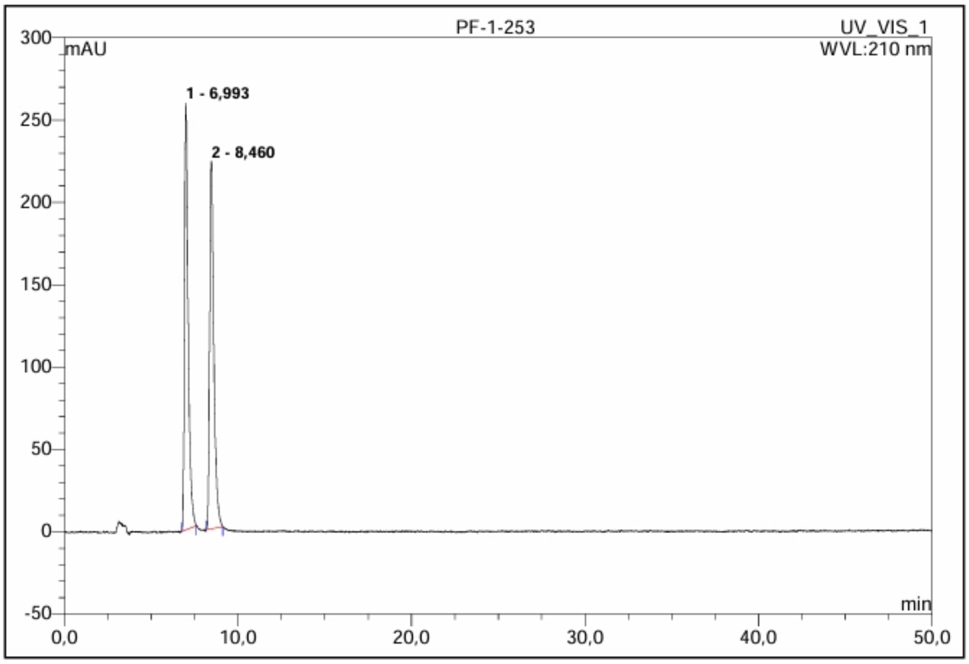

No.	Ret.Time min	Peak Name	Height mAU	Area mAU*min	Rel.Area %	Amount	Type
1	6,99	n.a.	259,144	62,670	50,13	n.a.	BMB
2	8,46	n.a.	223,197	62,336	49,87	n.a.	BMB
Total:			482,341	125,006	100,00	0,000	

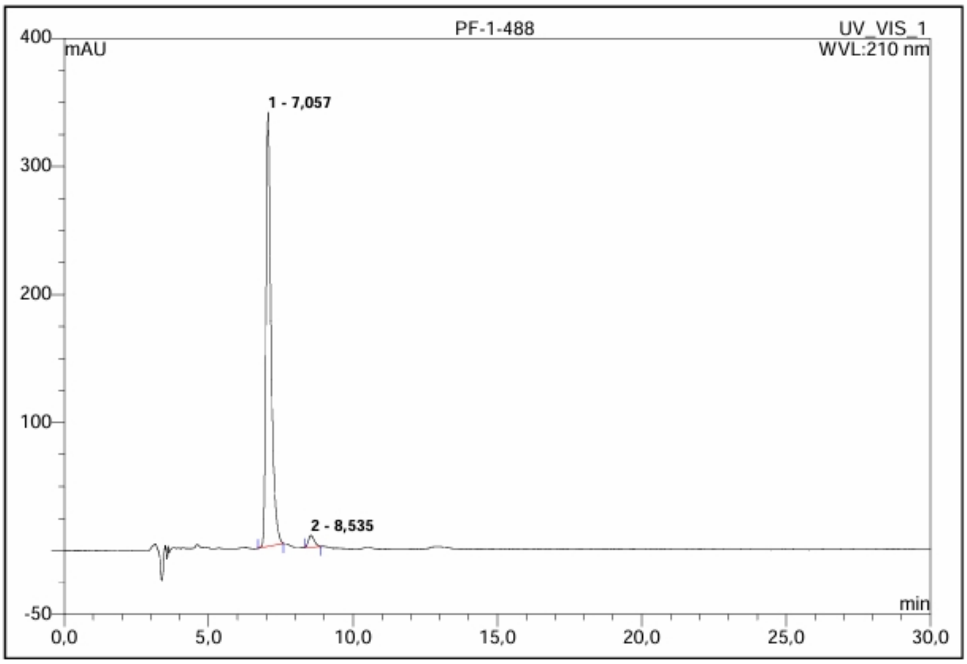

No.	Ret.Time min	Peak Name	Height mAU	Area mAU*min	Rel.Area %	Amount	Type
1	7,06	n.a.	339,253	70,533	96,99	n.a.	BMB
2	8,54	n.a.	9,191	2,188	3,01	n.a.	BMB*
Total:			348,445	72,721	100,00	0,000	

***tert*-Butyl (*R*)-5-(cyclopentylmethyl)-7-oxo-5,7-dihydro-6*H*-pyrrolo[3,4-*b*]pyrazine-6-carboxylate (6)**

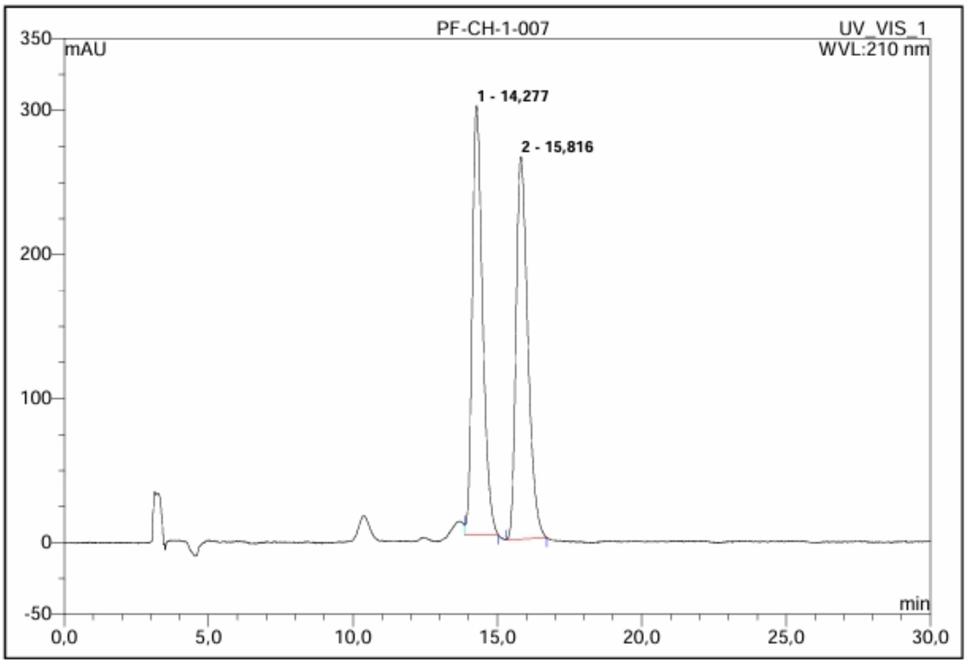

No.	Ret.Time min	Peak Name	Height mAU	Area mAU*min	Rel.Area %	Amount	Type
1	14,28	n.a.	298,160	124,104	49,83	n.a.	MB*
2	15,82	n.a.	265,376	124,945	50,17	n.a.	BMB
Total:			563,535	249,050	100,00	0,000	

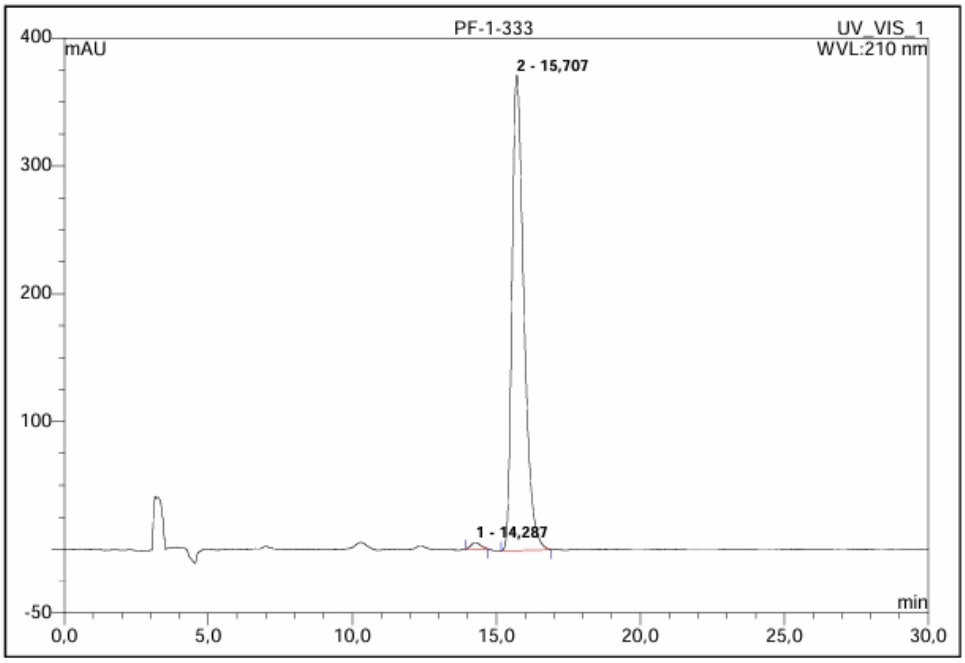

No.	Ret.Time min	Peak Name	Height mAU	Area mAU*min	Rel.Area %	Amount	Type
1	14,29	n.a.	5,221	2,021	1,12	n.a.	BMB*
2	15,71	n.a.	372,105	178,359	98,88	n.a.	BMB
Total:			377,326	180,380	100,00	0,000	

***tert*-Butyl (*R*)-(2-cyclopentyl-1-(pyrazin-2-yl)ethyl)carbamate (8)**

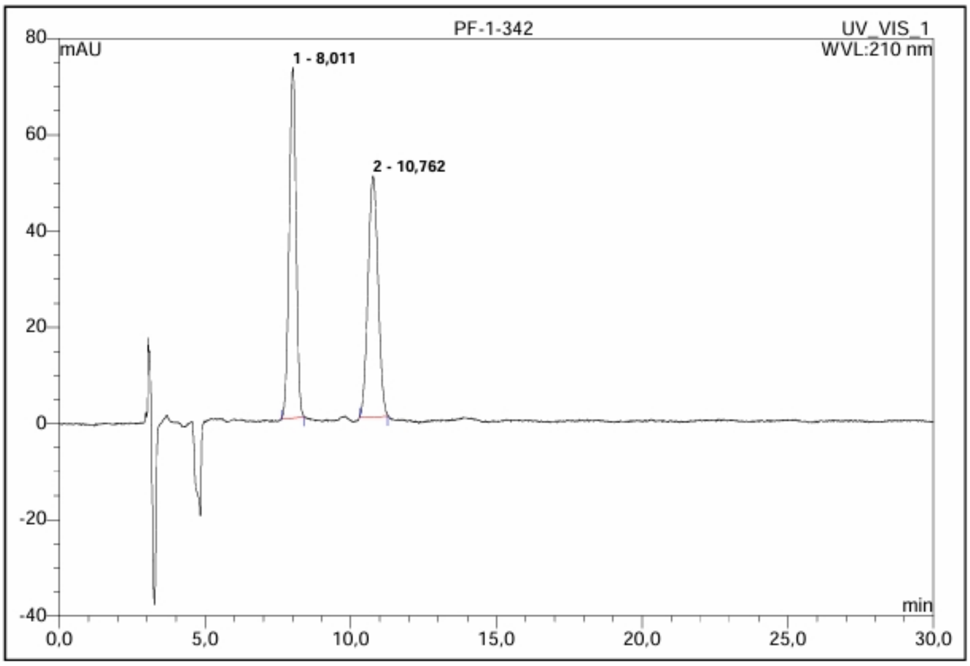

No.	Ret.Time min	Peak Name	Height mAU	Area mAU*min	Rel.Area %	Amount	Type
1	8,01	n.a.	72,860	20,018	50,02	n.a.	BMB
2	10,76	n.a.	50,095	19,999	49,98	n.a.	BMB
Total:			122,955	40,016	100,00	0,000	

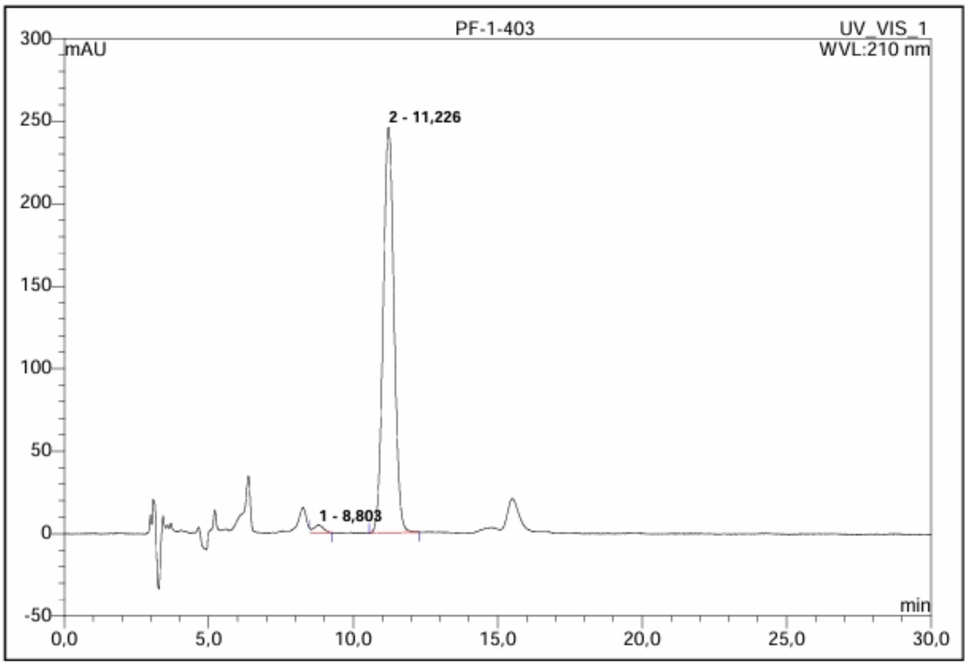

No.	Ret.Time min	Peak Name	Height mAU	Area mAU*min	Rel.Area %	Amount	Type
1	8,80	n.a.	4,831	1,875	1,73	n.a.	MB*
2	11,23	n.a.	245,649	106,401	98,27	n.a.	BMB*
Total:			250,480	108,276	100,00	0,000	

Methyl (*R*)-3-(1-((*tert*-butoxycarbonyl)amino)-2-cyclopentylethyl)pyrazine-2-carboxylate (9)

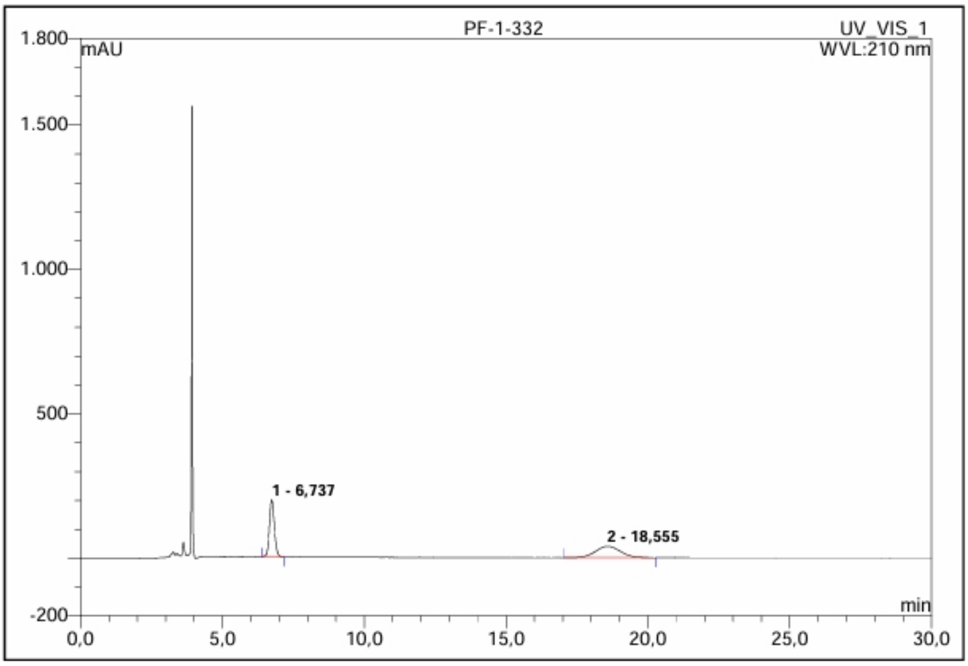

No.	Ret.Time min	Peak Name	Height mAU	Area mAU*min	Rel.Area %	Amount	Type
1	6,74	n.a.	197,794	40,228	49,98	n.a.	BMB
2	18,56	n.a.	39,442	40,252	50,02	n.a.	BMB*
Total:			237,235	80,480	100,00	0,000	

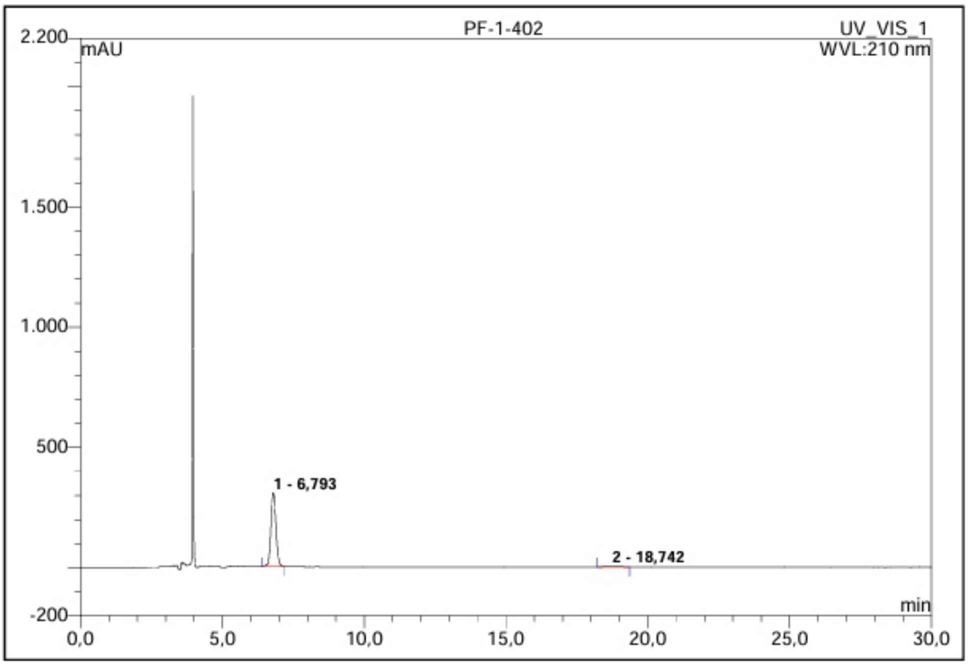

No.	Ret.Time min	Peak Name	Height mAU	Area mAU*min	Rel.Area %	Amount	Type
1	6,79	n.a.	307,078	62,991	98,37	n.a.	BM *
2	18,74	n.a.	1,666	1,046	1,63	n.a.	BMB*
Total:			308,744	64,036	100,00	0,000	

(*R*)-6,7-Dihydrooxazolo[3',2':1,2]pyrrolo[3,4-*b*]pyrazin-9(4*bH*)-one (10)

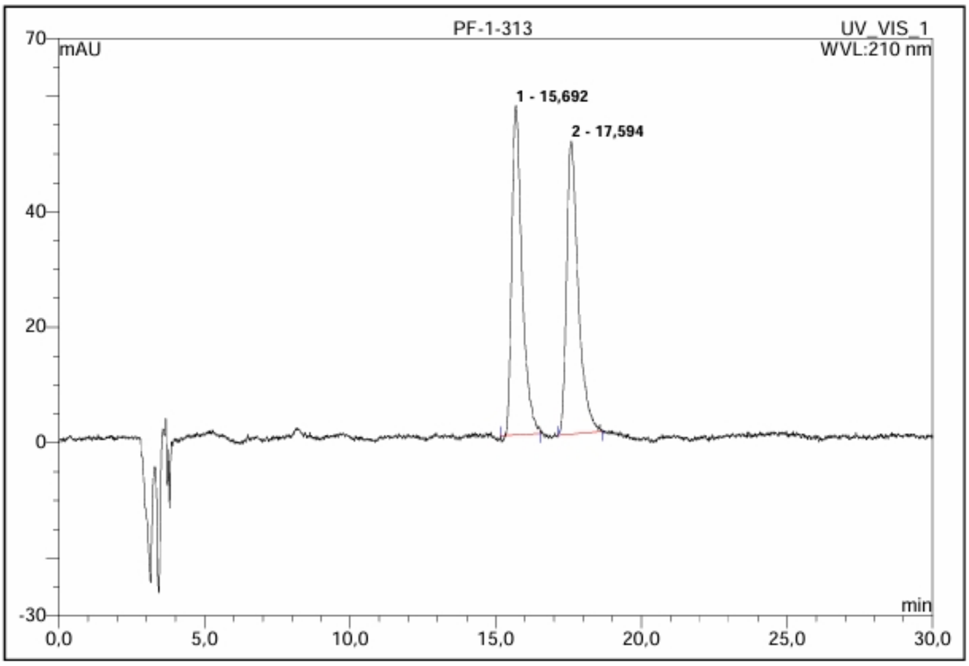

No.	Ret.Time min	Peak Name	Height mAU	Area mAU*min	Rel.Area %	Amount	Type
1	15,69	n.a.	57,006	23,189	49,60	n.a.	BMB*
2	17,59	n.a.	50,711	23,563	50,40	n.a.	BMB*
Total:			107,718	46,752	100,00	0,000	

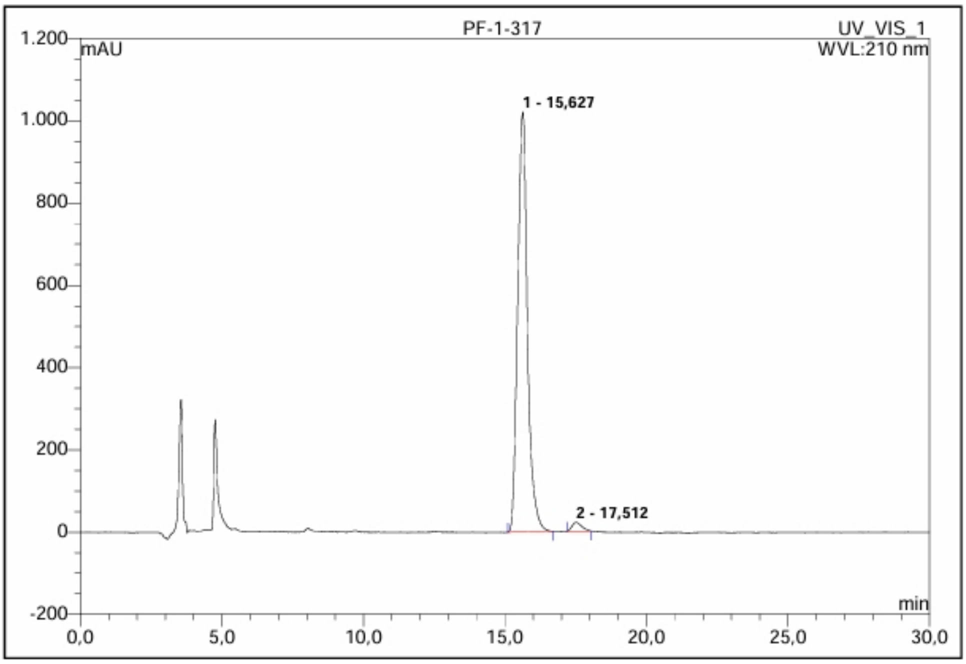

No.	Ret.Time min	Peak Name	Height mAU	Area mAU*min	Rel.Area %	Amount	Type
1	15,63	n.a.	1020,347	408,441	97,97	n.a.	BMB*
2	17,51	n.a.	21,078	8,477	2,03	n.a.	BMB*
Total:			1041,425	416,917	100,00	0,000	

(*R*)-7-((*tert*-Butyldimethylsilyl)oxy)-6-(5-chloropyridin-2-yl)-6,7-dihydro-5*H*-pyrrolo[3,4-*b*]pyrazin-5-one (11)

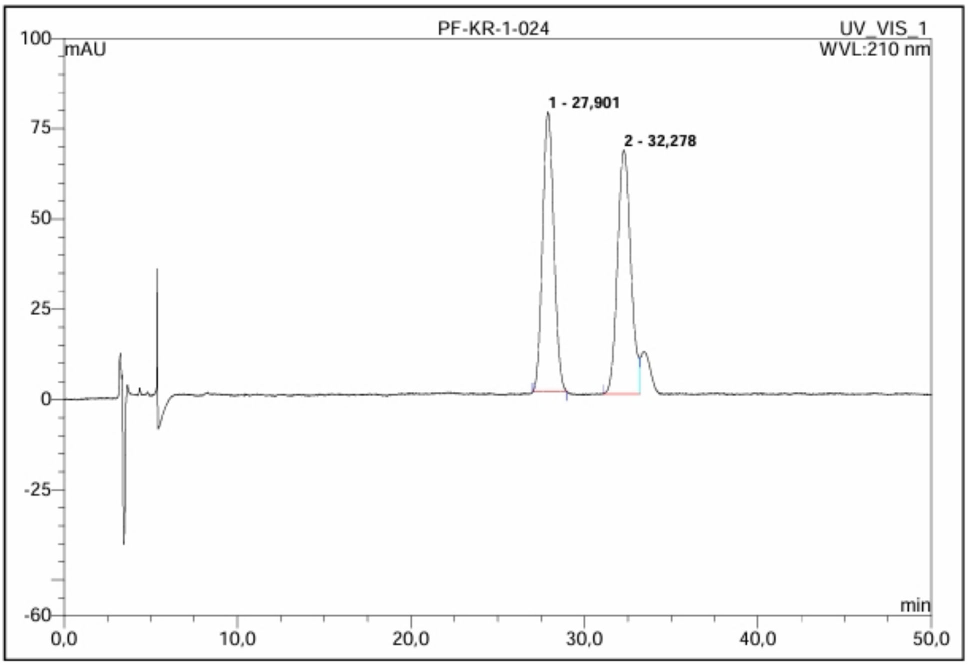

No.	Ret.Time min	Peak Name	Height mAU	Area mAU*min	Rel.Area %	Amount	Type
1	27,90	n.a.	77,554	58,260	49,17	n.a.	BMB
2	32,28	n.a.	67,611	60,233	50,83	n.a.	BM *
Total:			145,165	118,493	100,00	0,000	

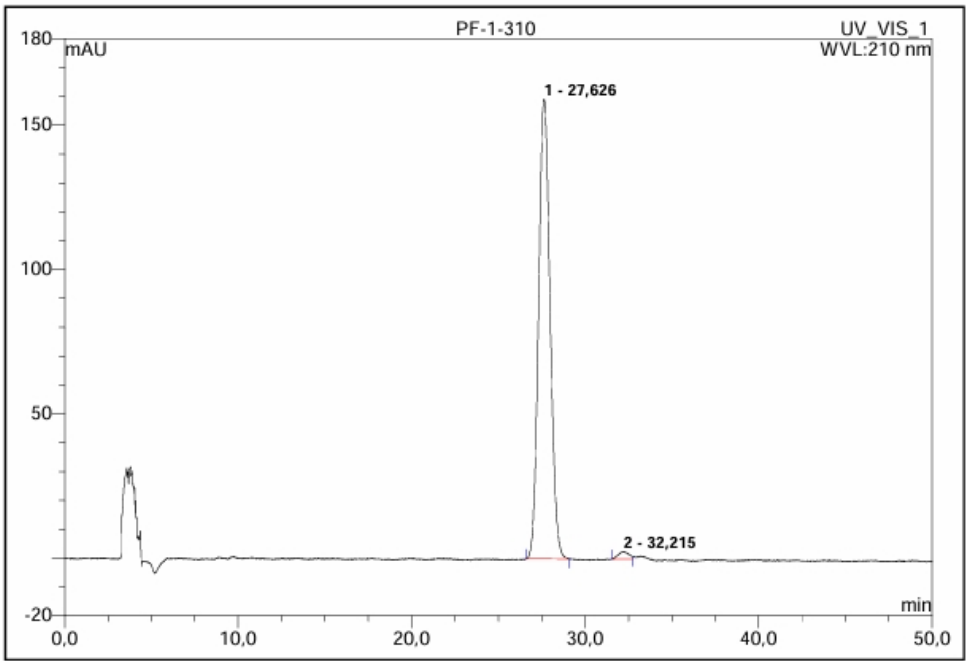

No.	Ret.Time min	Peak Name	Height mAU	Area mAU*min	Rel.Area %	Amount	Type
1	27,63	n.a.	159,289	121,988	98,55	n.a.	BMB*
2	32,22	n.a.	2,618	1,799	1,45	n.a.	BM *
Total:			161,907	123,787	100,00	0,000	

16. References

[1] Pérard-Viret, J.; Prangé, T.; Tomas, A.; Royer, J. A Simple and Efficient Asymmetric Synthesis of 3-Alkyl-Isoindolin-1-ones. *Tetrahedron* **2002**, *58*, 5103–5108.

[2] Krishna, Y.; Tanaka, F. Intramolecular Formal [4 + 2] Cycloadditions: Synthesis of Spiro Isoindolinone Derivatives and Related Molecules. *Org. Lett.* **2021**, *23*, 1874–1879.

[3] Goto, T.; Utsunomiya, S.; Aiba, H.; Hayasaka, H.; Endo, M.; Watanabe, R.; Ishizaki, T.; Sato, R.; Saito, M. Synthesis of 7-Substituted 6,7-Dihydro-5*H*-pyrrolo[3,4-*b*]pyridin-5-ones. Reaction of 7-Hydroxy Derivatives with Nucleophiles. *Bull. Chem. Soc. J.* **1991**, *64*, 1901-1910.

[4] Coleman, R. S.; Liu, P.-H. Divergent and Stereocontrolled Synthesis of the Enamide Side Chains of Oximidines I/II/III, Salicylihalamides A/B, Lobatamides A/D, and CJ-12,950. *Org. Lett.* **2004**, *6*, 577–580.

[5] Krause, M.; Rouleau, A.; Stark, H.; Garbarg, M.; Schwartz, J.; Schunack, W. Structure-activity relationships of novel azomethine prodrugs of the histamine H3-receptor agonist (*R*)-alpha-methylhistamine: from alkylaryl to substituted diaryl derivatives. *Pharmazie* **1996**, *51,* 720-726.

[6] Xu, D.; Chiaroni, A.; Fleury, M.-B.; Largeron, M. Electrochemically Induced Cascade Reaction for the Assembly of Libraries of Biologically Relevant 1,4-Benzoxazine Derivatives. *J. Org. Chem.* **2006**, *71,* 6374–6381.

[7] Bauer, A.; Westkämper, F.; Grimme, S.; Bach, T. Catalytic Enantioselective Reactions Driven by Photoinduced Electron Transfer. *Nature* **2005**, *436*, 1139–1140.

[8] Müller, C.; Bauer, A.; Bach, T. Light-Driven Enantioselective Organocatalysis. *Angew. Chem. Int. Ed* **2009**, *48*, 6640 – 6642.

[9] Bauer, A.; Bach, T. Assignment of the Absolute Configuration of 7-Substituted 3-Azabicyclo[3.3.1]Nonan-2-Ones by NMR-Titration Experiments. *Tetrahedron: Asymmetry*, **2004**, *15*, 3799–3803.

[10] *APEX4 Suite of Crystallographic Software, Version 2021-10.0,* Bruker AXS Inc., Madison, Wisconsin, USA, **2021**.

[11] Bruker, *SAINT*, *V8.40B*, Bruker AXS Inc., Madison, Wisconsin, USA.

[12] Krause, L.; Herbst-Irmer, R.; Sheldrick, G. M.; Stalke, D. Comparison of silver and molybdenum microfocus X-ray sources for single-crystal structure determination. *J. Appl. Cryst.* **2015**, *48*, 3–10, doi:10.1107/S1600576714022985.

[13] Sheldrick, G. M. SHELXT– Integrated Space-Group and Crystal-Structure Determination. *Acta Cryst.* **2015**, *A71*, 3–8, doi:10.1107/S2053273314026370.

[14] Sheldrick, G. M. Crystal Structure Refinement withSHELXL. *Acta Cryst.* **2015**, *C71*, 3–8, doi:10.1107/S2053229614024218.

[15] Hübschle, C. B.; Sheldrick, G. M.; Dittrich, B. ShelXle: A Qt Graphical User Interface forSHELXL. *J. Appl. Cryst.* **2011**, *44*, 1281–1284, doi:10.1107/S0021889811043202.

[16] Ed. E. Prince, *International Tables for Crystallography Volume C, Mathematical, Physical and Chemical Tables*, International Union of Crystallography, Chester, England, **2006**, 500–502; 219–222; 193–199.

[17] Groom, C. R.; Bruno, I. J.; Lightfoot, M. P.; Ward, S. C. The Cambridge Structural Database. *Acta Cryst.* **2016**, *B72*, 171–179, doi:10.1107/S2052520616003954.

[18] D. Kratzert, *FinalCif*, *V125*, https://dkratzert.de/finalcif.html.

[19] Bannwarth, C.; Ehlert, S.; Grimme, S. GFN2-xTB - An Accurate and Broadly Parametrized Self-Consistent Tight-Binding Quantum Chemical Method with Multipole Electrostatics and Density-Dependent Dispersion Contributions. *J. Chem. Theory Comput.* **2019**, *15* (3), 1652–1671.

[20] Semiempirical Extended Tight-Binding Program Package (`xtb`). https://github.com/grimme-lab/xtb. Accessed: 2024-18-06.

[21] Ehlert, S.; Stahn, M.; Spicher, S.; Grimme, S. Robust and Efficient Implicit Solvation Model for Fast Semiempirical Methods. *J. Chem. Theory Comput.* **2021**, *17* (7), 4250-4261.

[22] Pracht, P.; Bohle, F.; Grimme, S. Automated Exploration of the Low-Energy Chemical Space with Fast Quantum Chemical Methods. *Phys. Chem. Chem. Phys.* **2020**, *22*, 7169–7192.

[23] Conformer-Rotamer Ensemble Sampling Tool (CREST). https://github.com/grimme-lab/crest. Accessed: 18.06.2024.

[24] Grimme, S.; Bannwarth, C.; Shushkov, P. A Robust and Accurate Tight-Binding Quantum Chemical Method for Structures, Vibrational Frequencies, and Noncovalent Interactions of Large Molecular Systems Parametrized for All Spd-Block Elements (Z=1-86). *J. Chem. Theory Comput.* **2017**, *13*, 1989-2009.

[25] Grimme, S.; Bannwarth, C.; Caldeweyher, E.; Pisarek, J.; Hansen, A. A General Intermolecular Force Field Based on Tight-Binding Quantum Chemical Calculations. *J. Chem. Phys.* **2017**, *147*, 161708–161708.

[26] Neese, F. The ORCA Program System. *WIREs Comput. Mol. Sci.* **2012**, *2*, 73-78-73–78.

[27] Neese, F. Software Update: The ORCA Program System-Version 5.0. *Wiley Interdiscip. Rev.-Comput. Mol. Sci,* **2022**, *12* (5), e1606.

[28] Seritan, S.; Bannwarth, C.; Fales, B. S.; Hohenstein, E. G.; Isborn, C. M.; Kokkila-Schumacher, S. I. L.; Li, X.; Liu, F.; Luehr, N.; Snyder Jr., J. W.; Song, C.; Titov, A. V.; Ufimtsev, I. S.; Wang, L.-P.; Martínez, T. J. TeraChem: A Graphical Processing Unit-Accelerated Electronic Structure Package for Large-Scale Ab Initio Molecular Dynamics. *WIREs Comput. Mol. Sci.* **2021**, *11* (2), e1494.

[29] Seritan, S.; Bannwarth, C.; Fales, B. S.; Hohenstein, E. G.; Kokkila-Schumacher, S. I. L.; Luehr, N.; Snyder, J. W.; Song, C.; Titov, A. V.; Ufimtsev, I. S.; Martínez, T. J. TeraChem: Accelerating Electronic Structure and Ab Initio Molecular Dynamics with Graphical Processing Units. *J. Chem. Phys.* **2020**, *152* (22), 224110.

[30] Steinbach, P.; Bannwarth, C. Combining Low-Cost Electronic Structure Theory and Low-Cost Parallel Computing Architecture. *Phys. Chem. Chem. Phys.* **2024**, *26*, 16567–16578.

[31] Grimme, S.; Brandenburg, J. G.; Bannwarth, C.; Hansen, A. Consistent Structures and Interactions by Density Functional Theory with Small Atomic Orbital Basis Sets. *J. Chem. Phys.* **2015**, *143*, 054107.

[32] Barone, V.; Cossi, M. Quantum Calculation of Molecular Energies and Energy Gradients in Solution by a Conductor Solvent Model. *J. Phys. Chem. A* **1998**, *102*, 1995-2001.

[33] Weigend, F.; Ahlrichs, R. Balanced Basis Sets of Split Valence, Triple Zeta Valence and Quadruple Zeta Valence Quality for H to Rn: Design and Assessment of Accuracy. *Phys. Chem. Chem. Phys.* **2005**, *7*, 3297-3305.

[34] Perdew, J. P.; Burke, K.; Ernzerhof, M. Generalized Gradient Approximation Made Simple. *Phys. Rev. Lett.* **1996**, *77*, 3865-3868-3865–3868.

[35] Grimme, S.; Antony, J.; Ehrlich, S.; Krieg, H. A Consistent and Accurate Ab Initio Parametrization of Density Functional Dispersion Correction (DFT-D) for the 94 Elements H-Pu. *J. Chem. Phys.* **2010**, *132*, 154104.

[36] Grimme, S.; Ehrlich, S.; Goerigk, L. Effect of the Damping Function in Dispersion Corrected Density Functional Theory. *J. Comput. Chem.* **2011**, *32*, 1456–1465.

[37] Kruse, H.; Grimme, S. A Geometrical Correction for the Inter- and Intra-Molecular Basis Set Superposition Error in Hartree-Fock and Density Functional Theory Calculations for Large Systems. *J. Chem. Phys.* **2012**, *136* (15), 154101.

[38] Garcia-Ratés, M.; Neese, F. Effect of the Solute Cavity on the Solvation Energy and Its Derivatives within the Framework of the Gaussian Charge Scheme. *J. Comput. Chem.* **2020**, *41* (9), 922–939.

[39] Zhao, Y.; Truhlar, D. G. Design of Density Functionals That Are Broadly Accurate for Thermochemistry, Thermochemical Kinetics, and Nonbonded Interactions. *J. Phys. Chem. A* **2005**, *109* (25), 5656–5667.

[40] Caldeweyher, E.; Ehlert, S.; Hansen, A.; Neugebauer, H.; Spicher, S.; Bannwarth, C.; Grimme, S. A generally applicable atomic-charge dependent London dispersion correction. *J. Chem. Phys.* **2019**, *150*, 154122.

[41] Vahtras, O.; Almlöf, J.; Feyereisen, M. W. Integral Approximations for LCAO-SCF Calculations. *Chem. Phys. Lett.* **1993**, *213* (5), 514–518.

[42] Neese, F. An Improvement of the Resolution of the Identity Approximation for the Formation of the Coulomb Matrix. *J. Comput. Chem.* **2003**, *24* (14), 1740–1747.

[43] Weigend, F. Accurate Coulomb-Fitting Basis Sets for H to Rn. *Phys. Chem. Chem. Phys.* **2006**, *8* (9), 1057–1057.

[44] Neese, F.; Wennmohs, F.; Hansen, A.; Becker, U. Efficient, Approximate and Parallel Hartree–Fock and Hybrid DFT Calculations. A 'Chain-of-Spheres' Algorithm for the Hartree–Fock Exchange. *Chem. Phys.* **2009**, *356* (1), 98-109.

[45] Grimme, S. Supramolecular Binding Thermodynamics by Dispersion-Corrected Density Functional Theory. *Chem. Eur. J.* **2012**, *18* (32), 9955–9964.

[46] Spicher, S.; Grimme, S. Efficient Computation of Free Energy Contributions for Association Reactions of Large Molecules. *J. Phys. Chem. Lett.* **2020**, *11* (16), 6606–6611.

[47] Bannwarth, C.; Caldeweyher, E.; Ehlert, S.; Hansen, A.; Pracht, P.; Seibert, J.; Spicher, S.; Grimme, S. Extended Tight-Binding Quantum Chemistry Methods. *WIREs Comput. Mol. Sci.* **2021**, *11* (2), e1493.

[48] Neugebauer, H.; Bädorf, B.; Ehlert, S.; Hansen, A.; Grimme, S. High-Throughput Screening of Spin States for Transition Metal Complexes with Spin-Polarized Extended Tight-Binding Methods. *J. Comput. Chem.* **2023**, *44* (27), 2120–2129.

[49] Moradi, S.; Tomann, R.; Hendrix, J.; Head-Gordon, M.; Stein, C. J. Spin parameter optimization for spin-polarized extended tight-binding methods. *J. Comput. Chem.* **2024**, *45*, 2786–2792.

[50] Kutta, R. J.; Großkopf, J.; van Staalduinen, N.; Seitz, A.; Pracht, P.; Breitenlechner, S.; Bannwarth, C.; Nuernberger, P.; Bach, T. Multifaceted View on the Mechanism of a Photochemical Deracemization Reaction. *J. Am. Chem. Soc.* **2023**, *145* (4), 2354–2363.

[51] Baker, J. An Algorithm for the Location of Transition States. *J. Comput. Chem.* **1986**, *7* (4), 385–395.

[52] Pulay, P. Convergence Acceleration of Iterative Sequences. the Case of Scf Iteration. *Chem. Phys. Lett.* **1980**, *73* (2), 393–398.

[53] Pulay, P. Improved SCF Convergence Acceleration. *J. Comput. Chem.* **1982**, *3* (4), 556–560.

[54] Ishida, K.; Morokuma, K.; Komornicki, A. The Intrinsic Reaction Coordinate. An Ab Initio Calculation for HNC→HCN and H^- + CH4→CH4 + H^-. *J. Chem. Phys.* **2008**, *66* (5), 2153–2156.

[55] Pracht, P.; Bannwarth, C. Finding Excited-State Minimum Energy Crossing Points on a Budget: Non-Self-Consistent Tight-Binding Methods. *J. Phys. Chem. Lett.* **2023**, *14* (19), 4440–4448.

[56] Pracht, P.; Bannwarth, C. Fast Screening of Minimum Energy Crossing Points with Semiempirical Tight-Binding Methods. *J. Chem. Theory Comput.* **2022**, *18* (10), 6370–6385.

[57] Staalduinen, N. van; Bannwarth, C. MolBar: A Molecular Identifier for Inorganic and Organic Molecules with Full Support of Stereoisomerism. *Digit. Discov.* **2024**.

[58] MolBar: A Molecular Identifier for Inorganic and Organic Molecules with Full Support of Stereoisomerism. https://git.rwth-aachen.de/bannwarthlab/molbar. Accessed: 7.30.2024.

[59] Herbert, J. M. Dielectric Continuum Methods for Quantum Chemistry. *WIREs Computational Molecular Science* **2021**, *11* (4), e1519.

[60] Corni, S.; Cammi, R.; Mennucci, B.; Tomasi, J. Electronic Excitation Energies of Molecules in Solution within Continuum Solvation Models: Investigating the Discrepancy between State-Specific and Linear-Response Methods. *J. Chem. Phys* **2005**, *123* (13), 134512.

[61] Martin, R. L. Natural Transition Orbitals. *J. Chem. Phys.* **2003**, *118* (11), 4775-4777.

[62] Grimme, S.; Waletzke, M. A Combination of Kohn–Sham Density Functional Theory and Multi-Reference Configuration Interaction Methods. *J. Chem. Phys.* **1999**, *111* (13), 5645–5655.

[63] Dombrowski, D. R.; Schulz, T.; Kleinschmidt, M.; Marian, C. M. R2022: A DFT/MRCI Ansatz with Improved Performance for Double Excitations. *J. Phys. Chem. A* **2023**, *127* (8), 2011–2025.

[64] TURBOMOLE V7.6 2020, a development of University of Karlsruhe and Forschungszentrum Karlsruhe GmbH, 1989-2007, TURBOMOLE GmbH, since 2007; available from http://www.turbomole.com.

[65] Becke, A. D. A New Mixing of Hartree–Fock and Local Density-Functional Theories. *J. Chem. Phys.* **1993**, *98*, 1372-1377.

[66] GitHub cefine. https://github.com/grimme-lab/cefine. Accessed: 01.12.2023.

[67] Vahtras, O.; Almlöf, J.; Feyereisen, M. Integral approximations for LCAO-SCF calculations. *Chem. Phys. Lett.* **1993**, *213*, 514–518.

[68] Marian, C. M.; Heil, A.; Kleinschmidt, M. The DFT/MRCI Method. *WIREs Comput. Mol. Sci.* **2019**, *9* (2), e1394.

[69] Selenius, E.; Sigurdarson, A. E.; Schmerwitz, Y. L. A.; Levi, G. Orbital-Optimized Versus Time-Dependent Density Functional Calculations of Intramolecular Charge Transfer Excited States. *J. Chem. Theory Comput.* **2024**, *20* (9), 3809–3822.

[70] Gilbert, A. T. B.; Besley, N. A.; Gill, P. M. W. Self-Consistent Field Calculations of Excited States Using the Maximum Overlap Method (MOM). *J. Phys. Chem. A* **2008**, *112* (50), 13164–13171.

[71] Nair, R. N.; Bannister, T. D. One-Pot Directed Alkylation/Deprotection Strategy for the Synthesis of Substituted Pyrrole[3,4-*d*]Pyridazinones. *Eur. J. Org. Chem.* **2015**, 1764–1770.

[72] Buchelt, C.; Zuber, J.; Bach, T. Intramolecular Cobalt Porphyrin-Catalyzed Alkylation of 1-Isoindolinones by Site-Selective Insertion into a C(sp^3)–H Bond.*Org. Lett.* **2024**, *26*, 7302–7306.

[73 Camparini, A.; Ponticelli, F.; Tedeschi, P. Syntheses and Reactivities of 3-Methylisoxazolo[4,5-b]Pyridines. *J. Chem. Soc., Perkin Trans. 1*, **1982**, 2391-2394.

[74] Goto, T.; Saito, M.; Sato, R. The Thermally-Controlled Chemoselective Reduction of 5*H*-Pyrrolo[3,4-*b*]Pyridine-5,7(6*H*)-Dione with Sodium Borohydride. *Bull. Chem. Soc. J.* **1987**, *60*, 4178-4180.

[75] Zetschok, D.; Heieck, L.; Wennemers, H. Decarboxylative Organocatalyzed Addition Reactions of Fluoroacetate Surrogates for the Synthesis of Fluorinated Oxindoles. *Org. Lett.* **2021**, *23*, 1753–1757.

[76] Yao, T.; Liang, X.; Guo, Z.; Yang, D. Highly stereoselective synthesis of (Z)-3-methoxy-1-methyleneisoindoles via DMAP catalyzed cyclization of methyl 2-alkynylbenzimidates. Tetrahedron **2019**, *75,* 3088-3100

[77] Großkopf, J.; Heidecker, A. A.; Bach, T. Photochemical Deracemization of 3-Substituted Oxindoles. *Angew. Chem. Int. Ed.* **2023**, *62*, e202305274.

[78] Brenner, M.; Seebach, D. Enantioselective Preparation of γ -Amino Acids and γ -Lactams from Nitro Olefins and Carboxylic Acids, with the Valine-Derived 4-Isopropyl-

5,5-diphenyl-1,3-oxazolidin-2-one as an Auxiliary. *Helv. Chim. Acta*, **1999**, *82*, 2365-2379.

[79] Adamczyk M.; Akireddy S.; Reddy R. Transformation of Vitamin B_6 to (+)-Deoxypyridinoline, a useful Biochemical Marker for Diagnosis of Bone Diseases. *Tetrahedron* **2000**, *56*, 2379-2390.

[80] Brenna, E.; Fuganti, C.; Serra, S. A New Two Step Route to 1-Hydroxy-9H-3-Carbazolecarboxylic Acid Derivatives from 3-Formylindole. Application to the Synthesis of Mukonine. *Tetrahedron* **1998**, *54*, 1585-1588.

[81] Di Mola, A.; Gatta, E.; Petronzi, C.; Cupello, A.; De Caprariis, P.; Robello, M.; Massa, A.; Filosa, R. Synthesis and pharmacological evaluation of functionalized isoindolinones on GABA-activated chloride currents in rat cerebellum granule cells in culture. *Bioorg. Med. Chem. Lett.* **2016**, *26*, 5284-5289.
